# Thermodynamic Hydricity of Small Borane Clusters and Polyhedral *closo*-Boranes †

**DOI:** 10.3390/molecules25122920

**Published:** 2020-06-25

**Authors:** Igor E. Golub, Oleg A. Filippov, Vasilisa A. Kulikova, Natalia V. Belkova, Lina M. Epstein, Elena S. Shubina

**Affiliations:** 1 A. N. Nesmeyanov Institute of Organoelement Compounds and Russian Academy of Sciences (INEOS RAS), 28 Vavilova St, 119991 Moscow, Russia; h-bond@ineos.ac.ru (O.A.F.); kullisa99@gmail.com (V.A.K.); nataliabelk@ineos.ac.ru (N.V.B.); epst@ineos.ac.ru (L.M.E.); 2 Faculty of Chemistry, M.V. Lomonosov Moscow State University, 1/3 Leninskiye Gory, 119991 Moscow, Russia

**Keywords:** polyhedral boranes, borane clusters, borohydrides, Lewis acidity, hydride donating ability, DFT calculations

## Abstract

Thermodynamic hydricity (HDA^MeCN^) determined as Gibbs free energy (ΔG°[H]^−^) of the H^−^ detachment reaction in acetonitrile (MeCN) was assessed for 144 small borane clusters (up to 5 boron atoms), polyhedral *closo*-boranes dianions [B_n_H_n_]^2−^, and their lithium salts Li_2_[B_n_H_n_] (n = 5–17) by DFT method [M06/6-311++G(d,p)] taking into account non-specific solvent effect (SMD model). Thermodynamic hydricity values of diborane B_2_H_6_ (HDA^MeCN^ = 82.1 kcal/mol) and its dianion [B_2_H_6_]^2−^ (HDA^MeCN^ = 40.9 kcal/mol for Li_2_[B_2_H_6_]) can be selected as border points for the range of borane clusters’ reactivity. Borane clusters with HDA^MeCN^ below 41 kcal/mol are strong hydride donors capable of reducing CO_2_ (HDA^MeCN^ = 44 kcal/mol for HCO_2_^−^), whereas those with HDA^MeCN^ over 82 kcal/mol, predominately neutral boranes, are weak hydride donors and less prone to hydride transfer than to proton transfer (e.g., B_2_H_6_, B_4_H_10_, B_5_H_11_, etc.). The HDA^MeCN^ values of *closo*-boranes are found to directly depend on the coordination number of the boron atom from which hydride detachment and stabilization of quasi-borinium cation takes place. In general, the larger the coordination number (CN) of a boron atom, the lower the value of HDA^MeCN^.

## 1. Introduction

Boron-based chemistry is vast, diverse, and fascinating due to the ability of boron to form electron-deficient structures of various shapes (such as cages, clusters, etc.) with delocalized electrons and multicenter bonding. Boron hydrides, i.e., boranes (e.g., BH_4_^−^, [B_3_H_8_]^−^, [B_n_H_n_]^2−^ n = 6–12), are of great interest because of their use as ligands in inorganic chemistry [1,2,3,4,5], as building blocks in material chemistry [3,6,7,8], and as materials for the energetic purposes—components of batteries [9,10], rocket fuels [11,12,13] and systems of hydrogen storage [14,15,16,17]. Polyhedral boranes are widely used as sources of boron (^10^B) in boron neutron capture cancer therapy [18,19,20,21,22,23], in the creation of luminescent materials [24,25,26], thermally stable polymers [5], liquid crystals and nonlinear optical materials [27,28], as well as precursors of nanostructured materials [29,30].

Thermodynamic hydricity, i.e., hydride donating ability (HDA), determined as Gibbs free energy (ΔG°[H]^−^) for the reaction of hydride ion, H^−^, detachment, is a very important characteristic of transition metal hydrides [31] and main group hydrides [32,33,34] that describes their reactivity and is used for the rational design of catalytic reactions. In our recent work, we have demonstrated the existence of an inversely proportional dependence of the hydride-transfer ability of Li[L_3_B–H] on Lewis acidity of L_3_B [34]. High values of HDA indicate high Lewis acidity of parent borane L_3_B, whereas low HDA values indicate high hydride transfer ability of Li[L_3_B–H]. For that reason, the hydride donating ability (HDA) of boranes can be used as a measure of the Lewis acidity of parent neutral borane or boron cations.

However, there are significant problems in the experimental determination of the thermodynamic hydricity for hydrides of main group elements (E–H, where E = Si, C, B, Al) because of their instability in polar solvents (MeCN, H_2_O), which are typically used for that [32,35]. Besides, the E–H bond of main group hydrides is characterized by low polarizability compared to transition metal hydrides and, therefore, in many cases, the detachment of the hydride ion (H^−^) is challenging even in the presence of a large excess of strong Lewis acid. Due to these problems, there is a huge gap in our knowledge about the reactivity of boranes towards hydride transfer [32,35,36].

In our previous paper [34], we focused on the DFT investigation of thermodynamic hydricity of tetracoordinate borohydrides Li[L_3_B–H]. However, despite the attempts to evaluate the electron-donating properties in polyhedral boranes by ^1^H NMR [37], there is a lack of knowledge of the reactivity of B–H bond in small borane clusters and polyhedral boron hydrides [36]. Thus, in this paper, we report on the results of the DFT analysis of thermodynamic hydricity in MeCN (HDA^MeCN^) for both well-known, structurally characterized boranes and prospective reaction intermediates, some of which were previously known from theoretical works.

## 2. Results and Discussion

Borane clusters (such as B_2_H_6_, [B_2_H_7_]^−^, [B_3_H_8_]^−^, [B_12_H_12_]^2−^, [B_10_H_10_]^2−^, etc.) were discovered as intermediate products of borohydrides thermal decomposition [38,39,40,41,42,43,44,45,46,47,48,49,50]. These compounds are widely used in direct synthesis of other boranes [51,52], organoboron compounds [53], and new materials [5]. For example, hydride ion abstraction from borane anions (such as BH_4_^−^, [B_3_H_8_]^−^, [B_4_H_9_]^−^, etc.) by Lewis acids BX_3_ (X = F, Cl, Br) is the most convenient route for the preparation of higher boranes [54,55]. Many small borane clusters are highly reactive and unstable species, so their reactivity is hard to assess experimentally. Others, like polyhedral boron hydrides, require the presence of an excess of Lewis or Brønsted acids for generation of boron-centered quasi-borinium cations [36]. Thus, the reactivity of such compounds can be characterized in terms of their ability to hydride transfer as thermodynamic hydricity (HDA) through DFT calculations [32,34]. 

In this paper, we performed the DFT calculations of the thermodynamic hydricity in MeCN of small borane clusters (containing up to 5 boron atoms) and polyhedral boron hydrides [B_n_H_n_]^2−^ (n = 5–17). For anionic species we use their lithium salts to offset the effect of ionic species and to make a correct comparison with the HDA^MeCN^ values for neutral boranes. 

### 2.1. Thermodynamic Hydricity of Small Borane Clusters

**Scheme 1:**
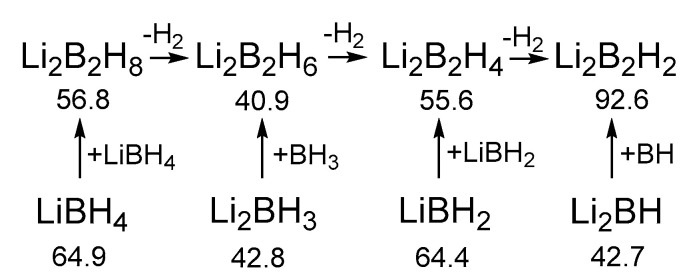
HDA^MeCN^ (in kcal/mol) for (LiBH_4_)_2_ decomposition intermediates [48] and their monomers.

Recently, the decomposition pathways of [LiBH_4_]_n_, n = 2–12 clusters were investigated by DFT calculations [48]. In the case of dimeric [LiBH_4_]_2_ clusters, the release of up to 4 equivalents of H_2_ was found, as well as [LiBH_m_]_2_ (m = 6, 4, 2) reaction intermediates, during the decomposition. Our calculations show that during the decomposition of [LiBH_4_]_2_ the first H_2_ release leads to the decrease of HDA^MeCN^ by 15.9 kcal/mol (Scheme 1). Further H_2_ release leads to an increase of HDA^MeCN^ due to formation unsaturated diboranes with double and triple bonds.

#### 2.1.1. General Pattern in Thermodynamic Hydricity of Borane Clusters

To gain insight into an effect of borane (BH_3_) aggregation in small clusters, we used the monomers LiBH_4_, Li_2_BH_3,_ LiBH_2,_ and Li_2_BH along with neutral BH_3_ and BH species to construct a homologous series of boron clusters (containing up to 5 boron atoms) by consequent addition of BH_3_ (Table 1, Schemes S1 and S2). Another two homologous series were constructed based on Li[B_2_H_3_] and B_2_H_2_. In each series there are the most stable boranes (namely BH_4_^−^, B_2_H_6_, [B_2_H_6_]^2−^, [B_3_H_8_]^−^, B_4_H_10_, [B_3_H_7_]^2−^, [B_4_H_9_]^−^, and B_5_H_11_), which are generally observed during thermal decomposition of metal borohydrides [38,39,40,41,42,43,44,45,46,47,48,49,50]. 

According to computed heats of formation [50], neural boranes (such as B_2_H_6_, B_4_H_10_, and B_5_H_11_) are less stable than anionic ones (BH_4_^−^, [B_3_H_8_]^−^ and [B_4_H_9_]^−^); however, the former may be derived from the latter as a result of hydride abstraction by Lewis acids BX_3_ (X = F, Cl, Br) [55]. This lower stability of neutral boranes compared to anionic boranes can be explained by higher Lewis acidity. In our previous research, we demonstrated that Lewis acidity of parent borane (R_3_B) can be estimated by the analysis of thermodynamic hydricity of their product of hydride addition [R_3_BH]^−^. Higher HDA^MeCN^ values of [R_3_BH]^−^ correspond to higher Lewis acidity of R_3_B.

Thermodynamic hydricity values of diborane B_2_H_6_ (HDA^MeCN^ = 82.1 kcal/mol) and its dianion [B_2_H_6_]^2−^ (HDA^MeCN^ = 40.9 kcal/mol for Li_2_[B_2_H_6_]) can be selected as border points for the range of borane clusters’ reactivity. Previously [57], the formal division of the thermodynamic hydricity scale into weak (above 80 kcal/mol), medium (between 80 and 50 kcal/mol) and strong hydride donors (less 50 kcal/mol) was suggested. 

Our study and literature analysis show that borane clusters with HDA^MeCN^ below 41 kcal/mol are strong hydride donors capable of reducing CO_2_ (HDA^MeCN^ = 44 kcal/mol for HCO_2_^−^ [31]); however, those with HDA^MeCN^ over 82 kcal/mol, predominately neutral boranes, are weak hydride donors and less prone to hydride transfer than to proton transfer (e.g., B_2_H_6_ [58], B_4_H_10_, B_5_H_11_, etc. [59]). Moreover, in higher boranes, the deprotonation of B–H–B bridges leads to B–B bond formation, in which boron is nucleophilic and susceptible to the attack of electrophiles [59]. Thus, higher borane anions could be generated by the insertion of such electrophile molecules as BH_3_, B_2_H_2_ or BH.

**Scheme 2:**
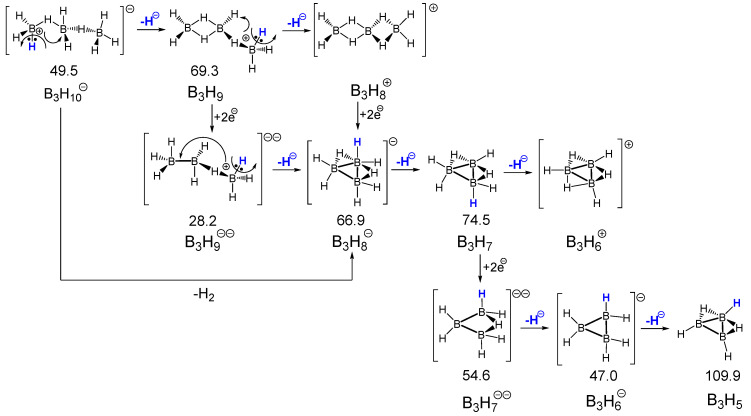
Interconversion scheme for Li[B_3_H_10_]. Lithium atoms are omitted for clarity.

Based on the data obtained for the homologous series (Table 1, Schemes S1 and S2), an evolution of thermodynamic hydricity can be traced on the example of B_3_ clusters transformations (Scheme 2). Thus, neutral boranes B_3_H_9_, B_3_H_7_, and B_3_H_5_ feature the highest Lewis acidity (highest HDA^MeCN^ values). Monoanionic boranes Li[B_3_H_10_] etc. have lower Lewis acidity than parent neutral boranes. The two-electron reduction of the neutral boranes leads to a significant decrease of HDA^MeCN^ values (by 41.1 kcal/mol for Li_2_[B_3_H_9_] and by 20.0 kcal/mol for Li_2_[B_3_H_7_]). 

**Figure 1:**
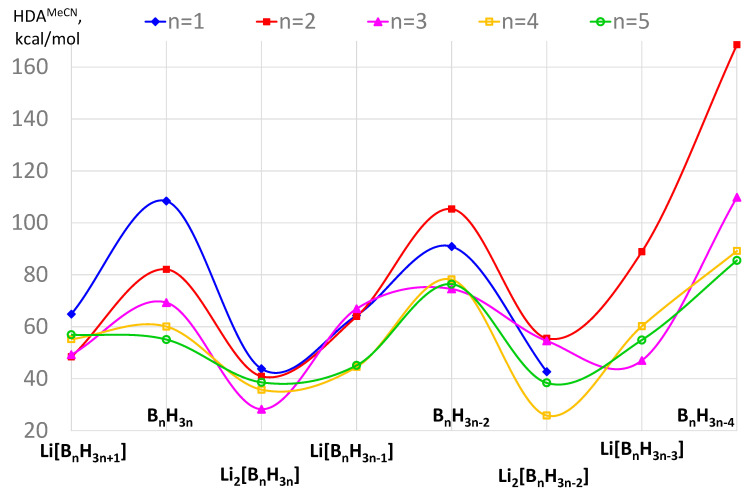
Plot of HDA^MeCN^ for borane clusters of different size.

Although it is generally acknowledged that, in clusters, borane anions’ hydridic hydrogen becomes less reactive due to increasing charge distribution across the cluster [8], our calculations show that HDA^MeCN^ values are not directly connected with the size of borane cluster (Figure S1). However, if we plot computed HDA^MeCN^ values for all series of boranes (n = 1–5; Figure 1), it appears that minimum HDA^MeCN^ values (nucleophilic boron) are typical for the dianionic species and maximum HDA^MeCN^ (electrophilic boron) for the neutral boranes, whereas borane monoanions are in the intermediate position. Since neutral transition metal hydrides are better hydride donors than cationic ones [31], it is obvious that anionic boranes should be much more better hydride donors than neutral boranes. 

An increasing size of borane cluster in neutral B_n_H_3n_, B_n_H_3n−2_, and B_n_H_3n−4_ and dianionic Li_2_[B_n_H_3n_] and Li_2_[B_n_H_3n−2_] series results in a drop of HDA^MeCN^ values. This leads to a smoothing of the extrema in the graph of HDA^MeCN^ for larger borane clusters as B_4_ and B_5_. Moreover, in the B_3_ clusters due to triangle form an effective charge distribution occurs, which leads to the systematically lower values of HDA^MeCN^.

It is worth noting that a decrease of saturation of the borane cluster results in an increase of HDA^MeCN^ that is especially pronounced for neutral diboranes.

#### 2.1.2. Features of Thermodynamic Hydricity in Homologous Series

**Scheme 3:**
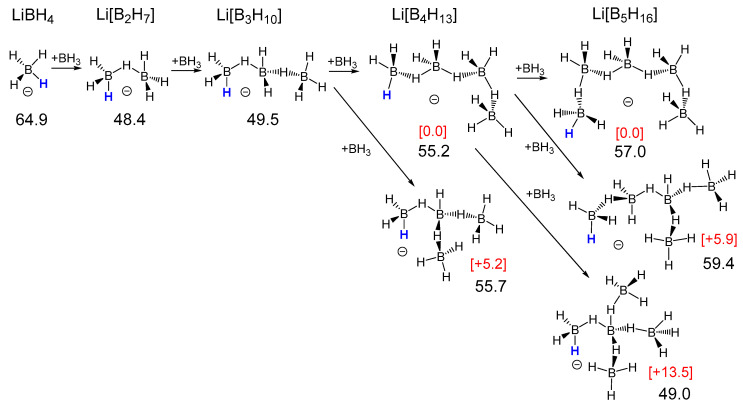
Formation of Li[B_n_H_3n+1_] series. Black numbers denote HDA^MeCN^ values for B–H groups marked by blue color (in kcal/mol), red numbers in square brackets are the Gibbs free energies (∆G°_MeCN_, in kcal/mol) of branched isomers relative to the most stable linear isomer for Li[B_4_H_13_] or Li[B_5_H_16_]. Lithium atoms are omitted for clarity.

The Li[B_n_H_3n+1_] series is formed by consecutive BH_3_ addition to tetrahydroborate-anion BH_4_^−^ (Scheme 3). The first addition of BH_3_ yields [B_2_H_7_]^−^ and leads to the HDA^MeCN^ decrease by 16.4 kcal/mol, while the subsequent BH_3_ addition leads to a gradual HDA^MeCN^ increase due to the delocalization of electrons between a greater number of atoms. 

Tetrahydroborate anions BH_4_^−^ [4,60,61,62,63,64,65,66,67,68] and [B_2_H_7_]^−^ [69,70] are used as ligands in the synthesis of transition metal complexes. Structures of other borohydride anions were accessed by DFT calculations [58,71], but only branched isomers of Li[B_4_H_13_] and Li[B_5_H_16_] have been reported [71]. However, we found that their linear isomers were energetically more favorable (see ∆G°_MeCN_, Scheme 3, Table S1). It is important to note that regardless of the structure of the isomer, the hydride detachment only occurs from the terminal BH_3_ groups, due to their increased hydricity. 

**Scheme 4:**
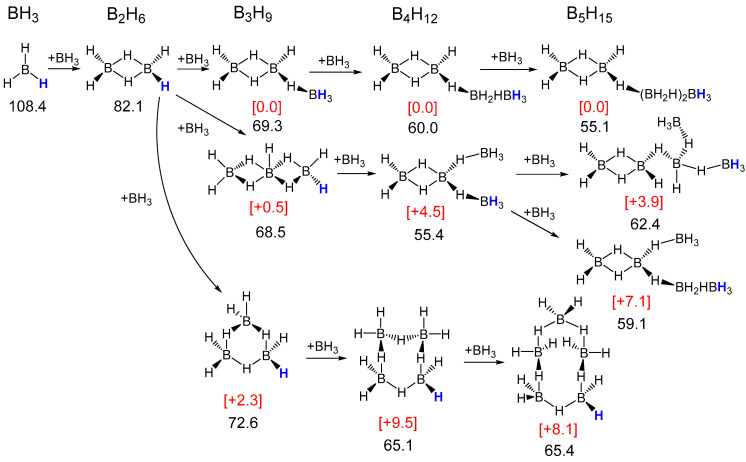
B_n_H_3n_ series. Black numbers denote HDA^MeCN^ values for B–H groups marked by blue color (in kcal/mol), red numbers in square brackets are the Gibbs free energies (∆G°_MeCN_ in kcal/mol) relative to the corresponding most stable isomer.

**Scheme 5:**
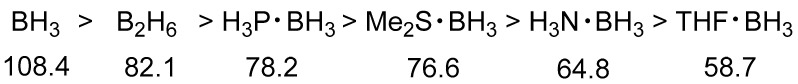
The trend of HDA^MeCN^ (in kcal/mol) for borane Lewis acid–base complexes. The HDA^MeCN^ values are taken from ref. [34].

The B_n_H_3n_ series represents neutral boranes formed by consecutive BH_3_ aggregation (Scheme 4), which causes a gradual decrease of HDA^MeCN^ at each step (from 108.4 to 55.1 kcal/mol). Borane BH_3_ has high HDA^MeCN^ value (HDA^MeCN^ = 108.4 kcal/mol) and cannot be isolated due to its high Lewis acidity; however, it can be stabilized by dimerization into diborane B_2_H_6_ molecule (HDA^MeCN^ = 82.1 kcal/mol) or by interaction with Lewis bases (Scheme 5). The Lewis acid–base complexes of BH_3_ are characterized by reduced HDA^MeCN^ and some of them, especially amine and phosphine boranes, are able to form σ-complexes with transition metals [72,73,74,75,76,77]. 

The diborane-based structures B_2_H_5_(µ-H)[(BH_2_)(µ-H)]_m_BH_3_ (m = 1–3) were found to be the most stable isomers for higher borane clusters (Scheme 4, Table S2). In previous theoretical works, cyclic structures of B_3_H_9_ and B_4_H_12_ have been reported to be the most stable isomers in the gas phase [78,79,80]. However, according to our optimization, [M06/ and MP2/6-311++G(d,p) theory levels in MeCN using SMD] is the most stable configuration for B_3_H_9_ is B_2_H_5_(µ-H)BH_3_, whereas butterfly-like and cyclic structures are slightly less favorable in terms of Gibbs free energy scale (∆G°_MeCN_ = 0.5 kcal/mol and 2.3 kcal/mol for M06 and 0.7 kcal/mol and 1.3 kcal/mol for MP2, respectively, Table S3). 

The Li_2_[B_n_H_3n_] series is formed by the consecutive addition of BH_3_ to Li_2_[BH_3_] (Scheme S3). Both [BH_3_]^2−^ [81] and [B_2_H_6_]^2−^ [82,83,84,85,86,87,88], which could be obtained by the reduction of B_2_H_6_ [89], are used as ligands in transition metal complexes. Boranes of this series formally could be obtained by two-electron reduction from their neutral analogues B_n_H_3n_, leading to a significant decrease in their thermodynamic hydricity by 24–65 kcal/mol. High reactivity of [B_2_H_6_]^2−^ towards hydride transfer (HDA^MeCN^ = 40.9 kcal/mol for Li_2_[B_2_H_6_]) results in full substitution of terminal BH hydrides in (Cp*M)_2_(κ^2^-B_2_H_6_) (M = V, Nb, Ta) in chlorinated solvents (CH_2_Cl_2_ and CHCl_3_) [86,88]. Higher boranes were not isolated, which is apparently related to their low HDA^MeCN^ (Table 1); however, according to our DFT calculations, the structures of [B_3_H_9_]^2−^, [B_4_H_12_]^2−^ and [B_5_H_15_]^2−^ are based on [B_2_H_6_]^2−^ geometry (Scheme S3, Table S4). 

The Li[B_n_H_3n−1_] series is formed via the consecutive addition of BH_3_ to LiBH_2_ (Scheme S4). Along this series, HDA^MeCN^ decreases from 64.4 kcal/mol for LiBH_2_ to 44.5–45.1 kcal/mol for Li[B_4_H_11_] and Li[B_5_H_14_]. The most stable structure in this series is Li[B_3_H_8_], and this anion is used as a ligand in transition metal complexes [70,90,91,92,93,94]. In the case of Li[B_4_H_11_] and Li[B_5_H_14_], the isomers, the structures of which are based on [B_3_H_8_]^−^, are found by 19.2 and 24.0 kcal/mol lower in ∆G°_MeCN_ scale than the isomers having a cyclic structure (Scheme S4, Table S5).

**Scheme 6:**
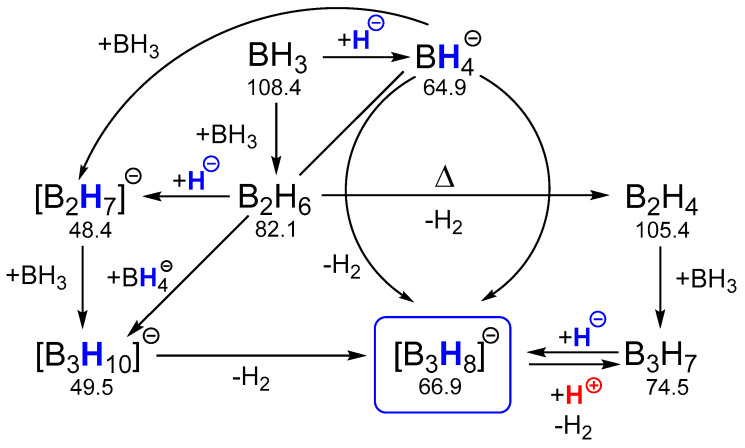
Reactions of tetrahydroborate anion with neutral boranes leading to [B_3_H_8_]^−^. HDA^MeCN^ values (in kcal/mol) are also given.

According to the literature, there are several possibilities for synthesizing [B_3_H_8_]^−^ (Scheme 6) [52,58,95]. Formation of the octahydrotriborate anion [B_3_H_8_]^−^ in the reaction of BH_4_^−^ with B_2_H_6_ or with B_2_H_4_ can be explained by the electrophilic nature of neutral boranes (evidenced by higher HDA^MeCN^, Table 1 and Scheme S1) [58,95].

The B_n_H_3n−2_ series is formed via the consecutive addition of BH_3_ to BH (Scheme S5). In this series, except for the B_5_H_13_, all compounds were obtained experimentally. The first BH_3_ addition leads to an increase in HDA^MeCN^ by 14.5 kcal/mol, the second addition causes its reduction by 30.9 kcal/mol, whereas for the next members in the series, B_3_H_7_, B_4_H_10_, and B_5_H_13_, HDA^MeCN^ varies in a narrow range of 74.5–78.2 kcal/mol.

Boron monohydride radical is known to be generated in the gas phase by photodissociation of BH_3_CO [96,97]. B_2_H_4_ can be obtained by the reaction of B_2_H_6_ with F radicals in the gas phase [98]. Due to its high electrophilicity (HDA^MeCN^ = 105.4 for B_2_H_4_ is comparable to HDA^MeCN^ = 108.4 for BH_3_), there is a lack of transition metal complexes with B_2_H_4_ [99]; however, it can be stabilized in the form of a Lewis acid–base complex (Me_3_P)_2_B_2_H_4_ [100,101,102,103,104]. In turn, (Me_3_P)_2_B_2_H_4_ can act as a bidentate ligand in transition metal complexes [102,103,104,105]. B_3_H_7_ can be easily obtained from B_3_H_8_^−^ by reaction with a non-oxidizing acid (Scheme 6) in ether solvents (e.g., THF) or in the presence of other Lewis bases (L = R_3_N, R_3_P) [52,106,107] with the formation of L∙B_3_H_7_ adduct [108]. The most stable borane in this series, B_4_H_10_, was characterized rather a long time ago, and its structure was determined in the gas phase by gas-phase electron diffraction [109,110]. B_4_H_10_ geometry is preserved in the structure of B_5_H_13_ (Scheme S5, Table S6), which is characterized by a slightly lower HDA^MeCN^ value. 

In the Li_2_[B_n_H_3n−2_] series formed by the consecutive addition of BH_3_ to Li_2_BH (Scheme S6), the HDA^MeCN^ values decrease by 20–52 kcal/mol relative to the corresponding neutral boranes B_3_H_3n−2_ (Table 1 and Scheme 2). Both [B_2_H_4_]^2−^ [99,102,104,111] and [B_3_H_7_]^2−^ [82,112,113,114] are used as ligands in transition metal complexes. The most stable structures, [B_4_H_12_]^2−^ and [B_5_H_15_]^2−^, are based on the geometry of [B_3_H_7_]^2−^, whereas cyclic structures are energetically less favorable by 7.4 and 16.8 kcal/mol (∆G°_MeCN_), respectively (Table S7). 

The Li[B_n_H_3n−3_] series is formed by the addition of BH_3_ to B^−^ (Scheme S7, Table S8). The change in the thermodynamic hydricity in this series is uneven. The first BH_3_ addition leads to a significant drop in HDA^MeCN^ by 41.9 kcal/mol, the second addition causes its reduction by 13.3 kcal/mol, and the third addition leads to an increase of HDA^MeCN^ by 11.3 kcal/mol. The first two members of this series, [B_2_H_3_]^−^ [115,116] and [B_3_H_6_]^−^ [80], are observed in the gas phase during collision-induced dissociation [117], and their structures were predicted by DFT calculations. [B_4_H_9_]^−^ can be synthesized in quantitative yield by deprotonation of B_4_H_10_, whereas the addition of B_2_H_6_ to [B_4_H_9_]^−^ yields [B_5_H_12_]^−^ [51,118]. [B_4_H_9_]^−^ is used as a ligand in transition metal complexes [70,119,120,121,122], and it is the most stable structure in this series (HDA^MeCN^ = 60.2 kcal/mol).

Finally, the B_n_H_3n−4_ series is formed via the consecutive addition of BH_3_ to B_2_H_2_ (Scheme S8, Table S9). Upon the BH_3_ addition, HDA^MeCN^ gradually decreases from an extremely high value of 168.6 kcal/mol for B_2_H_2_ to 85.5 kcal/mol for B_5_H_11_. B_2_H_2_ [100,123], B_3_H_5_ [100] and B_4_H_8_ [124,125] are obtained as a result of higher borane cleavage (e.g., B_5_H_9_) and need to be stabilized by the interaction with Lewis bases (Me_3_P, Me_3_N, Me_2_S) due to their high Lewis acidity. Their Lewis acid–base adducts (such as (Me_3_P)_2_∙B_2_H_2_) have used as ligands in transition metal complexes [104,126,127]. 

### 2.2. Thermodynamic Hydricity of Polyhedral Closo-Boranes

During thermal decomposition of metal tetrahydroborates, the formation of stable metal dodecaborane [B_12_H_12_]^2−^ with an admixture of [B_10_H_10_]^2−^ via intermediate boron clusters such as [B_2_H_7_]^−^ and [B_3_H_8_]^−^ was observed [42,45,46,47,50]. In octahedral [B_6_H_6_]^2−^ and icosahedral [B_12_H_12_]^2−^ *closo*-borane dianions, all terminal BH groups are equivalent. However, in their lithium salts Li_2_[B_n_H_n_], cation–anion interaction causes asymmetry in the bond lengths of terminal BH groups interacting with Li atoms. Therefore, thermodynamic hydricity of polyhedral *closo*-boranes was assessed for both [B_n_H_n_]^2−^ dianions and their lithium salts Li_2_[B_n_H_n_] (n = 5–17).

Theoretical calculations show that the thermodynamic stability of polyhedral *closo*-boranes increases with an increase in the number of boron atoms participating in the cluster formation from [B_5_H_5_]^2−^ to [B_12_H_12_]^2−^ [50]. According to thermodynamic stabilities, *closo*-borane dianions such as [B_17_H_17_]^2−^ and [B_16_H_16_]^2−^ should be even more stable than [B_12_H_12_]^2−^ [128]. Indeed, DFT calculations predict that the formation of large clusters (such as [B_42_H_42_]^2−^, [B_60_H_60_]^2−^, [B_92_H_92_]^2−^) is possible [129,130,131,132]. However, one should keep in mind that the more B–H bonds present in a polyhedral *closo*-borane, the higher its stability, since more energy is stored in these chemical bonds [50]. 

**Figure 2:**
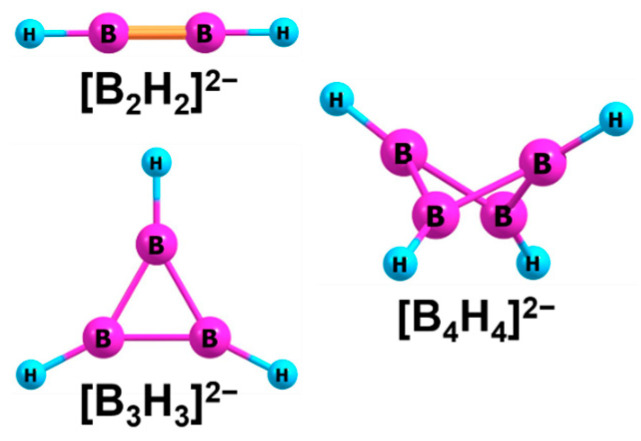
M06-optimized geometries of Li_2_[B_n_H_n_] (n = 2–4). Lithium atoms are omitted for clarity.

To gain insight in thermodynamic hydricity of polyhedral *closo*-boranes, we calculated at first stage HDA^MeCN^ of small dianionic boranes [B_n_H_n_]^2−^ and their lithium salts Li_2_[B_n_H_n_] (n = 2–4) (Figure 2), which can be formally viewed as building blocks or prototypes of polyhedral *closo*-boranes. 

**Scheme 7:**
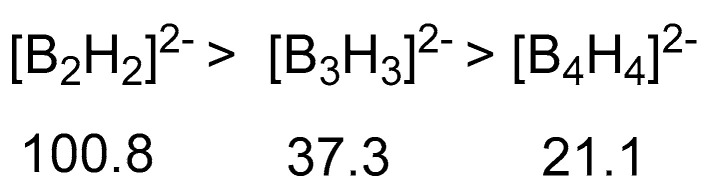
Order of HDA^MeCN^ (in kcal/mol) for borane dianions [B_n_H_n_]^2−^ (n = 2–4).

Whereas [B_2_H_2_]^2−^ is linear, and [B_3_H_3_]^2−^ is planar, [B_4_H_4_]^2−^ has been described as having an “intermediate configuration between planar and tetragonal geometry” [133], which is actually disphenoid. In each structure, all B-H_term_ bonds are equivalent (r_BHterm_ = 1.191 Å for [B_2_H_2_]^2−^; r_BHterm_ = 1.209 Å for [B_3_H_3_]^2−^ and r_BHterm_ = 1.206 Å for [B_4_H_4_]^2−^). The decrease of HDA^MeCN^ values in this series (Scheme 7) is apparently associated with a better delocalization of electron density and with an increase in the number of possible resonance structures as the cluster size grows. In [B_2_H_2_]^2−^ featuring a triple B≡B bond, there are two 2c-2ē π-bonds, whereas in [B_3_H_3_]^2−^ and [B_4_H_4_]^2−^ featuring π-aromatic systems, there is one 3c-2ē and one 4c-2ē delocalized π-bond, respectively [133]. 

**Figure 3:**
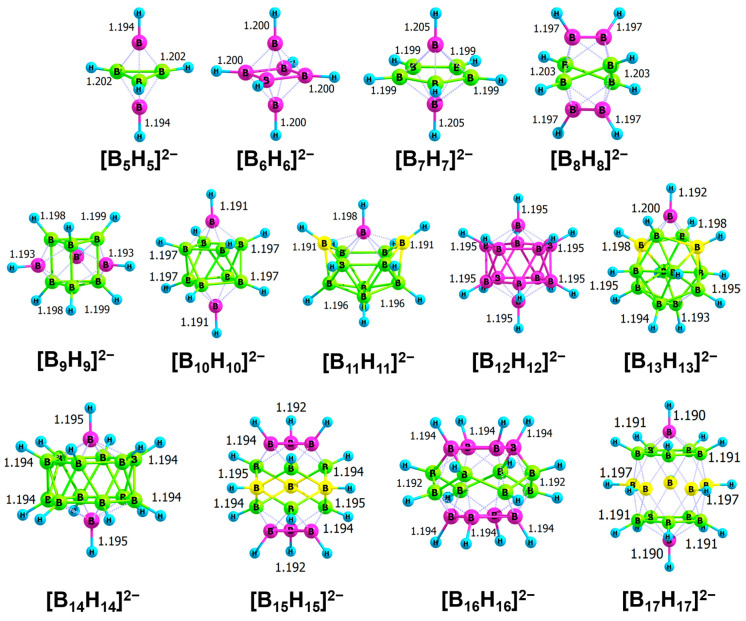
M06-optimized geometries of [B_n_H_n_]^2−^ (n = 5–17) dianions of polyhedral *closo*-boranes. Boron atoms given in violet, green or yellow color represent structural fragments with different coordination numbers of boron atom. Black numbers are B–H bond length in Å. Dotted lines represent the connection between capes and belts of a polyhedron.

**Figure 4:**
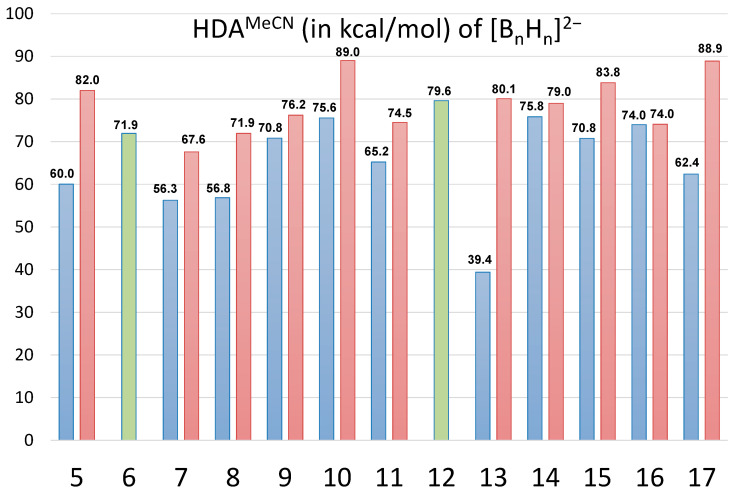
HDA^MeCN^ of polyhedral *closo*-borane dianions [B_n_H_n_]^2−^ (n = 5–17). Blue columns represent the lowest HDA^MeCN^ values, and red columns represent the highest in the given anion; green columns show HDA^MeCN^ for symmetric [B_6_H_6_]^2−^ and [B_12_H_12_]^2−^ dianions.

Further increase of borane cluster size results in the formation of polyhedral *closo*-boranes structures Li_2_[B_n_H_n_] (n = 5–17) (Figure 3), which are formed following the Wade’s rules [134,135]. Due to the 3D aromaticity, *closo*-boranes are characterized by higher HDA^MeCN^ values (Figure 4, Figure S2) than small [B_n_H_n_]^2−^ clusters (n = 2–4). Formal addition of BH to [B_4_H_4_]^2−^ yields [B_5_H_5_]^2−^, which is the least stable member of the polyhedral *closo*-boranes [B_n_H_n_]^2−^ according to formation enthalpy [50], and has never been synthesized [128,133]. The structure of [B_5_H_5_]^2−^ is typical for the polyhedral *closo*-boranes—there are two types of boron atoms—two B atoms form caps, and a group of three other B atoms has the geometry of a B_3_H_3_ triangle, forming a belt of the polyhedron (for further description of structural features of polyhedral *closo*-boranes, see in SI). 

Boron atoms in the belt and caps of the polyhedron have different coordination numbers (CN)—4 and 5, respectively. That in turn leads to a difference in B–H bonds’ lengths (r_BH_^ap^ = 1.194 Å and r_BH_^eq^ = 1.202 Å, Figure 3) and anisotropy in the charge distribution across the polyhedron (Figure S2). According to natural bond population analysis (NPA) of [B_5_H_5_]^2−^ the capping boron atoms are more negatively charged (q^MeCN^ = −0.437) than the equatorial ones (q^MeCN^ = −0.331÷ −0.332). Thus, in the case of [B_5_H_5_]^2−^, the apical BH groups (82.0 kcal/mol) have higher HDA^MeCN^ values than equatorial ones (60.0 kcal/mol) (Figure S2). This HDA^MeCN^(BH^ap^)/HDA^MeCN^(BH^eq^) ratio is conserved when going to larger dianions [B_10_H_10_]^2−^, [B_15_H_15_]^2−^ and [B_17_H_17_]^2−^.

To gain insight into the thermodynamic hydricity of polyhedral *closo*-boranes, it is highly important to consider the difference in geometry along with the charge distribution in their dianions (Figure S2, Table S10). These parameters suggest different reactivity of boron atoms forming the polyhedron’s caps and belt (Figure 3). Although different bond lengths of terminal B-H already indicate different properties of these centers, it is not possible to estimate their thermodynamic hydricity, since there is no general relationship between r_BHterm_ and HDA^MeCN^ (Figure S4).

Thermodynamic hydricity was assessed for both [B_n_H_n_]^2−^ dianions (Figure 4, Table S10) and their lithium salts Li_2_[B_n_H_n_] (n = 5–17) (Figure S3, Table S11). In lithium salts, the cation–anion interaction causes asymmetry in BH bond length and leads to different HDA^MeCN^ values for the same vertex types, which pushes us to use HDA^MeCN^ for dianions to make a correct comparison inside the [B_n_H_n_]^2−^ series. HDA^MeCN^ values for lithium salts (typically higher by 10.2–22.3 kcal/mol than those for dianions) are provided for the comparison with the neutral and monoanionic hydrides (Table S11). HDA^MeCN^ of *closo*-boranes directly depends on the coordination number (Table S10) of the boron atom, for which the hydride abstraction and stabilization of quasi-borinium cation take place. In general, the larger the coordination number (CN) of a boron atom, the lower the value of HDA^MeCN^. Deviation from this rule is observed only for [B_11_H_11_]^2−^, [B_13_H_13_]^2−^ and [B_14_H_14_]^2−^, where the boron atoms with the highest CN have the largest HDA^MeCN^ values. The probable explanation is that despite that the boron atoms forming the polyhedron belt have a lower CN than the boron atoms of the cape, the interaction between them and the surrounding atoms is stronger. This is suggested by shorter r_(B-B)_ for borons with lower CN, as in, e.g., [B_11_H_11_]^2−^ (1.773–1.802 Å), [B_13_H_13_]^2−^ (1.739–1.826 Å) and [B_14_H_14_]^2−^ (1.732–1.904 Å), in comparison to longer bonds at boron atoms with higher CN (r_(B-B)_ is 1.738–1.971 Å for [B_11_H_11_]^2−^, 1.842–1.904 Å for [B_13_H_13_]^2−^ and 1.904 Å for [B_14_H_14_]^2−^).

**Scheme 8:**
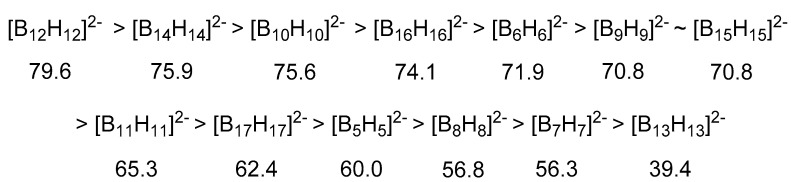
The trend of HDA^MeCN^ for dianions of polyhedral *closo*-borane [B_n_H_n_] (n = 5–17) taking the lowest value in the given anion.

In most cases, except for [B_7_H_7_]^2−^ and [B_16_H_16_]^2−^, the equatorial BH groups have the lowest HDA^MeCN^ values. The trend in the ability to hydride transfer (Scheme 8, for Li_2_[B_n_H_n_] see Scheme S10) correlates with the calculated Gibbs free energy per unit (G°/n) for both Li_2_[B_n_H_n_] and [B_n_H_n_]^2−^ (n = 5–17) (Figures S5 and S6) and with previously reported energy per unit by PRDDO calculations [136]. The data obtained indicate that the most stable *closo*-boranes [B_12_H_12_]^2−^, [B_10_H_10_]^2−^ and [B_6_H_6_]^2−^ are least prone to the reaction of hydride transfer. At the same time, *closo*-boranes which differ from [B_6_H_6_]^2−^ and [B_12_H_12_]^2−^ by one BH group (B_5_/B_7_ and B_11_/B_13_, respectively) and [B_8_H_8_]^2−^ are the most reactive in hydride-transfer reactions. The high reactivity of [B_13_H_13_]^2−^, which HDA^MeCN^ (39.7 kcal/mol) is comparable to that of “superhydride” Li[Et_3_BH] (HDA^MeCN^ = 37.3 kcal/mol) [34], which apparently explains the challenge of jumping over the so-called “icosahedral barrier” in the synthesis of higher *closo*-borane clusters. The higher propensity of some *closo*-boranes for hydride transfer could be used for the directed generation of quasi-borinium cations, which are postulated as intermediates of the electrophile-induced nucleophilic substitution [36,137].

Previously, electron-donating properties of polyhedral boranes were assessed by ^1^H NMR, which revealed the electron-donating ability decrease in the row [2-B_10_H_9_]^2−^ > [B_12_H_12_]^2−^ > [1-B_10_H_9_]^2−^ [37]. According to our calculations, the HDA^MeCN^ value for [B_12_H_12_]^2−^ (79.6 kcal/mol) is higher than HDA^MeCN^ for equatorial BH groups in [B_10_H_10_]^2−^ (75.6 kcal/mol), but lower than for apical BH terminal groups in [B_10_H_10_]^2−^ (89.0 kcal/mol).

## 3. Materials and Methods

### Computational Details

In the present manuscript, DFT/M06 was used to allow a comparison to hydride donating ability of tetracoordinated boron hydrides previously calculated by the same method [34]. Additionally, the values obtained can be used as a reference for assessing the effectiveness of the activation of B-H bonds by transition metals. In this regard, M06 is a more versatile method than M06-2X (generally recommended for calculation thermochemistry of main group elements) and can be used in the cases where multi-reference systems are or might be involved since it has been parametrized for both main group elements and transition metals [138]. 

All calculations were performed without symmetry constraints using the M06 hybrid functional [138] and MP2 implemented in the *Gaussian09* (Revision D.01) (Wallingford, CT, USA) [139] software package, using the 6-311++G(d,p) basis set [140]. 

Vibrational frequencies were calculated for all optimized complexes at the same level of theory to confirm a character of local minima on the potential energy surface. Visualization of the optimized geometries was realized using the *Chemcraft 1.8* graphical visualization program [141].

The inclusion of nonspecific solvent effects in the calculations was performed by using the SMD method [142]. Acetonitrile (MeCN, ε = 35.7) was chosen as a solvent for the geometry optimization because a large amount of data on reduction potentials, pKa values, and experimental hydride donating ability (HDA) of transition metal hydride complexes were determined in MeCN [31,143]. 

The calculations were carried out with an ultrafine integration grid and a very tight SCF option to improve the accuracy of the optimization procedure and thermochemical calculations. 

Hydride donating ability in MeCN (HDA^MeCN^) was calculated as Gibbs free energy of hydride transfer [HDA^MeCN^ ≡ ΔG°[H]^−^_Solv_ = G°_Solv_ (E^+^) + G°_Solv_ (H^−^) − G°_Solv_ (E–H)].

From the data obtained during the geometry optimization, for each molecule, the most stable configuration of each of its conformers was chosen. To find the most stable configuration of cationic boranes, the terminal hydrogen atoms (B–H_term_) were torn off from each vertex in optimized molecules. As is widely known, bridge hydrogen atoms (B–H_br_–B) have an increased acidity [5,58,144,145,146,147]; therefore, their participation in the hydride transfer was not considered herein. For most of the small borane clusters, due to the structural rearrangements during the geometry optimization only one stable configuration of cationic boranes was found. 

In the case of polyhedral *closo*-boranes due to the rigid frame of the boron cluster [133,148], several quasi-borinium cations were observed, localized on vertices from which hydride was torn off. 

## 4. Conclusions

Our DFT study of thermodynamic hydricity (HDA^MeCN^) revealed that for small borane clusters (up to 5 boron atoms), the hydride detachment occurs only from the ending terminal BH_n_ (n = 1–3) group having the largest number of hydrogen atoms. The experimental data and the HDA^MeCN^ pattern obtained suggest that stable borane clusters have HDA^MeCN^ between 48 and 82 kcal/mol. Hydrides with lower HDA^MeCN^ would be hydrolytically unstable, and those with higher HDA^MeCN^ tend to aggregation in larger clusters. Neutral boranes with high Lewis acidity such as B_2_H_2_ (168.6 kcal/mol), B_3_H_5_ (109.9 kcal/mol), BH_3_ (108.4 kcal/mol), B_2_H_4_ (105.4 kcal/mol) and B_4_H_8_ (89.2 kcal/mol) could be stabilized in the form a Lewis acid–base complex (L∙B_x_H_y_, where L = R_3_N, R_3_P, THF, etc.), the formation of which increases the BH group’s hydricity (lowers HDA^MeCN^). These HDA^MeCN^ border lines are exemplified by diborane B_2_H_6_ (HDA^MeCN^ = 82.1 kcal/mol) and its dianion [B_2_H_6_]^2−^ (HDA^MeCN^ = 40.9 kcal/mol for Li_2_[B_2_H_6_]). Borane clusters with HDA^MeCN^ less than 41 kcal/mol are strong hydride donors capable of reducing CO_2_ (HDA^MeCN^ = 44 kcal/mol for HCO_2_^−^), whereas those with HDA^MeCN^ over 82 kcal/mol, predominately neutral boranes, are weak hydride donors and could even serve as proton donors (e.g., B_2_H_6_, B_4_H_10_, B_5_H_11_ etc.). 

In *closo*-boranes, HDA^MeCN^ depends on the coordination number (CN) of the boron atom from which hydride detachment and stabilization of quasi-borinium cation takes place. Thus, HDA^MeCN^ for equatorial boron vertices (75.6 kcal/mol, CN = 6) in [B_10_H_10_]^2−^ is lower than those for apical boron vertices (89.0 kcal/mol, CN = 5). That could explain the observed experimental reactivity row [2-B_10_H_9_]^2−^ > [B_12_H_12_]^2−^ > [1-B_10_H_9_]^2−^ [37]. 

## Figures and Tables

**Table 1:** Calculated hydride donating ability in MeCN (HDA^MeCN^ in kcal/mol) for homologous series of neutral and anionic boranes clusters.

n	Li[B_n_H_3n+1_]	B_n_H_3n_	Li_2_[B_n_H_3n_]	Li[B_n_H_3n−1_]	B_n_H_3n−2_	Li_2_[B_n_H_3n−2_]	Li[B_n_H_3n−3_]	B_n_H_3n−4_
**1**	LiBH_4_	BH_3_	Li_2_BH_3_	LiBH_2_	BH	Li_2_BH	-	-
	64.9	108.4	43.8	64.4	90.9	42.7		
**2**	Li[B_2_H_7_]	B_2_H_6_	Li_2_[B_2_H_6_]	Li[B_2_H_5_]	B_2_H_4_	Li_2_[B_2_H_4_]	Li[B_2_H_3_]	B_2_H_2_
	48.4	82.1	40.9	63.9	105.4	55.6	88.9	168.6
**3**	Li[B_3_H_10_]	B_3_H_9_	Li_2_[B_3_H_9_]	Li[B_3_H_8_]	B_3_H_7_	Li_2_[B_3_H_7_]	Li[B_3_H_6_]	B_3_H_5_
	49.2	69.3	28.2	66.9	74.5	54.6	47.0	109.8
**4**	Li[B_4_H_13_]	B_4_H_12_	Li_2_[B_4_H_12_]	Li[B_4_H_11_]	B_4_H_10_	Li_2_[B_4_H_10_]	Li[B_4_H_9_]	B_4_H_8_
	55.2	60.1	35.8	44.5	78.2	25.8	60.2	89.2
**5**	Li[B_5_H_16_]	B_5_H_15_	Li_2_[B_5_H_15_]	Li[B_5_H_14_]	B_5_H_13_	Li_2_[B_5_H_13_]	Li[B_5_H_12_]	B_5_H_11_
	57.0	55.1	38.6	45.1	76.4	38.4	54.9	85.5

The HDA^MeCN^ values are given for the most energetically favorable isomer. The most stable boranes in the series according to calculated heats of formation [50,56] are shown by the violet shading of the cells.

## Supplementary Materials

The following are available online. Scheme S1. Scheme of homologous series of neutral and monoanionic boranes clusters. Scheme S2. Homologous series of neutral and dianionic boranes clusters, Figure S1. Plots of HDA^MeCN^ against the number of boron atoms in borane clusters derived from different starting species. Table S1. Computed [M06/6-311++G(d,p)] B-H terminal bond length (in Å), hydride donating ability (HDA^MeCN^ alias ΔG°_[H]−MeCN_ in kcal/mol) and enthalpy of hydride detaching reaction (ΔG°_[H]−MeCN_ in kcal/mol) for Li[B_n_H_3n+1_] series. Table S2. Computed [M06/6-311++G(d,p)] B-H terminal bond length (in Å), hydride donating ability (HDA^MeCN^ alias ΔG°_[H]−MeCN_ in kcal/mol) and enthalpy of hydride detaching reaction (ΔG°_[H]−MeCN_ in kcal/mol) for B_n_H_3n_ series. Table S3. Difference in Gibbs energy (∆G°_MeCN_ in kcal/mol) relative to the most stable isomer of B_3_H_9_ in MeCN (∆G°_MeCN_ in kcal/mol), B-H terminal bond length (in Å), hydride donating ability (HDA^MeCN^ alias ΔG°_[H]−MeCN_ in kcal/mol) and enthalpy of hydride detaching reaction (ΔG°_[H]−MeCN_ in kcal/mol). Scheme S3. Li_2_[B_n_H_3n_] series. Table S4. Computed [M06/6-311++G(d,p)] B-H terminal bond length (in Å), hydride donating ability (HDA^MeCN^ alias ΔG°_[H]−MeCN_ in kcal/mol) and enthalpy of hydride detaching reaction (ΔG°_[H]−MeCN_ in kcal/mol) for Li_2_[B_n_H_3n_] series; Scheme S4. Li[B_n_H_3n−1_] series. Table S5. Computed [M06/6-311++G(d,p)] B-H terminal bond length (in Å), hydride donating ability (HDA^MeCN^ alias ΔG°_[H]−MeCN_ in kcal/mol) and enthalpy of hydride detaching reaction (ΔG°_[H]−MeCN_ in kcal/mol) for Li[B_n_H_3n−1_] series. Scheme S5. B_n_H_3n−2_ series. Table S6. Computed [M06/6-311++G(d,p)] B-H terminal bond length (in Å), hydride donating ability (HDA^MeCN^ alias ΔG°_[H]−MeCN_ in kcal/mol) and enthalpy of hydride detaching reaction (ΔG°_[H]−MeCN_ in kcal/mol) for B_n_H_3n−2_ series. Scheme S6. Li_2_[B_n_H_3n−_] series. Table S7. Computed [M06/6-311++G(d,p)] B-H terminal bond length (in Å), hydride donating ability (HDA^MeCN^ alias ΔG°_[H]−MeCN_ in kcal/mol) and enthalpy of hydride detaching reaction (ΔG°_[H]−MeCN_ in kcal/mol) for Li_2_[B_n_H_3n−2_] series. Scheme S7. Li[B_n_H_3n−3_] series. Table S8. Computed [M06/6-311++G(d,p)] B-H terminal bond length (in Å), hydride donating ability (HDA^MeCN^ alias ΔG°_[H]−MeCN_ in kcal/mol) and enthalpy of hydride detaching reaction (ΔG°_[H]−MeCN_ in kcal/mol) for Li[B_n_H_3n−3_] series. Scheme S8. B_n_H_3n−4_ series. Table S9. Computed [M06/6-311++G(d,p)] B-H terminal bond length (in Å), hydride donating ability (HDA^MeCN^ alias ΔG°_[H]−MeCN_ in kcal/mol) and enthalpy of hydride detaching reaction (ΔG°_[H]−MeCN_ in kcal/mol) for B_n_H_3n−4_ series. Structural features of polyhedral *closo*−boranes. Figure S2. NPA charge distribution (showed in blue-green-red scale from −0.40 to 0.05), calculated for M06-optimized geometries of dianions [B_n_H_n_]^2−^ (n = 5–17) of polyhedral *closo*-boranes in MeCN. Table S10. Coordination numbers (CN) of boron atom in polyhedral *closo*-boranes. Computed [M06/6-311++G(d,p)] B-H terminal bond length (in Å), hydride donating ability (HDA^MeCN^ alias ΔG°_[H]−MeCN_ in kcal/mol) and enthalpy of hydride detaching reaction (ΔH°_[H]−MeCN_ in kcal/mol). Table S11. Computed [M06/6-311++G(d,p)] B-H terminal bond length (in Å), hydride donating ability (HDA^MeCN^ alias ΔG°_[H]−MeCN_ in kcal/mol) and enthalpy of hydride detaching reaction (ΔG°_[H]−MeCN_ in kcal/mol). Figure S3. HDA^MeCN^ of polyhedral *closo*-boranes Li_2_[B_n_H_n_] (n = 5–17). Scheme S9. General trend of HDA^MeCN^ for Li_2_[B_n_H_n_] (n = 2–4). Scheme S10. General trend of HDA^MeCN^ for polyhedral *closo*-boranes Li_2_[B_n_H_n_] (n = 5–17). Figure S4. Plot HDA^MeCN^ vs bond length of terminal B-H bond for Li salts polyhedral *closo*-boranes. Figure S5. Free Gibbs energy per BH unit calculated for dianions [B_n_H_n_]^2−^ (n = 5–17) of polyhedral *closo*-boranes and their Li-salts Li_2_[B_n_H_n_] in (n = 5–17) MeCN vs number boron atoms. Figure S6. Graph of normalized lowest HDA^MeCN^ of and free Gibbs energy per BH unit for dianions [B_n_H_n_]^2−^ (n = 5–17) of polyhedral *closo*-boranes vs number boron atoms. Table S12. DFT-optimized geometries (Cartesian coordinates) and electronic energies.

## References

[1] Greenwood N.N. (1994). Electron-Deficient Boranes as Novel Electron-Donor Ligands. ACS Symposium Series.

[2] Greenwood N.N. (2002). The concept of boranes as ligands. Co-ord. Chem. Rev..

[3] Alexandrova A.N., Boldyrev A.I., Zhai H.-J., Wang L.-S. (2006). All-boron aromatic clusters as potential new inorganic ligands and building blocks in chemistry. Co-ord. Chem. Rev..

[4] Besora M., Lledós A. (2008). Coordination Modes and Hydride Exchange Dynamics in Transition Metal Tetrahydroborate Complexe. Contemporary Metal Boron Chemistry I: Borylenes, Boryls, Borane Sigma-complexes, and Borohydrides.

[5] Hansen B.R.S., Paskevicius M., Li H.-W., Akiba E., Jensen T.R. (2016). Metal boranes: Progress and applications. Coord. Chem. Rev..

[6] Mohtadi R., Remhof A., Jena P. (2016). Complex metal borohydrides: Multifunctional materials for energy storage and conversion. J. Phys. Condens. Matter.

[7] Roll M.F. (2016). Ionic borohydride clusters for the next generation of boron thin-films: Nano-building blocks for electrochemical and refractory materials. J. Mater. Res..

[8] Huang Z., Wang S., Dewhurst R.D., Ignat’Ev N.V., Finze M., Braunschweig H. (2020). Boron: Its Role in Energy-Related Processes and Applications. Angew. Chem. Int. Ed..

[9] Lu Z., Ciucci F. (2017). Metal Borohydrides as Electrolytes for Solid-State Li, Na, Mg, and Ca Batteries: A First-Principles Study. Chem. Mater..

[10] Guzik M.N., Mohtadi R., Sartori S. (2019). Lightweight complex metal hydrides for Li-, Na-, and Mg-based batteries. J. Mater. Res..

[11] Huang S., Qi X., Zhang W., Liu T., Zhang Q. (2015). Exploring Sustainable Rocket Fuels: [Imidazolyl−Amine−BH_2_]^+^-Cation-Based Ionic Liquids as Replacements for Toxic Hydrazine Derivatives. Chem. Asian J..

[12] Huang S., Qi X., Liu T., Wang K., Zhang W., Li J., Zhang Q. (2016). Towards Safer Rocket Fuels: Hypergolic Imidazolylidene-Borane Compounds as Replacements for Hydrazine Derivatives. Chem. Eur. J..

[13] Liu T., Qi X., Huang S., Jiang L., Li J., Tang C., Zhang Q. (2016). Exploiting hydrophobic borohydride-rich ionic liquids as faster-igniting rocket fuels. Chem. Commun..

[14] Ley M.B., Jepsen L.H., Lee Y.-S., Cho Y.W., Von Colbe J.B., Dornheim M., Rokni M.M., Jensen J.O., Sloth M., Filinchuk Y. (2014). Complex hydrides for hydrogen storage—new perspectives. Mater. Today.

[15] Mohtadi R., Orimo S.-I. (2016). The renaissance of hydrides as energy materials. Nat. Rev. Mater..

[16] Callini E., Atakli Z., Özlem K., Hauback B.C., Orimo S.-I., Jensen C., Dornheim M., Grant D.M., Cho Y.W., Chen P. (2016). Complex and liquid hydrides for energy storage. Appl. Phys. A.

[17] Fisher S.P., Tomich A.W., Lovera S.O., Kleinsasser J.F., Guo J., Asay M.J., Nelson H.M., Lavallo V. (2019). Nonclassical Applications of closo-Carborane Anions: From Main Group Chemistry and Catalysis to Energy Storage. Chem. Rev..

[18] Hawthorne M.F. (1998). New horizons for therapy based on the boron neutron capture reaction. Mol. Med. Today.

[19] Barth R.F., Coderre J.A., Vicente M.G.H., Blue T.E. (2005). Boron Neutron Capture Therapy of Cancer: Current Status and Future Prospects. Clin. Cancer Res..

[20] Issa F., Kassiou M., Rendina L. (2011). Boron in Drug Discovery: Carboranes as Unique Pharmacophores in Biologically Active Compounds. Chem. Rev..

[21] Scholz M.S., Hey-Hawkins E. (2011). Carbaboranes as Pharmacophores: Properties, Synthesis, and Application Strategies. Chem. Rev..

[22] Detlef G. (2015). Boron clusters in medicinal chemistry: Perspectives and problems. Pure Appl. Chem..

[23] Leśnikowski Z. (2016). Challenges and Opportunities for the Application of Boron Clusters in Drug Design. J. Med. Chem..

[24] Li X., Yan H., Zhao Q. (2016). Carboranes as a Tool to Tune Phosphorescence. Chem. Eur. J..

[25] Mukherjee S., Thilagar P. (2016). Boron clusters in luminescent materials. Chem. Commun..

[26] Nunez R., Tarrés M., Ferrer-Ugalde A., De Biani F.F., Teixidor F. (2016). Electrochemistry and Photoluminescence of Icosahedral Carboranes, Boranes, Metallacarboranes, and Their Derivatives. Chem. Rev..

[27] Kaszyński P., Douglass A.G. (1999). Organic derivatives of closo-boranes: A new class of liquid crystal materials. J. Organomet. Chem..

[28] Ringstrand B., Kaszynski P. (2013). Functionalization of the [closo-1-CB_9_H_10_]− Anion for the Construction of New Classes of Liquid Crystals. Acc. Chem. Res..

[29] Han Y.-F., Jin G.-X. (2014). Half-Sandwich Iridium- and Rhodium-based Organometallic Architectures: Rational Design, Synthesis, Characterization, and Applications. Acc. Chem. Res..

[30] Housecroft C.E. (2015). Carboranes as guests, counterions and linkers in coordination polymers and networks. J. Organomet. Chem..

[31] Wiedner E.S., Chambers M.B., Pitman C.L., Bullock R.M., Miller A.J.M., Appel A.M. (2016). Thermodynamic Hydricity of Transition Metal Hydrides. Chem. Rev..

[32] Heiden Z.M., Lathem A.P. (2015). Establishing the Hydride Donor Abilities of Main Group Hydrides. Organometallics.

[33] Alherz A., Lim C.-H., Hynes J.T., Musgrave C.B. (2018). Predicting Hydride Donor Strength via Quantum Chemical Calculations of Hydride Transfer Activation Free Energy. J. Phys. Chem. B.

[34] Golub I.E., Filippov O.A., Belkova N.V., Epstein L.M., Shubina E.S. (2018). Hydride donating abilities of the tetracoordinated boron hydrides. J. Organomet. Chem..

[35] Lathem A.P., Treich N.R., Heiden Z.M. (2015). Establishing the Steric Bulk of Main Group Hydrides in Reduction Reactions. Isr. J. Chem..

[36] Sivaev I.B., Bregadze V.I. (2014). Lewis acidity of boron compounds. Coord. Chem. Rev..

[37] Sivaev I.B., Prikaznov A.V., Anufriev S.A. (2013). On relative electronic effects of polyhedral boron hydrides. J. Organomet. Chem..

[38] Greenwood N.N., Greatrex R. (1987). Kinetics and mechanism of the thermolysis and photolysis of binary boranes. Pure Appl. Chem..

[39] Hwang S.-J., Bowman R., Reiter J.W., Soloveichik G.L., Zhao J.-C., Kabbour H., Ahn C.C. (2008). Rijssenbeek NMR Confirmation for Formation of [B_12_H_12_]^2−^ Complexes during Hydrogen Desorption from Metal Borohydrides. J. Phys. Chem. C.

[40] Ozolins V., Majzoub E.H., Wolverton C. (2009). First-Principles Prediction of Thermodynamically Reversible Hydrogen Storage Reactions in the Li-Mg-Ca-B-H System. J. Am. Chem. Soc..

[41] Zhang Y., Majzoub E., Ozolins V., Wolverton C. (2010). Theoretical prediction of different decomposition paths for Ca(BH_4_)_2_ and Mg(BH_4_)_2_. Phys. Rev. B.

[42] Friedrichs O., Remhof A., Hwang S.-J., Züttel A. (2010). Role of Li_2_B_12_H for the Formation and Decomposition of LiBH_4_. Chem. Mater..

[43] Zhang Y., Majzoub E., Ozolins V., Wolverton C. (2012). Theoretical Prediction of Metastable Intermediates in the Decomposition of Mg(BH_4_)_2_. J. Phys. Chem. C.

[44] Pitt M., Paskevicius M., Brown D.H., Sheppard D.A., Buckley C.E. (2013). Thermal Stability of Li_2_B_12_H_12_ and its Role in the Decomposition of LiBH_4_. J. Am. Chem. Soc..

[45] Yan Y., Remhof A., Rentsch D., Lee Y.-S., Whan Cho Y., Zuttel A. (2013). Is Y_2_(B_12_H_12_)_3_ the main intermediate in the decomposition process of Y(BH_4_)_3_?. Chem. Commun..

[46] Yan Y., Remhof A., Rentsch D., Züttel A. (2015). The role of MgB_12_H_12_ in the hydrogen desorption process of Mg(BH_4_)_2_. Chem. Commun..

[47] Schouwink P., Sadikin Y., van Beek W., Černý R. (2015). Experimental observation of polymerization from BH_4_^−^ to B_12_H_12_^2−^ in mixed-anion A_3_BH_4_B_12_H_12_ (A = Rb^+^, Cs^+^). Int. J. Hydrogen Energy.

[48] Huang Z.-Q., Chen W.-C., Chuang F.-C., Majzoub E.H., Ozolins V. (2016). First-principles calculated decomposition pathways for LiBH4 nanoclusters. Sci. Rep..

[49] McKee M.L., Hnyk D., McKee M. (2015). Deconvoluting the Reaction Path from B_10_H_14_ Plus BH_4_^−^ to B_12_H_12_^2−^. Can Theory Make a Contribution?. Boron: The Fifth Element.

[50] Sethio D., Daku L.M.L., Hagemann H., Kraka E. (2019). Quantitative Assessment of B−B−B, B−Hb−B, and B−Ht Bonds: From BH_3_ to B_12_H_12_^2−^. ChemPhysChem.

[51] Remmel R.J., Johnson H.D., Jaworiwsky I.S., Shore S.G. (1975). Preparation and nuclear magnetic resonance studies of the stereochemically nonrigid anions nonahydrotetraborate(1-)dodecahydropentaborate(1-), undecahydrohexaborate(1-), and dodecahydroheptaborate(1-). Improved syntheses of pentaborane(11) and hexaborane(12). J. Am. Chem. Soc..

[52] Liu X.-R., Chen X.-M., Zhang J., Jensen T.R., Chen X. (2019). The interconversion between THF·B_3_H_7_ and B_3_H_8_−: An efficient synthetic method for MB_3_H_8_ (M = Li and Na). Dalton Trans..

[53] Fernández E., Whiting A. (2015). Synthesis and Application of Organoboron Compounds.

[54] Leach J.B., Toft M.A., Himpsl F.L., Shore S.G. (1981). New, systematic, good yield syntheses of boron hydrides: Preparation of tetraborane(10) and pentaborane(11). A practical conversion of pentaborane(9) to decaborane(14). J. Am. Chem. Soc..

[55] Toft M.A., Leach J.B., Himpsl F.L., Shore S.G. (1982). New systematic syntheses of boron hydrides via hydride ion abstraction reactions: Preparation of B_2_H_6_, B_4_H_10_, B_5_H_11_, and B_10_H_14_. Inorg. Chem..

[56] Nguyen M.T., Matus M.H., Dixon D.A. (2007). Heats of Formation of Boron Hydride Anions and Dianions and Their Ammonium Salts [B_n_H_m_^y−^][NH_4_^+^]_y_ with y = 1−2. Inorg. Chem..

[57] Ilic S., Alherz A., Musgrave C.B., Glusac K.D. (2018). Thermodynamic and kinetic hydricities of metal-free hydrides. Chem. Soc. Rev..

[58] Chen X.-M., Ma N., Zhang Q.-F., Wang J., Feng X., Wei C., Wang L.-S., Zhang J., Chen X. (2018). Elucidation of the Formation Mechanisms of the Octahydrotriborate Anion (B_3_H_8_-) through the Nucleophilicity of the B-H Bond. J. Am. Chem. Soc..

[59] Shore S.G. (1977). Studies of the smaller boron hydrides and their derivatives. Pure Appl. Chem..

[60] Marks T.J., Kolb J.R. (1977). Covalent transition metal, lanthanide, and actinide tetrahydroborate complexes. Chem. Rev..

[61] Makhaev V.D. (2000). Structural and dynamic properties of tetrahydroborate complexes. Russ. Chem. Rev..

[62] Visseaux M., Bonnet F. (2011). Borohydride complexes of rare earths, and their applications in various organic transformations. Co-ord. Chem. Rev..

[63] Golub I.E., Filippov O.A., Gutsul E.I., Belkova N.V., Epstein L.M., Rossin A., Peruzzini M., Shubina E.S. (2012). Dimerization Mechanism of Bis(triphenylphosphine)copper(I) Tetrahydroborate: Proton Transfer via a Dihydrogen Bond. Inorg. Chem..

[64] Belkova N.V., Bakhmutova-Albert E.V., Gutsul E.I., Bakhmutov V.I., Golub I.E., Filippov O.A., Epstein L.M., Peruzzini M., Rossin A., Zanobini F. (2013). Dihydrogen Bonding in Complex (PP_3_)RuH(η^1^-BH_4_) Featuring Two Proton-Accepting Hydride Sites: Experimental and Theoretical Studies. Inorg. Chem..

[65] Golub I.E., Filippov O.A., Belkova N.V., Epstein L.M., Rossin A., Peruzzini M., Shubina E.S. (2016). Two pathways of proton transfer reaction to (triphos)Cu(η^1^-BH_4_) via a dihydrogen bond [triphos = 1,1,1-tris(diphenylphosphinomethyl)ethane]. Dalton Trans..

[66] Belkova N.V., Golub I.E., Gutsul E.I., Lyssenko K.A., Peregudov A.S., Makhaev V.D., Filippov O.A., Epstein L.M., Rossin A., Peruzzini M. (2017). Binuclear Copper(I) Borohydride Complex Containing Bridging Bis(diphenylphosphino) Methane Ligands: Polymorphic Structures of [(µ_2_-dppm)_2_Cu_2_(η^2^-BH_4_)_2_] Dichloromethane Solvate. Crystals.

[67] Golub I.E., Filippov O.A., Belkova N.V., Gutsul E.I., Epstein L.M., Rossin A., Peruzzini M., Shubina E.S. (2017). Competition between the Hydride Ligands of Two Types in Proton Transfer to [{κ^3^-P-CH_3_C(CH_2_CH_2_PPh_2_)_3_}RuH(η^2^-BH_4_)]. Eur. J. Inorg. Chem..

[68] Safronov S.V., Gutsul E.I., Golub I.E., Dolgushin F.M., Nelubina Y.V., Filippov O.A., Epstein L.M., Peregudov A.S., Belkova N.V., Shubina E.S. (2019). Synthesis, structural properties and reactivity of ruthenocene-based pincer Pd(II) tetrahydroborate. Dalton Trans..

[69] Grebenik P.D., Green M.L.H., Kelland M.A., Leach J.B., Mountford P., Stringer G., Walker N.M., Wong L.-L. (1988). Formation of three-vertex metallaboranes from monoborane precursors: X-ray crystal structures of the molybdenum and ruthenium complexes [Mo(η-C_5_H_5_)_2_H(η^2^-B_2_H_5_)] and [Ru(η-C_5_Me_5_)(PMe_3_)(η^2^-B_2_H_7_)]. J. Chem. Soc. Chem. Commun..

[70] Green M.L.H., Leach J.B., Kelland M.A. (2007). Synthesis and Interconversion of Some Small Ruthenaboranes: Reaction of a Ruthenium Borohydride with Pentaborane(9) to Form Larger Ruthenaboranes. Organometallics.

[71] Srivastava A.K., Misra N. (2016). Designing new electrolytic salts for Lithium Ion Batteries using superhalogen anions. Polyhedron.

[72] Alcaraz G., Sabo-Etienne S. (2010). Coordination and Dehydrogenation of Amine-Boranes at Metal Centers. Angew. Chem. Int. Ed..

[73] Titov A.A., Guseva E.A., Smol’Yakov A.F., Dolgushin F.M., Filippov O.A., Golub I.E., Krylova A.I., Babakhina G.M., Epstein L.M., Shubina E.S. (2013). Complexation of trimeric copper(i) and silver(i) 3,5-bis(trifluoromethyl)pyrazolates with amine-borane. Russ. Chem. Bull..

[74] Johnson H.C., Hooper T.N., Weller A.S. (2015). The Catalytic Dehydrocoupling of Amine–Boranes and Phosphine–Boranes. Topics in Organometallic Chemistry.

[75] Rossin A., Peruzzini M. (2016). Ammonia–Borane and Amine–Borane Dehydrogenation Mediated by Complex Metal Hydrides. Chem. Rev..

[76] Todisco S., Luconi L., Giambastiani G., Rossin A., Peruzzini M., Golub I.E., Filippov O.A., Belkova N.V., Shubina E.S. (2017). Ammonia Borane Dehydrogenation Catalyzed by (κ^4^-EP_3_)Co(H) [EP_3_ = E(CH_2_CH_2_PPh_2_)_3_; E = N, P] and H_2_ Evolution from Their Interaction with NH Acids. Inorg. Chem..

[77] Colebatch A.L., Weller A.S. (2018). Amine–Borane Dehydropolymerization: Challenges and Opportunities. Chem. Eur. J..

[78] McKee M.L., Lipscomb W.N. (1985). Symmetry lowering in boranes B_3_H_9_ and B_4_H_12_. Inorg. Chem..

[79] Duke B., Gauld J.W., Schaefer H.F. (1995). Ab Initio Characterization of a Triborane(9) Isomer with a Pentacoordinated Central Boron Atom. J. Am. Chem. Soc..

[80] Korkin A.A., Schleyer P.V.R., McKee M.L. (1995). Theoretical ab Initio Study of Neutral and Charged B_3_H_n_ (n = 3–9) Species. Importance of Aromaticity in Determining the Structural Preferences. Inorg. Chem..

[81] Parshall G.W. (1964). Borane Complexes of Transition Metals. J. Am. Chem. Soc..

[82] Aldridge S., Shang M., Fehlner T.P. (1998). Synthesis of Novel Molybdaboranes from (η^5^-C_5_R_5_)MoCl_n_ Precursors (R = H, Me; n = 1, 2, 4). J. Am. Chem. Soc..

[83] Weller A.S., Shang M., Fehlner T.P. (1999). Synthesis of Mono- and Ditungstaboranes from Reaction of Cp*WCl_4_and [Cp*WCl_2_]_2_ with BH_3_·thf or LiBH_4_(Cp* = η^5^-C_5_Me_5_). Control of Reaction Pathway by Choice of Monoboron Reagent and Oxidation State of Metal Center. Organometallics.

[84] Geetharani K., Tussupbayev S., Borowka J., Holthausen M.C., Ghosh S. (2012). A Mechanistic Study of the Utilization of arachno-Diruthenaborane [(Cp*RuCO)_2_B_2_H_6_] as an Active Alkyne-Cyclotrimerization Catalyst. Chem. Eur. J..

[85] Sharmila D., Mondal B., Ramalakshmi R., Kundu S., Varghese B., Ghosh S. (2015). First-Row Transition-Metal-Diborane and -Borylene Complexes. Chem. Eur. J..

[86] Prakash R., Bakthavachalam K., Varghese B., Ghosh S. (2017). Chlorination of the terminal hydrogen atoms in the hydrogen-rich group 5 dimetallaboranes (Cp*M)_2_(B_2_H_6_)_2_ (M = Nb, Ta). J. Organomet. Chem..

[87] Prakash R., Pradhan A.N., Jash M., Kahlal S., Cordier M., Roisnel T., Halet J.-F., Ghosh S. (2020). Diborane(6) and Its Analogues Stabilized by Mono-, Bi-, and Trinuclear Group 7 Templates: Combined Experimental and Theoretical Studies. Inorg. Chem..

[88] Haridas A., Kar S., Raghavendra B., Roisnel T., Dorcet V., Ghosh S. (2019). B–H Functionalization of Hydrogen-Rich [(Cp*V)_2_(B_2_H_6_)_2_]: Synthesis and Structures of [(Cp*V)_2_(B_2_X_2_)_2_H_8_] (X = Cl, SePh; Cp* = η^5^-C_5_Me_5_). Organometallics.

[89] Godfroid R.A., Hill T.G., Onak T.P., Shore S.G. (1994). Formation of [BH_3_]^2−^ and [B_2_H_6_]^2−^ From the Homogeneous Reduction of B_2_H_6_. J. Am. Chem. Soc..

[90] Lippard S.J., Melmed K.M. (1969). Transition metal borohydride complexes. III. Structure of octahydrotriboratobis(triphenylphosphine)copper(I). Inorg. Chem..

[91] Ghosh S., Beatty A.M., Fehlner T.P. (2002). The Reaction of Cp*ReH_6_, Cp* = C_5_Me_5_, with Monoborane to Yield a Novel Rhenaborane. Synthesis and Characterization of arachno-Cp*ReH_3_B_3_H_8_. Collect. Czechoslov. Chem. Commun..

[92] Kim D.Y., You Y., Girolami G.S. (2008). Synthesis and crystal structures of two (cyclopentadienyl)titanium(III) hydroborate complexes, [Cp∗TiCl(BH_4_)]_2_ and Cp_2_Ti(B_3_H_8_). J. Organomet. Chem..

[93] Ramalakshmi R., Bhattacharyya M., Rao C.E., Ghosh S. (2015). Synthesis, structure and chemistry of low-boron containing molybdaborane: Arachno -[Cp*Mo(CO)_2_B_3_H_8_ ]. J. Organomet. Chem..

[94] Joseph B., Saha K., Prakash R., Nandi C., Roisnel T., Ghosh S. (2018). Chalcogenolato-bridged dinuclear half sandwich complexes of ruthenium and iridium. Inorganica Chim. Acta.

[95] Titov L.V., Levicheva M.D., Psikha S.B. (1984). Synthesis and Thermal Decomposition of Magnesium, Calcium, and Strontium Octahydrotriborates Solvated with Diglyme. Russ. J. Inorg. Chem..

[96] Rice J.K., Caldwell N.J., Nelson H.H. (1989). Gas-phase reaction kinetics of boron monohydride. J. Phys. Chem..

[97] Garland N.L., Stanton C.T., Fleming J.W., Baronavski A.P., Nelson H.H. (1990). Boron monohydride reaction kinetics studied with a high-temperature reactor. J. Phys. Chem..

[98] Ruscic B., Schwarz M., Berkowitz J. (1989). Molecular structure and thermal stability of B_2_H_4_ and B_2_H^+^_4_ species. J. Chem. Phys..

[99] Reddy K.H.K., Jemmis E.D. (2013). Stabilization of diborane(4) by transition metal fragments and a novel metal to π Dewar–Chatt–Duncanson model of back donation. Dalton Trans..

[100] Kameda M., Kodama G. (1980). Cleavage reaction of pentaborane(9). Formation of a new hypho triborane adduct. Inorg. Chem..

[101] Snow S.A., Shimoi M., Ostler C.D., Thompson B.K., Kodama G., Parry R.W. (1984). Metal complexes of the neutral borane adduct (B_2_H_4_∙2P(CH_3_)_3_). Inorg. Chem..

[102] Katoh K., Shimoi M., Ogino H. (1992). Syntheses and structures of [M(CO)_5_{B_2_H_4_∙2P(CH_3_)_3_}] and [M(CO)_4_{B_2_H_4_∙2P(CH_3_)_3_}] (M = chromium, molybdenum, tungsten). Inorg. Chem..

[103] Parry R., Kodama G. (1993). Coordination compounds formed using three-center hydrogen bridge bonds: An extension of the Lewis donor—acceptor coordinate bond. Co-ord. Chem. Rev..

[104] Hata M., Kawano Y., Shimoi M. (1998). Synthesis and Structure of a Dichromatetraborane Derivative [{(OC)_4_Cr}_2_(η^4^-H,H‘,H‘‘,H‘‘‘-BH_2_BH_2_·PMe_2_CH_2_PMe_2_)]. Inorg. Chem..

[105] Shimoi M., Katoh K., Tobita H., Ogino H. (1990). Syntheses and properties of bis{bis(trimethylphosphine)tetrahydrodiboron}copper(1+) halide (halide = chloride, iodide) and x-ray crystal structure of the iodide. Inorg. Chem..

[106] Ring M.A., Witucki E.F., Greenough R.C. (1967). Tautomerism exchange in B_3_H_7_.N(CH_3_)_3_ and B_3_H_7_.-THF. Inorg. Chem..

[107] Dewkett W., Beall H., Bushweller C. (1971). Quadrupolar relaxation and intramolecular exchange in (C_6_H_5_CH_2_)_2_NCH_3_(B_3_H_7_). Inorg. Nucl. Chem. Lett..

[108] Driess M., Nöth H. (2008). Molecular Clusters of the Main Group Elements.

[109] Nordman C.E., Lipscomb W.N. (1953). The molecular structure of B_4_H_10_. J. Am. Chem. Soc..

[110] Brain P.T., Morrison C.A., Parsons S., Rankin D.W.H. (1996). Tetraborane(10), B_4_H_10_: Structures in gaseous and crystalline phases. J. Chem. Soc. Dalton Trans..

[111] Kaufmann E., Schleyer P.v.R. (1988). Dilithiodiborane(6) (Li_2_B_2_H_4_). An experimentally viable species with a B=B double bond. Planar no-bond-double-bond isomers with pentacoordinate boron?. Inorg. Chem..

[112] Muetterties E.L., Kane A.R. (1971). Metalloboranes. VI. A B_3_H_7_^2−^ derivative of platinum. A possible π-allyl analog based on boron. J. Am. Chem. Soc..

[113] Guggenberger L.J., Kane A.R., Muetterties E.L. (1972). Metalloboranes. VII. Synthesis and chemistry of π-borallyl complexes and the crystal structure of [(CH_3_)_2_PC_6_H_5_]_2_PtB_3_H_7_. J. Am. Chem. Soc..

[114] DiPasquale A., Lei X., Fehlner T.P. (2001). Metallaborane Reactivity. Complexities of Cobalt Carbonyl Fragment Addition to 1,2-{Cp*RuH}_2_B_3_H_7_, Cp* = η^5^-C_5_Me_5_, and Characterization of a Diruthenium Analogue of Pentaborane(11) 1,2-{Cp*Ru}_2_(CO)_2_B_3_H_7_. Organometallics.

[115] Qu Z.-W., Li Z.-S., Ding Y.-H., Sun C.-C. (2000). Theoretical Study of the Gas-Phase Reaction of Diborane(3) Anion B2H3-with CO2. J. Phys. Chem. A.

[116] Guermoune A., Jarid A., Ouassas A., Chafiq S., Es-Sofi A. (2004). DFT and MP2 investigation of the B_2_H_3_^−^ anion potential energy surface. Chem. Phys. Lett..

[117] Krempp M., Damrauer R., DePuy C.H., Keheyan Y. (1994). Gas-Phase Ion Chemistry of Boron Hydride Anions. J. Am. Chem. Soc..

[118] Shore S.G., Johnson H.D. (1970). Deprotonation of tetraborane(10) by ammonia. The temperature-dependent boron-11 nuclear magnetic resonance spectrum of B_4_H_9_^−^. J. Am. Chem. Soc..

[119] Shore S.G., Parry R.W. (1955). The crystalline compound ammonia-borane,1 H_3_NBH_3_. J. Am. Chem. Soc..

[120] Boocock S.K., Toft M.A., Inkrott K.E., Hsu L.Y., Huffman J.C., Folting K., Shore S.G. (1984). Irida-, rhoda-, nickela-, and cuprapentaboranes derived from the arachno-[B4H9]- ion. Crystal structure of [Ir(η^4^-B_4_H_9_)(CO){P(CH_3_)_2_C_6_H_5_}_2_], an analog of arachno-B_5_H_11_ and [Ir(η^4^-C_4_H_6_)(CO){P(CH_3_)_2_C_6_H_5_}_2_]^+^. Inorg. Chem..

[121] Kawano Y., Matsumoto H., Shimoi M. (1999). Syntheses of Diruthenaborane Clusters, ({Cp*Ru(µ-H)}_2_B_3_H_7_), ({(Cp*Ru)_2_(µ-H)}B_4_H_9_), and ((Cp*Ru)_2_(µ-H)(PMe_3_)(µ-η^4^-B_2_H_5_)). Chem. Lett..

[122] Lenczyk C., Roy D.K., Oberdorf K., Nitsch J., Dewhurst R.D., Radacki K., Halet J.-F., Marder T.B., Bickelhaupt F.M., Braunschweig H. (2019). Toward Transition-Metal-Templated Construction of Arylated B_4_ Chains by Dihydroborane Dehydrocoupling. Chem. Eur. J..

[123] Hertz R.K., Denniston M.L., Shore S.G. (1978). Preparation and characterization of B_2_H_4_∙2P(CH_3_)_3_. Inorg. Chem..

[124] Dodds A.R., Kodama G. (1979). Isolation and characterization of trimethylamine-tetraborane(8). Inorg. Chem..

[125] Kameda M., Shimoi M., Kodama G. (1984). Tetraborane(8) adducts of strongly basic phosphines. Inorg. Chem..

[126] Snow S.A., Kodama G. (1985). Novel coordination of a neutral borane adduct to nickel(0). Formation of Ni(CO)_2_[B_2_H_4_∙2P(CH_3_)_3_]. Inorg. Chem..

[127] Shimoi M., Katoh K., Kawano Y., Kodama G., Ogino H. (2002). Fluxional behavior of chromium and tungsten complexes of monodentate bis(trimethylphosphine)diborane(4), [M(CO)_5_(η^1^-B_2_H_4_·2PMe_3_)] (M = Cr, W):. J. Organomet. Chem..

[128] Schleyer P.V.R., Najafian K., Mebel A.M. (1998). The Large closo-Borane Dianions, B_n_H_n_^2−^ (n = 13–17) Are Aromatic, Why Are They Unknown?. Inorg. Chem..

[129] Derecskei-Kovacs A., Dunlap B.I., Lipscomb W.N., Lowrey A., Marynick D.S., Massa L. (1994). Quantum Chemical Studies of Boron Fullerene Analogs. Inorg. Chem..

[130] Wang Z.-X., Schleyer P.V.R. (2003). A “Sea Urchin” Family of Boranes and Carboranes: The 6m + 2n Electron Rule. J. Am. Chem. Soc..

[131] Balakrishnarajan M.M., Hoffmann R., Pancharatna P.D., Jemmis E.D. (2003). Magic Electron Counts and Bonding in Tubular Boranes. Inorg. Chem..

[132] Pancharatna P., Marutheeswaran S., Austeria M.P., Balakrishnarajan M.M. (2013). Deltahedra with holes: Structural preferences of supraicosahedral boranes. Polyhedron.

[133] Shen Y.-F., Xu C., Cheng L. (2017). Deciphering chemical bonding in B_n_H_n_^2−^(n = 2–17): Flexible multicenter bonding. RSC Adv..

[134] Wade K. (1971). The structural significance of the number of skeletal bonding electron-pairs in carboranes, the higher boranes and borane anions, and various transition-metal carbonyl cluster compounds. J. Chem. Soc. D.

[135] Mark A.F., Ken W. (2003). Evolving patterns in boron cluster chemistry. Pure Appl. Chem..

[136] Bicerano J., Marynick D.S., Lipscomb W.N. (1978). Large closo boron hydrides. Inorg. Chem..

[137] Bregadze V.I., Timofeev S.V., Sivaev I.B., Lobanova I.A. (2004). Substitution reactions at boron atoms in metallacarboranes. Russ. Chem. Rev..

[138] Zhao Y., Truhlar D.G. (2008). The M06 suite of density functionals for main group thermochemistry, thermochemical kinetics, noncovalent interactions, excited states, and transition elements: Two new functionals and systematic testing of four M06 functionals and 12 other functionals. Theor. Chem. Accounts.

[139] Frisch M.J., Trucks G.W., Schlegel H.B., Scuseria G.E., Robb M.A., Cheeseman J.R., Scalmani G., Barone V., Mennucci B., Petersson G.A. (2009). Gaussian 09, Revision D.01.

[140] Krishnan R., Binkley J.S., Seeger R., Pople J.A. (1980). Self-consistent molecular orbital methods. XX. A basis set for correlated wave functions. J. Chem. Phys..

[141] Adrienko G.A. Chemcraft, Version 1.8 (build 574b). ChemCraft-Graphical Program for Visualisation of Quantum Chemistry Computations. http://www.chemcraftprog.com.

[142] Marenich A.V., Cramer C.J., Truhlar D.G. (2009). Universal Solvation Model Based on Solute Electron Density and on a Continuum Model of the Solvent Defined by the Bulk Dielectric Constant and Atomic Surface Tensions. J. Phys. Chem. B.

[143] Waldie K.M., Ostericher A.L., Reineke M.H., Sasayama A.F., Kubiak C.P. (2018). Hydricity of Transition-Metal Hydrides: Thermodynamic Considerations for CO_2_ Reduction. ACS Catal..

[144] Pitzer K.S. (1945). Electron Deficient Molecules. I. The Principles of Hydroboron Structures. J. Am. Chem. Soc..

[145] Goebbert D.J., Hernandez H., Francisco J.S., Wenthold P.G. (2005). The Binding Energy and Bonding in Dialane. J. Am. Chem. Soc..

[146] Kühl O. (2011). The coordination chemistry of the proton. Chem. Soc. Rev..

[147] Kaese M.S.T., Budy B.S.H., Bolte M., Lerner H.-W., Wagner M. (2017). Deprotonation of a Seemingly Hydridic Diborane(6) To Build a B−B Bond. Angew. Chem. Int. Ed..

[148] King R.B. (2001). Three-dimensional aromaticity in polyhedral boranes and related molecules. Chem. Rev..

