# Supplementary material for: Thermodynamic Hydricity of Small Borane Clusters and Polyhedral closo-Boranes
Source: Molecules. 2020 Jun 25;25(12):2920. doi: 10.3390/molecules25122920 (PMC7357072; doi:10.3390/molecules25122920)
Supplement: Supplementary file 1 [file molecules-25-02920-s001.pdf]

Thermodynamic Hydricity of Small Borane Clusters and Polyhedral *closo*-Boranes †

Igor E. Golub [1,*], Oleg A. Filippov [1], Vasilisa A. Kulikova [1,2], Natalia V. Belkova [1], Lina M. Epstein [1] and Elena S. Shubina [1,*]

1 A. N. Nesmeyanov Institute of Organoelement Compounds and Russian Academy of Sciences (INEOS RAS), 28 Vavilova St, 119991 Moscow, Russia; h-bond@ineos.ac.ru (O.A.F.); kullisa99@gmail.com (V.A.K.); nataliabelk@ineos.ac.ru (N.V.B.); epst@ineos.ac.ru (L.M.E.)

2 Faculty of Chemistry, M.V. Lomonosov Moscow State University, 1/3 Leninskiye Gory, 119991 Moscow, Russia

* Correspondence: seraph347@gmail.com (I.E.G.); shu@ineos.ac.ru (E.S.S.)

† Dedicated to Professor Bohumil Štibr (1940-2020), who unfortunately passed away before he could reach the age of 80, in the recognition of his outstanding contributions to boron chemistry.

Supplementary Materials

Scheme S1. Scheme of homologous series of neutral and monoanionic boranes clusters. **S3**

Scheme S2. Homologous series of neutral and dianionic boranes clusters. **S3**

Figure S1. Plots of HDA^{MeCN} against the number of boron atoms in borane clusters derived from different starting species. **S4**

Table S1. Computed [M06/6-311++G(d,p)] B–H terminal bond length (in Å), hydride donating ability (HDA^{MeCN} alias $\Delta G°_{[H]^{-}MeCN}$ in kcal/mol) and enthalpy of hydride detaching reaction ($\Delta G°_{[H]^{-}MeCN}$ in kcal/mol) for $Li[B_nH_{3n+1}]$ series. **S4**

Table S2. Computed [M06/6-311++G(d,p)] B–H terminal bond length (in Å), hydride donating ability (HDA^{MeCN} alias $\Delta G°_{[H]^{-}MeCN}$ in kcal/mol) and enthalpy of hydride detaching reaction ($\Delta G°_{[H]^{-}MeCN}$ in kcal/mol) for B_nH_{3n} series. **S5**

Table S3. Difference in Gibbs energy ($\Delta G°_{MeCN}$ in kcal/mol) relative to the most stable isomer of B_3H_9 in MeCN ($\Delta G°_{MeCN}$ in kcal/mol), B–H terminal bond length (in Å), hydride donating ability (HDA^{MeCN} alias $\Delta G°_{[H]^{-}MeCN}$ in kcal/mol) and enthalpy of hydride detaching reaction ($\Delta G°_{[H]^{-}MeCN}$ in kcal/mol). **S5**

Scheme S3. $Li_2[B_nH_{3n}]$ series. Black numbers denote HDA^{MeCN} (in kcal/mol), red numbers in square brackets is difference in Gibbs energy ($\Delta G°_{MeCN}$ in kcal/mol) relative to the most stable isomer. Lithium atoms are omitted for clarity. **S6**

Table S4. Computed [M06/6-311++G(d,p)] B–H terminal bond length (in Å), hydride donating ability (HDA^{MeCN} alias $\Delta G°_{[H]^{-}MeCN}$ in kcal/mol) and enthalpy of hydride detaching reaction ($\Delta G°_{[H]^{-}MeCN}$ in kcal/mol) for $Li_2[B_nH_{3n}]$ series. **S6**

Scheme S4. $Li[B_nH_{3n-1}]$ series. Black numbers denote HDA^{MeCN} (in kcal/mol), red numbers in square brackets is difference in Gibbs energy ($\Delta G°_{MeCN}$ in kcal/mol) relative to the most stable isomer. Lithium atoms are omitted for clarity. **S7**

Table S5. Computed [M06/6-311++G(d,p)] B–H terminal bond length (in Å), hydride donating ability (HDA^{MeCN} alias $\Delta G°_{[H]^{-}MeCN}$ in kcal/mol) and enthalpy of hydride detaching reaction ($\Delta G°_{[H]^{-}MeCN}$ in kcal/mol) for $Li[B_nH_{3n-1}]$ series. **S7**

Scheme S5. B_nH_{3n-2} series. Black numbers denote HDA^{MeCN} (in kcal/mol), red numbers in square brackets is difference in Gibbs energy ($\Delta G°_{MeCN}$ in kcal/mol) relative to the most stable isomer. **S8**

Table S6. Computed [M06/6-311++G(d,p)] B–H terminal bond length (in Å), hydride donating ability (HDA^{MeCN} alias $\Delta G^{\circ}_{[H]^{-}MeCN}$ in kcal/mol) and enthalpy of hydride detaching reaction ($\Delta G^{\circ}_{[H]^{-}MeCN}$ in kcal/mol) for B_nH_{3n-2} series. **S8**

Scheme S6. $Li_2[B_nH_{3n-2}]$ series. Black numbers denote HDA^{MeCN} (in kcal/mol), red numbers in square brackets is difference in Gibbs energy (ΔG°_{MeCN} in kcal/mol) relative to the most stable isomer. Lithium atoms are omitted for clarity. **S9**

Table S7. Computed [M06/6-311++G(d,p)] B–H terminal bond length (in Å), hydride donating ability (HDA^{MeCN} alias $\Delta G^{\circ}_{[H]^{-}MeCN}$ in kcal/mol) and enthalpy of hydride detaching reaction ($\Delta G^{\circ}_{[H]^{-}MeCN}$ in kcal/mol) for $Li_2[B_nH_{3n-2}]$ series. **S10**

Scheme S7. $Li[B_nH_{3n-3}]$ series. Black numbers denote HDA^{MeCN} (in kcal/mol), red numbers in square brackets is difference in Gibbs energy (ΔG°_{MeCN} in kcal/mol) relative to the most stable isomer. Lithium atoms are omitted for clarity. **S11**

Table S8. Computed [M06/6-311++G(d,p)] B–H terminal bond length (in Å), hydride donating ability (HDA^{MeCN} alias $\Delta G^{\circ}_{[H]^{-}MeCN}$ in kcal/mol) and enthalpy of hydride detaching reaction ($\Delta G^{\circ}_{[H]^{-}MeCN}$ in kcal/mol) for $Li[B_nH_{3n-3}]$ series. **S12**

Scheme S8. B_nH_{3n-4} series. Black numbers denote HDA^{MeCN} (in kcal/mol), red numbers in square brackets is difference in Gibbs energy (ΔG°_{MeCN} in kcal/mol) relative to the most stable isomer. **S13**

Table S9. Computed [M06/6-311++G(d,p)] B–H terminal bond length (in Å), hydride donating ability (HDA^{MeCN} alias $\Delta G^{\circ}_{[H]^{-}MeCN}$ in kcal/mol) and enthalpy of hydride detaching reaction ($\Delta G^{\circ}_{[H]^{-}MeCN}$ in kcal/mol) for B_nH_{3n-4} series. **S14**

Structural features of polyhedral closo-boranes **S15**

Figure S2. NPA charge distribution (showed in blue-green-red scale from −0.40 to 0.05), calculated for M06-optimized geometries of dianions $[B_nH_n]^{2-}$ (n = 5–17) of polyhedral closo-boranes in MeCN. **S16**

Table S10. Coordination numbers (CN) of boron atom in polyhedral closo-boranes. Computed [M06/6-311++G(d,p)] B–H terminal bond length (in Å), hydride donating ability (HDA^{MeCN} alias $\Delta G^{\circ}_{[H]^{-}MeCN}$ in kcal/mol) and enthalpy of hydride detaching reaction ($\Delta H^{\circ}_{[H]^{-}MeCN}$ in kcal/mol). **S17**

Table S11. Computed [M06/6-311++G(d,p)] B–H terminal bond length (in Å), hydride donating ability (HDA^{MeCN} alias $\Delta G^{\circ}_{[H]^{-}MeCN}$ in kcal/mol) and enthalpy of hydride detaching reaction ($\Delta G^{\circ}_{[H]^{-}MeCN}$ in kcal/mol). **S18**

Figure S3. HDA^{MeCN} of polyhedral closo-boranes $Li_2[B_nH_n]$ (n = 5–17). Blue columns represent lowest HDA^{MeCN} values, red columns – highest. **S19**

Scheme S9. General trend of HDA^{MeCN} for $Li_2[B_nH_n]$ (n = 2–4). **S19**

Scheme S10. General trend of HDA^{MeCN} for polyhedral closo-boranes $Li_2[B_nH_n]$ (n = 5–17). **S19**

Figure S4. Plot HDA^{MeCN} vs bond length of terminal B–H bond for Li salts polyhedral closo-boranes (green triangles), neutral and anionic borane clusters (red circles) and tetracoordinated boron hydrides (blue squares). HDA^{MeCN} for tetracoordinated boron hydrides are taken from ref.[5] **S20**

Figure S5. Free Gibbs energy per BH unit calculated for dianions $[B_nH_n]^{2-}$ (n = 5–17) of polyhedral closo-boranes and their Li-salts $Li_2[B_nH_n]$ in (n = 5–17) MeCN vs number boron atoms. **S21**

Figure S6. Graph of normalized lowest HDA^{MeCN} of and free Gibbs energy per BH unit for dianions $[B_nH_n]^{2-}$ (n = 5–17) of polyhedral closo-boranes vs number boron atoms. **S21**

Table S12. DFT-optimized geometries (Cartesian coordinates) and electronic energies **S22–S42**

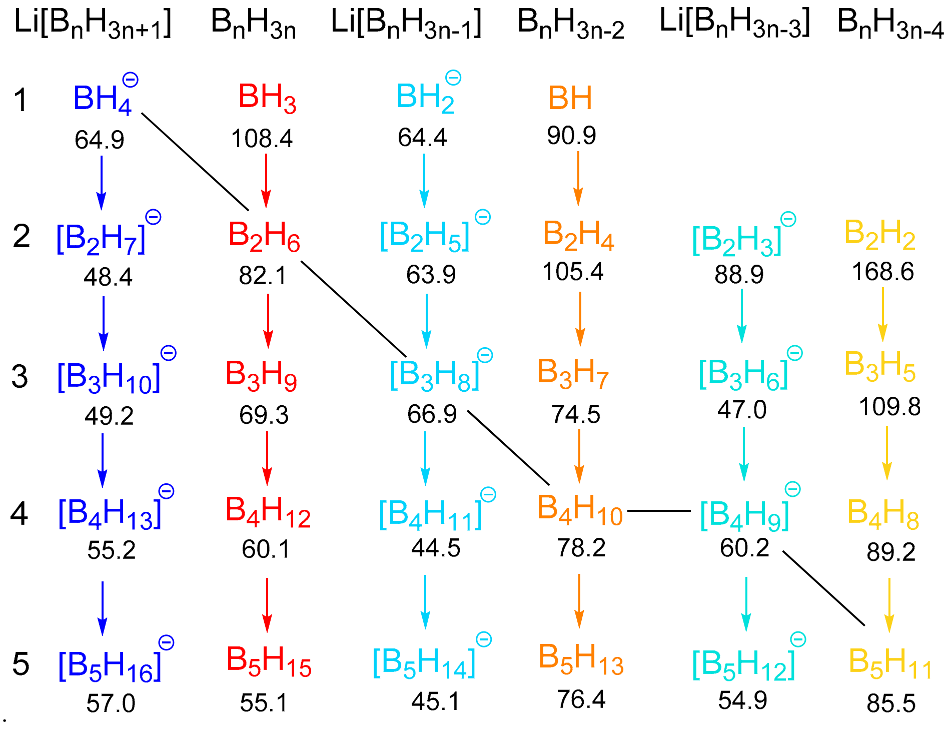

Scheme S1. Scheme of homologous series of neutral and monoanionic boranes clusters. The values of HDA^{MeCN} are given in black for the most energetically favorable isomer. Lithium atoms are omitted for clarity.

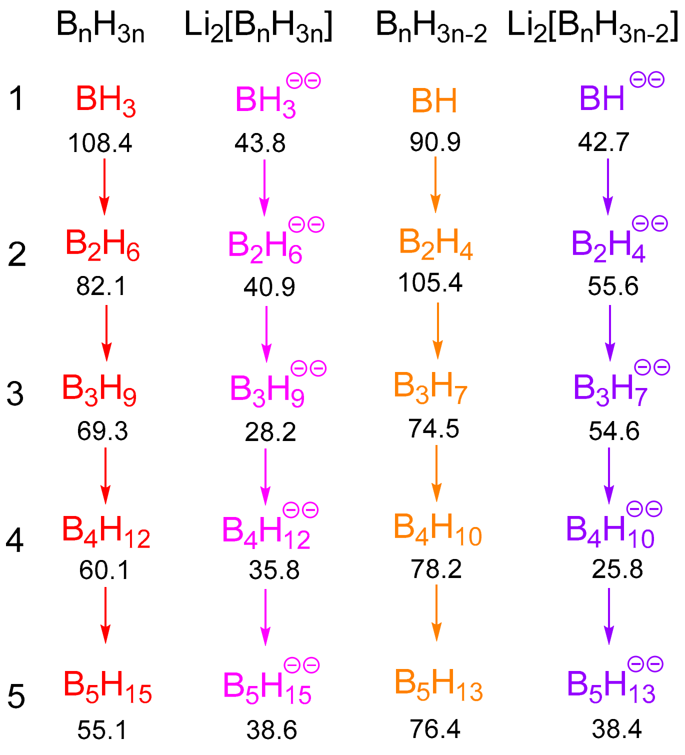

Scheme S2. Homologous series of neutral and dianionic boranes clusters. The values of HDA^{MeCN} are given in black for the most energetically favorable isomer. Lithium atoms are omitted for clarity.

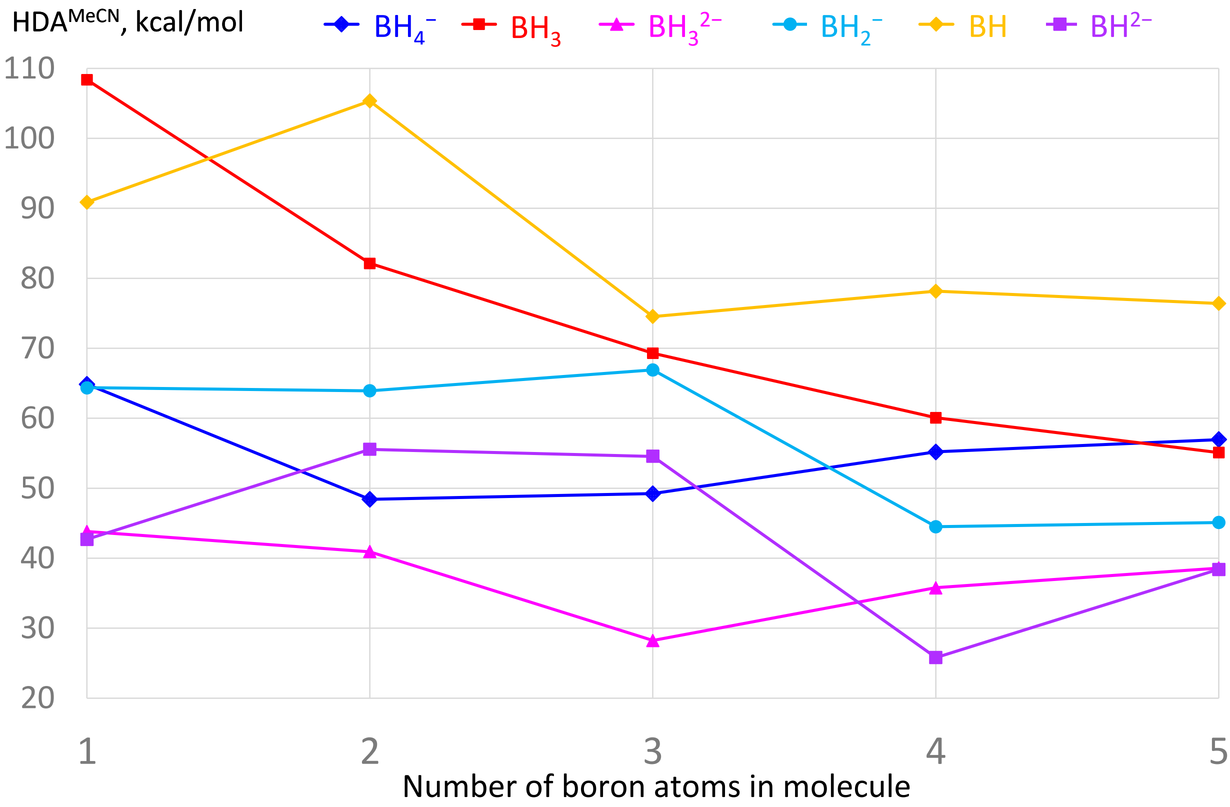

Figure S1. Plots of HDA^{MeCN} against the number of boron atoms in borane clusters derived from different starting species.

Table S1. Computed [M06/6-311++G(d,p)] B–H terminal bond length (in Å), hydride donating ability (HDA^{MeCN} alias $\Delta G°_{[H]^{-}MeCN}$ in kcal/mol) and enthalpy of hydride detaching reaction ($\Delta G°_{[H]^{-}MeCN}$ in kcal/mol) for Li[B_nH_{3n+1}] series.

Borohydride	$\Delta G°_{MeCN}$	r(B–H)	HDA^{MeCN}	$\Delta H°_{[H]^{-}MeCN}$
**$LiBH_4$**	–	1.206	64.86	72.77
**Li[B_2H_7]**	–	1.214	48.42	54.96
**Li[B_3H_{10}]**	–	1.210	49.22	55.54
***n*-Li[B_4H_{13}]**	0.0	1.206	55.20	62.13
***i*-Li[B_4H_{13}]**	5.2	1.207	55.65	61.78
***n*-Li[B_5H_{16}]**	0.0	1.204	56.95	62.61
***i*-Li[B_5H_{16}]**	5.9	1.212	59.36	66.05
***t*-Li[B_5H_{16}]**	13.5	1.208	48.96	54.29

n – normal, *i* – isomeric, *t* – tertiary.

Table S2. Computed [M06/6-311++G(d,p)] B–H terminal bond length (in Å), hydride donating ability (HDA^{MeCN} alias $\Delta G°_{[H]^{-}MeCN}$ in kcal/mol) and enthalpy of hydride detaching reaction ($\Delta G°_{[H]^{-}MeCN}$ in kcal/mol) for B_nH_{3n} series.

Borohydride	$\Delta G°_{MeCN}$	r(B–H)	HDA^{MeCN}	$\Delta H°_{[H]^{-} MeCN}$
BH_3	–	1.186	108.38	114.43
B_2H_6	–	1.186	82.12	89.19
lin-B_3H_9	0.0	1.202	69.32	75.69
btf-B_3H_9	0.5	1.190	68.51	75.75
cyc-B_3H_9	2.3	1.193	72.62	79.54
lin-B_4H_{12}	0.0	1.204	60.08	66.17
pinc-B_4H_{12}	4.5	1.200	55.36	60.16
cyc-B_4H_{12}	9.5	1.193	65.13	71.45
lin-B_5H_{15}	0.0	1.202	55.09	60.85
i-B_5H_{15}	3.9	1.200	62.40	70.67
pinc-B_5H_{15}	7.1	1.203	59.10	66.07
cyc-B_5H_{15}	8.1	1.197	65.35	72.10

lin – linear, *btf* – butterfly-like, *cyc* – cyclic, *pinc* – pincer-like, *i* – isomeric.

Table S3. Difference in Gibbs energy ($\Delta G°_{MeCN}$ in kcal/mol) relative to the most stable isomer of B_3H_9 in MeCN ($\Delta G°_{MeCN}$ in kcal/mol), B–H terminal bond length (in Å), hydride donating ability (HDA^{MeCN} alias $\Delta G°_{[H]^{-}MeCN}$ in kcal/mol) and enthalpy of hydride detaching reaction ($\Delta G°_{[H]^{-}MeCN}$ in kcal/mol).

MP2/6-311++G(d,p)	$\Delta G°_{MeCN}$	r(B–H)	HDA^{MeCN}	$\Delta H°_{[H]^{-}MeCN}$
$B_2H_5(\mu$-H)BH_3 (*lin*-B_3H_9)	0.0	1.203	72.18	78.50
btf-B_3H_9	0.8	1.191	71.33	78.63
cyc-B_3H_9	1.3	1.191	76.61	83.42

M06/6-311++G(d,p)	$\Delta G°_{MeCN}$	r(B–H)	HDA^{MeCN}	$\Delta H°_{[H]^{-}MeCN}$
$B_2H_5(\mu$-H)BH_3 (*lin*-B_3H_9)	0.0	1.202	69.32	75.69
btf-B_3H_9	0.5	1.190	68.51	75.75
cyc-B_3H_9	2.3	1.193	72.62	79.54

lin – linear, *btf* – butterfly-like, *cyc* – cyclic.

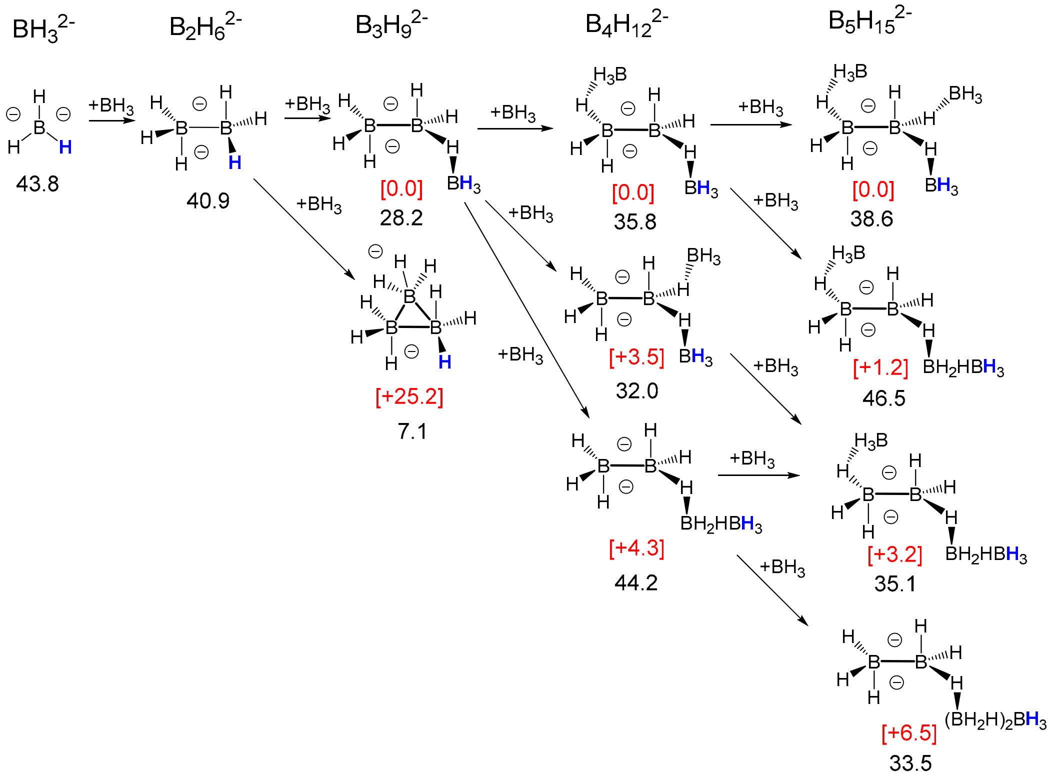

Scheme S3. $Li_2[B_nH_{3n}]$ series. Black numbers denote HDA^{MeCN} (in kcal/mol), red numbers in square brackets is difference in Gibbs energy ($\Delta G°_{MeCN}$ in kcal/mol) relative to the most stable isomer. Lithium atoms are omitted for clarity.

Table S4. Computed [M06/6-311++G(d,p)] B–H terminal bond length (in Å), hydride donating ability (HDA^{MeCN} alias $\Delta G°_{[H]^- MeCN}$ in kcal/mol) and enthalpy of hydride detaching reaction ($\Delta G°_{[H]^- MeCN}$ in kcal/mol) for $Li_2[B_nH_{3n}]$ series.

Borohydride	$\Delta G°_{MeCN}$	r(B–H)	HDA^{MeCN}	$\Delta H°_{[H]^- MeCN}$
**Li_2BH_3**	–	1.237	43.82	52.34
**$Li_2[B_2H_6]$**	–	1.241	40.94	48.55
***lin*-$Li_2[B_3H_9]$**	0.0	1.248	28.24	35.02
***cyc*-$Li_2[B_3H_9]$**	25.2	1.227	7.05	14.56
***1,2*-$Li_2[B_4H_{12}]$**	0.0	1.236	35.78	42.71
***1,1*-$Li_2[B_4H_{12}]$**	3.5	1.229	32.04	38.24
***lin*-$Li_2[B_4H_{12}]$**	4.3	1.233	44.16	51.41
***pinc*-$Li_2[B_5H_{12}]$**	0.0	1.218	38.55	44.71
***1,2*-$Li_2[B_5H_{12}]$**	1.2	1.228	46.53	53.50
***1,1*-$Li_2[B_5H_{12}]$**	3.2	1.235	35.05	40.50
***lin*-$Li_2[B_5H_{12}]$**	6.5	1.253	33.54	39.92

lin – linear, *cyc* – cyclic, *pinc* – pincer-like.

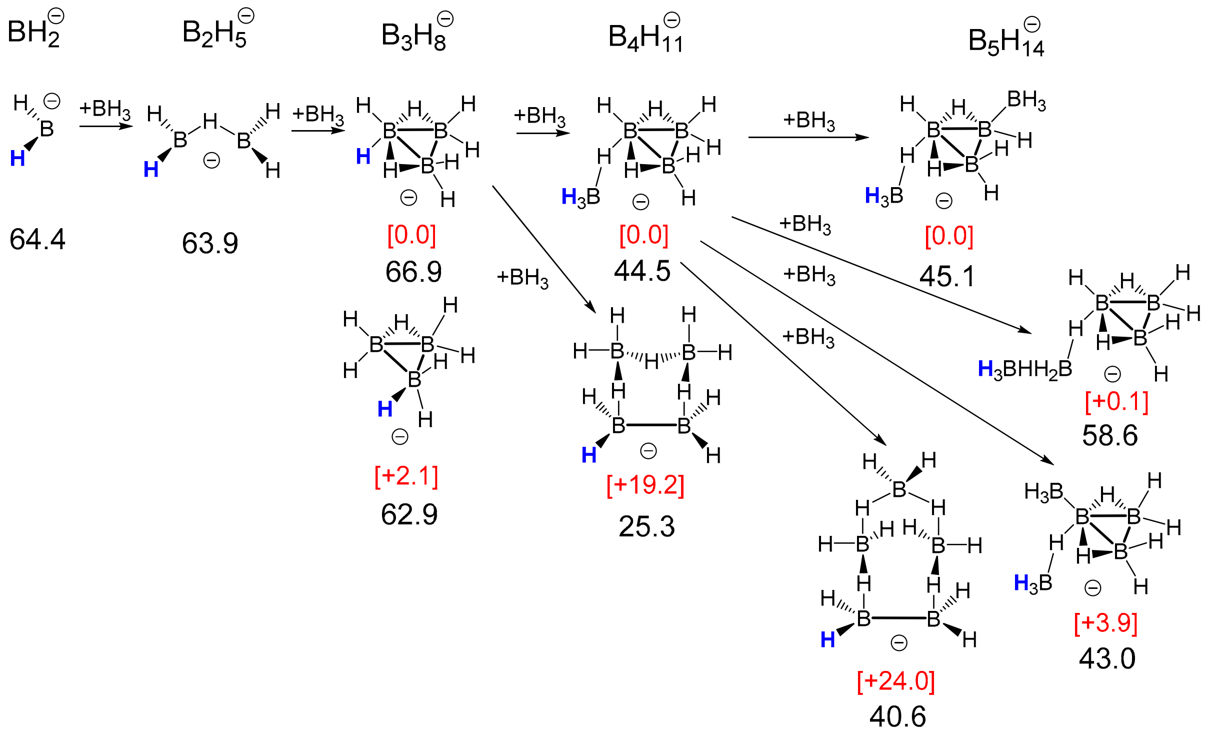

Scheme S4. $Li[B_nH_{3n-1}]$ series. Black numbers denote HDA^{MeCN} (in kcal/mol), red numbers in square brackets is difference in Gibbs energy ($\Delta G°_{MeCN}$ in kcal/mol) relative to the most stable isomer. Lithium atoms are omitted for clarity.

Table S5. Computed [M06/6-311++G(d,p)] B–H terminal bond length (in Å), hydride donating ability (HDA^{MeCN} alias $\Delta G°_{[H]^{-}MeCN}$ in kcal/mol) and enthalpy of hydride detaching reaction ($\Delta G°_{[H]^{-}MeCN}$ in kcal/mol) for $Li[B_nH_{3n-1}]$ series.

Borohydride	$\Delta G°_{MeCN}$	r(B–H)	HDA^{MeCN}	$\Delta H°_{[H]^{-}\ MeCN}$
**$LiBH_2$**	–	1.220	64.36	66.64
**$Li[B_2H_5]$**	–	1.217	63.94	71.81
**$Li[B_3H_8]$ conf0**	0.0	1.208	66.91	74.65
**$Li[B_3H_8]$ conf1**	2.1	1.222	62.85	70.95
***lin*-$Li[B_4H_{11}]$**	0.0	1.208	44.50	51.54
***cyc*-$Li[B_4H_{11}]$**	19.2	1.227	25.27	32.48
***1,2*-$Li[B_5H_{14}]$**	0.0	1.204	45.11	51.04
***lin*-$Li[B_5H_{14}]$**	0.1	1.207	58.64	65.88
***1,1*-$Li[B_5H_{14}]$**	3.9	1.205	43.03	48.81
***cyc*-$Li[B_5H_{14}]$**	24.0	1.228	40.60	45.51

lin – linear, cyc – cyclic.

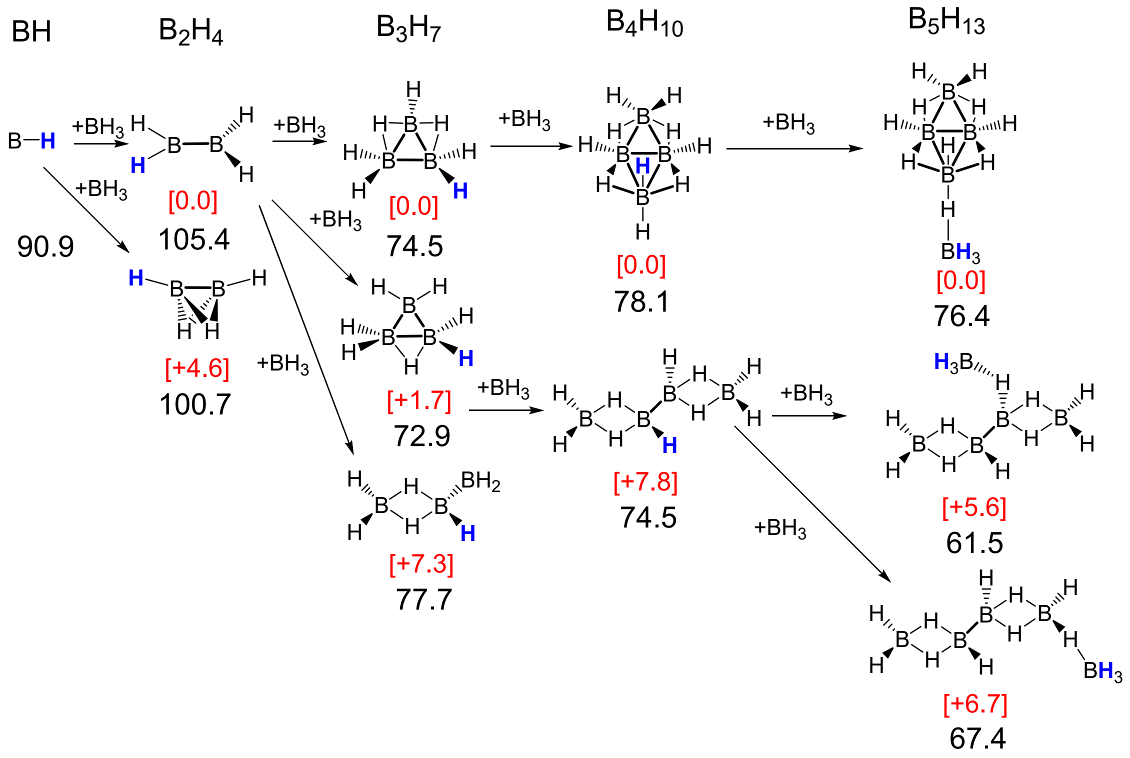

Scheme S5. B_nH_{3n-2} series. Black numbers denote HDA^{MeCN} (in kcal/mol), red numbers in square brackets is difference in Gibbs energy ($\Delta G°_{MeCN}$ in kcal/mol) relative to the most stable isomer.

Table S6. Computed [M06/6-311++G(d,p)] B–H terminal bond length (in Å), hydride donating ability (HDA^{MeCN} alias $\Delta G°_{[H]^{-}MeCN}$ in kcal/mol) and enthalpy of hydride detaching reaction ($\Delta H°_{[H]^{-}MeCN}$ in kcal/mol) for B_nH_{3n-2} series.

Borohydride	$\Delta G°_{MeCN}$	r(B–H)	HDA^{MeCN}	$\Delta H°_{[H]^{-}MeCN}$
**BH**	–	1.213	90.88	96.30
***st*-B_2H_4**	0.0	1.194	105.35	112.51
***btf*-B_2H_4**	4.6	1.168	100.72	108.64
**$B_3H_5(\mu_2H)_2$ (B_3H_7)**	0.0	1.191	74.54	81.52
**$B_3H_6(\mu_2H)$ (B_3H_7)**	1.7	1.191	72.91	79.67
**$B_2H_5(BH_2)$ (B_3H_7)**	7.3	1.196	77.65	85.01
***arachno*-B_4H_{10}**	0.0	1.192	78.17	84.97
***bis*-B_4H_{10}**	7.8	1.193	74.52	80.94
***arachno*-$B_4H_{10}(BH_3)$**	0.0	1.197	76.40	81.96
***i-bis*-$B_4H_{10}(BH_3)$**	5.6	1.200	61.46	67.19
***n-bis*-$B_4H_{10}(BH_3)$**	6.4	1.203	67.72	73.79

st – staggered, btf – butterfly-like, n – normal, i – isomeric.

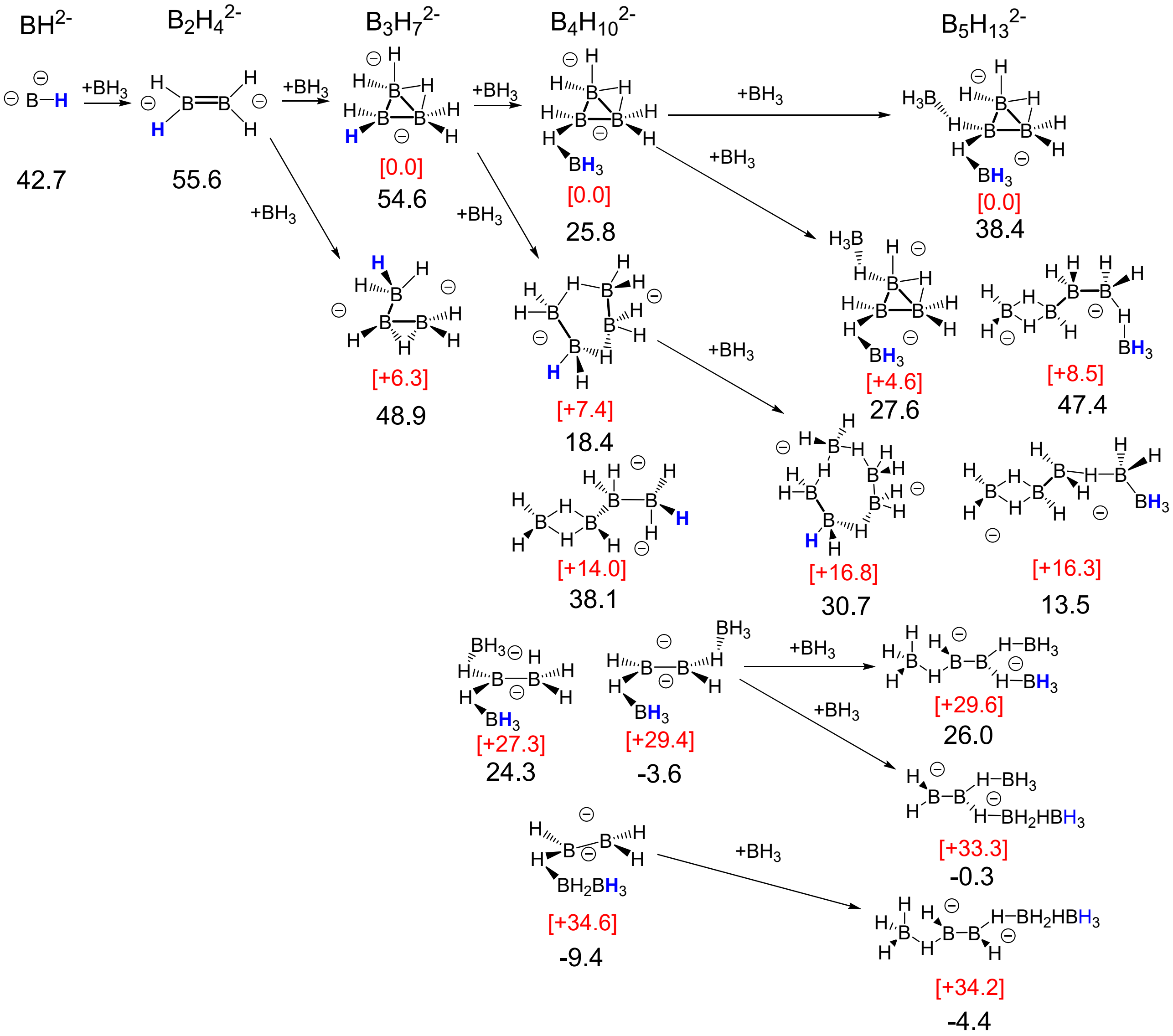

Scheme S6. Li2[BnH3n-2] series. Black numbers denote HDA^{MeCN} (in kcal/mol), red numbers in square brackets is difference in Gibbs energy ($\Delta G°_{MeCN}$ in kcal/mol) relative to the most stable isomer. Lithium atoms are omitted for clarity.

Table S7. Computed [M06/6-311++G(d,p)] B–H terminal bond length (in Å), hydride donating ability (HDA^{MeCN} alias $\Delta G°_{[H]^{-}MeCN}$ in kcal/mol) and enthalpy of hydride detaching reaction ($\Delta G°_{[H]^{-}MeCN}$ in kcal/mol) for $Li_2[B_nH_{3n-2}]$ series.

Borohydride	$\Delta G°_{MeCN}$	r(B–H)	HDA^{MeCN}	$\Delta H°_{[H]^{-} MeCN}$
**Li_2BH**	–	1.222	42.70	48.02
**$Li_2[B_2H_4]$**	–	1.220	55.57	65.00
**$Li_2[B_3H_7]$ conf0**	0.0	1.225	54.56	63.19
**$Li_2[B_3H_7]$ conf1**	6.3	1.238	48.91	57.39
***lin*-$Li_2[B_4H_{10}]$**	0.0	1.227	25.80	32.04
***cyc*-$Li_2[B_4H_{10}]$**	7.4	1.227	18.42	26.16
***bis*-$Li_2[B_4H_{10}]$**	14.0	1.250	38.13	45.35
***pinc-BB*-$Li_2[B_4H_{10}]$**	27.3	1.212	24.28	30.54
***tr-BB*-$Li_2[B_4H_{10}]$**	29.4	1.213	-3.56	2.03
***lin-BB*-$Li_2[B_4H_{10}]$**	34.6	1.219	-9.41	-4.51
***1,1*-$Li_2[B_5H_{13}]$**	0.0	1.215	38.41	46.30
***1,2*-$Li_2[B_5H_{13}]$**	4.6	1.217	27.61	34.17
***bis*-$Li_2[B_5H_{13}]$ conf0**	8.5	1.244	47.43	54.18
***bis*-$Li_2[B_5H_{13}]$ conf1**	16.3	1.249	13.54	18.93
***cyc*-$Li_2[B_5H_{13}]$**	16.8	1.235	30.71	36.27
***pinc-BB*-$Li_2[B_5H_{13}]$**	29.6	1.213	26.03	32.09
***1,1-BB*-$Li_2[B_5H_{13}]$**	33.3	1.212	-0.27	4.59
***tr-BB*-$Li_2[B_5H_{13}]$**	34.2	1.212	-4.38	0.96

lin – linear, *cyc* – cyclic, *pinc* – pincer-like, *tr* – trans.

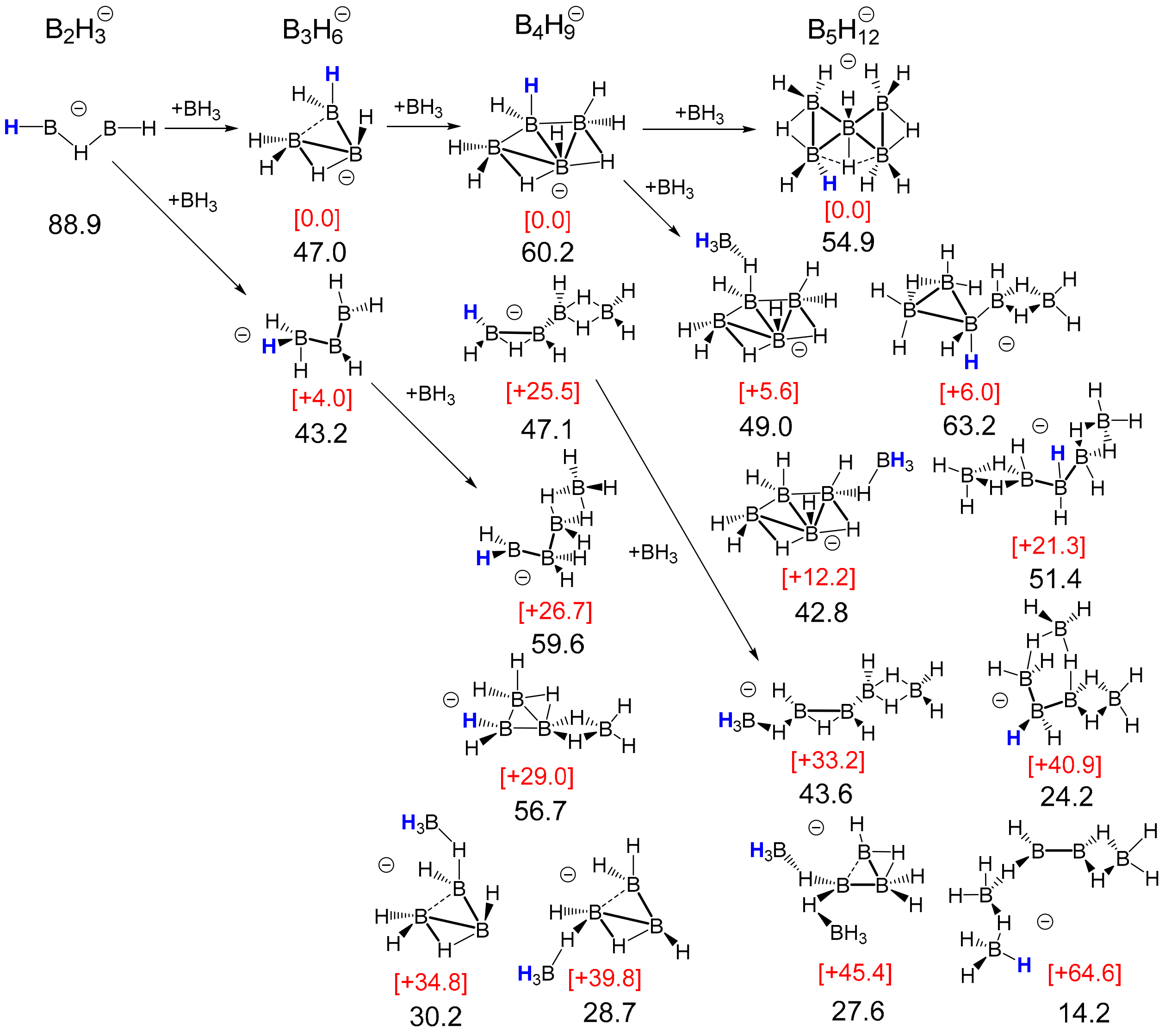

Scheme S7. Li[B_nH_{3n-3}] series. Black numbers denote HDA^{MeCN} (in kcal/mol), red numbers in square brackets is difference in Gibbs energy ($\Delta G°_{MeCN}$ in kcal/mol) relative to the most stable isomer. Lithium atoms are omitted for clarity.

Table S8. Computed [M06/6-311++G(d,p)] B–H terminal bond length (in Å), hydride donating ability (HDA^{MeCN} alias $\Delta G°_{[H]^{-}MeCN}$ in kcal/mol) and enthalpy of hydride detaching reaction ($\Delta G°_{[H]^{-}MeCN}$ in kcal/mol) for $Li[B_nH_{3n-3}]$ series.

Borohydride	$\Delta G°_{MeCN}$	r(B–H)	HDA^{MeCN}	$\Delta H°_{[H]^{-}\ MeCN}$
**$Li[B_2H_3]$**	–	1.178	88.90	96.60
**$Li[B_3H_6]$ conf0**	0.0	1.220	47.01	54.75
**$Li[B_3H_6]$ conf1**	4.0	1.232	43.15	50.42
***arachno*-$Li[B_4H_9]$**	0.0	1.210	60.19	68.19
***bis*-$Li[B_4H_9]$ conf0**	25.5	1.221	47.09	53.49
***bis*-$Li[B_4H_9]$ conf1**	26.7	1.224	59.63	67.94
***lin-BBB*-$Li[B_4H_9]$ conf0**	29.0	1.224	56.68	64.22
***lin-1-BBB*-$Li[B_4H_9]$ conf1**	34.8	1.215	30.18	35.69
***lin-3-BBB*-$Li[B_4H_9]$ conf1**	39.8	1.209	28.70	35.30
***commo*-$Li[B_5H_{12}]$**	0.0	1.205	54.90	61.86
***lin-1*-$Li[B_5H_{12}]$**	5.6	1.212	49.04	55.33
***lin-BBB*-$Li[B_5H_{12}]$**	6.0	1.223	63.20	71.16
***lin-2*-$Li[B_5H_{12}]$**	12.2	1.214	42.77	49.06
***bis-BH_2*-$Li[B_5H_{12}]$**	21.3	1.240	51.41	58.82
***lin-bis*-$Li[B_5H_{12}]$**	33.2	1.212	43.63	51.55
***lin-cyc*-$Li[B_5H_{12}]$**	40.9	1.234	24.20	30.99
***1,1-BBB*-$Li[B_5H_{12}]$**	45.4	1.213	11.20	15.49
***lin-BB*-$Li[B_5H_{12}]$**	64.6	1.212	14.19	20.89

lin – linear, *cyc* – cyclic.

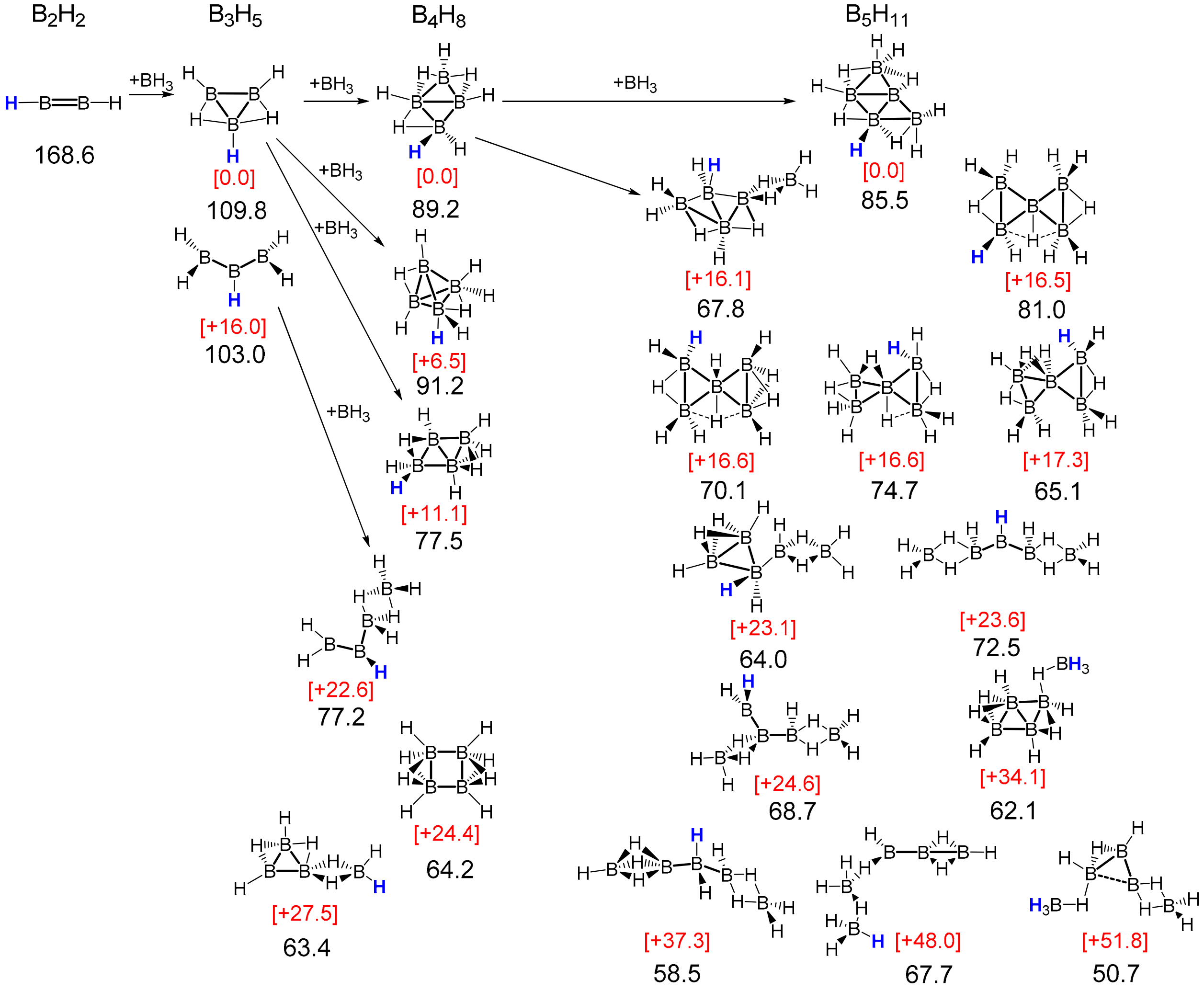

Scheme S8. B_nH_{3n-4} series. Black numbers denote HDA^{MeCN} (in kcal/mol), red numbers in square brackets is difference in Gibbs energy ($\Delta G°_{MeCN}$ in kcal/mol) relative to the most stable isomer.

Table S9. Computed [M06/6-311++G(d,p)] B–H terminal bond length (in Å), hydride donating ability (HDA^{MeCN} alias $\Delta G°_{[H]^{-}MeCN}$ in kcal/mol) and enthalpy of hydride detaching reaction ($\Delta G°_{[H]^{-}MeCN}$ in kcal/mol) for B_nH_{3n-4} series.

Borohydride	$\Delta G°_{MeCN}$	r(B–H)	HDA^{MeCN}	$\Delta H°_{[H]^{-} MeCN}$
**B_2H_2**	–	1.168	168.58	178.45
**B_3H_5 conf0**	0.0	1.173	109.84	117.88
**B_3H_5 conf1**	16.0	1.202	103.05	110.27
***arachno*-B_4H_8 conf0**	0.0	1.195	89.24	96.90
***disph*-B_4H_8**	6.5	1.191	91.15	98.01
***arachno*-B_4H_8 conf1**	11.1	1.198	77.54	85.03
***bis*-B_4H_8**	22.6	1.200	77.17	84.45
***sq*-B_4H_8**	24.4	1.192	64.24	72.13
***lin-BBB*-B_4H_8**	27.5	1.187	63.37	70.63
***arachno*-B_5H_{11}**	0.0	1.195	85.52	93.14
***lin-1*-B_5H_{11} conf0**	16.1	1.199	67.79	74.57
***commo*-B_5H_{11} conf0**	16.5	1.193	81.04	87.76
***commo*-B_5H_{11} conf1**	16.6	1.199	70.09	77.60
***commo*-B_5H_{11} conf2**	16.6	1.195	74.73	82.65
***commo*-B_5H_{11} conf3**	17.3	1.194	65.12	70.89
***lin-BBB*-B_5H_{11} conf0**	23.1	1.212	63.97	70.32
***bis-BH*-B_5H_{11}**	23.6	1.199	72.54	77.61
***i-bis*-$B_4H_8(BH_3)$**	24.6	1.194	68.67	74.20
***lin-1*-B_5H_{11} conf1**	34.1	1.199	62.11	68.27
***bis-BH_2*-B_5H_{11}**	37.3	1.223	58.51	65.07
***lin-BB*-B_5H_{11}**	48.0	1.201	67.76	74.55
***1,2-BBB*-LiB_5H_{11} conf1**	51.8	1.200	50.67	57.16

disp – disphenoidal, *lin* – linear, *sq* – squared, *i* – isomeric.

Structural features of polyhedral closo-boranes

From $[B_5H_5]^{2-}$ to $[B_7H_7]^{2-}$ a bent of polyhedron represent triangle, square and pentagon, however $[B_6H_6]^{2-}$ have geometry of octahedron with equivalent B–H vertices. In the structure of $[B_8H_8]^{2-}$ consist two caps formed of B_2H_2 fragments and B_4H_4 hypar shaped structure in the heart of the polyhedron belt which is a transitional form between planar and 3D structure patterns forming bent of polyhedron of closo-boranes.

In the next structures bent of polyhedron has shape of trigonal prism for $[B_9H_9]^{2-}$, tetragonal antiprism for $[B_{10}H_{10}]^{2-}$, pentagonal antiprism for $[B_{12}H_{12}]^{2-}$, hexagonal antiprism for $[B_{14}H_{14}]^{2-}$. $[B_{12}H_{12}]^{2-}$ is icosahedron with equivalent B–H vertices, however it neighbors of octadecahedral $[B_{11}H_{11}]^{2-}$ and docosahedral $[B_{13}H_{13}]^{2-}$ having a low symmetry C_{2v}. Polyhedron bent in their structures consists of two molecular subunits, which combined basket-shape and bowl-shape structures, correspondingly. In the next three closo-boranes an evolution of capping structures is observed: triangles in $[B_{15}H_{15}]^{2-}$, squares in $[B_{16}H_{16}]^{2-}$ and pentagonal pyramids in in $[B_{17}H_{17}]^{2-}$. At the same involution of polyhedron bent takes places: hexagonal rhombus loop, contained two types atoms of different reactivity in $[B_{15}H_{15}]^{2-}$, antiprismatic octagon in $[B_{16}H_{16}]^{2-}$ and expanded pentagonal ring in $[B_{17}H_{17}]^{2-}$. In this way $[B_{17}H_{17}]^{2-}$ formally can be formed by breaking icosahedral structure of $[B_{12}H_{12}]^{2-}$ into two nido-B_6H_6 fragments and insertion B_5H_5 pentagonal ring between them [1].

Natural population analysis (Figure S2) showed that electron density concentrates on boron atoms forming skeleton of polyhedron, whereas hydrogen atoms are less negatively charged. Increasing of BH group involved in polyhedron formation causes repolarization of hydrogens in closo-boranes that apparently explains better hydrolytic stability of large polyhedral closo-boranes [2-4]. In $[B_nH_n]^{2-}$ (n = 5–8) hydrogens atoms have partial negative charge (−0.047 ÷ −0.010), in $[B_9H_9]^{2-}$ the close to zero (0.001–0.006), whereas in $[B_nH_n]^{2-}$ (n = 10–17) the partial positive charge on H is observed (0.010–0.051). Terminal B–H groups attached to the most negative charged boron atoms has the shortest bond length. It worth to note that the majority closo-boranes difference between shortest and longest BH bond are 0.04–0.08 Å (Figure 4).

References

1. Balakrishnarajan, M. M.; Hoffmann, R.; Pancharatna, P. D.; Jemmis, E. D., Magic Electron Counts and Bonding in Tubular Boranes. *Inorg. Chem.* **2003,** 42, (15), 4650-4659.
2. King, R. B., Three-Dimensional Aromaticity in Polyhedral Boranes and Related Molecules. *Chem. Rev. (Washington, DC, U. S.)* **2001,** 101, (5), 1119-1152.
3. Schubert, D. M., Boron Hydrides, Heteroboranes, and their Metalla Derivatives. In *Kirk-Othmer Encyclopedia of Chemical Technology*.
4. Muetterties, E., *Boron hydride chemistry*. Elsevier: 1975; p 544.
5. Golub, I. E.; Filippov, O. A.; Belkova, N. V.; Epstein, L. M.; Shubina, E. S., Hydride donating abilities of the tetracoordinated boron hydrides. *J. Organomet. Chem.* **2018,** 865, 247-256.

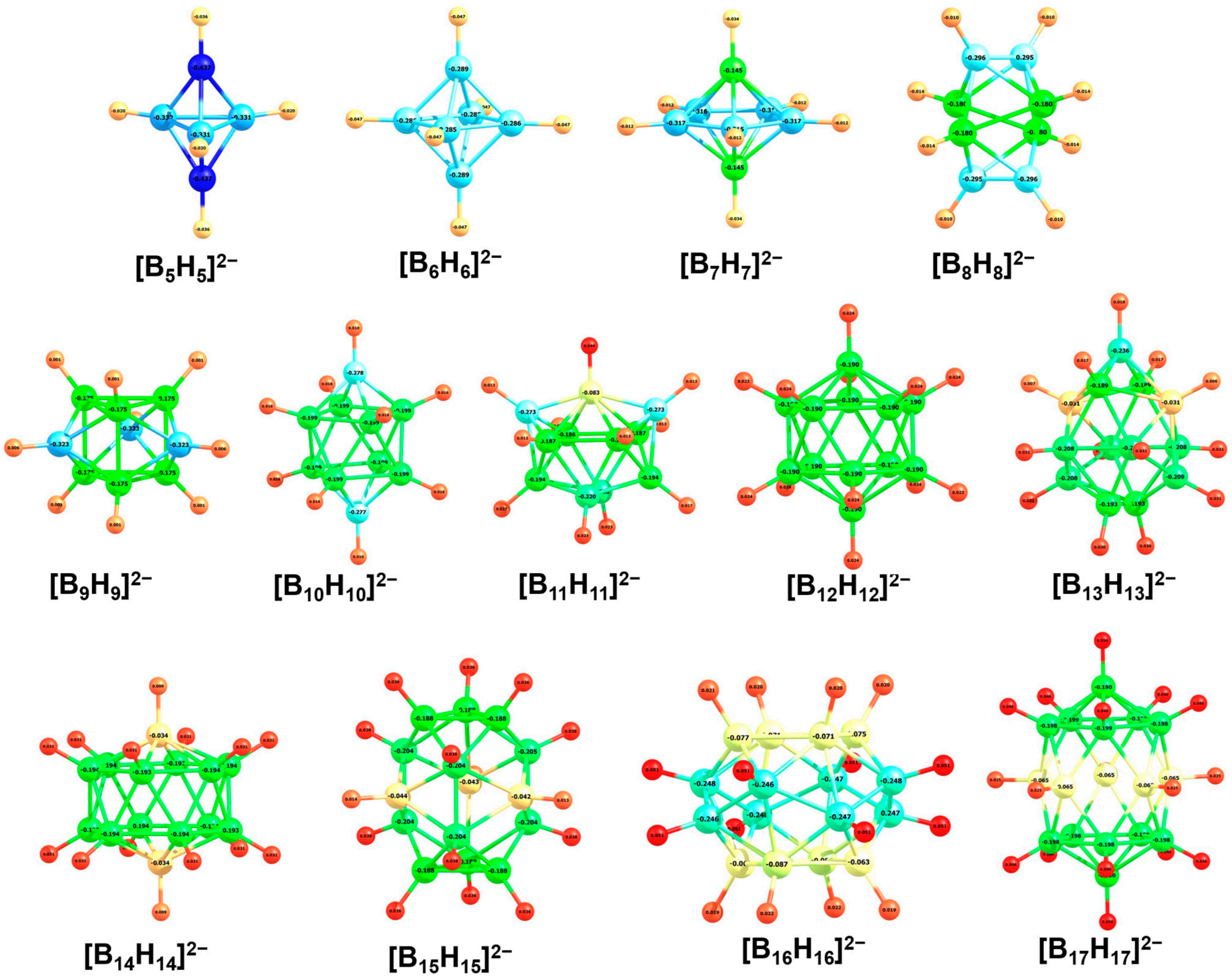

Figure S2. NPA charge distribution (showed in blue-green-red scale from −0.40 to 0.05), calculated for M06-optimized geometries of dianions $[B_nH_n]^{2-}$ (n = 5–17) of polyhedral closo-boranes in MeCN.

Table S10. Coordination numbers (CN) of boron atom in polyhedral closo-boranes. Computed [M06/6-311++G(d,p)] B–H terminal bond length (in Å), hydride donating ability (HDA^{MeCN} alias $\Delta G°_{[H]^{-}MeCN}$ in kcal/mol) and enthalpy of hydride detaching reaction ($\Delta H°_{[H]^{-}MeCN}$ in kcal/mol).

Borane	$CN(B^{ap})$	$CN(B^{eq})$	$r(BH^{ap})$	$r(BH^{eq})$	HDA^{MeCN} (BH^{ap})	$\Delta H°_{[H]^{-}MeCN}$ (BH^{ap})	HDA^{MeCN} (BH^{eq})	$\Delta H°_{[H]^{-}MeCN}$ (BH^{eq})
$[B_5H_5]^{2-}$	4	5	1.194	1.202	81.98	90.31	60.02	68.08
$[B_6H_6]^{2-}$	5		1.200		71.94	80.07	–	–
$[B_7H_7]^{2-}$	6	5	1.205	1.199	56.26	64.07	67.58	75.59
$[B_8H_8]^{2-}$	5	6	1.197	1.203	71.91	80.09	56.79	64.42
$[B_9H_9]^{2-}$	5	6	1.193	1.199	76.19	84.13	70.82	78.90
$[B_{10}H_{10}]^{2-}$	5	6	1.191	1.197	89.01	97.05	75.55	83.51
$[B_{11}H_{11}]^{2-}$	7	5/6	1.199	1.197<br>1.191	74.50	82.40	65.25<br>65.32	72.88<br>72.94
$[B_{12}H_{12}]^{2-}$	6		1.195		79.60	87.52	–	–
$[B_{13}H_{13}]^{2-}$	5	5/6	1.192	1.200<br>1.195<br>1.198<br>1.194	39.49	47.76	39.41<br>39.55<br>71.41<br>80.07	47.69<br>47.80<br>79.98<br>88.64
$[B_{14}H_{14}]^{2-}$	7	6	1.195	1.194	78.99	86.88	75.85	83.79
$[B_{15}H_{15}]^{2-}$	5	5/6	1.192	1.195<br>1.194	83.82	91.90	75.28<br>70.76	83.15<br>78.90
$[B_{16}H_{16}]^{2-}$	6	6	1.194	1.192	74.05	82.04	76.86	84.58
$[B_{17}H_{17}]^{2-}$	6	5/6	1.190<br>1.191	1.197	88.88<br>83.36	97.27<br>91.52	62.38	69.88

Table S11. Computed [M06/6-311++G(d,p)] B–H terminal bond length (in Å), hydride donating ability (HDA^{MeCN} alias $\Delta G°_{[H]^{-}MeCN}$ in kcal/mol) and enthalpy of hydride detaching reaction ($\Delta G°_{[H]^{-}MeCN}$ in kcal/mol).

Borane	r(B–H)	HDA^{MeCN}	$\Delta H°_{[H]^{-}MeCN}$
ap-$Li_2[B_{10}H_{10}]$	1.194	102.12	110.69
ap-$Li_2[B_5H_5]$	1.195	96.87	105.74
ap-$Li_2[B_{15}H_{15}]$	1.198	96.55	105.25
ap-$Li_2[B_{17}H_{17}]$	1.187	95.43	103.05
$Li_2[B_2H_2]$	1.187	92.88	100.69
ap-$Li_2[B_9H_9]$	1.201	92.06	100.13
eq-$Li_2[B_{13}H_{13}]$	1.189	91.17	99.75
$Li_2[B_{12}H_{12}]$	1.189	91.13	99.84
ap-$Li_2[B_{10}H_{10}]$	1.194	89.78	98.13
ap-$Li_2[B_{14}H_{14}]$	1.202	89.54	98.01
$Li_2[B_6H_6]$	1.199	89.32	96.64
eq-$Li_2[B_7H_7]$	1.203	89.20	97.12
ap-$Li_2[B_{11}H_{11}]$	1.194	88.20	96.44
ap-$Li_2[B_8H_8]$	1.201	88.07	96.45
eq-$Li_2[B_{14}H_{14}]$	1.189	87.14	95.38
eq-$Li_2[B_{16}H_{16}]$	1.189	87.10	95.19
ap-$Li_2[B_{16}H_{16}]$	1.202	85.33	93.54
eq-$Li_2[B_9H_9]$	1.193	85.10	93.64
eq-$Li_2[B_{15}H_{15}]$	1.190	83.04	91.66
eq-$Li_2[B_{11}H_{11}]$	1.206	78.34	87.15
eq-$Li_2[B_5H_5]$	1.205	75.98	85.00
eq-$Li_2[B_8H_8]$	1.210	75.19	83.50
eq-$Li_2[B_{17}H_{17}]$	1.192	75.11	82.19
ap-$Li_2[B_7H_7]$	1.197	73.55	80.81
$Li_2[B_3H_3]$	1.209	60.85	69.36
ap-$Li_2[B_{13}H_{13}]$	1.192	51.73	60.24
$Li_2[B_4H_4]$	1.205	41.34	49.28

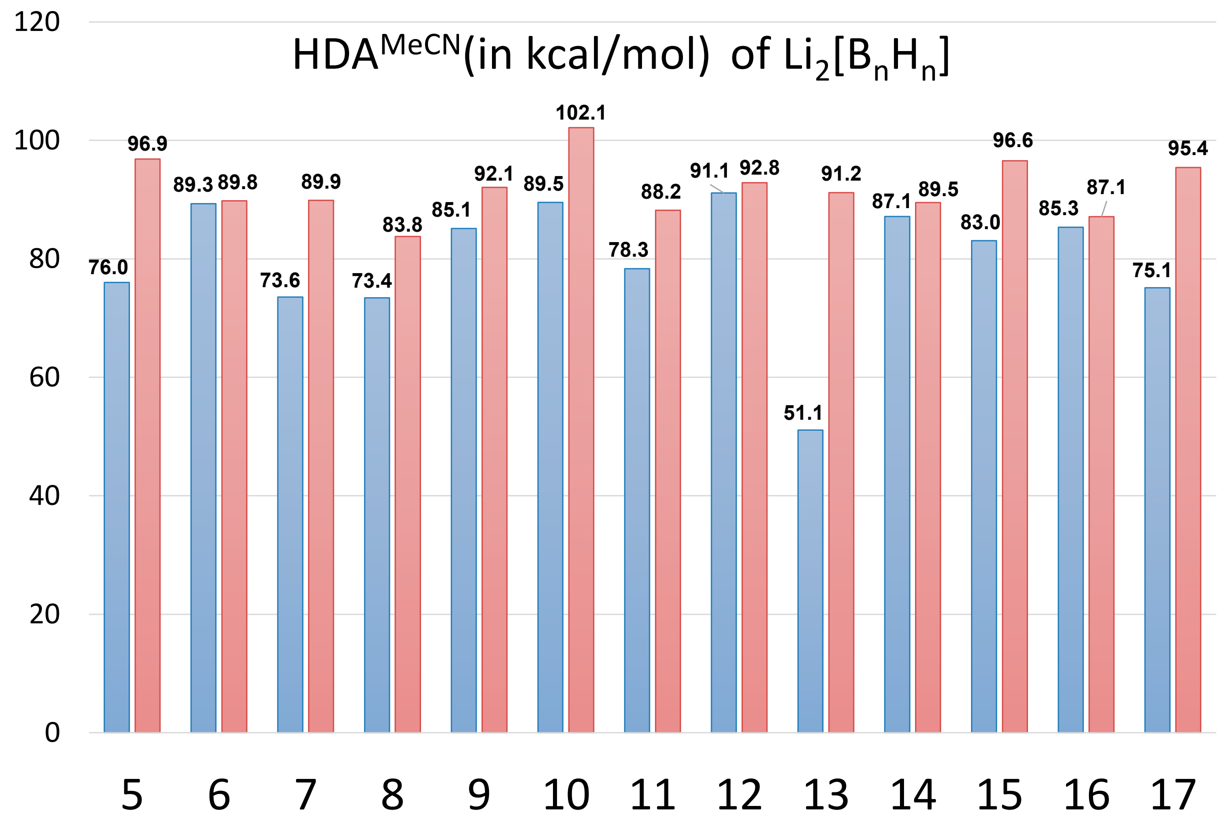

Figure S3. HDA^{MeCN} of polyhedral closo-boranes $Li_2[B_nH_n]$ (n = 5–17). Blue columns represent lowest HDA^{MeCN} values, red columns – highest.

$Li_2[B_2H_2] > Li_2[B_3H_3] > Li_2[B_4H_4]$

92.9 60.9 41.3

Scheme S9. General trend of HDA^{MeCN} for $Li_2[B_nH_n]$ (n = 2–4).

$Li_2[B_{12}H_{12}] > Li_2[B_{10}H_{10}] \sim Li_2[B_6H_6] > Li_2[B_{14}H_{14}] > Li_2[B_{16}H_{16}] \sim Li_2[B_9H_9] > Li_2[B_{15}H_{15}]$

91.1 89.8 89.3 87.1 85.5 85.1 83.0

$> Li_2[B_{11}H_{11}] > Li_2[B_5H_5] > Li_2[B_{17}H_{17}] > Li_2[B_7H_7] > Li_2[B_8H_8] > Li_2[B_{13}H_{13}]$

78.3 76.0 75.1 73.4 73.6 51.1

Scheme S10. General trend of HDA^{MeCN} for polyhedral closo-boranes $Li_2[B_nH_n]$ (n = 5–17).

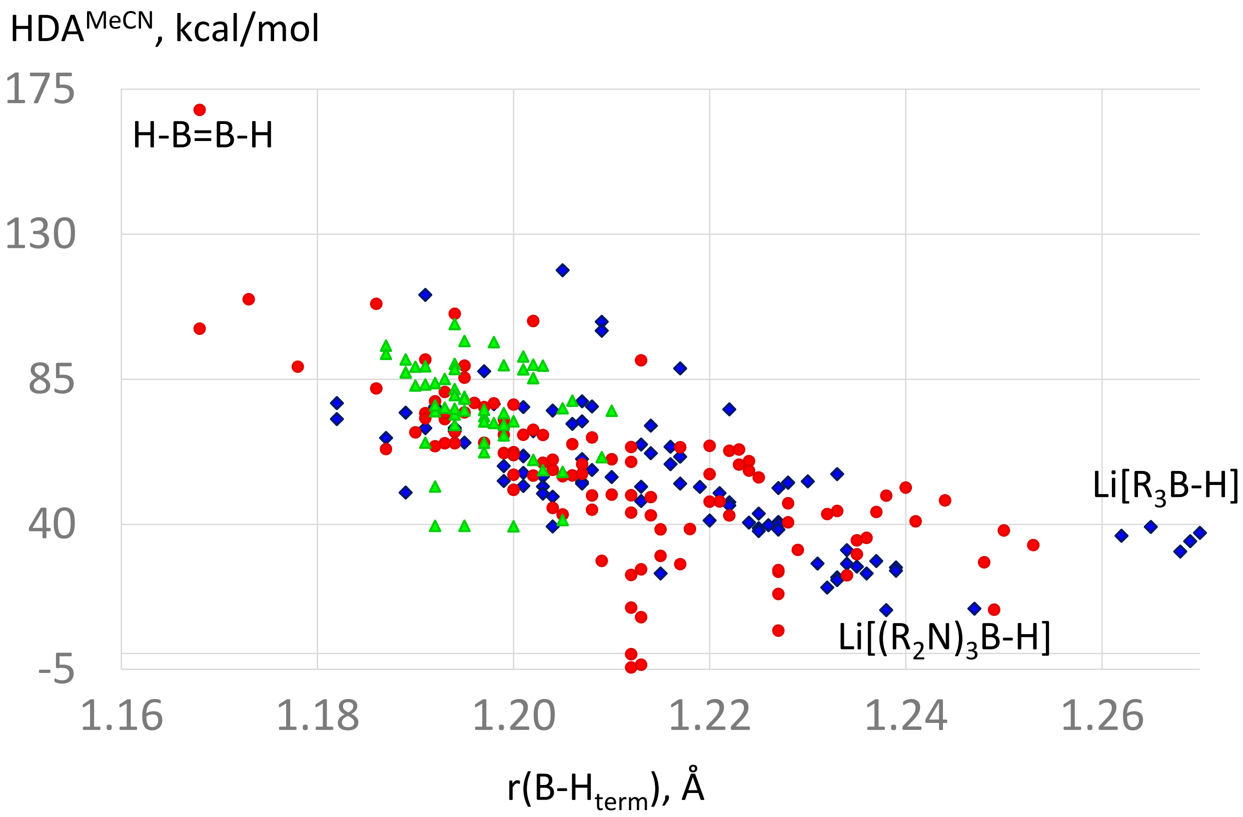

Figure S4. Plot HDA^{MeCN} vs bond length of terminal B–H bond for Li salts polyhedral closo-boranes (green triangles), neutral and anionic borane clusters (red circles) and tetracoordinated boron hydrides (blue squares). HDA^{MeCN} for tetracoordinated boron hydrides are taken from ref.[5]

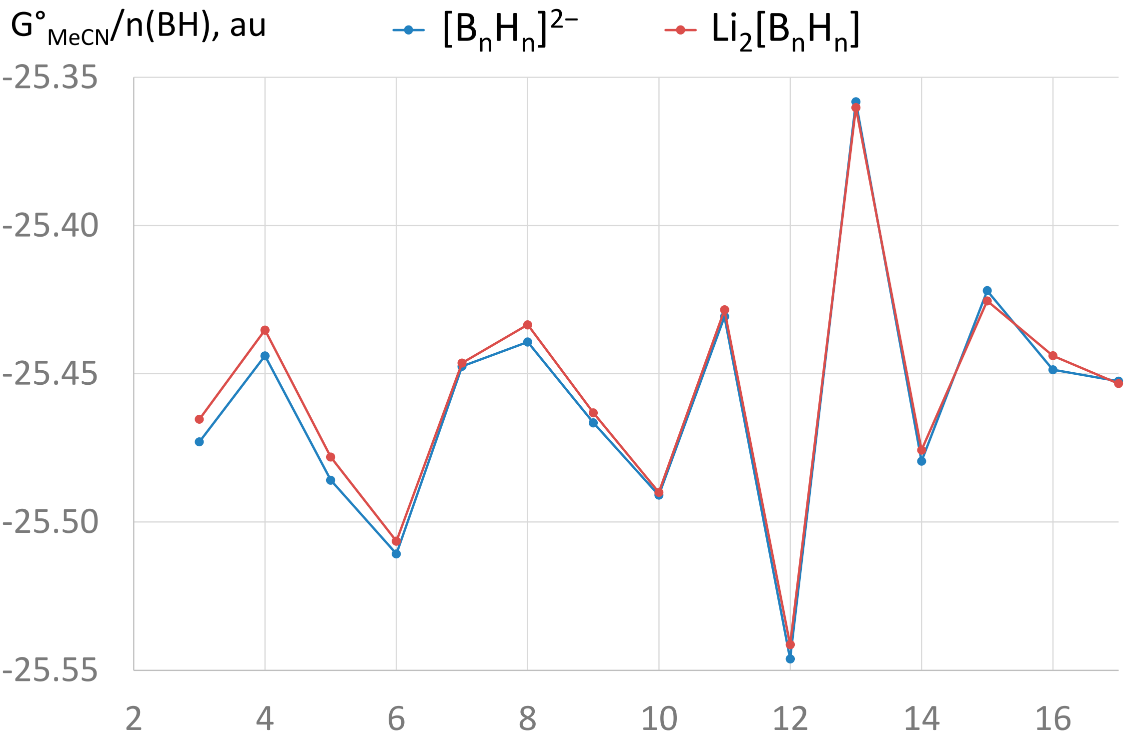

Figure S5. Free Gibbs energy per BH unit calculated for dianions $[B_nH_n]^{2-}$ (n = 5–17) of polyhedral closo-boranes and their Li-salts $Li_2[B_nH_n]$ in (n = 5–17) MeCN *vs* number boron atoms.

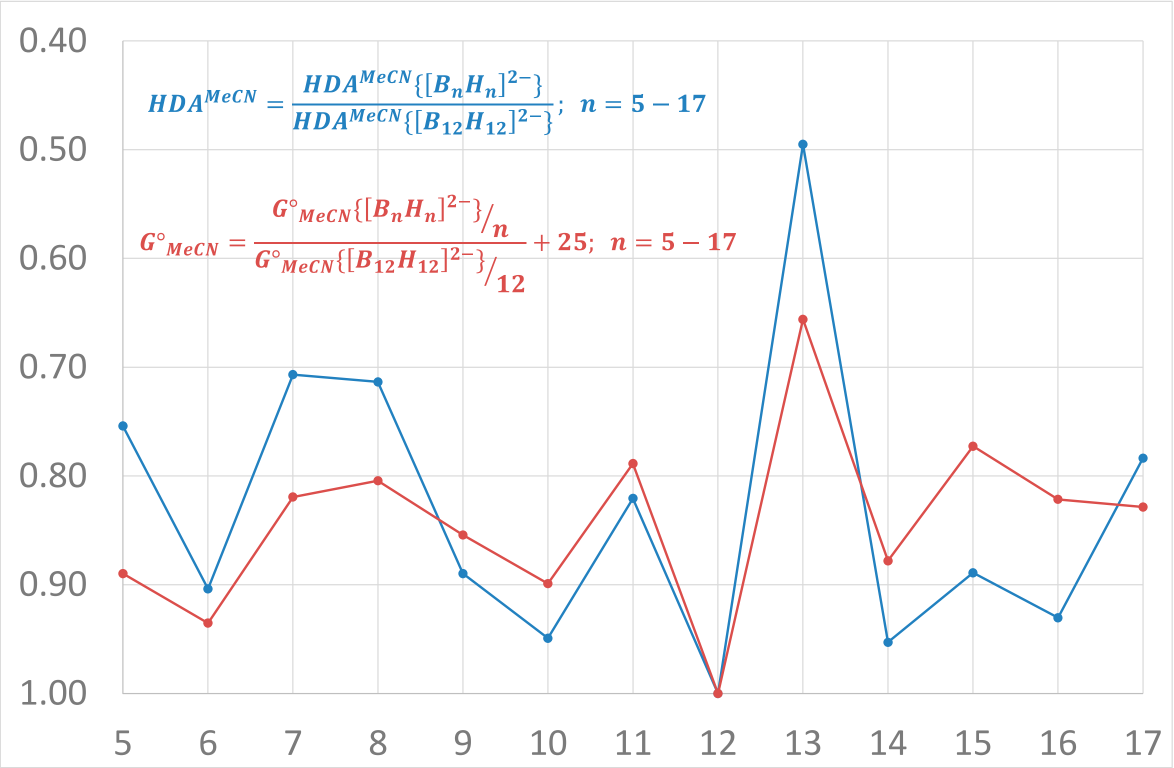

Figure S6. Graph of normalized lowest HDA^{MeCN} of and free Gibbs energy per BH unit for dianions $[B_nH_n]^{2-}$ (n = 5–17) of polyhedral closo-boranes vs number boron atoms.

Table S12. DFT-optimized geometries (Cartesian coordinates) and electronic energies.

LiBH$_4$_MeCN E = -34.8014599560 Ha

5	3.781549000	0.340343000	-0.381731000
1	3.350566000	1.339177000	0.195018000
1	2.993610000	0.071015000	-1.288982000
1	4.852053000	0.651056000	-0.904635000
1	3.914185000	-0.586514000	0.378708000
3	3.557240000	1.936114000	-1.694032000

LiBH$_3^+$_MeCN E = -34.0291019103 Ha

5	3.683581000	0.658581000	-0.359502000
1	3.611936000	0.138848000	0.695153000
1	2.835988000	0.461382000	-1.175646000
1	4.601070000	1.376664000	-0.612984000
3	3.804939000	1.686590000	-2.608552000

Li[B$_2$H$_7$]_MeCN E = -61.4282013960 Ha

5	4.071234000	1.657218000	19.959167000
5	4.933726000	3.673993000	20.277279000
1	4.023043000	2.805676000	20.614855000
1	5.173765000	1.194028000	19.760730000
1	3.524956000	1.042687000	20.841323000
1	3.414303000	1.786759000	18.962695000
1	4.348858000	4.643880000	20.674768000
1	5.154234000	3.738573000	19.088418000
1	5.924186000	3.424883000	20.933598000
3	6.553673000	2.420826000	19.376015000

{Li[B$_2$H$_6$]}$^+$_MeCN E = -60.6869426303 Ha

5	4.139542000	1.779815000	20.139773000
5	4.729112000	3.394245000	20.258572000
1	3.599002000	2.923717000	19.771675000
1	5.181834000	2.270615000	20.777645000
1	3.425243000	1.271895000	20.931349000
1	4.485390000	1.148080000	19.186346000
1	4.472638000	4.132450000	21.144001000
1	5.442437000	3.772186000	19.377544000
3	5.722593000	2.270636000	17.968345000

Li[B$_3$H$_{10}$]_MeCN E = -88.0414681055 Ha

5	4.221385000	1.433482000	19.678143000
5	4.701772000	3.378840000	20.539663000
1	4.043760000	2.291606000	20.697672000
1	5.378778000	1.315161000	19.343977000
1	3.875097000	0.500730000	20.352725000
1	3.468488000	1.746976000	18.801989000
1	4.804371000	3.828623000	19.435628000
1	5.756785000	3.246911000	21.114947000
3	6.535351000	2.870766000	19.257439000
1	4.087578000	4.049557000	21.417415000
5	3.054548000	4.778969000	20.874883000
1	3.544925000	5.796888000	20.472407000
1	2.551707000	4.856314000	21.966522000
1	2.410009000	4.155761000	20.079262000

{Li[B$_3$H$_9$]}$^+$_MeCN E = -87.2990993667 Ha

5	4.559742000	2.025562000	19.423394000
5	4.341237000	3.864709000	20.403316000
1	4.725815000	1.645190000	20.556936000
1	5.558781000	2.184532000	18.770401000
1	3.657321000	1.488806000	18.861536000
1	3.947195000	3.239325000	19.376733000
1	5.469381000	4.048371000	20.696422000
3	6.738948000	2.055004000	20.351646000
1	3.608816000	3.553875000	21.425882000
5	3.004167000	4.698569000	21.093741000
1	3.809744000	5.011204000	20.076246000
1	3.215711000	5.475546000	21.957837000
1	1.922598000	4.459160000	20.685858000

***n*-Li[B$_4$H$_{13}$]_MeCN E = -114.668663978 Ha**

5	4.084790000	1.658739000	19.580628000
5	4.532888000	3.537467000	20.562368000
1	4.630493000	2.231459000	20.614370000
1	5.009538000	1.256554000	18.941116000
1	3.238924000	2.201526000	18.953208000
1	4.695704000	4.055992000	19.511744000
1	5.374548000	3.722557000	21.392276000
3	1.296786000	2.480024000	20.114994000
1	3.468641000	3.662444000	21.251243000
5	2.536215000	4.473144000	20.719676000
1	2.893135000	5.602376000	20.868395000
1	1.730746000	4.088254000	21.538973000
1	2.247093000	4.180881000	19.588428000
1	3.806365000	0.566141000	20.204680000
5	2.558484000	0.558204000	20.701830000
1	1.752232000	0.457149000	19.810823000
1	2.755943000	-0.465435000	21.293120000
1	2.384277000	1.490759000	21.446373000

{*n*-Li[B$_4$H$_{12}$]}$^+$_MeCN E = -113.915310391 Ha

5	4.363605000	1.572465000	19.988148000
5	4.486441000	3.561478000	20.507604000
1	4.688571000	2.321407000	20.974106000
1	5.208028000	1.289834000	19.224356000
1	3.205028000	1.739991000	19.424687000
1	4.403891000	3.644871000	19.327071000
1	5.439028000	4.039965000	21.028340000
3	0.777120000	2.939467000	21.126105000
1	3.511686000	3.716293000	21.308812000
5	2.548761000	4.428825000	20.694751000
1	2.954746000	5.489685000	20.334477000
1	1.944897000	4.473805000	21.745000000
1	2.015139000	3.762363000	19.841381000
1	4.084029000	0.522696000	20.737603000
5	2.872477000	0.764585000	20.267569000
1	2.549824000	-0.161611000	19.612784000
1	2.191298000	1.194968000	21.140628000

***i*-Li[B$_4$H$_{13}$]_MeCN E = -114.656677363 Ha**

5	3.717801000	1.273600000	19.959447000
5	4.296564000	3.178510000	20.868921000
1	3.767921000	2.048442000	21.096326000
1	4.830764000	0.892385000	19.689270000
1	3.074278000	0.458960000	20.561555000
1	3.139980000	1.859988000	19.091816000
1	4.917283000	3.390620000	19.886224000
3	6.456242000	2.109615000	19.850131000
1	3.249163000	3.839174000	21.087854000
5	3.307675000	5.111195000	20.508308000
1	4.360328000	5.626680000	20.758122000
1	2.396927000	5.443655000	21.217726000
1	3.041900000	4.959857000	19.350882000
1	4.819281000	3.374711000	22.018542000
5	6.132498000	3.006788000	22.048047000
1	6.784368000	3.759095000	21.377062000
1	6.206420000	1.837911000	21.757607000
1	6.187089000	3.187451000	23.228568000

{*i*-Li[B$_4$H$_{12}$]}$^+$_MeCN E = -113.915310391 Ha

5	3.901732000	1.692061000	19.878975000
5	4.159054000	3.622320000	20.850404000
1	3.784461000	1.368136000	21.027617000
1	4.902157000	1.322284000	19.320789000
1	2.914393000	1.732541000	19.220213000
1	4.309193000	3.040930000	19.765921000
3	6.114826000	0.922005000	20.894970000
1	2.914219000	3.769754000	21.162280000
5	3.191081000	5.043263000	20.810884000
1	4.483235000	4.822407000	20.495758000
1	3.164098000	5.710542000	21.782119000
1	2.605236000	5.279326000	19.815823000
1	4.623655000	3.313395000	21.957427000
5	6.035716000	3.236757000	21.898974000
1	6.418370000	3.192135000	20.762306000
1	6.020293000	2.192261000	22.497465000
1	6.314001000	4.206135000	22.525212000

***n*-Li[B$_5$H$_{16}$]_MeCN E = -141.285709420 Ha**

5	3.957531000	1.742119000	19.470573000
5	4.541384000	3.439856000	20.650461000
1	4.248691000	2.178410000	20.682038000
1	4.945229000	1.604050000	18.818656000
1	3.039453000	2.274196000	18.940492000
1	4.695978000	3.992701000	19.617596000
1	5.452754000	3.443264000	21.419271000
3	1.165519000	2.171521000	20.352373000
1	3.599200000	3.812751000	21.458927000
5	2.762112000	4.619633000	20.834815000
1	3.219720000	5.716099000	20.744373000
1	2.242373000	4.179120000	19.863972000
1	3.837856000	0.528739000	19.878983000
5	2.597703000	0.223631000	20.327369000
1	2.940849000	-0.887410000	20.617309000
1	2.353259000	0.896524000	21.295564000
1	1.784657000	0.277141000	19.442301000
1	2.063547000	4.798502000	21.899153000
5	1.081069000	3.881654000	22.065177000
1	1.579181000	2.793520000	22.197312000
1	0.760306000	4.369095000	23.112181000
1	0.248341000	3.967461000	21.200796000

{*n*-Li[B$_5$H$_{15}$]}$^+$_MeCN E = -140.532206550 Ha

5	3.772427000	1.752151000	19.500853000
5	4.472595000	3.418218000	20.581882000
1	4.091495000	2.204577000	20.707534000
1	4.682971000	1.783365000	18.735950000
1	2.737626000	2.183458000	19.099016000
1	4.254722000	4.055845000	19.606537000
1	5.558485000	3.461564000	21.050914000
3	0.985085000	1.354566000	19.818820000
1	3.763964000	3.712279000	21.665160000
5	2.986056000	4.644021000	21.233838000
1	3.409296000	5.735286000	21.159803000
1	2.150954000	4.338415000	20.289575000
1	3.917575000	0.549036000	19.910033000
5	2.771505000	0.056948000	20.430523000
1	3.352195000	-0.857806000	20.934485000
1	2.262541000	0.800738000	21.233584000
1	2.069543000	-0.257201000	19.500413000
1	2.185809000	4.546091000	22.274493000
5	1.336911000	4.168926000	21.330623000
1	1.100461000	3.012776000	21.458802000
1	0.494190000	4.990231000	21.254671000

***i*-Li[B$_5$H$_{16}$]_MeCN E = -141.276126434 Ha**

5	3.244392000	1.124650000	20.080216000
5	4.478454000	2.864779000	20.638820000
1	4.479276000	1.614541000	20.546561000
1	3.802132000	0.122428000	19.725706000
1	2.554284000	1.034590000	21.052232000
1	2.848311000	1.831031000	19.197482000
3	7.225599000	4.895977000	20.073412000
1	3.527752000	3.456385000	21.013433000
1	5.232516000	3.091967000	21.670566000
5	6.427795000	2.510349000	21.612823000
1	6.985331000	2.589359000	20.568285000
1	6.325300000	1.420285000	22.080223000
1	5.027395000	3.088172000	19.528290000
5	4.999419000	4.398959000	19.184590000
1	3.996195000	4.555391000	18.561129000
1	5.999712000	4.237173000	18.520076000
1	5.145900000	5.133899000	20.123275000
1	6.863522000	3.124027000	22.644751000
5	7.448326000	4.316335000	22.361991000
1	6.610700000	5.066241000	21.929438000
1	8.431394000	4.184187000	21.681547000
1	7.671325000	4.498520000	23.523844000

{*i*-Li[B$_5$H$_{15}$]}$^+$_MeCN E = -140.516215454 Ha

5	3.345986000	1.626590000	20.516304000
5	4.745789000	2.622543000	20.440819000
1	4.642655000	1.337457000	20.300780000
1	2.757181000	1.438320000	19.513168000
1	3.045983000	1.196895000	21.571553000
3	7.012439000	5.401163000	20.262346000
1	3.520079000	2.961164000	20.641732000
1	5.266460000	2.917661000	21.528481000
5	6.574178000	2.361162000	21.648043000
1	7.142942000	2.425511000	20.615681000
1	6.367161000	1.312418000	22.162422000
1	5.240625000	2.869454000	19.321779000
5	5.091983000	4.233359000	19.148370000
1	4.221304000	4.262704000	18.340978000
1	6.220165000	4.326315000	18.738015000
1	4.869974000	4.795289000	20.189291000
1	6.812741000	3.092437000	22.641997000
5	7.331960000	4.348179000	22.369639000
1	6.365518000	5.009510000	22.088326000
1	8.229094000	4.341574000	21.572295000
1	7.672502000	4.448509000	23.509190000

***t*-Li[B$_5$H$_{16}$]_MeCN E = -141.273223117 Ha**

5	3.568838000	1.229430000	20.018707000
5	4.345922000	3.076720000	20.873542000
1	3.761112000	1.989836000	21.155972000
1	4.611870000	0.680214000	19.760631000
1	2.813365000	0.530200000	20.632478000
1	3.085538000	1.889020000	19.147378000
1	5.024049000	3.209032000	19.917348000
3	6.343884000	1.627501000	19.676597000
1	3.363049000	3.832385000	21.060348000
5	3.535798000	5.081760000	20.427599000
1	4.627098000	5.511608000	20.673022000
1	2.648268000	5.509705000	21.113323000
1	3.272567000	4.897543000	19.275052000
1	4.856729000	3.299097000	22.045036000
5	6.111422000	2.843271000	22.092795000
1	6.223891000	1.730921000	21.682512000
1	6.183908000	2.945851000	23.276220000
1	6.666082000	3.860980000	21.573014000
5	7.837712000	3.503020000	20.994005000
1	7.596222000	3.090861000	19.883991000
1	8.190262000	4.649430000	20.980532000
1	8.476485000	2.779367000	21.700266000

{*t*-Li[B$_5$H$_{15}$]}$^+$_MeCN E = -140.557420018 Ha

5	4.113447000	4.331566000	17.730951000
5	3.789221000	2.754896000	22.310213000
1	3.633904000	2.973648000	21.164849000
1	3.932524000	3.905473000	18.978210000
1	3.676940000	3.521266000	16.987767000
3	6.869412000	3.163958000	20.612855000

1	2.756526000	3.200502000	22.984523000
5	2.713713000	1.931560000	23.372258000
1	1.713721000	1.468591000	22.947140000
1	3.039748000	1.842613000	24.504697000
1	3.704790000	1.493832000	22.593602000
1	4.651096000	3.426413000	22.951602000
5	5.842461000	2.812625000	22.769211000
1	5.813157000	1.959652000	21.915948000
1	6.050811000	2.407613000	23.869426000
1	6.422863000	3.822075000	22.455913000
1	3.801836000	5.471018000	17.669369000
5	5.232446000	3.838798000	18.940612000
1	5.563339000	2.694046000	19.023108000
1	5.683562000	4.643623000	19.699260000
1	5.439863000	4.248905000	17.721826000

BH$_3$_MeCN E = -26.5923628927 Ha

5	3.937319000	0.172814000	-0.602166000
1	3.069478000	0.298380000	-1.399609000
1	4.997248000	0.666594000	-0.797230000
1	3.746429000	-0.447554000	0.390721000

BH$_2^+$_MeCN E = -25.7531978290 Ha

5	4.001351000	0.379263000	-0.932999000
1	2.900384000	0.169304000	-1.277624000
1	5.102309000	0.589219000	-0.588382000

B$_2$H$_6$_MeCN E = -53.2496516866 Ha

5	1.537959000	0.030344000	0.016623000
1	2.170386000	0.314479000	-0.945488000
1	0.681710000	-0.916429000	-0.300370000
1	-0.823693000	-0.245094000	0.895507000
1	-0.726471000	0.328391000	-1.084500000
1	0.665017000	0.985793000	0.250489000
5	-0.191227000	0.039041000	-0.066579000
1	2.073250000	-0.259003000	1.034519000

B$_2$H$_5^+$_MeCN E = -52.4511740928 Ha

5	1.256846000	0.140597000	-0.223046000
1	2.356942000	0.282769000	-0.556667000
1	0.748868000	-1.010157000	0.208849000
1	-1.258163000	-0.176907000	0.541634000
1	0.205418000	0.356827000	-1.009473000
1	0.692849000	0.812421000	0.778103000
5	-0.157974000	-0.035223000	0.208054000

***lin*-B$_3$H$_9$_MeCN E = -79.8564987956 Ha**

5	3.826379000	2.742534000	22.318350000
1	3.662286000	2.947716000	21.171806000
1	2.757473000	3.185902000	22.972622000
5	2.715787000	1.933589000	23.363570000
1	1.729622000	1.455288000	22.918835000
1	3.005596000	1.854029000	24.508266000
1	3.723144000	1.482802000	22.631493000
1	4.648308000	3.444166000	22.971279000
5	5.846296000	2.824468000	22.765989000
1	5.764284000	1.960917000	21.933848000
1	6.050321000	2.441867000	23.882001000
1	6.422980000	3.805314000	22.396574000

***lin*-B$_3$H$_8^+$_MeCN E = -79.0805617779 Ha**

5	4.010320000	2.504940000	22.718649000
1	4.288470000	2.694702000	21.494354000
1	3.055349000	3.259919000	23.084599000
5	2.526131000	2.018563000	23.361412000
1	1.651508000	1.829016000	22.600088000
1	2.480719000	1.899801000	24.529786000
1	3.623751000	1.310738000	22.915214000
1	5.070231000	2.750036000	23.375433000
5	5.492275000	3.013556000	22.088988000
1	6.265010000	2.179688000	21.792887000
1	5.638392000	4.175765000	21.991222000

***btf*-B$_3$H$_9$_MeCN E = -79.8562848596 Ha**

5	3.679582000	3.137948000	21.132483000
1	3.889777000	1.999048000	20.906882000
1	2.405488000	3.341902000	20.361622000
5	1.998169000	3.622783000	21.504781000
1	1.190626000	2.804215000	21.808493000
1	1.775776000	4.786569000	21.619108000
1	2.995937000	3.351351000	22.271474000
1	4.861131000	3.467174000	22.009998000
1	4.168986000	3.980619000	20.204481000
5	5.066307000	4.269417000	21.080190000
1	4.870804000	5.393920000	21.418651000
1	6.080685000	3.950238000	20.547440000

***btf*-B$_3$H$_8^+$_MeCN E = -79.0805381951 Ha**

5	3.799397000	3.754632000	21.187222000
1	3.276869000	2.613117000	20.988461000
1	1.546468000	3.470101000	20.261967000
5	2.157389000	3.367920000	21.260425000
1	1.744196000	3.026941000	22.306415000
1	2.843384000	4.549255000	21.452106000
1	4.634702000	3.706616000	22.144831000
1	4.443086000	4.140497000	20.161090000
5	5.441798000	4.142351000	21.113729000
1	5.724412000	5.262422000	21.328944000
1	6.180942000	3.267116000	20.851920000

***cyc*-B$_3$H$_9$_MeCN E = -79.8552502295 Ha**

1	-1.062478000	5.525448000	5.008743000
1	0.494385000	6.958134000	5.002293000
5	0.092426000	5.740545000	5.147956000
1	-0.942433000	8.045246000	5.891316000
5	0.062765000	7.456555000	6.131997000
1	-0.457745000	6.798142000	7.127145000
5	0.265437000	5.715945000	7.117669000
1	1.289982000	5.837326000	7.704725000
1	1.030395000	8.072142000	6.438029000
1	0.840003000	5.350900000	4.304174000
1	-0.592632000	5.041375000	7.590524000
1	0.717783000	5.083107000	6.065479000

***cyc*-B$_3$H$_8^+$_MeCN E = -79.0724553563 Ha**

1	-0.977457000	6.054996000	5.168186000
1	0.748350000	7.021658000	5.264942000
5	0.318025000	5.772139000	5.175840000
1	-0.977451000	8.033924000	5.720702000
5	-0.422010000	7.019112000	5.888055000
1	-0.397122000	6.833256000	7.150439000
5	0.286260000	5.662685000	7.169802000
1	1.300016000	5.965003000	7.687896000
1	0.643651000	5.297685000	4.158169000
1	-0.517570000	4.929799000	7.623176000
1	0.702802000	4.962467000	6.084815000

***lin*-B$_3$H$_9$_MP2_MeCN E = -79.2160680965 Ha**

5	3.842541000	2.728084000	22.357993000
1	3.711036000	2.977076000	21.212588000
1	2.790682000	3.188916000	23.017628000
5	2.653491000	1.921142000	23.347197000
1	1.669628000	1.515594000	22.822819000
1	2.888525000	1.781062000	24.501184000
1	3.682530000	1.469535000	22.643166000
1	4.673557000	3.331611000	23.092612000
5	5.881604000	2.848018000	22.740417000
1	5.790190000	1.900224000	22.005621000
1	6.226439000	2.585416000	23.859648000
1	6.342252000	3.831914000	22.233759000

***lin*-B$_3$H$_8^+$_MP2_MeCN E = -78.4882868564 Ha**

5	4.011138000	2.504987000	22.720680000
1	4.286883000	2.697604000	21.498137000
1	3.056491000	3.257335000	23.086554000
5	2.511985000	2.013390000	23.367422000
1	1.643942000	1.826185000	22.591121000
1	2.473975000	1.895766000	24.540588000
1	3.625640000	1.312890000	22.918174000
1	5.069912000	2.753015000	23.373311000
5	5.505917000	3.018632000	22.081514000
1	6.271233000	2.170569000	21.788284000
1	5.645041000	4.186353000	21.986848000

***btf*-B$_3$H$_9$_MP2_MeCN E = -79.2055049386 Ha**

5	3.678340000	3.149679000	21.135282000
1	3.888203000	2.005681000	20.908538000
1	2.435527000	3.396302000	20.384719000
5	1.984009000	3.615843000	21.530680000
1	1.190942000	2.758123000	21.762490000
1	1.736360000	4.771555000	21.678778000
1	2.973309000	3.332628000	22.288808000
1	4.811752000	3.510449000	22.005079000
1	4.196808000	3.976795000	20.181450000
5	5.080120000	4.278023000	21.054205000
1	4.907703000	5.415983000	21.361316000
1	6.100195000	3.894124000	20.574258000

***btf*-B$_3$H$_8^+$_MP2_MeCN E = -78.4882573253 Ha**

5	3.799510000	3.753891000	21.185370000
1	3.275862000	2.615357000	20.986732000
1	1.535643000	3.470476000	20.255374000
5	2.141542000	3.364572000	21.261720000
1	1.739758000	3.024455000	22.317323000
1	2.845210000	4.547330000	21.449128000
1	4.632959000	3.705172000	22.141539000
1	4.444206000	4.138569000	20.162337000
5	5.457755000	4.146339000	21.114056000
1	5.728200000	5.273431000	21.332425000
1	6.191998000	3.261377000	20.851105000

***cyc*-B$_3$H$_9$_MP2_MeCN E = -79.2060007087 Ha**

1	-1.060786000	5.510346000	4.968243000
1	0.464322000	6.957315000	5.010597000
5	0.093265000	5.728516000	5.124106000
1	-0.955198000	8.057768000	5.900741000
5	0.059652000	7.479162000	6.133627000
1	-0.426779000	6.801038000	7.127486000
5	0.268485000	5.705272000	7.138439000
1	1.306730000	5.824381000	7.704695000
1	1.036660000	8.088304000	6.429159000
1	0.877957000	5.354561000	4.304219000
1	-0.598650000	5.043169000	7.616981000
1	0.672231000	5.075033000	6.071756000

***cyc*-B$_3$H$_8^+$_MP2_MeCN E = -78.4715043739 Ha**

1	-0.973753000	6.055804000	5.170755000
1	0.750035000	7.014697000	5.278819000
5	0.319526000	5.769707000	5.159004000
1	-0.982698000	8.053118000	5.705586000
5	-0.429914000	7.032479000	5.879995000
1	-0.398360000	6.814046000	7.136299000
5	0.292867000	5.652052000	7.194757000
1	1.314342000	5.968227000	7.697169000
1	0.648001000	5.300558000	4.134260000
1	-0.515601000	4.917453000	7.646467000
1	0.683049000	4.974581000	6.088911000

***lin*-B$_4$H$_{12}$_MeCN E = -106.468663139 Ha**

5	4.208856000	2.733282000	22.093432000
1	3.759273000	3.344219000	21.196951000
1	2.400965000	3.119466000	23.261193000
5	2.701049000	1.961874000	23.261797000
1	1.884549000	1.210076000	22.850345000
1	3.651931000	1.622137000	22.395292000
1	4.650809000	3.483147000	23.050940000
5	5.788653000	2.840588000	22.768829000
1	5.336850000	2.171655000	21.718176000
1	6.046218000	2.093412000	23.647692000
1	6.543329000	3.655684000	22.367386000
1	3.260211000	1.859796000	24.384111000
5	3.373298000	0.544803000	24.785312000
1	3.845841000	-0.143765000	23.925642000
1	4.157247000	0.894089000	25.629552000
1	2.297805000	0.213559000	25.195765000

***lin*-B$_4$H$_{11}^+$_MeCN E = -105.709329548 Ha**

5	4.095701000	2.951094000	22.888474000
1	4.079149000	2.913136000	21.607507000
1	3.332060000	3.709182000	23.355582000
5	2.969287000	1.372821000	23.462077000
1	2.173001000	1.571835000	22.623900000
1	4.216369000	1.728499000	23.324470000
1	5.314945000	3.260528000	23.155643000
5	5.402699000	3.148726000	21.802161000
1	6.023670000	2.190145000	21.516448000
1	5.615325000	4.233253000	21.393428000
1	2.579913000	1.695717000	24.639767000
5	2.941764000	0.409240000	24.876528000
1	3.250465000	0.122793000	23.582489000
1	3.915318000	0.384627000	25.538275000
1	1.951000000	-0.180987000	25.117976000

***pinc*-B$_4$H$_{12}$_MeCN E = -106.458471004 Ha**

5	4.111081000	2.396374000	22.419381000
1	2.531727000	3.081192000	23.784609000
5	2.876678000	1.976328000	24.100934000
1	3.580176000	1.896587000	25.062663000
1	3.779850000	1.453173000	23.154590000
1	4.638940000	3.423512000	23.033472000
5	5.747258000	2.923251000	22.504435000
1	5.192147000	1.895782000	21.878185000
1	6.411877000	2.504162000	23.386165000
1	6.135295000	3.679061000	21.684605000
1	2.074926000	1.113045000	23.900873000
1	3.368659000	2.990862000	21.621791000
5	2.930392000	2.031029000	20.688950000
1	1.769629000	2.184022000	20.926559000
1	3.397142000	2.570170000	19.730878000
1	3.374168000	0.946761000	20.948272000

***pinc*-B$_4$H$_{11}^+$_MeCN E = -105.709302817 Ha**

5	4.696122000	2.759413000	21.850369000
1	1.852175000	2.890763000	24.201901000
5	1.808654000	1.953775000	23.490139000
1	2.897353000	1.166432000	23.692414000
1	4.124492000	2.406490000	22.967907000
1	5.279547000	3.785006000	22.360454000
5	6.353403000	2.959553000	22.226381000
1	5.664419000	1.946115000	21.642453000
1	6.729601000	2.620876000	23.289159000
1	7.036545000	3.433229000	21.391246000
1	0.886742000	1.226123000	23.393901000
1	4.084383000	3.027923000	20.886003000
5	3.179267000	1.620196000	22.520801000
1	2.147992000	2.324346000	22.230516000
1	3.409621000	0.761049000	21.756159000

***cyc*-B$_4$H$_{12}$_MeCN E = -106.453454792 Ha**

1	-0.973711000	4.507024000	5.200208000
1	0.646482000	5.512012000	4.187835000
5	0.184062000	4.688342000	5.088931000
1	-0.948122000	8.194248000	6.015689000
5	0.031690000	7.533007000	6.122286000
1	-0.572308000	6.501208000	6.640827000
5	0.264092000	5.578176000	7.023844000
1	1.215883000	5.879138000	7.664406000
1	0.947906000	8.022129000	6.695100000
1	0.842492000	3.764181000	4.719294000
1	-0.551855000	4.901315000	7.557710000
1	0.957221000	5.012305000	6.079526000
1	0.709144000	7.302516000	5.036194000

5	-0.015590000	6.637928000	4.188510000
1	-1.192458000	6.637922000	4.189866000
1	0.524547000	7.062326000	3.212598000

cyc-$B_4H_{11}^+$_MeCN E = -105.684419501 Ha

1	-0.721096000	5.496728000	4.858110000
1	1.189159000	5.528295000	4.317076000
5	0.395526000	4.865332000	5.149274000
1	-1.175600000	7.326424000	5.961156000
5	-0.004499000	7.429739000	6.083782000
1	0.699099000	6.799834000	6.977459000
5	0.287032000	5.567028000	7.029266000
1	0.943715000	5.279365000	7.974479000
1	0.439927000	8.499979000	6.338085000
1	0.265150000	3.754530000	4.804537000
1	-0.847189000	5.236827000	7.015353000
1	1.112135000	4.938766000	6.218072000
1	0.727383000	7.342392000	4.993484000
5	0.154988000	6.357912000	4.390686000
1	-0.203796000	6.672707000	3.322139000

lin-B_5H_{15}_MeCN E = -133.082511176 Ha

5	4.127825000	2.781482000	22.016076000
1	3.616701000	3.433490000	21.184763000
1	2.428328000	3.147170000	23.363488000
5	2.717164000	1.993338000	23.270774000
1	1.861193000	1.242757000	22.948637000
1	3.565042000	1.665383000	22.315290000
1	4.661857000	3.482817000	22.959239000
5	5.758327000	2.821245000	22.565305000
1	5.199612000	2.201130000	21.532389000
1	6.060131000	2.035563000	23.395371000
1	6.500148000	3.630785000	22.131624000
1	3.371464000	1.837570000	24.343352000
5	3.502246000	0.505609000	24.664123000
1	3.885835000	-0.146286000	23.751665000
1	2.513776000	0.198959000	25.245718000
1	4.366065000	1.015313000	25.449132000
5	5.240612000	0.056645000	25.899523000
1	6.173065000	0.138816000	25.149770000
1	5.370460000	0.611476000	26.958252000
1	4.709159000	-1.016722000	25.961739000

lin-$B_5H_{14}^+$_MeCN E = -132.332277713 Ha

5	4.655282000	2.228400000	22.578110000
1	4.320692000	1.253383000	22.018458000
1	2.493278000	2.520517000	23.332068000
5	3.310279000	2.180535000	24.117783000
1	4.576801000	2.376602000	23.867528000
1	4.304526000	3.315640000	21.982319000
5	5.633276000	3.522578000	22.018985000
1	5.947694000	2.338374000	22.578516000
1	5.874638000	4.398454000	22.770287000
1	6.019085000	3.483466000	20.904913000
1	3.326544000	2.692013000	25.187108000
5	3.444330000	0.423556000	25.177971000
1	3.114277000	0.900932000	24.011471000
1	2.568407000	0.356576000	25.953654000
1	4.565078000	0.761770000	25.709337000
5	4.960400000	-0.377556000	25.104984000
1	5.637743000	-0.116019000	24.173265000
1	5.277607000	-1.114815000	25.968590000
1	3.737879000	-0.750622000	24.702247000

iso-B_5H_{15}_MeCN E = -133.074163066 Ha

5	4.238723000	2.219233000	22.370068000
1	2.810108000	3.083256000	24.239277000
5	2.897891000	1.893134000	24.158530000
1	3.389412000	1.343036000	25.103629000
1	4.035332000	1.524003000	23.380546000
1	4.557605000	3.410803000	22.858627000
5	5.830392000	3.493017000	22.700901000
1	5.092800000	1.643709000	21.782886000
1	6.247414000	3.829095000	21.657456000
1	2.006637000	1.321784000	23.603329000
1	3.267897000	2.758229000	21.814492000
5	2.890785000	2.004825000	20.674920000
1	1.816984000	2.534369000	20.756677000
1	3.609042000	2.375124000	19.794709000
1	2.909389000	0.839326000	20.946626000
1	6.537543000	2.578198000	23.272632000
5	6.558007000	3.491771000	24.257657000
1	5.955144000	4.462768000	23.570129000
1	5.870449000	3.131103000	25.147857000
1	7.686656000	3.811545000	24.386546000

iso-$B_5H_{14}^+$_MeCN E = -132.305697001 Ha

5	3.984333000	2.122483000	23.073910000
5	2.506414000	2.009828000	23.927821000
1	2.415149000	2.565239000	24.960962000
1	3.626575000	1.232436000	23.927726000
1	4.857913000	2.672345000	23.867281000
5	5.532070000	3.439366000	23.044173000
1	4.330176000	1.622776000	22.058601000
1	4.995801000	4.092942000	22.231072000
1	1.654914000	1.373124000	23.420150000
1	2.959673000	2.883596000	22.987263000
5	3.382605000	1.904175000	20.253390000
1	2.439945000	1.258909000	20.576575000
1	3.336472000	3.089385000	20.314562000
1	4.345318000	1.371743000	19.807496000
1	6.513764000	2.750696000	22.594707000
5	7.143267000	3.387438000	23.616174000
1	6.061001000	4.137557000	23.981367000
1	7.400650000	2.616678000	24.467494000
1	7.912062000	4.134355000	23.127493000

pinc-B_5H_{15}_MeCN E = -133.068649074 Ha

5	4.237581000	2.529458000	22.068732000
1	2.404639000	3.151561000	23.154820000
5	2.642940000	1.987386000	23.280807000
1	1.816044000	1.225706000	22.912020000
1	3.572520000	1.515216000	22.411924000
1	4.696798000	3.306466000	23.003587000
5	5.825509000	2.645426000	22.717123000
1	5.331210000	1.916048000	21.716085000
1	6.073655000	1.926974000	23.620243000
1	6.579896000	3.423651000	22.253266000
1	3.214880000	1.952507000	24.394561000
5	3.288433000	0.659720000	24.904079000
1	3.741152000	-0.099368000	24.095633000
1	4.081261000	1.063480000	25.713569000
1	2.202127000	0.398614000	25.333046000
1	3.855243000	3.364777000	21.245873000
5	3.393804000	2.656371000	20.077443000
1	3.777217000	1.521417000	20.115944000
1	2.226200000	2.830857000	20.250998000
1	3.986405000	3.387860000	19.344662000

pinc-$B_5H_{14}^+$_MeCN E = -132.308414181 Ha

5	4.326378000	3.143771000	23.231852000
1	4.290252000	3.691481000	24.266708000
5	2.797416000	1.888176000	23.643911000
1	2.040715000	2.775336000	23.511730000
1	3.986489000	1.891327000	23.103464000
1	5.540213000	2.924245000	22.836217000
5	5.061836000	3.574980000	21.752559000
1	5.002909000	2.827591000	20.826386000
1	5.638496000	4.596994000	21.670576000
1	2.950417000	1.513321000	24.858748000
5	2.646428000	0.313967000	24.292313000
1	2.405775000	0.820166000	23.048633000
1	3.601724000	-0.373922000	24.269798000
1	1.597118000	-0.003599000	24.723565000
1	3.839839000	3.834343000	22.257236000
5	3.811841000	1.534412000	20.189930000
1	4.236856000	0.697975000	20.920521000
1	2.803890000	2.098064000	20.473180000
1	4.295267000	1.688525000	19.116844000

cyc-B_5H_{15}_MeCN E = -133.069556321 Ha

1	-0.914044000	4.500811000	5.133692000
1	1.068881000	4.523243000	4.240131000
5	0.239158000	4.288722000	5.228961000
1	-0.982449000	8.134669000	6.106669000
5	0.029298000	7.513675000	6.156630000
1	-0.507254000	6.413110000	6.605289000
5	0.369562000	5.511532000	6.956723000
1	1.320471000	5.826291000	7.590868000
1	0.934415000	8.007544000	6.742388000
1	0.602175000	3.183587000	5.474494000
1	-0.409720000	4.781977000	7.478328000
1	1.020184000	5.086273000	5.929567000
5	0.540500000	5.208603000	3.271774000
1	-0.497273000	4.891074000	2.812362000
1	1.508040000	5.073817000	2.579620000
1	0.677529000	7.309356000	5.059591000
5	-0.177939000	7.111456000	4.079360000
1	-1.224757000	6.611548000	4.272113000
1	0.811643000	6.453232000	3.517120000
1	-0.124699000	8.100891000	3.422656000

cyc-$B_5H_{14}^+$_MeCN E = -132.300360632 Ha

1	-0.649268000	4.758231000	4.791995000
1	1.250825000	4.590996000	4.167948000
5	0.523794000	4.388530000	5.272783000
1	-0.842053000	8.239001000	6.277406000
5	0.104050000	7.533624000	6.177482000
1	-0.500013000	6.435278000	6.544191000
5	0.317333000	5.498997000	6.921678000
1	1.077796000	5.664750000	7.804906000
1	1.126307000	7.863319000	6.672861000
1	0.570507000	3.245396000	5.511177000
1	-0.537419000	4.677758000	7.065015000
1	1.207711000	5.239612000	5.982291000
5	0.108220000	5.107293000	3.775960000
1	-0.289868000	4.556602000	2.821455000
1	0.633433000	7.389658000	4.991935000
5	-0.303506000	7.174443000	4.126373000
1	-1.398593000	6.796997000	4.353080000
1	0.504043000	6.285706000	3.511010000
1	-0.127618000	8.011404000	3.309170000

Li_2BH_3_MeCN E = -41.6940037919 Ha

5	3.194943000	6.307194000	4.721672000
1	2.287602000	6.889678000	5.301471000
1	3.184171000	5.105361000	5.013980000
1	4.289547000	6.753301000	5.084845000
3	1.809630000	7.189535000	3.222969000
3	4.711104000	5.333211000	3.751665000

$\{Li_2BH_2\}^+$_MeCN E = -40.9539413983 Ha

5	2.999110000	6.062162000	4.773787000
1	2.054887000	6.681549000	5.231070000
1	3.436798000	5.372684000	5.662166000
3	2.191829000	7.333852000	3.135242000
3	4.504826000	5.374733000	3.209492000

$Li_2[B_2H_6]$_MeCN E = -68.4069532578 Ha

5	3.239848000	6.300474000	2.792059000
1	3.268493000	7.467042000	2.371972000
1	4.174520000	5.705560000	2.255042000
1	2.194051000	5.809024000	2.340294000
5	3.230624000	6.271512000	4.579291000
1	2.296240000	6.866508000	5.116541000
1	3.201773000	5.104846000	4.999238000
1	4.276659000	6.762601000	5.030913000
3	1.579874000	7.369738000	3.415592000
3	4.891826000	5.203076000	3.955028000

$\{Li_2[B_2H_5]\}^+$_MeCN E = -67.6748063751 Ha

5	3.308317000	6.330242000	2.752208000
1	2.793171000	7.167961000	2.053714000
1	4.227506000	5.755176000	2.223583000
1	2.366246000	5.411813000	3.080811000
5	2.790816000	5.982551000	4.236034000
1	1.850252000	6.533739000	4.751755000
1	3.286390000	5.123194000	4.922446000
3	2.395985000	8.205719000	3.742243000
3	5.058565000	5.587385000	4.062262000

lin-$Li_2[B_3H_9]$_MeCN E = -95.0398557864 Ha

5	0.344445000	-0.291635000	1.251990000
1	1.539925000	-0.335367000	1.552304000
1	-0.308827000	-0.351707000	2.267364000
1	0.479782000	1.067853000	1.095287000
3	-1.133229000	0.290311000	-1.191279000
5	-0.144278000	-1.329567000	-0.065091000
1	-1.350521000	-1.370001000	-0.273689000
1	0.229062000	-2.478046000	0.165689000
1	0.411071000	-1.018424000	-1.138957000
3	1.956510000	-1.506857000	0.097795000
5	-0.358151000	1.735004000	0.396975000
1	0.076680000	1.748761000	-0.741088000
1	-0.243484000	2.811208000	0.928377000
1	-1.498294000	1.327540000	0.460451000

$\{$_lin_-$Li_2[B_3H_8]\}^+$_MeCN E = -94.3304396877 Ha

5	0.379912000	-0.329946000	1.437903000
1	1.519815000	-0.677778000	1.610188000
1	-0.302693000	-0.293836000	2.413919000
1	0.560899000	1.115662000	1.306280000
3	-2.039760000	0.719691000	-0.877282000
5	-0.263426000	-0.842736000	-0.147417000
1	-1.428707000	-0.982498000	-0.426438000
1	-0.190072000	-1.555499000	0.880377000
1	0.458328000	-1.391272000	-0.947216000
3	2.095259000	-0.502194000	-0.491033000
5	0.217789000	0.872031000	0.126404000
1	1.179883000	1.181711000	-0.537480000
1	-0.688243000	1.658196000	-0.002528000

cyc-$Li_2[B_3H_9]$_MeCN E = -95.0005677922 Ha

1	-0.667841000	5.401203000	4.494660000
1	0.106466000	7.146784000	4.169619000
5	0.229189000	6.134883000	4.850848000
1	-1.107868000	7.779478000	6.103953000
5	-0.033836000	7.349586000	6.512711000
1	-0.096185000	7.394504000	7.714841000
5	0.247773000	5.444154000	6.765551000
1	1.211169000	5.533365000	7.525841000
1	0.894081000	8.087270000	6.196709000
1	1.332802000	5.706826000	4.587796000
1	-0.757661000	5.233599000	7.442345000
1	0.433182000	4.393083000	6.163427000
3	-0.168967000	8.750697000	4.829835000
3	0.450414000	3.761491000	7.978410000

$\{$_cyc_-$Li_2[B_3H_8]\}^+$_MeCN E = -94.3227817426 Ha

1	-0.917797000	5.341497000	4.870533000
1	-0.688674000	7.254714000	4.918653000
5	-0.103396000	6.194968000	5.097256000
5	0.214542000	7.162647000	6.633737000
1	-0.724384000	7.658996000	7.172011000
5	0.333473000	5.472441000	6.727764000
1	0.784150000	6.351062000	7.570241000
1	1.132638000	7.901399000	6.385036000
1	0.870412000	6.107264000	4.370862000

1	-0.584311000	4.923590000	7.287935000
1	1.300668000	4.796357000	6.487868000
3	1.203233000	7.944873000	4.383329000
3	0.360033000	3.227637000	7.327369000

1,2-**$Li_2[B_4H_{12}]$_MeCN E = -121.668798779 Ha**

5	0.364945000	-0.251558000	1.405122000
1	1.567085000	-0.243885000	1.565015000
1	-0.187140000	-0.405923000	2.479361000
1	0.153381000	1.074820000	1.427558000
3	-1.892414000	0.048669000	1.283450000
5	-0.289848000	-1.290076000	0.161619000
1	-1.509131000	-1.357335000	0.104526000
1	0.087672000	-1.153103000	-0.986958000
3	1.750786000	-0.507512000	-0.371843000
5	-0.093188000	1.604899000	0.264351000
1	0.724986000	1.346159000	-0.586270000
1	0.020754000	2.735478000	0.672718000
1	-1.229600000	1.361403000	-0.088017000
1	-0.210473000	-2.572871000	0.433901000
5	1.003383000	-3.020779000	0.689784000
1	1.916253000	-2.488711000	0.099356000
1	1.120542000	-2.987396000	1.887750000
1	0.794157000	-4.125339000	0.246333000

{*1,2*-$Li_2[B_4H_{11}]$}$^+$_MeCN E = -120.946310331 Ha

5	0.781056000	-0.527440000	0.933558000
1	1.892945000	-0.159169000	0.676012000
1	0.928717000	-0.983844000	2.077491000
1	0.001317000	0.376457000	1.387063000
3	-1.563005000	0.476529000	-1.210678000
5	-0.004809000	-1.609154000	-0.238175000
1	-1.130247000	-1.267542000	-0.502501000
1	0.692780000	-1.822605000	-1.201559000
3	1.973071000	-0.407709000	-1.204175000
5	-0.208912000	1.358304000	0.498569000
1	0.232584000	1.236422000	-0.621644000
1	0.272817000	2.276063000	1.090434000
1	-1.418337000	1.310655000	0.544464000
1	-0.175651000	-2.772396000	0.200547000
5	0.315007000	-2.193995000	1.417039000
1	-0.636209000	-2.305275000	2.124475000
1	1.243718000	-2.917514000	1.606373000

1,1-**$Li_2[B_4H_{12}]$_MeCN E = -121.661148790 Ha**

5	3.984956000	2.248559000	22.213019000
1	3.725041000	3.158030000	21.428695000
1	2.317838000	3.043583000	23.955186000
5	2.726147000	2.041116000	23.385551000
1	4.215820000	1.260381000	21.531948000
1	5.028715000	2.588296000	22.795181000
1	2.941766000	1.474205000	24.570532000
5	3.629929000	0.364765000	24.579076000
1	3.523496000	-0.320624000	23.590270000
1	4.767306000	0.717513000	24.815304000
1	3.094398000	-0.126032000	25.537489000
3	3.572269000	4.212462000	22.996898000
3	5.260901000	0.559661000	22.938080000
1	1.654973000	1.336862000	23.255009000
5	1.085980000	1.431955000	22.057038000
1	1.105922000	2.552622000	21.624046000
1	0.004069000	1.108555000	22.488146000
1	1.607301000	0.600135000	21.361534000

{*1,1*-$Li_2[B_4H_{11}]$}$^+$_MeCN E = -120.946307002 Ha

5	3.224374000	1.982452000	22.004635000
1	3.798543000	3.034627000	21.850561000
1	2.418050000	2.855036000	24.272961000
5	2.370436000	1.890675000	23.561096000
1	3.904553000	1.009960000	21.795182000
1	2.524500000	0.770789000	24.155572000
5	3.733929000	0.660273000	24.725356000
1	4.020174000	-0.395796000	24.205877000
1	4.551497000	1.528758000	24.523574000
1	3.413455000	0.579566000	25.872306000
3	3.916370000	3.797592000	23.596395000
3	5.297096000	0.454115000	22.984320000
1	1.146450000	1.695844000	23.492803000
5	1.452128000	1.763370000	22.021517000
1	0.752332000	2.701917000	21.795168000
1	1.055735000	0.682487000	21.717552000
1	2.451385000	1.979998000	21.016178000

lin-**$Li_2[B_4H_{12}]$_MeCN E = -121.660350782 Ha**

5	4.431678000	2.648978000	22.087925000
1	3.709032000	3.602105000	22.195699000
1	1.760910000	2.084687000	22.450116000
5	2.586031000	1.377194000	22.949757000
1	2.348999000	0.206281000	22.898726000
1	3.752909000	1.463100000	22.496966000
1	5.785515000	3.178814000	24.122381000
5	5.885111000	2.604300000	23.046418000
1	4.484685000	2.302897000	20.912548000
1	6.288675000	1.442055000	23.239825000
1	6.800721000	3.161275000	22.437545000
1	2.594683000	1.956693000	24.108663000
5	3.091322000	1.189936000	25.062834000
1	3.927646000	0.386454000	24.725916000
1	2.152702000	0.686109000	25.608939000
1	3.558233000	2.129703000	25.677405000
3	5.418073000	1.592126000	25.026731000
3	6.342469000	1.762019000	21.144484000

{*lin*-$Li_2[B_4H_{11}]$}$^+$_MeCN E = -120.924331997 Ha

5	4.461404000	2.050639000	22.134349000
1	3.338384000	2.792324000	22.345449000
5	2.962539000	1.709421000	22.932507000
1	1.932251000	1.316916000	22.515356000
1	3.914957000	0.916435000	22.632657000
1	5.495228000	3.174270000	24.028143000
5	5.830448000	2.509219000	23.057803000
1	4.414966000	1.851135000	20.947953000
1	6.379224000	1.469662000	23.428644000
1	6.686494000	3.169800000	22.480039000
1	2.948034000	2.147828000	24.112328000
5	3.168143000	1.212204000	25.111274000
1	3.939411000	0.364394000	24.752404000
1	2.073399000	0.844312000	25.395224000
1	3.637966000	2.058804000	25.830873000
3	5.568346000	1.665220000	25.221136000
3	6.407292000	2.437457000	20.816624000

pinc-**$Li_2[B_5H_{15}]$_MeCN E = -148.286835846 Ha**

5	3.714258000	2.353175000	22.260355000
1	3.139744000	3.201492000	21.601810000
1	2.879787000	3.023073000	24.560447000
5	2.924058000	2.103865000	23.776455000
1	3.859420000	1.370985000	21.560577000
1	4.929427000	2.849519000	22.064820000
5	5.594851000	3.113667000	23.153567000
1	5.819962000	2.134924000	23.828891000
1	5.096054000	4.016104000	23.785662000
1	6.565537000	3.474088000	22.533633000
1	3.345649000	1.323394000	24.724902000
5	3.436655000	0.020441000	24.482680000
1	3.134071000	-0.331957000	23.373688000
1	4.618641000	-0.105814000	24.725345000
1	2.720217000	-0.395696000	25.346362000
3	3.100349000	4.376957000	23.225356000
3	5.027635000	0.468994000	22.872366000
1	1.803891000	1.583693000	23.437959000
5	0.732970000	2.358708000	23.711264000
1	0.033298000	1.659739000	23.020807000
1	0.493119000	2.308404000	24.885562000
1	0.879308000	3.469361000	23.259144000

{*pinc*-$Li_2[B_5H_{14}]$}$^+$_MeCN E = -147.561523802 Ha

5	3.700948000	1.615513000	22.475711000
1	3.167826000	2.169911000	21.574878000
1	3.208664000	2.154133000	24.835827000
5	2.700796000	1.564655000	23.931730000
1	4.040019000	0.507049000	22.015183000
1	4.942201000	1.894982000	22.708809000
5	5.128182000	3.160714000	23.026139000
1	5.486957000	3.045679000	24.178828000
1	4.238787000	3.947450000	22.870719000
1	6.014847000	3.382468000	22.232693000
1	2.520413000	0.444669000	24.437027000
5	3.328362000	0.049901000	23.233698000
1	2.531787000	-0.642064000	22.683388000
1	4.223324000	-0.465485000	23.826073000
3	3.080214000	4.104583000	24.521615000
3	7.398782000	3.180919000	23.618477000
1	1.476995000	1.741569000	23.618652000
5	1.088456000	3.035671000	23.767138000
1	0.035779000	2.815852000	23.241989000
1	1.031475000	3.297199000	24.938991000
1	1.783869000	3.767445000	23.107725000

1,2-**$Li_2[B_5H_{15}]$_MeCN E = -148.284984930 Ha**

5	4.491812000	2.417905000	21.972703000
1	4.189543000	3.249787000	21.157265000
1	2.490265000	3.109729000	23.671809000
5	2.629902000	1.991676000	23.270482000
1	1.620819000	1.412386000	22.992234000
1	3.237230000	1.894520000	22.167438000
1	4.933785000	3.926572000	23.874794000
5	5.463496000	3.021994000	23.266163000
1	4.855551000	1.390307000	21.417272000
1	5.997806000	2.241061000	24.049514000
1	3.278139000	1.541990000	24.289684000
5	3.442931000	0.218148000	24.268215000
1	3.572610000	-0.259612000	23.169833000
1	2.550576000	-0.252109000	24.908216000
1	4.491664000	0.285099000	24.876634000
3	6.240906000	3.874028000	25.239884000
3	5.477875000	0.581781000	23.093471000
1	6.446490000	3.407431000	22.485727000
5	7.200460000	4.417426000	22.898699000
1	6.626153000	5.436901000	22.629583000
1	8.107820000	4.143336000	22.152795000
1	7.588360000	4.348439000	24.045987000

{*1,2*-$Li_2[B_5H_{14}]$}$^+$_MeCN E = -147.545868019 Ha

5	4.496744000	2.511483000	22.018548000
1	4.143532000	3.294159000	21.174728000
1	3.053740000	3.195297000	24.194234000
5	2.944134000	2.141432000	23.616211000
1	1.810149000	1.781761000	23.537703000
1	3.226867000	2.193473000	22.381244000
1	5.611923000	4.464832000	23.112867000
5	5.646357000	3.132041000	23.104352000
1	4.760572000	1.454437000	21.481312000
1	5.783843000	2.791741000	24.254520000
1	3.745544000	1.421433000	24.292141000
5	3.586264000	0.093293000	24.147141000
1	3.227994000	-0.231692000	23.044927000
1	2.873065000	-0.254586000	25.035680000
1	4.768483000	-0.094861000	24.356187000
3	4.433882000	3.472277000	25.584897000
3	5.135038000	-0.151620000	22.398869000
1	6.856509000	3.024929000	22.581271000
5	6.817248000	4.336715000	22.624271000
1	6.802801000	4.742989000	21.507753000
1	7.621144000	4.730824000	23.403561000

1,1-**$Li_2[B_5H_{15}]$_MeCN E = -148.284187016 Ha**

5	4.554566000	2.682778000	22.027841000
1	4.042448000	3.640571000	21.481972000
1	2.482979000	3.019584000	23.360460000
5	2.767903000	1.900957000	23.054819000
1	1.874038000	1.229419000	22.639028000
1	3.562518000	1.783387000	22.050881000
1	4.982612000	3.706000000	24.195443000
5	5.610691000	3.070306000	23.342745000
1	6.239278000	2.215825000	23.942286000
1	3.327895000	1.568579000	24.156644000
5	3.541719000	0.254507000	24.260104000
1	3.823848000	-0.296481000	23.229043000
1	2.591864000	-0.204327000	24.821888000
1	4.501181000	0.430749000	24.981427000
3	4.824477000	4.969632000	22.776916000
3	5.765324000	0.557928000	23.299990000
1	6.444141000	3.843008000	22.859087000
1	4.942548000	2.209282000	20.876476000
5	5.790575000	1.183168000	20.942478000
1	5.252294000	0.239256000	21.469072000
1	5.793278000	1.053758000	19.746485000
1	6.851193000	1.498293000	21.408967000

{*1,1*-$Li_2[B_5H_{14}]$}$^+$_MeCN E = -147.566380276 Ha

5	4.368288000	2.468498000	21.677248000
1	3.954842000	3.501652000	21.239022000
1	3.031035000	3.230377000	23.566167000
5	2.931154000	2.089598000	23.216271000
1	1.822266000	1.732064000	22.981718000
1	3.437067000	1.674164000	22.082705000
5	5.854223000	2.579077000	22.640787000
1	5.759824000	2.059071000	23.720890000
1	3.568396000	1.563413000	24.182108000
5	3.480539000	0.218832000	24.221500000
1	3.699873000	-0.284478000	23.148435000
1	2.451510000	-0.095942000	24.732280000
1	4.427471000	0.183046000	24.979516000
3	4.543777000	4.673554000	22.694413000
3	5.751051000	-0.015205000	23.474450000
1	6.262716000	3.712662000	22.684252000
1	4.444746000	1.777596000	20.647058000
5	5.829261000	1.546505000	21.186063000
1	5.766821000	0.369621000	21.415245000
1	6.366453000	1.845191000	20.167360000
1	6.833445000	2.020882000	22.071122000

lin-**$Li_2[B_5H_{15}]$_MeCN E = -148.277330806 Ha**

5	4.574342000	2.378119000	21.850557000
1	3.815893000	3.064644000	21.220247000
1	2.557951000	3.025070000	23.394933000
5	2.716220000	1.855375000	23.216567000
1	1.778704000	1.246899000	22.798683000
1	3.738239000	1.479567000	22.611943000
1	5.178460000	3.763635000	23.797791000
5	5.732893000	3.195504000	22.854029000
1	4.885493000	1.410909000	21.160821000
1	6.652387000	2.504106000	23.350893000
1	6.305505000	4.048755000	22.198578000
1	3.018324000	1.629094000	24.478865000
5	2.955361000	0.366430000	24.764762000
1	3.220388000	-0.461595000	23.960806000
1	1.934191000	0.226446000	25.371381000
1	3.723605000	0.489791000	25.799180000
5	5.009590000	0.299683000	25.526542000
1	5.362708000	0.613186000	24.416957000
1	5.371155000	1.085059000	26.381408000

1	5.237688000	-0.840362000	25.801345000
3	5.784765000	2.538043000	25.077759000
3	6.346673000	1.013933000	22.285084000

{*lin*-$Li_2[B_5H_{14}]$}$^+$_MeCN E = -147.559743641 Ha

5	4.307122000	2.263314000	21.676915000
1	3.698103000	2.805259000	20.812468000
5	3.369392000	2.263045000	23.228572000
1	2.664809000	3.051104000	23.771014000
1	2.741826000	1.623616000	22.424289000
1	4.795418000	4.333069000	23.144598000
5	4.958381000	3.164722000	22.994809000
1	4.734700000	1.176924000	21.395831000
1	5.807231000	2.650159000	23.687795000
1	5.425357000	3.039263000	21.803998000
1	3.800932000	1.559619000	24.250448000
5	3.516603000	0.302418000	24.232449000
1	4.092814000	-0.337841000	23.405569000
1	2.357505000	0.067566000	24.354064000
1	3.840600000	0.064312000	25.456837000
5	5.122986000	0.214928000	25.794080000
1	5.923034000	0.509088000	24.937939000
1	4.967950000	1.085834000	26.621810000
1	5.316686000	-0.869916000	26.253469000
3	6.063489000	2.500090000	25.838478000
3	5.837648000	0.440647000	22.838767000

$LiBH_2$_MeCN E = -33.4990979138 Ha

5	1.877261000	-0.366328000	-0.398690000
1	1.860027000	0.578727000	-1.169552000
1	2.305226000	0.081071000	0.652499000
3	1.250201000	-2.508100000	-0.825288000

{LiBH}$^+$_MeCN E = -32.7368703398 Ha

5	1.735538000	-0.416774000	-0.823000000
1	1.980458000	0.752929000	-0.908301000
3	1.271493000	-2.631856000	-0.662229000

$Li[B_2H_5]$_MeCN E = -60.2207886654 Ha

5	1.469485000	-0.040700000	0.210726000
1	2.159246000	0.439963000	-0.669670000
1	0.685268000	-0.989553000	-0.377242000
1	-0.852204000	-0.546270000	0.946064000
1	-0.733117000	0.459019000	-0.810528000
5	-0.138865000	-0.031072000	0.132181000
1	2.092872000	-0.567237000	1.088802000
3	0.725349000	1.764681000	-0.925635000

{$Li[B_2H_4]$}$^+$_MeCN E = -59.4509061030 Ha

5	1.545279000	-0.065217000	-0.258367000
1	2.585806000	-0.078801000	-0.784815000
1	0.673986000	-1.048910000	-0.464678000
1	-0.719137000	-0.346116000	1.038534000
5	0.269462000	-0.166857000	0.446857000
1	1.448435000	-0.329693000	1.043327000
3	0.337319000	2.065406000	-0.615632000

$Li[B_3H_8]$_conf0_MeCN E = -86.8815377270 Ha

1	-0.756758000	5.181923000	4.888354000
1	-0.045402000	6.976828000	4.703045000
5	0.156859000	5.866186000	5.244923000
1	-0.982345000	8.026554000	6.140750000
5	0.030604000	7.395080000	6.135536000
1	-0.002196000	7.022421000	7.582288000
5	0.183947000	5.895145000	7.069993000
1	1.274911000	5.556904000	7.483541000
1	1.043601000	8.052740000	6.110020000
1	1.235412000	5.514946000	4.810185000
1	-0.718380000	5.223309000	7.475506000
3	2.292560000	6.364174000	6.119409000

{$Li[B_3H_7]$}$^+$_conf0_MeCN E = -86.1069241752 Ha

1	-0.372615000	4.807165000	5.080611000
5	0.377862000	5.559215000	5.609560000
1	-0.972529000	7.998999000	6.273380000
5	0.037003000	7.374872000	6.288318000
1	0.286552000	7.037209000	7.596900000
5	-0.237278000	5.958199000	7.088097000
1	0.657794000	5.042879000	6.852989000
1	1.001360000	7.968089000	5.926811000
1	1.433897000	5.683972000	5.077231000
1	-1.195939000	5.573497000	7.644871000
3	2.742107000	7.095287000	5.621736000

$Li[B_3H_8]$_conf1_MeCN E = -86.8781378642 Ha

1	-0.763003000	5.255547000	4.924429000
1	-0.781492000	7.190534000	4.799506000
5	-0.080207000	6.235839000	5.089966000
5	0.279998000	7.148636000	6.664077000
1	-0.650561000	7.700418000	7.171415000
5	0.275249000	5.445575000	6.721496000
1	0.804617000	6.352941000	7.562550000
1	1.212543000	7.865839000	6.385840000
1	0.913466000	6.182677000	4.381509000
1	-0.654249000	4.967431000	7.300925000
1	1.201739000	4.728659000	6.487230000
3	1.062454000	8.035711000	4.416281000

{$Li[B_3H_7]$}$^+$_conf1_MeCN E = -86.1090897476 Ha

1	-0.380325000	7.583303000	4.878814000
5	0.440483000	6.923306000	5.452762000
5	0.143389000	6.985240000	7.070758000
1	-0.621002000	7.615781000	7.699607000
5	0.146896000	5.396137000	6.625271000
1	0.746392000	6.031945000	7.703637000
1	0.996180000	7.724102000	6.392669000
1	1.366413000	6.501211000	4.834024000
1	-0.875492000	4.946195000	7.034007000
1	0.961739000	4.663165000	6.185331000
3	0.658886000	7.483875000	3.103914000

***lin*-$Li[B_4H_{11}]$_MeCN E = -113.497521659 Ha**

1	-1.089837000	5.055681000	5.045196000
1	0.072292000	6.564962000	4.671829000
5	-0.025584000	5.504864000	5.349740000
1	-0.838338000	7.849525000	5.948510000
5	0.098150000	7.117790000	6.024804000
1	0.176442000	6.989046000	7.478288000
5	0.036104000	5.777706000	7.152162000
1	1.009908000	5.255309000	7.638886000
1	0.920893000	4.857433000	4.971597000
1	-1.001973000	5.455460000	7.647978000
3	2.295546000	5.171269000	6.244893000
1	1.057044000	7.930908000	5.865129000
5	2.380066000	7.540542000	5.876962000
1	2.545961000	6.795676000	4.949669000
1	2.762698000	8.658015000	5.697884000
1	2.616859000	7.097057000	6.967807000

{*lin*-$Li[B_4H_{11}]$}$^+$_MeCN E = -112.760825298 Ha

1	-1.192226000	5.151819000	5.708572000
1	-0.048954000	6.446118000	4.601044000
5	-0.070470000	5.531323000	5.683255000
1	-0.638390000	8.174629000	5.431523000
5	0.180995000	7.326250000	5.466781000
1	-0.228265000	7.122961000	7.880184000
5	0.443167000	6.654438000	7.030211000
1	0.727460000	4.767346000	5.217652000
1	0.355897000	5.401257000	7.032358000
3	2.774577000	4.778666000	5.083046000
1	1.248557000	7.710862000	4.927489000
5	1.861497000	7.422122000	6.173488000
1	2.731016000	6.729108000	5.725384000
1	2.205490000	8.482702000	6.573268000
1	1.655972000	6.666334000	7.358193000

***cyc*-$Li[B_4H_{11}]$_MeCN E = -113.468317653 Ha**

1	-1.700353000	4.894422000	5.082444000
5	-0.511833000	5.056504000	4.972324000
1	-1.405139000	7.139422000	6.361847000
5	-0.251256000	7.393758000	6.247626000
1	0.512494000	6.692491000	7.033959000
5	0.653062000	5.459605000	6.663802000
1	1.706846000	5.389427000	6.095506000
1	0.091971000	8.436148000	6.740349000
1	0.002871000	4.049427000	4.510427000
1	0.699020000	4.992660000	7.769930000
1	-0.380055000	4.821549000	6.297388000
1	0.290299000	7.611702000	5.119025000
5	-0.067921000	6.596748000	4.313760000
1	-0.898563000	7.124327000	3.620478000
1	1.033434000	6.692375000	3.781775000
3	1.600483000	4.947772000	4.139005000

{*cyc*-$Li[B_4H_{11}]$}$^+$_MeCN E = -112.760817295 Ha

1	-0.995885000	5.552512000	4.642505000
5	-0.918958000	5.870909000	5.856222000
1	-1.992531000	5.972307000	6.335542000
5	0.106550000	7.230769000	6.110678000
1	1.220101000	6.907884000	6.592908000
5	0.675822000	5.602270000	6.705233000
1	1.565354000	5.051004000	6.119647000
1	-0.386906000	8.100982000	6.736818000
1	0.630821000	5.416271000	7.874238000
1	-0.376856000	4.791157000	6.200108000
1	0.594489000	7.666623000	5.038716000
5	-0.264409000	6.741384000	4.391468000
1	-1.031913000	7.427039000	3.804776000
1	0.594668000	6.232110000	3.727813000
3	1.952143000	4.685688000	4.102546000

***1,2*-$Li[B_5H_{14}]$_MeCN E = -140.110009587 Ha**

1	0.236793000	6.452356000	4.557331000
5	0.111383000	5.454762000	5.280981000
1	-0.879906000	7.717023000	5.806959000
5	0.096328000	7.069040000	6.002407000
1	0.077283000	6.941625000	7.397055000
5	0.062477000	5.705666000	7.076245000
1	1.032210000	5.259353000	7.631351000
1	1.036775000	4.760000000	4.982924000
1	-0.985847000	5.308031000	7.482311000
3	2.493637000	5.219360000	6.377136000
1	1.015448000	7.918002000	5.840125000
5	2.353890000	7.575966000	5.962250000
1	2.602102000	6.778515000	5.097741000
1	2.695864000	8.691955000	5.712962000
1	2.539274000	7.221649000	7.093422000
1	-1.056380000	5.174815000	4.905903000
5	-1.328544000	3.828769000	4.816030000
1	-0.807727000	3.183750000	5.679255000
1	-2.499616000	4.044563000	4.995388000
1	-1.019107000	3.549067000	3.691403000

{*1,2*-$Li[B_5H_{13}]$}$^+$_MeCN E = -139.374237706 Ha

1	0.147247000	6.596571000	4.414960000
5	0.326457000	5.397057000	4.772691000
1	-1.053755000	7.401449000	5.710888000
5	-0.005771000	6.850692000	5.790466000
1	-0.227244000	6.255847000	7.043954000
5	0.086907000	5.177591000	6.462762000
1	0.977373000	4.652591000	7.042679000
1	1.356987000	4.997840000	4.342474000
3	2.830452000	5.198420000	5.908912000
1	0.808308000	7.758657000	6.025532000
5	2.200218000	7.515755000	6.189032000
1	2.593400000	7.091722000	5.137738000
1	2.381770000	8.676944000	6.376270000
1	2.312380000	6.807569000	7.150357000
1	-0.636010000	4.968581000	4.097486000
5	-1.126436000	4.416972000	5.315782000
1	-1.009050000	4.629074000	6.718745000
1	-2.206763000	4.904929000	5.223204000
1	-0.994285000	3.247976000	5.182934000

***lin*-$Li[B_5H_{14}]$_MeCN E = -140.111995979 Ha**

1	-1.107416000	5.217691000	4.876221000
1	0.120978000	6.714000000	4.701630000
5	-0.026677000	5.574679000	5.238167000
1	-0.788098000	7.850832000	6.115568000
5	0.125265000	7.089143000	6.096870000
1	0.225355000	6.792440000	7.521536000
5	0.067010000	5.628454000	7.056265000
1	1.037341000	5.051463000	7.480378000
1	0.894444000	4.954784000	4.765096000
1	-0.967962000	5.268844000	7.531164000
3	2.261927000	4.824578000	6.082126000
1	1.110594000	7.900346000	6.065851000
5	2.438560000	7.566268000	5.919704000
1	2.560522000	6.695507000	5.122377000
1	2.764126000	8.664712000	5.611945000
1	2.556024000	7.352958000	7.173086000
5	3.760632000	6.755286000	7.335627000
1	3.556154000	5.566395000	7.350642000
1	4.548871000	7.124676000	6.512471000
1	3.903155000	7.225802000	8.428111000

{*lin*-$Li[B_5H_{13}]$}$^+$_MeCN E = -139.352372522 Ha

1	-0.486601000	5.283876000	4.475173000
1	0.674384000	6.773089000	4.886460000
5	0.384153000	5.564173000	5.237965000
1	-0.666301000	7.826074000	6.000210000
5	0.166215000	7.006058000	6.153354000
1	0.158557000	6.697612000	7.508610000
5	-0.063088000	5.530774000	6.998011000
1	0.729480000	4.891960000	7.632921000
1	1.449831000	5.043859000	5.036182000
1	-1.207959000	5.330787000	7.264376000
3	1.931704000	4.000854000	6.513358000
1	1.237112000	7.955016000	6.478418000
5	2.475238000	7.998224000	6.440694000
1	2.851839000	9.009086000	5.971055000
1	2.880121000	7.822575000	7.661895000
5	3.438602000	6.749131000	7.100046000
1	2.931196000	5.778425000	7.553238000
1	2.987340000	6.961404000	5.857906000
1	4.608462000	6.900375000	7.092586000

***1,1*-$Li[B_5H_{14}]$_MeCN E = -140.102605859 Ha**

1	-1.090251000	4.865410000	5.404716000
1	-0.123095000	6.465359000	4.877473000
5	-0.033729000	5.395022000	5.559799000
5	0.092464000	7.011352000	6.188633000
1	0.261911000	6.944747000	7.618749000
5	0.233344000	5.705283000	7.334876000
1	1.310816000	5.334025000	7.726562000
1	0.901877000	4.849714000	5.032069000
1	-0.704217000	5.293618000	7.944522000
3	2.400966000	5.126788000	6.172647000
1	1.002415000	7.827275000	5.918394000
5	2.392199000	7.541660000	5.746922000
1	2.440459000	6.813945000	4.796688000
1	2.642614000	8.689720000	5.549204000
1	2.748883000	7.100784000	6.803137000
1	-0.799771000	7.867670000	6.206238000
5	-2.264713000	7.689454000	6.182990000
1	-2.484547000	6.757923000	6.894086000
1	-2.446794000	7.555659000	5.010355000
1	-2.414082000	8.781962000	6.647676000

{*1,1*-$Li[B_5H_{13}]$}$^+$_MeCN E = -139.371329426 Ha

1	-0.943328000	5.056478000	6.284465000
1	-0.373254000	5.874742000	4.528664000
5	-0.809951000	6.105548000	5.600060000
5	0.072382000	7.309726000	6.443927000
1	0.451196000	6.973810000	7.576878000
5	-0.060478000	5.657296000	7.196738000
1	0.905820000	4.962255000	7.148035000
1	-0.745889000	5.567638000	8.162172000
3	2.839638000	5.339529000	6.531293000
1	0.882653000	7.886628000	5.710735000
5	2.252629000	7.487905000	5.676912000
1	2.218133000	6.488818000	5.008767000
1	2.534707000	8.489566000	5.100659000
1	2.669456000	7.389017000	6.797976000
1	-0.632566000	8.235755000	6.880903000
5	-1.705831000	7.620266000	6.102775000
1	-2.040660000	6.355870000	5.575429000
1	-1.905697000	8.386291000	5.222907000
1	-2.444090000	7.580517000	7.034373000

***cyc*-Li[B_5H_{14}]_MeCN E = -140.071745961 Ha**

1	-1.003832000	4.609484000	5.152135000
1	0.825554000	4.245515000	4.136472000
5	0.126436000	4.281627000	5.259920000
1	-1.251479000	7.513710000	6.400965000
5	-0.076097000	7.284959000	6.367723000
5	0.456458000	5.715069000	6.885441000
1	1.535335000	5.601678000	7.460960000
1	0.511476000	8.270229000	6.801415000
1	0.351806000	3.142772000	5.542806000
1	-0.345509000	5.059699000	7.495703000
1	0.983850000	4.990214000	5.872239000
5	0.660983000	5.244221000	3.328439000
1	-0.304139000	5.138951000	2.651769000
1	1.721768000	5.135384000	2.782025000
1	0.416126000	7.648948000	5.102388000
5	-0.112019000	7.135739000	4.069526000
1	-1.209592000	6.712100000	4.138647000
1	0.926460000	6.314269000	3.972070000
1	0.113653000	7.959714000	3.232560000
3	2.052795000	7.374520000	7.343383000

{*cyc*-Li[B_5H_{13}]}$^+$_MeCN E = -139.346349355 Ha

1	-0.315426000	3.497985000	4.999311000
1	1.331287000	4.756479000	4.343355000
5	0.633354000	4.149014000	5.253098000
1	-0.278030000	8.173641000	6.934109000
5	0.277348000	7.336848000	6.298962000
5	-0.405479000	5.683025000	6.187530000
1	0.006692000	6.253595000	7.228752000
1	1.474022000	7.382445000	6.253738000
1	1.587745000	3.547007000	5.654657000
1	-1.450282000	5.237416000	6.516280000
1	0.606129000	4.877456000	6.326437000
5	0.503465000	5.385287000	3.561770000
1	-0.495620000	4.907473000	3.160522000
1	1.348749000	5.588698000	2.739453000
1	-0.029087000	7.890289000	5.004128000
5	-0.452793000	6.725975000	4.784576000
1	-1.528412000	6.904644000	4.326324000
1	0.517737000	6.633440000	3.925471000
3	2.934981000	6.488374000	7.265553000

BH_MeCN E = -25.2824014729 Ha

5	4.045233000	-0.057097000	-0.137931000
1	3.990207000	-0.679466000	0.901242000

B^+_MeCN E = -24.4752516727 Ha

5	4.045452000	-0.054616000	-0.142074000

***st*-B_2H_4_MeCN E = -52.0082422175 Ha**

5	1.479524000	-0.052117000	0.189496000
1	2.130325000	0.920693000	-0.045746000
1	-0.826044000	0.226421000	1.054747000
1	-0.732118000	-0.305571000	-0.896587000
5	-0.147035000	-0.042838000	0.110455000
1	2.092764000	-1.032884000	0.485210000

***btf*-B_2H_4_MeCN E = -52.0037086799 Ha**

5	1.539947000	-0.050275000	-0.262122000
1	2.583868000	-0.075934000	-0.785194000
1	0.683807000	-1.062309000	-0.449251000
1	-0.718707000	-0.342761000	1.037384000
5	0.266907000	-0.152279000	0.440530000
1	1.448010000	-0.352036000	1.039510000

$B_2H_3^+$_MeCN E = -51.1723768703 Ha

5	1.344948000	0.396037000	0.068575000
1	2.480539000	0.686496000	0.051753000
1	-0.894126000	0.172869000	1.079598000
1	-0.750126000	-0.490102000	-0.880159000
5	-0.276583000	-0.018713000	0.092598000

$B_3H_5(\mu_2H)_2$_(B_3H_7) _MeCN E = -78.6702183073 Ha

1	-0.922144000	5.292235000	5.151288000
5	-0.008280000	6.031689000	5.280040000
1	-0.568359000	7.285578000	5.409137000
5	0.315288000	7.236740000	6.359909000
1	-0.256984000	7.061114000	7.512116000
5	0.280193000	5.826466000	7.216531000
1	1.302702000	5.744717000	7.821715000
1	1.108353000	8.104437000	6.336291000
1	0.793324000	6.104174000	4.402323000
1	-0.578437000	5.048057000	7.451748000

{$B_3H_4(\mu_2H)_2$}$^+$_($B_3H_6^+$) _MeCN E = -77.8847125775 Ha

1	-0.990693000	6.096589000	5.351911000
5	0.187964000	5.533591000	5.605176000
1	-0.582949000	8.103713000	5.688817000
5	-0.341882000	7.022247000	6.051506000
1	-0.516740000	6.881500000	7.361596000
5	0.535492000	6.112445000	7.101891000
1	1.318804000	6.128934000	7.964946000
1	0.563750000	4.875857000	4.719010000
1	0.183557000	4.875893000	6.759954000

$B_3H_6(\mu_2H)$_(B_3H_7)_MeCN E = -78.6662241218 Ha

1	-0.625283000	5.132884000	5.133500000
1	-0.035213000	7.006860000	4.650326000
5	0.277328000	5.897565000	5.286438000
1	-1.106519000	7.850855000	6.144704000
5	-0.003794000	7.483390000	5.877066000
1	0.344837000	6.936810000	7.950498000
5	0.404315000	6.200199000	7.026262000
1	0.675273000	5.072054000	7.257530000
1	0.880058000	8.282674000	5.925687000
1	1.360832000	5.566335000	4.913736000

{$B_3H_5(\mu_2H)$}$^+$_($B_3H_6^+$) _MeCN E = -77.8847177194 Ha

1	0.429852000	5.488346000	4.682004000
5	0.320623000	6.050622000	5.697222000
1	-0.800215000	8.415164000	6.295238000
5	-0.243831000	7.401246000	6.441547000
1	0.061462000	7.170910000	7.714211000
5	-0.239829000	5.965289000	7.239622000
1	0.815025000	5.351442000	6.714961000
1	0.815863000	7.283951000	5.649043000
1	-0.790331000	5.303389000	8.025769000

$B_2H_5(BH_2)$_(B_3H_7)_MeCN E = -78.6563776258 Ha

5	1.488154000	0.007770000	0.108627000
1	2.142361000	0.300418000	-0.848743000
1	0.627604000	-0.926767000	-0.272412000
1	-0.952710000	-0.229434000	0.790464000
1	-0.683267000	0.350499000	-1.168265000
1	0.599445000	0.976462000	0.285006000
5	-0.232355000	0.047316000	-0.112437000
5	2.348506000	-0.382557000	1.476148000
1	2.692900000	0.442144000	2.266889000
1	2.732905000	-1.492963000	1.684870000

{$B_2H_4(BH_2)$}$^+$_(B_3H_7)_MeCN E = -77.8653285569 Ha

5	1.314273000	-0.076921000	0.406876000
1	1.590600000	0.213389000	-0.855616000
1	0.363115000	-0.937697000	0.027903000
1	-0.527558000	0.423758000	-1.444011000
1	0.413888000	0.923868000	0.465625000
5	0.268018000	0.209276000	-0.628658000
5	2.486698000	-0.400864000	1.549738000
1	2.797102000	0.427918000	2.332827000
1	3.010115000	-1.460406000	1.565002000

***arachno*-B_4H_{10}_MeCN E = -104.517592389 Ha**

5	-1.931890000	2.174843000	6.147719000
5	-2.447555000	0.679494000	6.666631000
5	-0.805996000	0.761921000	6.399425000
5	-0.410904000	2.034903000	5.163253000
1	-2.233985000	3.114050000	6.781723000
1	-3.036902000	1.463028000	5.792567000
1	-3.124018000	0.295188000	7.536082000
1	-1.688517000	-0.227978000	6.096058000
1	-0.087214000	0.424110000	7.262270000
1	-0.262442000	0.716523000	5.212175000
1	0.256492000	2.697319000	5.895653000
1	-0.119499000	2.062158000	4.012719000
1	-1.644295000	2.448382000	4.902432000

***arachno*-$B_4H_9^+$_MeCN E = -104.532528181 Ha**

5	-1.727224000	2.383672000	6.540227000
5	-2.505379000	0.856037000	6.224733000
5	-0.807653000	0.891130000	6.620661000
5	-1.258269000	1.561015000	5.076976000
1	-1.441968000	3.185398000	7.346239000
1	-2.975143000	2.109916000	6.614261000
1	-2.378697000	0.859158000	4.896304000
1	-3.505106000	0.243156000	6.342916000
1	-1.616740000	-0.092813000	6.737468000
1	-0.000925000	0.854057000	7.469970000
1	-0.263032000	0.670995000	5.484853000
1	-0.916954000	1.712409000	3.959165000
1	-1.621053000	2.879765000	5.366482000

***bis*-B_4H_{10}_MeCN E = -105.308987052 Ha**

5	1.526144000	0.027208000	0.025687000
1	2.111549000	0.321298000	-0.970905000
1	0.655542000	-0.917064000	-0.287790000
1	-0.864359000	-0.236327000	0.862798000
1	-0.739097000	0.337624000	-1.114678000
1	0.639255000	0.978668000	0.260790000
5	-0.213245000	0.045914000	-0.091056000
5	2.317118000	-0.381311000	1.466680000
1	1.731716000	-0.675407000	2.463272000
1	3.187714000	0.562965000	1.780160000
1	3.204014000	-1.332764000	1.231575000
5	4.056508000	-0.400006000	1.583422000
1	4.582364000	-0.691714000	2.607041000
1	4.707618000	-0.117757000	0.629567000

***bis*-$B_4H_9^+$_MeCN E = -104.524294050 Ha**

5	1.489924000	0.116606000	-0.183696000
1	1.955859000	0.460038000	-1.216872000
1	0.689277000	-0.886813000	-0.394921000
1	-0.645375000	-0.353860000	1.068420000
1	-0.935601000	0.256449000	-0.889099000
1	0.623230000	1.007758000	0.201353000
5	-0.224148000	-0.006332000	0.018401000
5	2.521322000	-0.254669000	1.073851000
1	2.163761000	-0.585734000	2.325542000
1	3.460306000	0.574763000	1.548232000
1	3.404059000	-1.260499000	1.045250000
5	3.476109000	-0.586204000	2.182810000
1	4.216500000	-0.842419000	3.037724000

***arachno*-$B_4H_{10}(BH_3)$_MeCN E = -131.928729254 Ha**

1	0.277994000	6.519336000	4.457650000
5	0.383786000	5.325947000	4.862792000
1	-1.050257000	7.360709000	5.610054000
5	-0.006462000	6.823768000	5.795747000
1	-0.301807000	6.278810000	7.059363000
5	0.005209000	5.171657000	6.536465000
1	0.846155000	4.637897000	7.168763000
1	1.436228000	4.883733000	4.564437000
1	0.800282000	7.695090000	6.117825000
5	2.264338000	7.586426000	6.067431000
1	2.485172000	7.668643000	4.896996000
1	2.341569000	8.590623000	6.713477000
1	2.541111000	6.564026000	6.615860000
1	-0.538861000	4.913955000	4.122883000
5	-1.150559000	4.435461000	5.322210000
1	-1.129175000	4.672273000	6.726383000
1	-2.200095000	4.963770000	5.130660000
1	-1.072894000	3.255692000	5.228958000

***arachno*-$B_4H_{10}(BH_2)^+$_MeCN E = -131.142827956 Ha**

1	-0.284261000	6.686346000	4.229662000
5	0.142785000	5.502412000	4.591431000
1	-0.875538000	8.006197000	5.494159000
5	-0.112863000	7.102827000	5.497921000
1	-0.614061000	6.612334000	6.655345000
5	0.131545000	5.598281000	6.324270000
1	1.320720000	5.576664000	6.775879000
1	1.198484000	5.297971000	4.116793000
1	1.025566000	7.680581000	5.541006000
5	1.676563000	6.839142000	6.390287000
1	2.617525000	6.597579000	5.720917000
1	1.706716000	7.435436000	7.408136000
1	-0.812678000	4.864726000	4.091926000
5	-0.867326000	4.315935000	5.397192000
1	-0.451441000	4.915152000	7.117730000
1	-2.001345000	4.522606000	5.659104000
1	-0.409768000	3.229602000	5.370337000

***i-bis*-$B_4H_{10}(BH_3)$_MeCN E = -131.919092668 Ha**

5	1.430977000	-0.037957000	0.017603000
1	2.023361000	0.089957000	-1.006842000
1	0.427557000	-0.864070000	-0.191295000
1	-0.915578000	0.116567000	0.973846000
1	-0.811410000	0.524380000	-1.047561000
1	0.717195000	1.052649000	0.203570000
5	-0.285438000	0.230190000	-0.026057000
5	2.252375000	-0.444052000	1.435058000
1	3.134975000	0.504291000	1.710488000
1	3.092510000	-1.427373000	1.275059000
5	3.974220000	-0.508234000	1.638165000
1	4.413578000	-0.765838000	2.707051000
1	4.668011000	-0.317696000	0.696663000
1	1.676647000	-0.409632000	2.558611000
5	1.157181000	-1.652087000	2.849103000
1	0.100718000	-1.218658000	3.211504000
1	1.104912000	-2.341795000	1.867950000
1	1.926170000	-2.006835000	3.696042000

***i-bis*-$B_4H_{10}(BH_2)^+$_MeCN E = -131.156356588 Ha**

5	1.562153000	-0.060432000	0.509600000
1	2.469215000	0.412499000	-0.166958000
1	0.881663000	-0.824577000	-0.264519000
1	-0.912677000	-0.270495000	0.558928000
1	-0.154044000	0.769985000	-1.081240000
1	0.756289000	0.902787000	0.771786000
5	-0.022675000	0.174398000	-0.074010000

5	1.979844000	-0.911314000	1.967222000
1	3.753434000	0.775627000	1.401406000
1	3.167008000	-0.991919000	2.269994000
5	3.267534000	-0.211778000	0.971635000
1	3.880667000	-1.000335000	0.340631000
1	1.382638000	-0.389699000	2.974965000
5	0.866984000	-1.649855000	3.027606000
1	-0.277091000	-1.578627000	2.750669000
1	1.529661000	-2.112208000	1.931213000
1	1.289347000	-2.192556000	3.983368000

n-bis-$B_4H_{10}(BH_3)$_MeCN E = -131.917406720 Ha

5	1.436526000	-0.030588000	0.029472000
1	2.004619000	0.138235000	-1.004379000
1	0.502094000	-0.939706000	-0.171942000
1	-0.924803000	-0.066845000	0.973723000
1	-0.847330000	0.328540000	-1.050513000
1	0.624760000	0.993457000	0.210664000
5	-0.300313000	0.090745000	-0.024780000
5	2.251504000	-0.365237000	1.474133000
1	1.718471000	-0.513674000	2.527291000
1	3.223002000	0.507702000	1.684130000
1	3.033102000	-1.430908000	1.318874000
5	3.977949000	-0.557199000	1.488297000
1	4.647055000	-0.741041000	2.438997000
1	4.474313000	-0.117130000	0.412181000
5	5.147941000	-1.177277000	-0.120700000
1	6.265047000	-0.761379000	-0.018968000
1	4.937785000	-2.196364000	0.480116000
1	4.628279000	-1.113863000	-1.198591000

n-bis-$B_4H_{10}(BH_2)^+$_MeCN E = -131.144884304 Ha

5	1.485970000	-0.095051000	0.075467000
1	2.189640000	-0.168041000	-0.878302000
1	0.433335000	-0.847124000	-0.127800000
1	-0.896280000	0.401481000	0.779710000
1	-0.584103000	0.527358000	-1.260457000
1	0.888579000	1.070861000	0.060553000
5	-0.182549000	0.325782000	-0.164390000
5	2.049920000	-0.389396000	1.643517000
1	1.451339000	-0.279876000	2.657543000
1	3.246124000	0.288777000	1.841630000
1	2.635022000	-1.643225000	1.687071000
5	3.637042000	-0.909807000	1.935972000
1	4.559119000	-1.138431000	1.082813000
1	4.150514000	-1.167759000	3.074848000
5	5.202470000	-1.460584000	2.249671000
1	5.353091000	-2.626734000	2.262164000
1	6.032333000	-0.647894000	2.432142000

Li_2BH_MeCN E = -40.3932025845 Ha

5	4.045452000	-0.054616000	-0.142074000
1	3.989988000	-0.681947000	0.905385000
3	2.597968000	0.390187000	-1.812668000
3	5.721953000	1.107899000	-1.131207000

$\{Li_2B\}^+$_MeCN E = -39.6615849955 Ha

5	1.663657000	-0.528289000	-1.065902000
3	1.379564000	-2.708065000	-0.347846000
3	1.978928000	1.675406000	-1.694124000

$Li_2[B_2H_4]$_MeCN E = -67.1751644909 Ha

5	0.023394000	0.078419000	-0.062958000
5	1.419259000	-0.095271000	-0.799200000
1	1.581650000	-0.367921000	-1.976733000
1	-0.138968000	0.350822000	1.114638000
3	-0.236255000	-0.382875000	-2.159560000
3	1.678842000	0.365675000	1.297675000
1	-1.078253000	-0.035095000	-0.573698000
1	2.520804000	0.018688000	-0.288507000

$\{Li_2[B_2H_3]\}^+$_MeCN E = -66.4127187897 Ha

5	0.018754000	0.092469000	-0.000562000
5	1.440455000	-0.024740000	-0.516940000
1	-0.465134000	-0.762709000	0.695607000
3	-0.123395000	-1.041569000	-2.125028000
3	1.444544000	1.113177000	1.662319000
1	-0.671694000	1.039560000	-0.268774000
1	2.545293000	-0.115825000	-0.918233000

$Li_2[B_3H_7]$_conf0_MeCN E = -93.8450241019 Ha

1	-0.598376000	5.398842000	4.672642000
3	-1.087153000	7.193352000	4.237502000
5	0.337615000	5.856937000	5.307700000
1	-0.773746000	8.107591000	5.936011000
5	0.193326000	7.383550000	6.099652000
1	-0.085749000	7.041620000	7.381182000
5	0.196350000	5.764556000	7.027592000
1	1.194221000	5.607998000	7.717307000
1	1.207537000	8.036537000	6.271585000
1	1.396809000	5.580252000	4.758093000
1	-0.778680000	5.230777000	7.491543000
3	2.270767000	6.263539000	6.204147000

$\{Li_2[B_3H_6]\}^+$_conf0_MeCN E = -93.0887188017 Ha

1	-0.440439000	5.533680000	4.328178000
3	-0.858091000	7.525929000	3.976678000
5	0.344202000	5.905652000	5.169858000
1	-1.203951000	7.698214000	6.091704000
5	-0.205048000	7.043917000	6.120602000
1	0.353404000	7.180588000	7.299291000
5	0.099035000	5.823498000	7.210960000
1	1.157909000	5.295404000	7.407125000
1	1.401552000	5.352499000	5.047572000
1	-0.744728000	5.568157000	8.014884000
3	2.361539000	6.501477000	6.166520000

$Li_2[B_3H_7]$_conf1_MeCN E = -93.8328798254 Ha

5	1.431915000	-0.403794000	0.080745000
1	2.071776000	-0.826597000	-0.875971000
1	-0.785454000	-1.339389000	0.318004000
1	-0.678151000	0.553946000	0.890229000
1	-0.861292000	0.109580000	-1.024369000
1	2.089053000	0.810623000	0.386184000
5	-0.308988000	-0.236398000	0.020466000
5	2.353495000	-0.041210000	1.368938000
1	1.856351000	0.431455000	2.380629000
1	3.543234000	-0.211227000	1.444394000
3	0.632902000	-0.331787000	-2.027536000
3	0.288817000	-0.796006000	1.998228000

$\{Li_2[B_3H_6]\}^+$_conf1_MeCN E = -93.0866560984 Ha

5	1.619649000	0.254534000	-0.386423000
1	2.395566000	0.166588000	-1.286904000
1	-0.546322000	0.017316000	0.810969000
1	-0.076554000	-1.443922000	-0.520157000
1	2.178663000	0.954469000	0.599310000
5	0.276481000	-0.440680000	0.060360000
5	1.943853000	-0.251296000	1.172520000
1	1.424567000	0.007973000	2.221219000
1	3.004304000	-0.793932000	1.275899000
3	0.348796000	-0.277640000	-2.239690000
3	-0.257194000	-1.028161000	2.362609000

lin-$Li_2[B_4H_{10}]$_MeCN E = -120.475768438 Ha

1	-0.152985000	5.520715000	4.916804000
3	0.278262000	7.471780000	4.233579000
5	0.651809000	5.914207000	5.749568000
1	-0.614582000	8.015846000	6.097837000
5	0.379163000	7.436907000	6.496497000
1	-0.007200000	7.140759000	7.731206000
5	0.301059000	5.854323000	7.426223000
1	1.203086000	5.636332000	8.192602000
1	1.319517000	8.164730000	6.695239000
1	-0.759296000	5.364944000	7.782005000
3	-0.717022000	4.184895000	6.249274000
1	1.744226000	5.317121000	5.599388000
5	2.422540000	6.139550000	4.708864000
1	2.975546000	5.157804000	4.258511000
1	1.955261000	6.761648000	3.772025000
1	3.153036000	6.809685000	5.393170000

$\{$_lin_-$Li_2[B_4H_9]\}^+$_MeCN E = -119.771045221 Ha

1	0.042679000	5.158211000	4.929251000
3	0.284252000	6.744487000	3.771373000
5	0.881279000	5.482788000	5.758549000
1	-0.322845000	7.618362000	5.779790000
5	0.610900000	7.082926000	6.297375000
1	0.482885000	7.152365000	7.597198000
5	0.401230000	5.805555000	7.474575000
1	1.275294000	5.461031000	8.207074000
1	1.674623000	7.832016000	6.191960000
1	-0.721168000	5.608478000	7.874509000
3	-1.396277000	4.790759000	6.246718000
1	1.708107000	4.644017000	5.937931000
5	2.059309000	6.824903000	5.364271000
1	2.069532000	7.359261000	4.281916000
1	3.127362000	6.564438000	5.818280000

cyc-$Li_2[B_4H_{10}]$_MeCN E = -120.465182617 Ha

1	-1.754587000	5.126155000	5.553130000
5	-0.677151000	5.127266000	4.986164000
1	-0.973567000	7.492759000	6.736281000
5	0.125207000	7.281526000	6.226681000
5	0.814951000	5.711290000	6.427766000
1	1.907042000	5.558213000	5.911697000
1	0.860247000	8.209645000	6.498239000
1	-0.557156000	4.097543000	4.330491000
1	0.825159000	5.236371000	7.558813000
1	0.135850000	4.662958000	5.945184000
1	-0.131948000	7.685368000	4.994288000
5	-0.254357000	6.617664000	4.223848000
1	-1.111962000	7.095314000	3.508482000
1	0.821622000	6.674381000	3.631843000
3	1.345502000	4.934997000	4.036898000
3	-1.194083000	5.777930000	7.418557000

$\{$_cyc_-$Li_2[B_4H_9]\}^+$_MeCN E = -119.770486850 Ha

1	-1.892019000	4.892784000	4.916305000
5	-0.774896000	5.118676000	4.577719000
1	-1.070863000	7.595825000	6.846886000
5	-0.029801000	7.384162000	6.271016000
5	0.246758000	5.611974000	6.049497000
1	0.869633000	8.118180000	6.546805000
1	-0.316895000	4.380392000	3.741381000
1	1.380581000	5.201774000	6.153516000
1	-0.520258000	5.018895000	6.759140000
1	-0.421357000	7.809533000	5.056027000
5	0.050799000	6.634891000	4.694683000
1	-0.942416000	6.295870000	3.919709000
1	1.037435000	6.788924000	4.040700000
3	1.597592000	4.657527000	4.341986000
3	-0.940566000	6.221760000	8.161293000

bis-$Li_2[B_4H_{10}]$_MeCN E = -120.453322607 Ha

5	1.407686000	-0.486645000	0.017197000
1	1.536275000	-1.228474000	-0.934454000
1	0.049197000	-0.463859000	0.119561000
1	-0.043480000	1.526039000	0.549721000
1	-0.408376000	0.838947000	-1.346517000
1	1.459042000	0.740782000	-0.557017000
5	0.191603000	0.737467000	-0.325482000
5	2.276570000	-0.575830000	1.518780000
1	1.499011000	-1.104631000	2.340010000
1	2.421207000	0.600976000	1.907624000
1	3.721607000	-2.636231000	1.440467000
5	3.848081000	-1.408335000	1.636832000
1	4.402003000	-1.265871000	2.713323000
1	4.640037000	-1.040064000	0.744309000
3	0.637915000	0.583094000	2.361137000
3	3.241102000	-1.944112000	-0.237403000

$\{$_bis_-$Li_2[B_4H_9]\}^+$_MeCN E = -119.726506674 Ha

5	1.574195000	-0.462204000	-0.060213000
1	1.844134000	-1.111468000	-1.040588000
1	0.258045000	-0.613398000	0.105231000
1	-0.098685000	1.321773000	0.611774000
1	-0.381890000	0.702653000	-1.315054000
1	1.497185000	0.781269000	-0.558831000
5	0.229578000	0.622003000	-0.304087000
5	2.400742000	-0.660788000	1.415991000
1	1.963718000	-0.167086000	2.438261000
1	4.331068000	-2.061751000	0.703930000
5	3.766644000	-1.507786000	1.628656000
1	4.349496000	-1.583100000	2.670614000
1	3.639176000	-0.182410000	1.269015000
3	1.395928000	1.543929000	1.992309000
3	2.611133000	-2.643750000	0.051070000

_1,1-BB_-$Li_2[B_4H_{10}]$_MeCN E = -120.429713059 Ha

5	1.605701000	0.128756000	-0.020730000
1	2.233739000	0.707566000	-0.880034000
1	0.457938000	-0.126920000	-0.312964000
5	2.227514000	-0.259862000	1.362606000
1	3.226808000	0.303809000	1.903556000
1	4.161624000	-1.714322000	1.620848000
5	4.264629000	-0.512065000	1.539595000
1	4.939108000	-0.067146000	2.431665000
1	4.641472000	-0.131273000	0.451819000
3	0.507758000	1.316598000	1.783256000
3	3.393732000	-1.482694000	-0.300273000
1	1.811056000	-1.186803000	2.121909000
5	1.002809000	-0.514571000	3.000312000
1	1.075900000	-1.427990000	3.780617000
1	1.367239000	0.524528000	3.500993000
1	-0.092163000	-0.444421000	2.484424000

$\{$_1,1-BB_-$Li_2[B_4H_9]\}^+$_MeCN E = -119.727541977 Ha

5	1.444138000	-0.019140000	0.533336000
1	1.540198000	1.090497000	0.026102000
1	1.095824000	-0.862942000	-0.266169000
5	2.717190000	-0.388621000	1.556394000
1	3.752613000	0.405232000	1.692468000
1	3.343432000	-1.540144000	1.495966000
5	4.393996000	-0.755989000	1.691681000
1	4.887908000	-0.967890000	2.746803000
1	5.032309000	-0.787317000	0.688928000
3	0.122685000	1.704131000	1.222478000
3	2.857361000	-0.312155000	-1.091602000
1	2.280495000	-0.414362000	2.775153000
5	1.012661000	-0.188806000	2.184176000
1	0.771972000	0.781470000	2.871730000
1	0.496184000	-1.202784000	2.559536000

_tr-BB_-$Li_2[B_4H_{10}]$_MeCN E = -120.426547228 Ha

5	0.214749000	-0.178451000	1.507100000
1	1.174095000	-0.146283000	2.228213000
1	-0.272054000	1.005302000	1.505030000
3	-1.997127000	0.096475000	0.658838000
5	-0.395532000	-1.427990000	0.799945000
1	-1.300844000	-1.529388000	0.002531000
3	1.664297000	-0.732638000	-0.197161000
5	0.095974000	1.542458000	0.275657000
1	1.174568000	1.302876000	-0.212594000
1	0.062520000	2.649212000	0.750204000
1	-0.830755000	1.321893000	-0.474756000
1	-0.087075000	-2.582035000	1.231454000
5	0.985476000	-2.880721000	0.399540000
1	0.926565000	-2.558729000	-0.767794000

1	1.986782000	-2.495448000	0.957166000
1	0.789978000	-4.060567000	0.531979000

{*tr-BB*-$Li_2[B_4H_9]$}$^+$_MeCN E = -119.770486850 Ha

1	-1.892019000	4.892784000	4.916305000
5	-0.774896000	5.118676000	4.577719000
1	-1.070863000	7.595825000	6.846886000
5	-0.029801000	7.384162000	6.271016000
5	0.246758000	5.611974000	6.049497000
1	0.869633000	8.118180000	6.546805000
1	-0.316895000	4.380392000	3.741381000
1	1.380581000	5.201774000	6.153516000
1	-0.520258000	5.018895000	6.759140000
1	-0.421357000	7.809533000	5.056027000
5	0.050799000	6.634891000	4.694683000
1	-0.942416000	6.295870000	3.919709000
1	1.037435000	6.788924000	4.040700000
3	1.597592000	4.657527000	4.341986000
3	-0.940566000	6.221760000	8.161293000

***lin-BB*-$Li_2[B_4H_{10}]$_MeCN E = -120.419380287 Ha**

5	4.732299000	1.772503000	22.241243000
1	2.345008000	2.342573000	22.268705000
5	2.806089000	1.475664000	22.945468000
1	2.205409000	0.443755000	22.903973000
1	3.987972000	0.869856000	22.753358000
1	5.652820000	3.018804000	24.202622000
5	5.693606000	2.733893000	23.024543000
1	4.741863000	1.505914000	21.058027000
1	6.612083000	3.325254000	22.483749000
1	2.829389000	2.102600000	24.065771000
5	3.015433000	1.299837000	25.123146000
1	3.617051000	0.276731000	24.905804000
1	1.925210000	1.119442000	25.578121000
1	3.681550000	2.121443000	25.711671000
3	5.377674000	1.169059000	24.652707000
3	5.698231000	3.153239000	20.838447000

{*lin-BB*-$Li_2[B_4H_9]$}$^+$_MeCN E = -119.772775153 Ha

5	4.415735000	0.988610000	23.432450000
1	3.796467000	3.420100000	23.275040000
5	3.252223000	2.216265000	23.214520000
1	2.643197000	2.111774000	22.195637000
1	4.344026000	0.179443000	22.538313000
1	5.572942000	3.147265000	24.028249000
5	4.925942000	2.710708000	23.114883000
1	5.317656000	3.073056000	22.036319000
1	2.420291000	2.351614000	24.210933000
5	2.989442000	1.166623000	24.567293000
1	5.247696000	0.637247000	24.222618000
1	2.078411000	0.401443000	24.536501000
1	3.369845000	1.468246000	25.671936000
3	5.268218000	1.923407000	25.550498000
3	4.537732000	1.428853000	21.104138000

***1,1*-$Li_2[B_5H_{13}]$_MeCN E = -147.107011882 Ha**

3	1.563450000	8.225417000	4.944250000
5	0.865754000	5.936767000	5.727214000
1	-0.976507000	7.536541000	5.950167000
5	0.105934000	7.308739000	6.423977000
1	-0.206489000	6.931885000	7.664523000
5	0.342214000	5.757386000	7.351403000
1	1.254072000	5.688082000	8.133493000
1	0.750071000	8.301837000	6.698261000
1	-0.603046000	5.044258000	7.623446000
3	0.162755000	3.656581000	6.517084000
1	2.044433000	5.495428000	5.749978000
5	2.779942000	6.387865000	5.042633000
1	3.654120000	5.564448000	4.953631000
1	2.405314000	6.733675000	3.944457000
1	3.096197000	7.308127000	5.765118000
1	0.290532000	5.820114000	4.613820000
5	0.016151000	4.499018000	4.483058000
1	-1.019622000	4.246349000	5.059091000
1	-0.156816000	4.674865000	3.304101000
1	0.891938000	3.684437000	4.670472000

{*1,1*-$Li_2[B_5H_{12}]$}$^+$_MeCN E = -146.377673768 Ha

3	1.934708000	8.579000000	5.571074000
5	0.847077000	6.140845000	5.285244000
1	-0.955323000	7.655690000	5.885120000
5	0.090078000	7.258125000	6.316442000
1	-0.388444000	6.461879000	7.310113000
5	0.211210000	5.446371000	6.729915000
1	1.215360000	5.176382000	7.337870000
1	0.713290000	8.044189000	6.991415000
1	-0.681854000	4.632584000	6.864970000
3	0.500416000	3.637885000	8.320086000
1	1.983309000	5.592540000	5.180823000
5	2.835076000	6.593785000	4.910017000
1	3.643757000	5.805401000	4.509531000
1	2.504031000	7.380208000	4.057047000
1	3.138930000	7.089101000	5.969537000
1	0.235664000	5.936470000	4.207197000
5	-0.242969000	4.837446000	5.002262000
1	-1.403043000	5.063827000	4.849203000
1	0.187187000	3.785654000	4.651838000

***1,2*-$Li_2[B_5H_{13}]$_MeCN E = -147.100994834 Ha**

1	-0.431266000	6.146053000	5.116994000
3	1.534727000	8.273482000	5.120639000
5	0.525869000	6.156303000	5.855929000
5	0.700221000	7.390236000	7.010480000
1	0.649901000	6.787532000	8.187455000
5	0.457675000	5.625639000	7.488251000
1	1.387401000	5.009510000	7.931716000
1	1.702385000	8.043399000	7.155863000
1	-0.616181000	5.330755000	7.979877000
3	-1.295768000	4.847464000	6.219176000
1	1.467981000	5.428667000	5.406156000
5	2.167818000	6.217067000	4.559021000
1	2.639790000	5.246948000	4.022424000
1	1.579449000	6.898249000	3.747491000
1	2.995762000	6.835371000	5.191101000
1	-0.304433000	8.089060000	7.399862000
5	-0.857027000	8.809587000	6.417972000
1	-1.756425000	8.139673000	5.990734000
1	-1.193511000	9.679396000	7.182973000
1	-0.114081000	9.246897000	5.570625000

{*1,2*-$Li_2[B_5H_{12}]$}$^+$_MeCN E = -146.389402596 Ha

1	-0.574699000	6.210984000	5.398319000
3	1.650668000	8.767275000	4.209511000
5	0.401497000	6.476136000	6.030937000
5	0.878338000	7.315907000	7.435802000
1	0.386431000	6.726000000	8.514061000
5	0.222356000	5.712562000	7.665758000
1	0.983652000	4.876972000	8.041892000
1	2.045016000	7.470339000	7.555137000
1	-0.948939000	5.503562000	7.871396000
3	-1.412211000	4.665831000	6.213527000
1	1.450997000	5.910883000	5.577560000
5	1.948001000	6.511204000	4.494658000
1	2.472179000	5.506546000	4.104389000
1	1.144658000	6.943260000	3.704381000
1	2.736437000	7.326446000	4.917544000
1	0.239721000	8.443299000	7.572972000
5	-0.011855000	8.232580000	6.260655000
1	-1.199583000	8.317287000	6.241200000
1	0.584048000	9.144543000	5.754306000

***bis*-$Li_2[B_5H_{13}]^+$_conf0_MeCN E = -147.090545340 Ha**

5	1.342521000	-0.533275000	-0.010470000
1	1.354057000	-1.325672000	-0.926526000
1	0.005394000	-0.371902000	0.150630000
1	0.140263000	1.628850000	0.516993000
1	-0.382852000	0.920597000	-1.340582000
1	1.503141000	0.659807000	-0.622653000
5	0.248899000	0.798681000	-0.341987000
5	2.248443000	-0.633829000	1.463087000
1	1.533909000	-1.221049000	2.295567000
1	2.378486000	0.531052000	1.871876000
1	4.032661000	-2.518972000	1.272957000
5	3.836446000	-1.353933000	1.606821000
1	4.338372000	-1.193437000	2.696748000
1	4.878228000	-0.709372000	1.003087000
5	4.801177000	-0.370577000	-0.238389000
1	4.325793000	0.738509000	-0.279707000
1	5.988056000	-0.395558000	-0.483250000
1	4.226025000	-1.147908000	-0.975294000
3	0.621837000	0.491188000	2.413919000
3	2.917997000	-2.222275000	-0.169948000

{*bis*-$Li_2[B_5H_{12}]$}$^+$_conf0_MeCN E = -146.349686684 Ha

5	1.287184000	-0.368375000	-0.126196000
1	1.393372000	-0.952747000	-1.172396000
1	-0.043604000	-0.312828000	0.027201000
1	0.019926000	1.545354000	0.901867000
1	-0.447812000	1.324132000	-1.082552000
1	1.436319000	0.917848000	-0.434715000
5	0.164089000	0.961701000	-0.135125000
5	2.154465000	-0.816542000	1.272929000
1	1.553469000	-1.290241000	2.215778000
1	2.808732000	0.230105000	1.775209000
5	3.747380000	-0.776907000	1.493358000
1	4.342887000	-1.062609000	2.481650000
1	4.499268000	0.045292000	0.818459000
5	4.876816000	-0.718453000	-0.187610000
1	4.103767000	-0.536880000	-1.098365000
1	5.930558000	-0.167263000	-0.327840000
1	5.023340000	-1.878896000	0.131936000
3	0.391878000	0.257189000	2.547411000
3	3.064159000	-2.109982000	-0.471077000

***bis*-$Li_2[B_5H_{13}]^+$_conf1_MeCN E = -147.076094452 Ha**

5	1.454720000	-0.423326000	0.371434000
1	2.118837000	-1.091904000	-0.385228000
1	0.171394000	-0.798947000	0.246262000
1	-0.702633000	0.999585000	0.097622000
1	-0.073280000	0.087966000	-1.611106000
1	1.268969000	0.785003000	-0.193892000
5	0.091104000	0.301684000	-0.440925000
5	1.894846000	-0.283308000	2.004482000
1	1.240370000	0.491144000	2.670600000
1	3.041551000	0.282084000	1.927196000
1	1.903537000	-1.380264000	2.537029000
5	4.295077000	-0.150630000	2.415332000
1	4.781615000	0.943581000	2.239756000
1	4.087707000	-0.301495000	3.615098000
5	4.930152000	-1.452676000	1.448691000
1	4.404111000	-2.573802000	1.613640000
1	4.820843000	-1.216321000	0.249066000
1	6.124807000	-1.594158000	1.707544000
3	3.548576000	-2.069641000	3.137868000
3	1.432905000	-1.046477000	-2.187372000

{*bis*-$Li_2[B_5H_{12}]$}$^+$_conf1_MeCN E = -146.394134705 Ha

5	1.888422000	-0.498912000	0.217432000
1	2.192557000	-0.895974000	-0.877000000
1	0.573782000	-0.684189000	0.313039000
1	0.168152000	1.071205000	1.224873000
1	0.041172000	0.910504000	-0.832793000
1	1.837782000	0.828751000	0.116050000
5	0.553327000	0.616238000	0.196732000
5	2.634268000	-1.053198000	1.656198000
1	2.037123000	-0.622746000	2.622760000
1	2.486664000	-2.262220000	1.534806000
5	4.112051000	-0.044270000	1.295630000
1	4.493406000	-0.012246000	0.151348000
1	3.887151000	1.012480000	1.794737000
5	4.365400000	-1.549003000	2.078596000
1	4.409604000	-1.799630000	3.252418000
1	4.849340000	-2.406166000	1.384653000
1	5.157370000	-0.500176000	1.989676000
3	2.506885000	-2.028968000	3.764536000
3	3.854804000	-1.904388000	-0.322462000

***cyc*-$Li_2[B_5H_{13}]$_MeCN E = -147.075807594 Ha**

1	-0.990627000	4.446195000	5.473739000
3	-1.596368000	5.076537000	3.815097000
5	0.164282000	4.350040000	5.090866000
1	-1.400441000	6.998427000	6.215410000
5	-0.212032000	7.072317000	6.355346000
5	0.635593000	5.664551000	6.880815000
1	1.810683000	5.791931000	7.238742000
1	0.060694000	8.168927000	6.829716000
1	0.498379000	3.186422000	5.183390000
1	0.056642000	5.033123000	7.733777000
1	1.034530000	4.832456000	5.946388000
5	0.641459000	5.034812000	3.576308000
1	-0.086554000	4.876874000	2.608552000
1	1.786142000	4.819981000	3.259619000
1	0.377187000	7.430195000	5.117736000
5	0.015484000	7.183035000	3.935658000
1	-1.143164000	6.925113000	3.776266000
1	0.918328000	6.391453000	3.536594000
1	0.331061000	8.178580000	3.338399000
3	1.794958000	7.628418000	7.346768000

{*cyc*-$Li_2[B_5H_{12}]$}$^+$_MeCN E = -146.365670185 Ha

1	-1.696808000	5.900987000	5.409393000
3	-1.361906000	4.481187000	3.649228000
5	0.290237000	4.757893000	5.266080000
1	-0.712707000	6.964165000	6.684695000
5	-0.602594000	6.297066000	5.683485000
5	0.775660000	5.633490000	6.642989000
1	1.640149000	6.472369000	6.581604000
1	-0.409734000	3.800810000	5.485911000
1	0.464688000	5.246758000	7.739131000
1	1.341556000	4.597877000	6.027764000
5	0.875502000	5.104459000	3.683134000
1	0.315401000	4.643000000	2.710785000
1	2.066296000	4.970336000	3.571009000
1	-0.172285000	7.322659000	4.977297000
5	-0.092415000	7.015487000	3.716918000
1	-1.094383000	6.643188000	3.189928000
1	1.009910000	6.435767000	3.441685000
1	0.233749000	8.120202000	3.376708000
3	1.765227000	6.512761000	8.591726000

***pinc-BB*-$Li_2[B_5H_{13}]$_MeCN E = -147.054900171 Ha**

5	3.875884000	2.328731000	22.353903000
1	3.449431000	2.755136000	21.321784000
5	3.113862000	1.843231000	23.622885000
1	5.140281000	2.117399000	22.279990000
5	5.669113000	3.258514000	22.831428000
1	5.811828000	3.054368000	24.009259000
1	5.072506000	4.280761000	22.564288000
1	6.676599000	3.174479000	22.178743000
1	3.688780000	1.728613000	24.745093000
5	3.907059000	0.373011000	24.818708000
1	3.000589000	-0.381093000	24.545865000
1	4.937152000	0.122066000	24.234201000
1	4.058218000	0.463308000	26.008382000
3	3.532135000	4.130163000	23.898985000

3	3.709426000	-0.239802000	22.653414000
1	1.877671000	1.653375000	23.705948000
5	1.476243000	2.934438000	24.176300000
1	0.431907000	2.440007000	24.515377000
1	2.060857000	3.403581000	25.129306000
1	1.371023000	3.692218000	23.243974000

{*pinc-BB*-Li$_2$[B$_5$H$_{12}$]}$^+$_MeCN E = -146.350526975 Ha

5	4.632080000	2.077121000	22.952636000
1	5.513891000	1.315295000	22.645166000
5	3.230910000	1.498082000	23.681933000
1	5.232245000	3.073735000	23.608438000
5	5.047792000	3.695962000	22.474183000
1	4.314807000	4.622652000	22.649152000
1	4.349351000	2.779130000	21.858375000
1	6.076118000	3.898165000	21.923904000
1	3.466131000	0.993598000	24.865454000
5	2.816179000	-0.001006000	24.107176000
1	1.774667000	-0.266062000	24.625832000
1	3.605346000	-0.917362000	24.032549000
3	3.243632000	5.128107000	24.254570000
3	4.213465000	-0.262971000	22.293575000
1	2.139731000	2.168482000	23.666928000
5	2.122075000	3.119198000	24.599473000
1	1.274707000	2.691382000	25.330169000
1	3.147772000	3.398865000	25.172077000
1	1.722513000	3.998067000	23.862042000

***1,1-BB*-Li$_2$[B$_5$H$_{13}$]_MeCN E = -147.048318143 Ha**

5	4.159268000	2.159078000	22.539658000
1	3.730317000	2.655381000	21.522593000
5	3.236040000	1.828951000	23.766240000
1	5.349575000	1.956309000	22.544823000
1	3.678523000	1.749640000	24.954772000
5	3.774221000	0.390044000	25.076529000
1	2.981472000	-0.424040000	24.698072000
1	3.785035000	0.478475000	26.271222000
3	3.269444000	4.193874000	23.630980000
3	3.425256000	-0.280583000	22.743036000
1	1.989775000	1.637299000	23.751420000
5	1.511445000	2.892858000	24.108532000
1	0.449138000	2.392006000	24.368652000
1	1.968140000	3.482337000	25.060831000
1	1.464395000	3.562569000	23.099794000
1	4.999484000	0.340665000	24.705473000
1	5.124269000	-1.699304000	25.243311000
5	5.418062000	-0.902254000	24.400916000
1	5.043602000	-1.173022000	23.282164000
1	6.565114000	-0.541744000	24.415604000

{*1,1-BB*-Li$_2$[B$_5$H$_{12}$]}$^+$_MeCN E = -146.388560990 Ha

5	4.657783000	1.789222000	23.235889000
1	4.359755000	1.857293000	22.071373000
5	3.456675000	1.586109000	24.546090000
1	5.461739000	2.615441000	23.566549000
1	3.631302000	2.430386000	25.371265000
5	4.096422000	0.293758000	25.697296000
1	3.354115000	-0.527072000	26.134926000
1	4.880849000	0.810210000	26.428782000
3	3.825304000	3.926872000	24.131817000
3	2.868877000	0.663673000	21.797498000
1	2.228359000	1.279363000	24.424466000
5	1.595732000	2.170963000	23.627939000
1	0.605913000	2.160939000	24.304207000
1	2.032330000	3.278226000	23.455103000
1	1.438579000	1.564252000	22.593987000
1	4.958150000	-0.420007000	24.892598000
1	3.702137000	-0.527781000	23.308933000
5	4.326732000	0.235656000	23.981415000
1	5.398221000	0.684058000	23.332322000

***tr-BB*-Li$_2$[B$_5$H$_{13}$]_MeCN E = -147.049098383 Ha**

5	4.597511000	2.684046000	22.260436000
1	2.585580000	3.221035000	23.526144000
5	2.842803000	2.099057000	23.182884000
1	1.920215000	1.540934000	22.670858000
1	3.698527000	1.765739000	22.216537000
1	6.135382000	2.010939000	24.152093000
5	5.847183000	2.676626000	23.188241000
1	4.343995000	3.443158000	21.364984000
1	3.254091000	1.645342000	24.308650000
5	3.371489000	0.305023000	24.334613000
1	3.583442000	-0.213608000	23.266788000
1	2.378201000	-0.084905000	24.870768000
1	4.348886000	0.342804000	25.047446000
3	4.413285000	4.594986000	23.526138000
3	5.514511000	0.407937000	23.218965000
1	6.885617000	3.296505000	22.778471000
5	6.718235000	4.525497000	23.365433000
1	6.131564000	5.210167000	22.557689000
1	7.903334000	4.729150000	23.384501000
1	6.244968000	4.582089000	24.479316000

{*tr-BB*-Li$_2$[B$_5$H$_{12}$]}$^+$_MeCN E = -146.394727865 Ha

5	4.494180000	1.776920000	22.937446000
1	4.007797000	2.446086000	25.395165000
5	3.459106000	2.343400000	24.325713000
1	2.919766000	3.357344000	23.969186000
1	4.069169000	2.115208000	21.851087000
1	6.563218000	2.258790000	24.378463000
5	5.846415000	2.663077000	23.503435000
1	2.442637000	1.537268000	24.518390000
5	3.216937000	0.735254000	23.766532000
1	4.965158000	0.661662000	22.812561000
1	2.472183000	0.345698000	22.926965000
1	3.616552000	-0.104697000	24.534971000
3	3.591863000	3.867510000	22.132307000
3	5.288782000	0.798924000	25.072806000
1	6.661369000	2.979193000	22.488061000
5	6.297894000	4.188601000	22.798616000
1	5.574548000	4.612690000	21.943173000
1	7.256927000	4.815269000	23.100500000
1	5.630322000	3.941167000	23.880596000

Li[B$_2$H$_3$]_MeCN E = -58.9679475224 Ha

5	1.010776000	0.581857000	0.183087000
1	2.061948000	0.828907000	0.653333000
1	0.156107000	-0.118563000	0.931660000
1	-1.396264000	-0.167647000	-0.551191000
5	-0.310679000	0.196364000	-0.275917000
3	1.283700000	-0.949797000	-1.608079000

{Li[B$_2$H$_2$]}$^+$_MeCN E = -58.1577370651 Ha

5	0.470962000	-0.169663000	0.582776000
1	1.383934000	-0.372349000	1.281216000
1	-1.666757000	0.316882000	-0.982432000
5	-0.735299000	0.103867000	-0.312031000
3	1.290798000	-0.336524000	-1.889967000

Li[B$_3$H$_6$]_conf0_MeCN E = -85.6400986607 Ha

1	-0.410496000	5.496016000	4.312095000
3	-0.922692000	7.441223000	4.021669000
5	0.360716000	5.925206000	5.153742000
1	-1.196487000	7.718040000	6.146101000
5	-0.203302000	7.047555000	6.113290000
1	0.444096000	7.151149000	7.257049000
5	0.082826000	5.824653000	7.200029000
1	1.081681000	5.201528000	7.380382000
1	1.460233000	5.466764000	5.079416000
1	-0.792731000	5.655404000	8.003080000

{Li[B$_3$H$_5$]}$^+$_conf0_MeCN E = -84.8979332547 Ha

1	-0.073352000	5.714410000	4.050090000
3	-1.099816000	7.665105000	3.929562000
5	0.184632000	5.885056000	5.187461000
1	-1.112690000	7.845875000	6.114265000
5	-0.344151000	6.959873000	6.232837000
1	0.096219000	6.941709000	7.384105000
5	0.686614000	5.850229000	6.685850000
1	1.341140000	5.355553000	7.526076000
1	1.017979000	5.054323000	5.553798000

Li[B$_3$H$_6$]_conf1_MeCN E = -85.6314444486 Ha

5	1.637591000	6.160933000	5.708224000
1	0.772900000	6.711055000	6.293639000
5	3.049320000	7.001684000	5.115361000
1	2.665480000	7.918970000	4.432675000
1	3.571685000	7.385812000	6.158986000
1	3.884280000	6.372489000	4.462697000
5	2.422631000	4.973834000	5.060725000
1	3.248787000	4.366568000	5.704298000
1	2.290100000	4.620010000	3.926840000
3	4.670992000	5.792705000	6.097686000

{Li[B$_3$H$_5$]}$^+$_conf1_MeCN E = -84.8973460108 Ha

5	1.856646000	6.036679000	5.636009000
1	0.973391000	6.425245000	6.300799000
5	3.147293000	6.844960000	5.124825000
1	3.427443000	7.963745000	5.333144000
1	3.986307000	6.274451000	4.425024000
5	2.975289000	5.309905000	4.786330000
1	3.376043000	4.340146000	4.256054000
1	1.723102000	4.850413000	5.328194000
3	4.082772000	5.339546000	7.338077000

***arachno*-Li[B$_4$H$_9$]_MeCN E = -112.321268918 Ha**

5	-1.931323000	2.287729000	6.562652000
5	-2.691138000	0.618156000	6.616904000
5	-1.006785000	0.890745000	6.277133000
5	-0.818154000	2.166191000	5.108747000
1	-1.554599000	2.815953000	7.571059000
1	-2.839573000	2.912300000	6.079048000
1	-3.516327000	0.496328000	5.739705000
1	-2.993243000	0.094843000	7.646447000
1	-1.744995000	-0.195633000	6.116762000
1	-0.170266000	0.669075000	7.087553000
1	-0.452575000	0.872665000	5.075306000
1	0.220939000	2.751592000	5.058741000
1	-1.454168000	2.201030000	4.079593000
3	-3.288327000	2.010948000	4.585063000

{*arachno*-Li[B$_4$H$_8$]}$^+$_MeCN E = -111.557750718 Ha

5	-2.017995000	2.200995000	6.147800000
5	-2.705804000	0.613140000	6.044610000
5	-0.994985000	0.858166000	6.476060000
5	-0.497973000	2.227522000	5.605695000
1	-2.495894000	3.157149000	6.663728000
1	-1.570474000	2.502256000	4.893303000
1	-3.117744000	0.473950000	4.934539000
1	-3.385981000	0.187218000	6.940761000
1	-1.673646000	-0.201160000	5.996719000
1	-0.448866000	0.504588000	7.459356000
1	-0.148359000	0.959201000	5.428380000
1	0.428605000	2.926232000	5.438997000
3	-4.157249000	1.981635000	7.495171000

***bis*-Li[B$_4$H$_9$]_conf0_MeCN E = -112.279585850 Ha**

5	1.582531000	0.374659000	0.157652000
1	2.320797000	1.040963000	-0.526819000
1	1.003561000	-0.533323000	-0.676931000
1	-0.786099000	-0.403682000	0.334360000
1	-0.459935000	0.846934000	-1.219401000
5	0.016124000	0.293691000	-0.266741000
5	2.320169000	-0.492792000	1.426516000
1	1.704630000	-1.090766000	2.280432000
1	3.164700000	0.297708000	2.080633000
1	3.239341000	-1.349247000	0.995690000
5	4.040727000	-0.596575000	1.705471000
1	4.454436000	-1.195169000	2.644587000
1	4.792078000	-0.066834000	0.950403000
3	0.085064000	0.093725000	1.959938000

{*bis*-Li[B$_4$H$_8$]}$^+$_conf0_MeCN E = -111.538581294 Ha

5	2.221770000	0.478526000	-0.085279000
1	2.343650000	1.630183000	-0.341217000
1	1.267577000	-0.112067000	-0.767937000
1	0.325980000	-1.078331000	0.664665000
1	-0.137653000	0.913594000	0.464827000
5	0.690466000	0.047969000	0.441124000
5	2.173457000	-0.250540000	1.554764000
1	1.728880000	-0.288329000	2.653151000
1	3.413016000	0.216240000	1.736178000
1	2.696291000	-1.419177000	1.203937000
5	3.428827000	-0.474235000	0.557046000
1	4.528844000	-0.859101000	0.399860000
3	-1.657417000	-0.390271000	0.720087000

***bis*-Li[B$_4$H$_9$]_conf1_MeCN E = -112.275635636 Ha**

5	4.617679000	2.325851000	22.048066000
1	2.864869000	3.396819000	23.676754000
5	3.530266000	2.421832000	23.536305000
1	2.752637000	1.388737000	23.312582000
1	4.452347000	1.220842000	21.547273000
1	6.119409000	1.655010000	23.926368000
5	5.584538000	2.561575000	23.310808000
1	4.196814000	3.215602000	21.353570000
1	3.923532000	2.069047000	24.748521000
5	3.087072000	1.067679000	24.544754000
1	2.162208000	1.145832000	25.284681000
1	3.727212000	0.066600000	24.472330000
3	6.078332000	0.626629000	22.268621000
1	5.750878000	3.625491000	23.843850000

{*bis*-Li[B$_4$H$_8$]}$^+$_conf1_MeCN E = -111.510388866 Ha

5	4.994126000	0.621693000	22.380846000
1	2.889581000	3.327224000	22.902164000
5	3.300140000	2.409830000	23.541848000
1	2.432489000	1.417075000	23.402132000
1	4.960383000	-0.436093000	22.935314000
1	5.689559000	2.715142000	23.786871000
5	4.906265000	1.968422000	23.273370000
1	4.914631000	0.613471000	21.192223000
1	3.082233000	2.648326000	24.818006000
5	2.234420000	1.647440000	24.676246000
1	1.132181000	2.058790000	24.826062000
1	2.633692000	0.723648000	25.305622000
3	7.507322000	2.138337000	23.236639000

***lin-BBB*-Li[B$_4$H$_9$]_conf0_MeCN E = -112.275635636 Ha**

5	4.617679000	2.325851000	22.048066000
1	2.864869000	3.396819000	23.676754000
5	3.530266000	2.421832000	23.536305000
1	2.752637000	1.388737000	23.312582000
1	4.452347000	1.220842000	21.547273000
1	6.119409000	1.655010000	23.926368000
5	5.584538000	2.561575000	23.310808000
1	4.196814000	3.215602000	21.353570000
1	3.923532000	2.069047000	24.748521000
5	3.087072000	1.067679000	24.544754000
1	2.162208000	1.145832000	25.284681000
1	3.727212000	0.066600000	24.472330000
3	6.078332000	0.626629000	22.268621000
1	5.750878000	3.625491000	23.843850000

{*lin-BBB*-Li[B$_4$H$_8$]}$^+$_conf0_MeCN E = -111.510388866 Ha

5	4.994126000	0.621693000	22.380846000
1	2.889581000	3.327224000	22.902164000
5	3.300140000	2.409830000	23.541848000
1	2.432489000	1.417075000	23.402132000
1	4.960383000	-0.436093000	22.935314000
1	5.689559000	2.715142000	23.786871000

5	4.906265000	1.968422000	23.273370000
1	4.914631000	0.613471000	21.192223000
1	3.082233000	2.648326000	24.818006000
5	2.234420000	1.647440000	24.676246000
1	1.132181000	2.058790000	24.826062000
1	2.633692000	0.723648000	25.305622000
3	7.507322000	2.138337000	23.236639000

***lin-1-BBB*-Li[B_4H_9]_conf1_MeCN E = -112.263000179 Ha**

5	3.972868000	2.251108000	22.302927000
1	3.478345000	2.674874000	21.305790000
5	3.429932000	1.837214000	23.717615000
1	5.285054000	2.473356000	22.294640000
5	4.862420000	3.210919000	23.341232000
1	5.485570000	2.887117000	24.299421000
1	4.949380000	4.384773000	23.057553000
1	3.795406000	1.285376000	24.698824000
3	2.927720000	4.552554000	22.849720000
1	2.141242000	1.914142000	23.676655000
5	1.711466000	3.053869000	24.230407000
1	0.842992000	2.594293000	24.913706000
1	2.522483000	3.705585000	24.839729000
1	1.291313000	3.604378000	23.232389000

{*lin-1-BBB*-Li[B_4H_8]}$^+$_conf1_MeCN E = -111.551730638 Ha

5	4.649341000	2.542068000	22.625102000
1	3.554361000	2.153798000	21.986257000
5	3.236176000	1.837798000	23.265454000
1	5.589110000	2.285035000	21.970098000
5	4.174518000	3.004419000	24.099881000
1	4.916539000	3.214174000	24.991352000
1	4.521980000	3.781755000	22.997089000
1	3.119591000	0.675416000	23.431374000
3	2.016847000	4.949496000	22.039008000
1	1.990417000	2.352008000	23.214340000
5	2.432831000	3.082956000	24.230055000
1	1.872007000	2.661766000	25.191386000
1	2.099990000	4.183285000	23.879483000

***lin-3-BBB*-Li[B_4H_9]_conf1_MeCN E = -112.254230537 Ha**

5	4.545135000	1.704918000	22.111269000
1	2.192799000	2.415374000	22.721008000
5	3.285648000	2.145623000	23.101890000
1	3.632291000	0.922949000	22.751299000
1	5.579733000	2.774325000	24.181133000
5	5.321712000	2.753557000	23.005751000
1	4.388557000	1.501482000	20.953936000
1	5.730169000	3.703742000	22.399940000
1	3.465901000	2.393766000	24.323798000
5	3.092343000	1.317480000	25.112166000
1	4.118709000	0.694310000	25.254734000
1	2.167971000	0.677847000	24.707909000
1	2.849660000	2.063913000	26.019156000
3	5.841767000	0.803910000	24.172023000

{*lin-3-BBB*-Li[B_4H_8]}$^+$_conf1_MeCN E = -111.543096285 Ha

5	3.936507000	1.347490000	22.444012000
1	1.841875000	2.762872000	23.474884000
5	2.969784000	2.489634000	23.293985000
1	2.717145000	1.258356000	22.330671000
5	4.512069000	2.778508000	23.203919000
1	4.494612000	0.900230000	21.503301000
1	5.571414000	3.275458000	23.176865000
1	3.613370000	3.658071000	23.343749000
5	4.044428000	1.373689000	24.272890000
1	5.110255000	1.567072000	24.787655000
1	4.198327000	0.399743000	23.421767000
1	3.150366000	1.001501000	24.978698000
3	4.472510000	0.286247000	26.402485000

***commo*-Li[B_5H_{12}]_MeCN E = -138.955351626 Ha**

1	0.959081000	4.963173000	4.898041000
5	1.564823000	5.752043000	5.558442000
1	-0.488801000	7.032101000	5.525449000
5	0.464991000	7.036671000	6.239921000
1	-0.031340000	6.597273000	7.362166000
5	1.036603000	5.785231000	7.325526000
1	0.883455000	8.133267000	6.508223000
1	0.523486000	4.749281000	7.612146000
3	2.741066000	7.692972000	6.851016000
5	2.421759000	6.984978000	4.524343000
1	1.826756000	6.980844000	3.491963000
1	2.676732000	8.081556000	4.950122000
1	1.831234000	6.216100000	8.122339000
1	3.579779000	6.495418000	4.177541000
5	3.384141000	5.713947000	5.249495000
1	2.249632000	5.105873000	6.364883000
1	4.094316000	6.135488000	6.126357000
1	3.685498000	4.653473000	4.798626000

{ *commo*-Li[B_5H_{11}]}$^+$_MeCN E = -138.201001815 Ha

1	1.055297000	4.419215000	4.997834000
5	1.518060000	5.340448000	5.577798000
1	-0.508262000	6.835793000	5.380087000
5	0.576657000	6.777916000	5.837910000
1	0.587836000	7.182585000	7.057399000
5	0.912893000	5.860323000	7.267554000
1	1.342599000	7.758970000	5.331788000
1	-0.059514000	5.347441000	7.712453000
3	3.387185000	7.309736000	8.105661000
5	1.885731000	6.766329000	4.642186000
1	1.578093000	6.848984000	3.507484000
1	1.832323000	5.980758000	8.023570000
1	3.076565000	7.156455000	4.801823000
5	3.272602000	5.880657000	5.350897000
1	2.483424000	5.029949000	6.291082000
1	3.897200000	6.273087000	6.299317000
1	3.887788000	5.259487000	4.551633000

***lin-1*-Li[B_5H_{12}]_MeCN E = -138.944830882 Ha**

3	2.265897000	8.506977000	5.962898000
5	0.700732000	6.116040000	5.080372000
1	-0.985184000	7.706955000	5.804670000
5	0.039633000	7.284062000	6.250314000
1	-0.429681000	6.509177000	7.246381000
5	0.276245000	5.580849000	6.639927000
1	1.193713000	5.255893000	7.316597000
1	0.683925000	8.075791000	6.893600000
1	-0.637610000	4.653936000	6.641171000
1	1.886542000	5.641511000	4.929211000
5	2.810722000	6.586605000	4.885428000
1	3.588720000	5.899801000	4.282654000
1	2.532051000	7.588522000	4.270282000
1	3.149624000	6.781684000	6.032689000
1	0.207217000	6.413622000	4.043000000
5	-0.285309000	4.591789000	5.324656000
1	-1.349215000	4.770238000	4.810853000
1	0.220022000	3.516045000	5.214915000

{*lin-1*-Li[B_5H_{11}]}$^+$_MeCN E = -138.200950997 Ha

5	1.681296000	0.637327000	3.358161000
5	0.824996000	1.035903000	1.909833000
5	-0.138925000	0.263563000	3.132682000
5	-0.862418000	1.517517000	2.157020000
5	-1.170005000	1.583450000	3.922001000
1	2.474278000	-0.226398000	3.180566000
1	1.850713000	1.308177000	4.333425000
1	1.026725000	0.463683000	0.899294000
1	-0.538375000	-0.838069000	2.971362000
1	-1.626971000	1.259412000	1.298202000
1	-0.662832000	2.382277000	4.662553000
1	-2.266945000	1.246556000	4.214993000
1	1.900061000	1.571679000	2.368743000
1	0.169125000	2.184433000	1.663524000
1	-1.295157000	2.480428000	2.850928000
1	-0.128320000	0.599553000	4.327674000
3	1.276441000	3.219025000	4.769632000

***lin-BBB*-Li[B_5H_{12}]_MeCN E = -138.943973204 Ha**

5	4.458396000	1.811442000	22.903891000
1	3.988112000	2.614890000	25.305540000
5	3.417416000	2.375886000	24.276645000
1	2.760887000	3.301921000	23.852662000
1	4.004602000	2.146484000	21.818425000
1	6.518322000	2.153100000	24.338107000
5	5.831651000	2.616281000	23.480260000
1	2.500468000	1.513042000	24.566049000
5	3.305624000	0.713393000	23.778214000
1	4.837188000	0.659712000	22.802952000
1	2.506556000	0.285044000	22.995568000
1	3.764862000	-0.063229000	24.565590000
3	3.657538000	3.888960000	22.220683000
1	6.677980000	2.995586000	22.477517000
5	6.319548000	4.176220000	22.832889000
1	5.611926000	4.650319000	21.985715000
1	7.278871000	4.792702000	23.165789000
1	5.646093000	3.908687000	23.892670000

{*lin-BBB*-Li[B_5H_{11}]}$^+$_MeCN E = -138.174713418 Ha

5	4.751271000	1.751880000	23.262753000
1	3.966111000	2.500098000	25.436443000
5	3.541988000	2.458662000	24.325636000
1	3.220378000	3.516505000	23.845752000
1	4.946579000	0.897131000	22.458156000
1	6.861193000	2.251494000	24.325917000
5	6.065585000	2.731739000	23.581136000
1	2.478070000	1.688854000	24.400455000
5	3.147466000	1.022279000	23.460557000
1	2.531852000	1.116449000	22.432600000
1	3.279496000	-0.074139000	23.899443000
3	1.794140000	3.081482000	22.368782000
1	6.699659000	3.086573000	22.479865000
5	6.338981000	4.287690000	22.882081000
1	5.508102000	4.720413000	22.153876000
1	7.326263000	4.897754000	23.122174000
1	5.791717000	3.945864000	24.024587000

***lin-2*-Li[B_5H_{12}]_MeCN E = -138.934274753 Ha**

5	2.486793000	0.882534000	3.329351000
5	0.618063000	0.769163000	2.126277000
5	-0.716856000	0.308309000	3.256636000
5	-0.934946000	1.549581000	2.114455000
5	-1.450368000	1.923928000	3.728127000
1	3.373449000	1.341469000	2.668892000
1	2.384455000	1.417643000	4.414243000
1	1.045307000	0.117608000	1.235003000
1	-1.427943000	-0.619435000	2.995905000
1	-1.717606000	1.312335000	1.258109000
1	-0.765691000	2.518730000	4.525860000
1	-2.623653000	2.053531000	3.901110000
1	1.433205000	1.517205000	2.765731000
1	0.201243000	1.890306000	1.511197000
1	-1.198019000	2.716308000	2.660990000
1	-0.007675000	0.056350000	4.189298000
3	0.617150000	1.458659000	5.197765000
1	2.399737000	-0.306864000	3.352836000

{*lin-2*-Li[B_5H_{11}]}$^+$_MeCN E = -138.201028772 Ha

5	1.384722000	0.406152000	3.523078000
5	0.745439000	0.899003000	1.919971000
5	-0.400556000	0.247021000	3.061540000
5	-0.817777000	1.691050000	2.186037000
5	-1.392032000	1.645079000	3.814754000
1	1.711179000	1.198949000	4.365228000
1	0.920518000	0.391595000	0.870681000
1	-0.896968000	-0.791434000	2.788337000
1	-1.568143000	1.613465000	1.280031000
1	-0.867127000	2.032858000	4.816004000
1	-2.574542000	1.560006000	3.852672000
1	1.853249000	1.184437000	2.453389000
1	0.368090000	2.157499000	1.759479000
1	-1.099086000	2.682448000	2.956623000
1	0.080888000	0.261053000	4.206036000
3	0.892090000	2.990284000	5.210843000
1	2.003253000	-0.603573000	3.498191000

***bis-BH$_2$*-Li[B_5H_{12}]_MeCN E = -138.918278214 Ha**

5	4.534993000	2.457448000	22.341983000
1	2.669029000	3.246083000	23.921743000
5	3.313680000	2.315201000	23.533294000
1	2.406365000	1.313684000	23.333931000
1	4.680091000	1.397133000	21.715487000
1	6.566121000	2.060478000	23.881808000
5	5.952093000	2.864371000	23.210824000
1	4.250062000	3.325190000	21.536635000
1	3.734660000	1.756236000	24.721926000
5	2.955466000	0.800685000	24.374136000
1	2.141244000	0.657856000	25.229428000
1	3.590122000	-0.166521000	24.043739000
3	5.374731000	0.559440000	23.172782000
1	6.895429000	3.515190000	22.504044000
5	6.673608000	4.451987000	23.369826000
1	6.205309000	5.376479000	22.781636000
1	7.624135000	4.612339000	24.067749000
1	5.715941000	3.892135000	24.043734000

{*bis-BH$_2$*-Li[B_5H_{11}]}$^+$_MeCN E = -138.168737344 Ha

5	4.707730000	2.648671000	22.561359000
1	3.411754000	3.158043000	24.650447000
5	3.696062000	2.292068000	23.883848000
1	2.531317000	1.737309000	23.474993000
1	5.024347000	1.809604000	21.745402000
1	6.209075000	2.699548000	24.838317000
5	5.952675000	3.142331000	23.785268000
1	3.969186000	3.480684000	22.084867000
1	4.032466000	1.235852000	24.672837000
5	2.945775000	0.743192000	24.184583000
1	2.187091000	0.554273000	25.074999000
1	3.189186000	-0.181750000	23.460645000
3	4.835072000	0.115124000	22.427682000
1	7.029898000	3.325527000	23.027483000
5	5.964488000	3.932678000	22.493462000
1	6.236952000	4.676088000	21.635281000
1	5.735871000	4.453833000	23.715485000

***lin-bis*-Li[B_5H_{12}]_MeCN E = -138.896760443 Ha**

5	4.550952000	1.938723000	23.463054000
1	5.385954000	1.221474000	23.933646000
5	2.927064000	1.510052000	23.405716000
1	4.798464000	3.169184000	23.960543000
5	5.208581000	3.406904000	22.759415000
1	4.473572000	4.203397000	22.264006000
1	4.967681000	2.246686000	22.226460000
1	6.381979000	3.606274000	22.758613000
1	2.679230000	0.533213000	24.446784000
5	2.521872000	-0.038803000	23.290472000
1	1.436421000	-0.531711000	23.148426000
1	2.034228000	2.309179000	23.519304000
1	3.591705000	-0.684891000	22.996732000
5	3.579229000	-1.854252000	23.639565000
1	4.770100000	-1.982877000	23.588117000
1	2.984909000	-2.623021000	22.915820000
1	3.172305000	-1.859651000	24.778161000
3	1.405754000	-2.288322000	24.044065000

{*lin-bis*-Li[B_5H_{11}]}$^+$_MeCN E = -138.159668541 Ha

5	4.331403000	2.442655000	23.874000000
1	4.629736000	2.894408000	24.938707000

5	2.964073000	1.492162000	23.787362000
1	4.332746000	3.448491000	23.006352000
5	5.377929000	2.815552000	22.537451000
1	5.117315000	2.377438000	21.463611000
1	5.421510000	1.837499000	23.399160000
1	6.335025000	3.502441000	22.688798000
1	2.815398000	-0.556677000	25.109756000
5	2.992657000	-0.163699000	23.986996000
1	1.898134000	2.023365000	23.667309000
1	4.121404000	-0.709747000	23.516201000
5	3.272816000	-1.414011000	22.821755000
1	3.466502000	-1.203425000	21.673322000
1	2.164806000	-0.803969000	23.162200000
1	3.259303000	-2.554388000	23.187116000
3	2.932822000	-2.614828000	25.170376000

***lin-cyc*-Li[B_5H_{12}]_MeCN E = -138.887743093 Ha**

5	4.050797000	2.198802000	22.420588000
1	3.231571000	2.266284000	21.499866000
5	3.383736000	1.408159000	23.721254000
1	5.100929000	1.742113000	22.007419000
5	4.261081000	3.663726000	23.375317000
1	4.039957000	4.794541000	22.970910000
1	5.212155000	3.648899000	24.113956000
1	4.239500000	1.292798000	24.739689000
5	3.764473000	0.077585000	24.782717000
1	2.974921000	0.126089000	23.745194000
1	4.659394000	-0.645092000	24.486482000
1	3.144239000	-0.080564000	25.781829000
3	2.971065000	4.059027000	21.588674000
1	2.312545000	1.842184000	24.322511000
5	2.313363000	3.166494000	24.248777000
1	1.708892000	3.327245000	25.273439000
1	3.413441000	3.709991000	24.531735000
1	1.715938000	3.475476000	23.261395000

{*lin-cyc*-Li[B_5H_{11}]}$^+$_MeCN E = -138.183998926 Ha

5	4.006220000	2.315559000	22.646382000
1	3.531848000	2.133161000	21.555373000
5	3.464671000	1.342818000	23.949941000
1	5.185593000	2.149068000	22.744147000
5	4.178446000	4.019298000	23.355588000
1	3.811280000	4.967062000	22.711078000
1	5.203705000	4.136454000	23.938384000
1	4.409334000	0.857537000	24.683747000
5	3.562005000	-0.194201000	24.674816000
1	2.674385000	0.335773000	23.803607000
1	4.113771000	-1.058382000	24.090334000
1	3.070274000	-0.333189000	25.737652000
3	2.796202000	3.899693000	21.297850000
1	2.832365000	2.116697000	24.826898000
5	2.880788000	2.964405000	23.779333000
1	3.295072000	3.998615000	24.425892000
1	1.773144000	3.096143000	23.377290000

***1,1-BBB*-Li[B_5H_{12}]_MeCN E = -138.875412557 Ha**

5	3.968862000	2.155810000	22.252302000
1	3.522351000	2.480558000	21.205163000
5	3.397736000	1.865778000	23.682015000
1	5.272848000	2.425595000	22.284051000
5	4.812772000	3.151243000	23.316927000
1	5.447234000	2.859726000	24.277213000
1	4.892589000	4.311710000	22.990485000
1	3.809148000	1.549227000	24.808905000
5	3.956159000	0.165933000	24.740006000
1	2.986653000	-0.398694000	24.315688000
1	4.999503000	-0.078162000	24.202920000
1	4.009073000	0.200555000	25.944221000
3	2.936949000	4.767993000	23.154645000
1	2.128712000	1.901482000	23.680219000
5	1.720547000	3.069767000	24.230553000
1	0.816251000	2.589950000	24.848660000
1	2.495869000	3.694786000	24.910675000
1	1.366352000	3.645953000	23.224128000

{*1,1-BBB*-Li[B_5H_{11}]}$^+$_MeCN E = -138.197340220 Ha

5	4.180447000	1.454584000	22.946456000
1	3.634829000	1.589127000	21.909305000
5	3.243246000	1.904158000	24.344624000
1	5.207240000	2.308928000	22.942522000
5	4.608248000	2.921989000	23.946168000
1	5.356359000	2.943709000	24.858843000
1	4.419032000	4.018756000	23.427010000
1	3.209971000	1.735646000	25.515327000
5	3.832152000	0.158327000	24.016764000
1	3.055837000	-0.626933000	23.582342000
1	4.797865000	0.340988000	23.044520000
1	4.500643000	-0.121800000	24.955524000
3	1.488522000	5.363912000	23.063871000
1	2.134946000	2.287543000	23.893369000
5	2.917772000	3.510323000	23.697594000
1	2.546335000	4.297775000	24.518439000
1	2.589914000	3.682229000	22.557437000

***lin-BB*-Li[B_5H_{12}]_MeCN E = -138.846402650 Ha**

5	4.443639000	2.626841000	21.866791000
1	2.362434000	3.244444000	23.172718000
5	2.777414000	2.132444000	23.078402000
1	1.957088000	1.302282000	22.829514000
1	3.547325000	1.740603000	22.084026000
5	5.498259000	3.233389000	22.787704000
1	4.416578000	2.681244000	20.675086000
1	3.389271000	2.037116000	24.196880000
5	3.783851000	0.783889000	24.507384000
1	3.926942000	0.032725000	23.576475000
1	2.979659000	0.416160000	25.310481000
1	4.833529000	1.161738000	24.981928000
3	5.762451000	0.769441000	23.116016000
1	6.512891000	3.968079000	22.425236000
5	6.674474000	3.986079000	23.737843000
1	6.581689000	5.119751000	24.085786000
1	7.621056000	3.348354000	24.072373000
1	5.540087000	3.393305000	24.080548000

{*lin-BB*-Li[B_5H_{11}]}$^+$_MeCN E = -138.157999783 Ha

5	4.887562000	2.275933000	21.355494000
1	2.658773000	3.582114000	23.780576000
5	3.495381000	2.744466000	23.678254000
1	2.851126000	1.653351000	23.243944000
1	5.082699000	1.097819000	21.196697000
5	4.955785000	3.004658000	22.834317000
1	4.480714000	2.813064000	20.374206000
1	3.746141000	2.276060000	24.898607000
5	3.201377000	1.193898000	24.409708000
1	4.053213000	0.358379000	24.298577000
1	2.226065000	0.928564000	25.027283000
3	5.335534000	-0.242650000	22.720516000
1	5.278758000	4.287568000	22.807002000
5	6.231612000	3.882203000	23.613350000
1	6.039036000	4.299544000	24.709473000
1	7.274682000	4.066726000	23.076081000
1	5.976649000	2.594447000	23.579179000

B_2H_2_MeCN E = -50.7115834790 Ha

5	0.761432000	-0.000289000	-0.000015000
5	-0.761432000	0.000212000	-0.000046000
1	-1.929791000	-0.000303000	0.000182000
1	1.929793000	0.000685000	0.000121000

B_2H^+_MeCN E = -49.7720084214 Ha

5	0.000000000	0.000000000	0.581202000
5	0.000000000	0.000000000	-0.930111000
1	0.000000000	0.000000000	1.744545000

B_3H_5_conf0_MeCN E = -77.4600558437 Ha

5	4.567786000	1.664778000	22.321974000
5	3.737043000	1.788421000	23.656462000
1	3.510391000	0.861926000	22.878280000
1	5.209998000	3.788940000	23.935268000
5	4.876162000	2.857331000	23.305669000
1	4.794257000	1.093060000	21.318308000
1	5.517586000	2.742568000	22.260705000
1	3.044750000	1.758395000	24.602971000

$B_3H_4^+$_conf0_MeCN E = -76.6147264255 Ha

5	-0.125084000	5.579068000	5.991059000
1	-0.575505000	8.108425000	6.606915000
5	-0.271925000	7.010062000	6.387032000
1	-1.202905000	6.134940000	5.723118000
5	1.118826000	6.044576000	6.671345000
1	2.155013000	6.208630000	7.167485000
1	0.793231000	4.754790000	6.125709000

B_3H_5_conf1_MeCN E = -77.4281949133 Ha

5	4.520245000	2.072672000	22.336458000
1	2.268091000	2.688081000	23.147401000
5	3.226484000	1.998639000	23.321561000
1	6.780492000	2.690023000	23.126939000
5	5.817963000	3.039174000	22.513779000
1	4.516476000	1.343019000	21.381310000
1	5.857462000	4.129757000	22.030682000
1	3.185927000	1.248967000	24.249340000

$B_3H_4^+$_conf1_MeCN E = -76.5958090969 Ha

5	4.413546000	2.046669000	22.252810000
1	2.143509000	2.501970000	23.074293000
5	3.181653000	1.975562000	23.318967000
5	5.521736000	3.263542000	22.283705000
1	4.533604000	1.220270000	21.406401000
1	6.303463000	4.133568000	22.303333000
1	3.295137000	1.378730000	24.341022000

***arachno*-B_4H_8_conf0_MeCN E = -104.116408480 Ha**

5	1.148677000	5.741231000	5.237547000
5	0.385320000	5.238908000	6.682893000
1	0.327549000	4.431174000	7.540149000
1	-0.888305000	5.729659000	6.383126000
1	1.989959000	5.230671000	4.584515000
1	0.049787000	6.070304000	4.501827000
5	-0.450990000	5.152798000	5.304641000
1	-1.217243000	4.489570000	4.709611000
1	1.502905000	7.018759000	5.456947000
5	1.428604000	6.637734000	6.727272000
1	0.739025000	7.519448000	7.146764000
1	2.554607000	6.560157000	7.111777000

***arachno*-$B_4H_7^+$_conf0_MeCN E = -103.305001843 Ha**

5	1.169552000	5.590198000	5.121516000
5	0.483707000	5.090303000	6.656668000
1	0.480318000	4.198173000	7.421801000
1	-0.693424000	5.695627000	6.581545000
1	1.959418000	5.109275000	4.398352000
1	0.137205000	6.099822000	4.490063000
5	-0.464527000	5.210279000	5.338430000
1	-1.420033000	4.824334000	4.787110000
1	1.768040000	6.812863000	5.438223000
5	1.476565000	6.439729000	6.638574000
1	1.934047000	7.230357000	7.368031000

***disph*-B_4H_8_MeCN E = -104.104903539 Ha**

5	3.783974000	1.304255000	22.454652000
1	1.846133000	2.756116000	23.588544000
5	2.992496000	2.588647000	23.433193000
1	2.736285000	0.940149000	22.022475000
5	4.496071000	2.775355000	23.204443000
1	4.714941000	1.188367000	21.721617000
1	5.562472000	3.218233000	23.022647000
1	3.610397000	3.708684000	23.276376000
5	4.050539000	1.418491000	24.297091000
1	5.151839000	1.373663000	24.747146000
1	4.042169000	0.411724000	23.416705000
1	3.172836000	1.128940000	25.047508000

***disph*-$B_4H_7^+$_MeCN E = -103.293327816 Ha**

5	4.204739000	1.361612000	22.596735000
1	1.864966000	2.710536000	23.568794000
5	3.006238000	2.435351000	23.566612000
5	4.523498000	2.882168000	23.162142000
1	4.630304000	1.059427000	21.550716000
1	5.258296000	3.552724000	22.548170000
1	3.652568000	3.593588000	23.768654000
5	4.295581000	1.351700000	24.226358000
1	4.762850000	0.864716000	25.187009000
1	4.117648000	0.342063000	23.364176000
1	3.107180000	1.718591000	24.670557000

***arachno*-B_4H_8_conf1_MeCN E = -104.098682233 Ha**

5	4.423291000	1.409596000	22.588561000
1	5.042271000	0.837093000	21.754226000
5	2.858339000	1.888451000	23.296730000
1	5.147789000	1.623177000	23.683774000
5	4.301663000	2.681201000	23.580912000
1	4.792134000	3.629434000	24.076995000
1	4.627225000	2.708792000	22.268742000
1	2.525817000	0.933534000	24.115223000
5	3.037142000	0.229074000	23.062876000
1	2.217189000	-0.330252000	22.409205000
1	1.940229000	2.377394000	22.721930000
1	3.865989000	-0.439559000	23.612511000

***arachno*-$B_4H_7^+$_conf1_MeCN E = -103.307420986 Ha**

5	5.066647000	1.168700000	22.571215000
1	6.042180000	0.598237000	22.304539000
5	2.393201000	1.846681000	23.356602000
1	5.063942000	2.169122000	23.519359000
5	3.943740000	2.111147000	22.858003000
1	4.498377000	2.053430000	21.681673000
1	2.718630000	0.926407000	24.298880000
5	3.280684000	0.511890000	23.165947000
1	2.162246000	0.812643000	22.507803000
1	1.387248000	2.375968000	23.635388000
1	3.430050000	-0.655724000	23.195281000

***bis*-B_4H_8_MeCN E = -104.074481343 Ha**

5	4.539387000	2.047056000	22.323217000
1	2.281796000	2.723363000	22.964689000
5	3.196825000	2.042322000	23.319030000
1	2.723640000	0.824546000	23.557792000
1	6.774928000	2.708338000	23.126648000
5	5.810843000	3.042321000	22.503320000
1	4.520267000	1.304231000	21.380993000
1	5.841389000	4.144693000	22.044339000
1	3.446837000	2.356465000	24.588372000
5	2.966927000	1.161760000	24.797963000
1	3.835973000	0.466700000	25.213824000
1	1.938210000	1.253635000	25.384294000

***bis*-$B_4H_7^+$_MeCN E = -103.283962892 Ha**

5	4.656891000	2.037955000	22.277239000
1	2.348400000	2.500004000	23.476915000
5	3.479541000	1.811918000	23.435047000
1	2.808551000	0.662621000	23.651853000
1	6.907753000	2.828598000	22.907235000
5	5.862446000	3.120982000	22.414907000
1	4.577550000	1.358429000	21.302560000
1	5.745726000	4.236471000	22.011960000
1	3.649211000	1.974108000	24.762619000
5	2.419004000	1.608183000	24.477929000

1	1.585976000	1.469462000	25.272394000

***sq*-B_4H_8_MeCN E = -104.079044407 Ha**

5	5.259178000	1.707259000	23.336362000
5	2.816143000	1.675312000	23.266266000
1	4.881444000	2.823597000	23.973297000
5	4.025245000	2.859036000	22.943275000
1	4.025215000	3.998170000	22.593502000
1	4.939857000	2.269067000	22.163149000
1	3.135688000	1.113114000	24.439286000
5	4.049544000	0.523500000	23.658549000
1	3.193270000	0.559529000	22.628757000
1	1.625110000	1.650941000	23.239360000
1	4.050272000	-0.616203000	24.006477000
1	6.450417000	1.732346000	23.364239000

***sq*-$B_4H_7^+$_MeCN E = -103.307400134 Ha**

5	5.091885000	1.845834000	23.286619000
5	3.348764000	1.611369000	23.302251000
1	4.973028000	3.017908000	23.908523000
5	4.073186000	3.034563000	22.891275000
1	3.866887000	4.134498000	22.548759000
1	5.029951000	2.470564000	22.111881000
1	3.063862000	1.088274000	24.460245000
5	3.913678000	0.295167000	23.725089000
1	3.127013000	0.525072000	22.619926000
1	4.166140000	-0.786099000	24.064879000
1	6.171880000	1.407578000	23.453712000

***lin-BBB*-B_4H_8_MeCN E = -104.071572706 Ha**

5	4.775836000	1.655097000	22.424577000
5	3.824390000	1.859638000	23.868843000
1	3.586045000	1.122159000	22.816205000
1	5.708838000	3.741079000	23.747271000
5	5.008383000	2.852383000	23.424748000
1	5.020464000	0.960630000	21.499210000
1	4.701823000	2.915305000	22.102195000
1	4.047752000	1.062912000	24.914661000
5	2.771990000	1.255862000	25.103516000
1	2.197697000	0.233338000	24.924414000
1	2.513431000	2.107795000	24.165769000
1	2.674912000	1.853171000	26.124528000

***lin-BBB*-$B_4H_7^+$_MeCN E = -103.301265097 Ha**

5	4.858358000	1.630184000	22.403854000
5	4.014644000	1.208454000	23.708286000
1	3.748263000	0.835950000	22.493992000
1	5.002333000	3.665577000	24.131496000
5	4.580064000	2.692439000	23.638117000
1	5.520189000	1.268962000	21.512441000
1	4.515981000	2.889213000	22.297590000
1	3.529262000	0.558099000	24.619551000
5	3.183127000	2.113236000	24.850967000
1	2.025765000	2.189502000	24.627886000
1	3.655876000	2.334417000	25.907343000

***arachno*-B_5H_{11}_MeCN E = -130.764222891 Ha**

5	1.748326000	0.872717000	3.248088000
5	0.768655000	0.898009000	1.817996000
5	-0.064235000	0.397827000	3.252437000
5	-0.909816000	1.391359000	2.098940000
5	-1.092891000	1.825462000	3.834162000
1	2.554412000	0.000403000	3.190503000
1	1.936731000	1.771343000	4.003727000
1	0.916164000	0.123392000	0.939468000
1	-0.424993000	-0.722889000	3.380201000
1	-1.738085000	0.945725000	1.386487000
1	-0.561918000	2.763823000	4.348702000
1	-2.158769000	1.501101000	4.244090000
1	1.839301000	1.562904000	2.058550000
1	0.047567000	1.948483000	1.378928000
1	-1.292196000	2.481458000	2.614365000

1	0.047611000	1.020451000	4.321711000

***arachno*-$B_5H_{10}^+$_MeCN E = -129.959629281 Ha**

5	1.881251000	0.596483000	3.182269000
5	0.811585000	1.163848000	1.960710000
5	0.008918000	0.552438000	3.404786000
5	-0.826697000	1.781157000	2.348879000
5	-1.106649000	1.607985000	4.013601000
1	2.397756000	-0.368489000	2.742021000
1	2.238224000	1.098730000	4.189286000
1	0.700758000	0.582717000	0.943688000
1	-0.241528000	-0.543215000	3.770006000
1	-1.125937000	2.700400000	3.249635000
1	-1.997740000	1.604921000	4.766020000
1	1.967532000	1.614080000	2.229362000
1	0.243598000	2.386323000	1.891013000
1	-1.675229000	1.716335000	1.543086000
1	0.078107000	1.342131000	4.497507000

***lin-1*-B_5H_{11}_conf0_MeCN E = -130.736549045 Ha**

5	4.511240000	1.375163000	22.708919000
1	5.133590000	0.579824000	22.095984000
5	2.906770000	1.908408000	23.367200000
1	5.240367000	1.857510000	23.703278000
5	4.312356000	2.802966000	23.425811000
1	4.722209000	3.874424000	23.662543000
1	4.569029000	2.514919000	22.075133000
1	2.623646000	1.111261000	24.407989000
5	3.124979000	0.304248000	23.478848000
1	2.298175000	-0.519887000	22.952955000
1	1.942630000	2.141151000	22.725101000
1	3.924547000	-0.434534000	24.159334000
5	3.059895000	-1.375955000	23.680872000
1	3.667059000	-2.032928000	22.915305000
1	2.425126000	-1.783218000	24.584481000

***lin-1*-$B_5H_{10}^+$_conf0_MeCN E = -129.962460154 Ha**

5	4.409773000	1.359641000	22.683087000
1	4.940214000	0.598008000	21.951909000
5	2.973317000	1.774063000	23.711117000
1	5.309077000	1.963477000	23.444811000
5	4.246974000	2.799415000	23.386660000
1	4.587031000	3.916270000	23.481032000
1	4.184698000	2.464396000	22.024499000
1	3.040729000	1.001563000	24.805923000
5	3.381407000	0.205083000	23.800168000
1	1.861004000	1.878932000	23.326322000
1	4.397868000	-0.418202000	24.277268000
5	3.552178000	-1.462465000	24.045042000
1	3.207146000	-1.893150000	25.084438000
1	4.021977000	-2.086160000	23.163533000
1	2.545632000	-0.720238000	23.512945000

***commo*-B_5H_{11}_conf0_MeCN E = -130.733269276 Ha**

5	1.971552000	6.066750000	5.982603000
1	-0.261862000	6.635236000	5.358669000
5	0.493606000	6.962851000	6.222400000
1	-0.049605000	6.587806000	7.345863000
5	0.964835000	5.739882000	7.410772000
1	0.712348000	8.122125000	6.377816000
1	0.482433000	4.653536000	7.393389000
5	2.587009000	7.041448000	4.671245000
1	1.786457000	7.146824000	3.797611000
1	3.039131000	8.034498000	5.154463000
1	1.619161000	6.058670000	8.355613000
1	3.586462000	6.415767000	4.115045000
5	3.381188000	5.508285000	5.054411000
1	2.417524000	5.099632000	6.545829000
1	4.377719000	5.478301000	5.701601000
1	3.027584000	4.511002000	4.503390000

***commo*-$B_5H_{10}^+$_conf0_MeCN E = -129.938379161 Ha**

5	1.969104000	6.268054000	5.935558000
1	-0.541157000	5.935815000	5.606441000
5	0.197580000	6.690057000	6.141412000
1	-0.006811000	6.762387000	7.441306000
5	1.076658000	6.045821000	7.484213000
1	0.295474000	7.812617000	5.754723000
1	0.850448000	4.915494000	7.752260000
5	2.778347000	7.048989000	4.730865000
1	2.780465000	7.991326000	4.044370000
1	2.793583000	7.323989000	6.018676000
1	1.878552000	6.656449000	8.127801000
1	3.723499000	6.167034000	4.391030000
5	2.694109000	5.401256000	4.726939000
1	2.641304000	5.136248000	6.045326000
1	2.626668000	4.428776000	4.088200000

***commo*-B_5H_{11}_conf1_MeCN E = -130.735128900 Ha**

5	4.516699000	2.167930000	22.809162000
1	3.819367000	3.399659000	24.965970000
5	3.356021000	2.921695000	23.968195000
1	2.653932000	3.628326000	23.309227000
1	4.268018000	2.192011000	21.643829000
1	7.169206000	1.357145000	23.311818000
5	6.310601000	2.153011000	23.257344000
1	2.517941000	1.995429000	24.396188000
5	3.402107000	1.174976000	23.870961000
1	5.114892000	1.110195000	23.083106000
1	2.740439000	0.506706000	23.136015000
1	3.923065000	0.622354000	24.795749000
1	6.663239000	3.199638000	22.565136000
5	5.549145000	3.495861000	23.314500000
1	5.432178000	4.656711000	23.382261000
1	6.179496000	2.888819000	24.349614000

***commo*-$B_5H_{10}^+$_conf1_MeCN E = -129.955491048 Ha**

5	4.587448000	2.084682000	22.946866000
1	3.666280000	2.191819000	25.178095000
5	3.443569000	2.932310000	24.097409000
1	3.260359000	4.070005000	24.281324000
1	4.302769000	1.753310000	21.843162000
1	7.322059000	1.731119000	23.698061000
5	6.375101000	2.338169000	23.366241000
1	2.356636000	2.211735000	23.850150000
5	3.393425000	1.420139000	24.118861000
1	5.390470000	1.239060000	23.444204000
1	3.143515000	0.300141000	24.320810000
1	6.614222000	3.044454000	22.290525000
5	5.511987000	3.556057000	22.909524000
1	5.321941000	4.662761000	22.591591000
1	6.186124000	3.428000000	24.086238000

***commo*-B_5H_{11}_conf2_MeCN E = -130.734955123 Ha**

1	0.927313000	5.857283000	4.442042000
5	1.598339000	6.282328000	5.323490000
1	-0.693101000	5.609869000	6.100511000
5	0.006601000	6.570004000	6.160163000
1	0.213411000	6.815762000	7.444587000
5	1.437128000	6.500120000	7.119798000
1	-0.389563000	7.593022000	5.692183000
1	1.711027000	5.457874000	7.636223000
5	3.168628000	6.782336000	5.161195000
1	2.117139000	7.428943000	4.761035000
1	3.923982000	7.431903000	5.781074000
1	2.071751000	7.457807000	7.438085000
1	3.710932000	6.117848000	4.173888000
5	3.121892000	5.136775000	4.927696000
1	3.941641000	4.730697000	5.690124000
1	2.630482000	4.387484000	4.159368000

***commo*-$B_5H_{10}^+$_conf2_MeCN E = -129.946959015 Ha**

1	0.928300000	5.649624000	4.569898000
5	1.647479000	6.207673000	5.325427000
1	-0.839005000	6.782020000	6.070127000
5	0.251958000	6.604021000	6.430664000
1	0.487362000	5.667800000	7.337508000
5	1.561890000	6.442713000	7.155516000
1	2.438440000	6.332047000	7.911903000
5	3.200611000	6.674633000	5.176672000
1	2.093423000	7.311643000	4.739645000
1	3.889689000	7.355852000	5.835299000
1	0.800982000	7.517174000	7.235356000
1	3.792841000	6.055657000	4.210760000
5	3.108045000	5.030386000	4.814492000
1	3.837382000	4.521872000	5.603299000
1	2.687769000	4.413917000	3.902713000

***commo*-B_5H_{11}_conf3_MeCN E = -130.733035128 Ha**

1	1.678718000	4.823507000	5.103482000
5	1.772922000	5.907122000	5.886943000
1	-0.273448000	7.203840000	5.555112000
5	0.588443000	7.108045000	6.370853000
1	-0.037984000	6.577162000	7.437467000
5	0.752167000	5.574496000	7.277547000
1	1.084898000	8.100345000	6.801155000
1	0.040805000	4.647595000	7.033359000
5	2.341179000	7.004567000	4.615334000
1	1.591080000	7.154439000	3.703988000
1	3.024693000	7.904923000	4.987102000
1	1.419204000	5.532990000	8.266843000
1	3.257364000	6.191461000	4.049954000
5	2.918102000	5.310293000	4.977558000
1	2.965923000	5.524147000	6.311626000
1	3.629872000	4.386816000	4.794618000

***commo*-$B_5H_{10}^+$_conf3_MeCN E = -129.963900579 Ha**

1	1.582407000	5.145666000	4.579454000
5	1.815379000	5.606418000	5.820920000
1	-0.405235000	6.477415000	5.306515000
5	0.481060000	6.679925000	6.052284000
1	0.149489000	6.873661000	7.241603000
5	0.962971000	5.716985000	7.444202000
1	0.960891000	7.875179000	5.737449000
1	0.227555000	4.837443000	7.718405000
5	2.128823000	7.313039000	5.475737000
1	2.122315000	7.275147000	4.134651000
1	2.912475000	8.069139000	5.915648000
1	1.787922000	6.125600000	8.188575000
1	3.475966000	5.722305000	3.803488000
5	2.567330000	6.055097000	4.456363000
1	2.354718000	4.791913000	6.503029000

***lin-BBB*-B_5H_{11}_conf0_MeCN E = -130.723409354 Ha**

5	4.649753000	2.719617000	22.538719000
1	3.393285000	3.128197000	24.728009000
5	3.709024000	2.318035000	23.909425000
1	2.567348000	1.740125000	23.474505000
1	4.880683000	1.785309000	21.815529000
1	6.071725000	2.786864000	24.892078000
5	5.851079000	3.170936000	23.805241000
1	3.888421000	3.563124000	22.118225000
1	4.119003000	1.225920000	24.587650000
5	3.008364000	0.730967000	24.149771000
1	2.283437000	0.537099000	25.072673000
1	3.263353000	-0.147309000	23.388090000
1	6.983034000	3.223558000	23.084596000
5	6.001819000	3.875903000	22.467250000
1	6.380231000	4.562002000	21.599229000
1	5.773318000	4.487606000	23.658286000

***lin-BBB*-$B_5H_{10}^+$_conf0_MeCN E = -129.955628485 Ha**

5	4.278463000	2.933092000	22.873214000
1	4.008998000	2.241040000	25.506255000
5	3.857124000	1.982262000	24.378696000
1	2.626907000	1.623069000	24.037759000
1	4.991716000	2.356107000	22.001220000
1	5.378964000	4.480441000	24.799865000
5	5.425495000	3.803767000	23.850124000
1	3.538263000	3.692471000	22.339652000
1	4.296824000	0.790270000	23.988385000
5	3.489333000	1.326500000	23.065874000
1	3.087478000	0.625816000	22.226207000
1	6.442618000	2.898150000	23.864160000
5	6.101385000	3.139305000	22.611882000
1	6.938709000	2.849310000	21.843970000
1	6.078165000	4.429254000	22.829338000

***bis-BH*-B_5H_{11}_MeCN E = -130.720923528 Ha**

5	4.315194000	2.440212000	23.862617000
1	4.604961000	2.929469000	24.913445000
5	2.954648000	1.478426000	23.790696000
1	4.326701000	3.417153000	22.959007000
5	5.381745000	2.776113000	22.529641000
1	5.140730000	2.307314000	21.463603000
1	5.414255000	1.822846000	23.416004000
1	6.334619000	3.471021000	22.675063000
1	2.814975000	-0.581369000	25.144366000
5	2.987815000	-0.170749000	24.035808000
1	1.889736000	2.009494000	23.642794000
1	4.113826000	-0.740100000	23.592968000
5	3.286661000	-1.362623000	22.804167000
1	3.471080000	-1.003990000	21.685402000
1	2.166698000	-0.844352000	23.234851000
1	3.297112000	-2.520770000	23.069663000

***bis-BH*-$B_5H_{10}^+$_MeCN E = -129.941346488 Ha**

5	4.409773000	1.359641000	22.683087000
1	4.940214000	0.598008000	21.951909000
5	2.973317000	1.774063000	23.711117000
1	5.309077000	1.963477000	23.444811000
5	4.246974000	2.799415000	23.386660000
1	4.587031000	3.916270000	23.481032000
1	4.184698000	2.464396000	22.024499000
1	3.040729000	1.001563000	24.805923000
5	3.381407000	0.205083000	23.800168000
1	1.861004000	1.878932000	23.326322000
1	4.397868000	-0.418202000	24.277268000
5	3.552178000	-1.462465000	24.045042000
1	3.207146000	-1.893150000	25.084438000
1	4.021977000	-2.086160000	23.163533000
1	2.545632000	-0.720238000	23.512945000

***i-bis*-$B_4H_8(BH_3)$_MeCN E = -130.719355925 Ha**

5	4.913023000	2.550384000	21.106509000
1	2.412002000	3.161126000	23.213392000
5	3.413851000	2.583507000	23.497364000
1	3.143649000	1.287703000	23.449231000
1	5.219937000	1.467600000	20.706292000
5	4.888761000	2.918993000	22.720474000
1	4.518305000	3.316544000	20.279460000
1	3.547067000	2.657855000	24.819549000
5	3.293120000	1.378662000	24.748953000
1	4.272501000	0.781321000	25.062517000
1	2.251894000	1.149934000	25.272316000
1	5.234931000	4.179083000	22.972941000
5	6.189104000	3.598725000	23.657011000
1	6.016183000	3.807165000	24.816713000
1	7.233943000	3.864160000	23.155242000
1	5.891301000	2.356032000	23.404782000

***i-bis*-$B_4H_8(BH_2)^+$_MeCN E = -129.944862992 Ha**

5	4.305787000	2.745661000	21.647043000
1	2.879704000	2.396614000	21.736735000
5	3.244398000	2.394828000	22.939158000
1	3.029636000	1.316803000	23.636101000
1	4.714385000	1.821227000	21.030828000
5	4.823343000	2.731420000	23.400340000
1	4.234109000	3.834256000	21.187621000
1	2.602679000	3.158628000	23.774328000
5	3.497641000	2.336136000	24.465134000
1	3.224901000	2.187280000	25.587847000
1	5.343657000	3.876465000	23.677208000
5	6.458834000	3.104690000	23.683285000
1	6.870110000	3.118113000	24.789415000
1	7.124031000	3.335960000	22.736503000
1	5.813857000	1.919390000	23.528681000

***lin-1*-B_5H_{11}_conf1_MeCN E = -130.704799304 Ha**

5	4.426365000	1.423737000	22.584083000
1	5.030148000	0.804453000	21.774821000
5	2.863348000	1.886129000	23.311570000
1	5.158822000	1.614862000	23.677385000
5	4.307118000	2.670650000	23.600517000
1	4.797269000	3.621797000	24.087027000
1	4.621800000	2.697844000	22.261583000
1	2.497710000	0.939479000	24.124817000
5	2.993111000	0.229594000	23.073654000
1	2.220285000	-0.353105000	22.400547000
1	1.953611000	2.354312000	22.711619000
1	3.960586000	-0.373678000	23.591407000
5	3.531179000	-1.490130000	24.371643000
1	4.417693000	-1.295826000	25.155551000
1	3.679235000	-2.379560000	23.586857000
1	2.421031000	-1.323032000	24.786222000

***lin-1*-$B_5H_{10}^+$_conf1_MeCN E = -129.941346488 Ha**

5	4.409773000	1.359641000	22.683087000
1	4.940214000	0.598008000	21.951909000
5	2.973317000	1.774063000	23.711117000
1	5.309077000	1.963477000	23.444811000
5	4.246974000	2.799415000	23.386660000
1	4.587031000	3.916270000	23.481032000
1	4.184698000	2.464396000	22.024499000
1	3.040729000	1.001563000	24.805923000
5	3.381407000	0.205083000	23.800168000
1	1.861004000	1.878932000	23.326322000
1	4.397868000	-0.418202000	24.277268000
5	3.552178000	-1.462465000	24.045042000
1	3.207146000	-1.893150000	25.084438000
1	4.021977000	-2.086160000	23.163533000
1	2.545632000	-0.720238000	23.512945000

***bis-BH$_2$*-B_5H_{11}_MeCN E = -130.698438386 Ha**

5	4.516775000	2.426707000	22.420829000
1	2.846733000	3.143808000	24.248318000
5	3.377711000	2.233244000	23.676390000
1	2.376341000	1.397576000	23.317485000
1	4.903383000	1.360853000	21.965749000
1	6.632214000	2.859581000	24.061927000
5	5.809811000	3.250482000	23.021332000
1	4.036971000	3.122558000	21.537558000
1	3.826328000	1.407908000	24.649848000
5	2.823131000	0.678402000	24.291654000
1	2.018882000	0.627737000	25.169466000
1	3.252719000	-0.334451000	23.830072000
1	6.965331000	3.662339000	22.370741000
5	6.990618000	4.017464000	23.610271000
1	7.884865000	4.605469000	24.066222000
1	5.786760000	4.497841000	23.606062000

***bis-BH$_2$*-$B_5H_{10}^+$_MeCN E = -129.939066112 Ha**

5	5.333395000	2.220981000	21.867680000
1	3.535831000	2.892043000	25.581228000
5	3.729267000	2.321515000	24.560517000
1	2.600737000	2.188956000	23.919338000
1	4.559109000	1.333035000	21.908192000
1	6.137376000	3.107676000	23.959378000
5	4.893857000	2.913081000	23.511879000
1	6.020373000	2.337571000	20.916128000
1	3.972557000	1.064418000	24.810874000
5	2.786450000	0.908188000	24.227689000
1	1.988369000	0.571817000	25.033457000
1	2.945590000	0.306238000	23.219834000
1	6.827229000	4.338914000	22.351060000
5	5.985972000	3.648671000	22.760046000
1	4.847596000	4.198947000	23.150403000

***lin-BB*-B_5H_{11}_MeCN E = -130.681068004 Ha**

5	4.088893000	2.769937000	22.036531000
1	2.971177000	3.073278000	22.690209000
5	2.958812000	1.775490000	22.897913000
1	1.961583000	1.325586000	22.463715000
1	3.998081000	1.445130000	22.204124000
5	5.458147000	3.358220000	22.749333000
1	3.852007000	2.954534000	20.888448000
1	3.095688000	1.851998000	24.149089000
5	3.643133000	0.657581000	24.542143000
1	3.541308000	-0.181440000	23.689037000
1	2.887943000	0.520119000	25.460926000
1	4.759962000	1.010277000	24.793207000
1	5.903575000	4.587993000	22.479770000
5	6.658786000	3.934436000	23.367022000
1	7.540341000	4.491184000	23.894283000
1	5.532797000	3.699035000	24.043119000

***lin-BB*-$B_5H_{10}^+$_MeCN E = -129.907084287 Ha**

5	4.197771000	2.847528000	22.265438000
1	3.227858000	2.891253000	23.278198000
5	3.416138000	1.648563000	23.185904000
1	2.359935000	1.031791000	22.827644000
1	4.364948000	1.465516000	22.373358000
5	5.580051000	3.610689000	22.694315000
1	3.609027000	3.025008000	21.257282000
1	3.731815000	1.168941000	24.324692000
5	2.601819000	0.441105000	24.042789000
1	2.934987000	-0.668320000	23.841582000
1	1.758518000	0.791298000	24.783447000
1	5.777748000	4.882563000	22.401707000
5	6.832686000	4.302622000	23.008141000
1	7.743299000	4.954011000	23.336137000
1	5.955671000	3.870512000	23.935029000

***1,2-BBB*-B_5H_{11}_conf1_MeCN E = -130.673977162 Ha**

5	4.600558000	2.541792000	21.526241000
5	3.731774000	2.271953000	22.949359000
1	2.553807000	2.360452000	22.888373000
1	4.186439000	1.386302000	22.003541000
5	5.402920000	3.260999000	22.631139000
1	4.062628000	2.807727000	20.511739000
1	4.275610000	2.069058000	24.042233000
5	3.842346000	0.809637000	24.590120000
1	3.423597000	0.051953000	23.765240000
1	3.054231000	1.208286000	25.395331000
1	4.961188000	0.604085000	24.973321000
1	5.603577000	4.539378000	22.698447000
5	6.240667000	4.131486000	23.802723000
1	5.631981000	4.401873000	24.780359000
1	7.374285000	4.434931000	23.656809000
1	6.214461000	2.812928000	23.526394000

***1,2-BBB*-$B_5H_{10}^+$_conf1_MeCN E = -129.927800025 Ha**

5	4.387683000	2.858241000	22.267632000
5	3.787936000	1.771266000	23.208195000
1	2.762139000	1.010651000	23.008640000
1	4.771942000	1.452662000	22.400938000
5	5.693458000	3.739752000	22.765526000
1	3.972466000	2.987674000	21.161263000
1	4.100932000	1.498211000	24.432596000
5	3.174167000	0.555634000	24.200756000
1	3.665450000	-0.514434000	24.104686000
1	2.291709000	0.787611000	24.951969000
1	5.654992000	5.064596000	22.973445000
5	6.856795000	4.575737000	23.229357000
1	6.363563000	3.580345000	23.928831000
1	7.743461000	5.228902000	23.594353000
1	6.879146000	3.887705000	22.117850000

$[B_2H_2]^{2-}$_MeCN E = -50.9706139720 Ha

5	0.119821000	0.018307000	-0.037701000
5	1.372895000	-0.127801000	-0.763316000
1	2.398447000	-0.246822000	-1.357298000
1	-0.905708000	0.137830000	0.556182000

$[B_2H]^-$_MeCN E = -50.1400781290 Ha

5	0.143915000	0.015441000	-0.051641000
5	1.367668000	-0.127089000	-0.760311000
1	2.379580000	-0.244668000	-1.346363000

$[B_3H_3]^{2-}$_MeCN E = -76.4472074480 Ha

5	4.228450000	1.128745000	1.482883000
5	4.084852000	1.049338000	-0.101913000
5	2.787926000	0.962811000	0.821502000
1	1.590292000	0.852907000	0.940120000
1	4.919759000	1.241089000	2.468645000
1	4.589141000	1.035250000	-1.200044000

$[B_3H_2]^-$_MeCN E = -75.7220326107 Ha

5	4.210911000	1.128945000	1.457682000
5	4.244571000	1.060262000	-0.031387000
5	2.801667000	0.972557000	0.997485000
1	1.655671000	0.849273000	0.726911000
1	4.367841000	1.018015000	-1.208142000

$[B_4H_4]^{2-}$_MeCN E = -101.902796323 Ha

5	2.497825000	0.150325000	2.335139000
5	2.825264000	0.841955000	0.920305000
1	3.079914000	1.920616000	0.444219000
5	2.513516000	-0.671567000	0.477386000
5	3.546453000	-0.884021000	1.690758000
1	1.877717000	0.339649000	3.351905000
1	1.904512000	-1.295472000	-0.356509000
1	4.525114000	-1.525632000	1.983047000

$[B_4H_3]^-$_MeCN E = -101.203037829 Ha

5	3.358431000	0.333165000	1.934567000
5	2.628240000	1.147605000	0.855147000
1	2.164474000	2.084083000	0.302861000
5	2.783613000	-0.499888000	0.608477000
5	3.605229000	-1.183614000	1.895047000
1	2.372548000	-1.093988000	-0.338741000
1	3.980062000	-2.251160000	2.236986000

$[B_5H_5]^{2-}$_MeCN E = -127.403478081 Ha

5	4.444889000	-1.143844000	0.796074000
5	5.043102000	-1.316542000	-0.745670000
5	2.946005000	-0.465874000	0.559372000
5	3.409358000	-1.578619000	-0.588130000
5	4.129190000	0.048513000	-0.490167000
1	2.726724000	-2.379478000	-1.168811000
1	4.971710000	-1.445379000	1.833155000
1	4.288580000	1.147388000	-0.949759000
1	1.985312000	-0.080511000	1.153832000

1	6.003830000	-1.701612000	-1.340271000

$[B_5H_4]^-$_ap_MeCN E = -126.602717680 Ha

5	4.603196000	-1.078245000	0.873663000
5	4.913211000	-1.345604000	-0.623079000
5	3.030310000	-0.426336000	0.477284000
5	3.388327000	-1.595866000	-0.768788000
5	4.096758000	0.165393000	-0.494563000
1	2.521145000	-2.287074000	-1.191950000
1	4.884550000	-1.277328000	2.009324000
1	4.446747000	1.155819000	-1.065270000
1	2.090842000	-0.501163000	1.201187000

$[B_5H_4]^-$_eq_MeCN E = -126.639100797 Ha

5	4.403736000	-1.024876000	0.886930000
5	5.090097000	-1.154698000	-0.828498000
5	2.873264000	-0.505940000	0.624844000
5	3.271223000	-1.492675000	-0.614826000
5	4.124206000	-0.080998000	-0.445793000
1	2.911954000	-2.452282000	-1.209823000
1	5.083679000	-1.548280000	1.703915000
1	1.955617000	0.036428000	1.151292000
1	5.946343000	-1.840025000	-1.258657000

$[B_6H_6]^{2-}$_MeCN E = -152.929889855 Ha

5	1.218442000	-0.002599000	0.112491000
1	2.418507000	-0.012102000	0.115197000
5	0.012376000	1.222906000	0.151577000
5	-0.000493000	-0.034082000	1.325378000
5	0.005307000	0.049161000	-1.105522000
5	-1.213638000	0.017698000	0.107396000
5	-0.007594000	-1.207816000	0.068309000
1	-2.413704000	0.027354000	0.105059000
1	0.007879000	0.087931000	-2.304939000
1	-0.016678000	-2.407221000	0.029693000
1	-0.003074000	-0.072835000	2.524789000
1	0.021555000	2.422294000	0.190299000

$[B_6H_5]^-$_MeCN E = -152.145398235 Ha

5	1.016915000	-0.000630000	0.112002000
5	0.017165000	1.284429000	0.152647000
5	0.003968000	-0.035126000	1.386923000
5	0.009830000	0.050226000	-1.167034000
5	-1.116609000	0.016540000	0.107696000
5	-0.003346000	-1.269451000	0.067243000
1	-2.307248000	0.026377000	0.105247000
1	0.003086000	0.090017000	-2.350141000
1	-0.022368000	-2.452427000	0.027590000
1	-0.008249000	-0.074723000	2.569993000
1	0.017234000	2.467559000	0.192363000

$[B_7H_7]^{2-}$_MeCN E = -178.390435612 Ha

5	8.299703000	19.095461000	4.107398000
1	9.121194000	18.975959000	4.972105000
5	6.937472000	20.019415000	4.193295000
1	6.598823000	20.685761000	5.130442000
5	6.126735000	19.864980000	2.767361000
1	5.093392000	20.399260000	2.478158000
5	6.987458000	18.844278000	1.801502000
1	6.689352000	18.507597000	0.689885000
5	8.331002000	18.368417000	2.629262000
1	9.180338000	17.624812000	2.225999000
5	7.912105000	20.126092000	2.675713000
1	8.520031000	21.064488000	2.227156000
5	6.760676000	18.351084000	3.523774000
1	6.152729000	17.412691000	3.972327000

$[B_7H_6]^-$_ap_MeCN E = -177.631934001 Ha

5	8.322546000	19.080816000	4.144021000
1	9.142969000	18.973018000	4.990842000
5	6.918451000	20.033521000	4.231690000
1	6.590045000	20.704266000	5.150494000
5	6.082861000	19.873467000	2.761535000
1	5.069119000	20.413232000	2.473388000
5	6.970417000	18.821836000	1.765255000
1	6.683480000	18.500681000	0.662244000
5	8.354485000	18.331645000	2.619760000
1	9.201336000	17.608922000	2.216720000
5	7.862356000	20.049562000	2.712428000
1	8.462599000	20.974381000	2.270652000
5	6.897615000	18.562256000	3.423021000

$[B_7H_6]^-$_eq_MeCN E = -177.613739297 Ha

5	8.375116000	19.040521000	4.095070000
1	9.102492000	19.016252000	5.030577000
5	6.979948000	19.988931000	4.186209000
1	6.711086000	20.625963000	5.155674000
5	6.158161000	19.830943000	2.739577000
1	5.135267000	20.323107000	2.379377000
5	7.043900000	18.784500000	1.751994000
1	6.623448000	18.540981000	0.671012000
5	8.240784000	18.447818000	2.672194000
5	7.878646000	20.207488000	2.686010000
1	8.497649000	21.119478000	2.234929000
5	6.684116000	18.366153000	3.565833000
1	6.100059000	17.423348000	3.999920000

$[B_8H_8]^{2-}$_MeCN E = -203.842628331 Ha

5	1.597360000	4.449706000	2.296386000
5	1.631042000	4.878782000	3.951855000
1	1.378988000	3.302312000	2.010134000
1	1.446677000	4.086220000	4.829820000
5	0.598767000	5.909642000	2.898473000
5	3.038219000	5.427308000	2.974483000
5	1.084087000	5.827740000	1.260341000
5	2.657989000	5.516946000	1.309423000
5	1.941282000	6.453529000	3.965255000
5	2.079122000	6.890311000	2.317387000
1	-0.557478000	6.135413000	3.139977000
1	4.176708000	5.198978000	3.287506000
1	0.321811000	5.986434000	0.351059000
1	3.475997000	5.362149000	0.449523000
1	2.069769000	7.241701000	4.856785000
1	2.314295000	8.038981000	2.050228000

$[B_8H_7]^-$_ap_MeCN E = -203.058784578 Ha

5	1.609903000	4.370608000	2.365385000
5	1.649458000	4.858379000	3.966220000
1	1.381450000	3.261450000	1.998514000
1	1.442636000	4.140486000	4.892225000
5	0.687459000	5.892126000	2.875806000
5	3.058686000	5.422737000	3.012843000
5	1.082947000	5.829709000	1.096170000
5	2.507071000	5.544763000	1.458967000
5	1.966188000	6.464702000	3.979788000
5	2.120760000	6.956909000	2.387329000
1	-0.483377000	6.122828000	2.916501000
1	4.213064000	5.193683000	3.207946000
1	0.165037000	6.017701000	0.383766000
1	2.045017000	7.192352000	4.917900000
1	2.332341000	8.075571000	2.039751000

$[B_8H_7]^-$_eq_MeCN E = -203.084110828 Ha

5	1.490735000	4.449704000	2.281846000
5	1.647607000	4.855998000	3.972984000
1	1.258101000	3.308340000	2.047781000
1	1.502288000	4.002099000	4.782638000
5	0.610045000	5.907160000	2.939112000
5	2.851028000	5.464489000	2.938066000
5	1.151704000	5.814365000	1.275875000
5	2.798387000	5.488286000	1.366614000
5	1.965745000	6.467619000	3.986878000
5	1.980797000	6.930900000	2.303030000
1	-0.531568000	6.130162000	3.217698000
1	0.546079000	5.942405000	0.259928000
1	3.453965000	5.367770000	0.389537000
1	2.153127000	7.299163000	4.811133000
1	2.199885000	8.078714000	2.088012000

$[B_9H_9]^{2-}$_MeCN E = -229.322982551Ha

5	1.639800000	1.663838000	6.855571000
5	0.677705000	0.726092000	7.902333000
5	1.621089000	0.710011000	9.592193000
1	1.657010000	1.691324000	5.662766000
1	-0.025241000	-0.133626000	7.451350000
1	1.628210000	-0.162024000	10.413846000
5	2.612736000	0.770658000	7.931217000
5	2.570870000	2.551735000	7.971659000
5	0.636143000	2.507428000	7.942925000
5	0.183902000	1.574584000	9.293950000
5	3.025538000	1.639515000	9.336332000
1	3.366439000	-0.056271000	7.501301000
5	1.579851000	2.490593000	9.632704000
1	3.285841000	3.430688000	7.580768000
1	-0.105514000	3.353566000	7.529847000
1	-0.855717000	1.536916000	9.878131000
1	4.048856000	1.649251000	9.949987000
1	1.548359000	3.324247000	10.492726000

$[B_9H_8]^-$_ap_MeCN E = -228.532455594 Ha

5	1.619421000	1.593781000	6.796682000
5	0.715156000	0.699108000	7.941277000
5	1.606772000	0.683804000	9.539260000
1	1.875539000	1.628158000	5.637993000
1	-0.020002000	-0.229778000	7.824671000
1	1.312689000	-0.252740000	10.212229000
5	2.571762000	0.764617000	7.953739000
5	2.290429000	2.621225000	8.128462000
5	0.414755000	2.437818000	7.531711000
5	0.599683000	2.057730000	9.066377000
5	3.063359000	1.568978000	9.383644000
1	3.370688000	0.001430000	7.500467000
5	1.812027000	2.413192000	10.035566000
1	2.851304000	3.627754000	7.824842000
1	-0.272895000	3.205347000	6.948588000
1	4.184543000	1.588614000	9.773347000
1	1.951515000	3.167353000	10.937767000

$[B_9H_8]^-$_eq_MeCN E = -228.540583622 Ha

5	1.638936000	1.700539000	6.859800000
5	0.666739000	0.744563000	7.915669000
5	1.622238000	0.663634000	9.586872000
1	1.656114000	1.719606000	5.672039000
1	-0.046801000	-0.117550000	7.504702000
1	1.630201000	-0.207507000	10.387867000
5	2.622164000	0.789268000	7.944611000
5	2.637639000	2.556297000	7.945060000
5	0.570453000	2.509067000	7.914344000
5	0.153089000	1.594297000	9.313672000
5	3.054879000	1.660400000	9.356662000
1	3.385994000	-0.039234000	7.555496000
5	1.586111000	2.348115000	9.430561000
1	3.332359000	3.441163000	7.566375000
1	-0.152487000	3.361330000	7.514307000
1	-0.847723000	1.554650000	9.943369000
1	4.037614000	1.665641000	10.015474000

$[B_{10}H_{10}]^{2-}$_MeCN E = -254.829971350 Ha

5	0.329222000	5.335743000	5.514006000
5	3.073272000	4.588887000	6.136002000
5	1.771017000	5.826677000	6.257885000
5	2.521595000	3.594064000	4.715556000
5	3.112644000	6.071636000	5.081973000
5	0.990819000	4.419620000	4.249584000
5	1.408609000	6.171349000	4.507986000
5	3.868991000	4.620819000	4.639190000
5	2.560979000	5.076738000	3.661570000
5	1.353315000	4.074570000	5.998720000
1	3.674521000	4.296405000	7.128620000
1	0.270992000	3.878638000	3.461064000
1	3.748200000	7.083977000	5.149235000
1	-0.805977000	5.565037000	5.791073000
1	1.738723000	6.520361000	7.233208000
1	2.638579000	2.429838000	4.463328000
1	5.003969000	4.390902000	4.361686000
1	2.713721000	5.216812000	2.482613000
1	1.057028000	7.167860000	3.945985000
1	0.952796000	3.229816000	6.746040000

$[B_{10}H_9]^-$_ap_MeCN E = -254.018178472 Ha

5	0.515369000	5.298177000	5.467467000
5	3.066206000	4.587669000	6.154152000
5	1.792752000	5.855763000	6.300008000
5	2.507812000	3.580429000	4.715803000
5	3.106221000	6.089172000	5.087181000
5	0.979134000	4.388216000	4.204929000
5	1.414842000	6.215479000	4.474908000
5	3.830127000	4.628384000	4.648997000
5	2.547604000	5.081756000	3.648903000
5	1.356774000	4.028364000	6.029719000
1	3.681496000	4.295117000	7.130823000
1	0.233648000	3.866388000	3.442122000
1	3.755433000	7.085866000	5.148178000
1	1.721600000	6.543689000	7.265388000
1	2.644998000	2.425289000	4.460640000
1	4.960575000	4.400483000	4.371198000
1	2.718943000	5.215291000	2.477897000
1	1.031189000	7.200447000	3.933873000
1	0.924271000	3.208734000	6.772064000

$[B_{10}H_9]^-$_eq_MeCN E = -254.040117288 Ha

5	0.306222000	5.360612000	5.547678000
5	3.111612000	4.581430000	6.147369000
5	1.792689000	5.685929000	6.060553000
5	2.538559000	3.588821000	4.729614000
5	3.151659000	6.083233000	5.079541000
5	0.985078000	4.439225000	4.276478000
5	1.393594000	6.189426000	4.459946000
5	3.886916000	4.620406000	4.640243000
5	2.578535000	5.090908000	3.661987000
5	1.336097000	4.023670000	5.999274000
1	3.642339000	4.317507000	7.177233000
1	0.210593000	3.929457000	3.529266000
1	3.716950000	7.123232000	5.183144000
1	-0.817133000	5.590988000	5.829610000
1	2.669426000	2.429566000	4.491176000
1	5.007415000	4.376585000	4.339800000
1	2.744369000	5.241766000	2.492483000
1	1.048131000	7.176875000	3.905970000
1	0.941240000	3.189753000	6.740749000

$[B_{11}H_{11}]^{2-}$_MeCN E = -280.273290874 Ha

5	1.788200000	16.615973000	15.250711000
5	2.550220000	18.177531000	15.320049000
5	0.614752000	15.503881000	15.889745000
5	2.718194000	17.420860000	16.790698000
5	0.910324000	18.376869000	15.131154000
5	-0.150008000	16.910910000	15.443510000
5	1.658213000	15.955404000	17.103249000
5	1.667057000	18.853650000	16.671443000
5	-0.036805000	16.499427000	17.172987000
5	1.257127000	17.542197000	17.792723000
5	0.020078000	18.195853000	16.657286000
1	2.316839000	16.039135000	14.342858000
1	3.416056000	18.605150000	14.623010000
1	0.371546000	14.398468000	15.518763000
1	3.789779000	17.274474000	17.303924000

1	0.454316000	19.037828000	14.243253000
1	-1.085124000	16.908684000	14.695758000
1	2.249709000	15.145096000	17.756890000
1	1.882571000	19.959212000	17.073880000
1	-0.906295000	16.106043000	17.893791000
1	1.326409000	17.743912000	18.969341000
1	-0.894797000	18.915396000	16.931351000

$[B_{11}H_{10}]^-$_ap_MeCN E = -279.485017945 Ha

5	1.655710000	16.760814000	15.478803000
5	2.559010000	18.193434000	15.319099000
5	0.603545000	15.490972000	15.894823000
5	2.755026000	17.449120000	16.799167000
5	0.918886000	18.418988000	15.113945000
5	-0.190361000	16.886041000	15.440655000
5	1.645806000	15.916380000	17.126005000
5	1.679031000	18.867336000	16.666004000
5	-0.046345000	16.483121000	17.173993000
5	1.268672000	17.533556000	17.791558000
5	0.017497000	18.194422000	16.643162000
1	3.417830000	18.518937000	14.571065000
1	0.442001000	14.406392000	15.446764000
1	3.810483000	17.260590000	17.303373000
1	0.448632000	19.036368000	14.218651000
1	-1.091941000	16.907454000	14.672296000
1	2.269730000	15.131319000	17.757164000
1	1.882097000	19.965107000	17.076643000
1	-0.910690000	16.104710000	17.898080000
1	1.370172000	17.711213000	18.963855000
1	-0.903269000	18.910634000	16.878410000

$[B_{11}H_{10}]^-$_eq1_MeCN E = -279.500480098 Ha

5	1.436051000	16.497627000	14.931353000
5	2.426601000	17.602775000	15.760373000
5	0.531818000	15.470829000	15.908418000
5	2.900014000	17.617459000	17.235683000
5	0.960108000	18.231308000	15.136828000
5	-0.242569000	16.961574000	15.407610000
5	1.865110000	16.133885000	16.780856000
5	1.641456000	18.870164000	16.660996000
5	-0.004141000	16.493216000	17.120906000
5	1.246478000	17.602619000	17.800076000
5	0.012301000	18.199492000	16.670198000
1	1.958296000	16.042518000	13.962473000
1	0.287995000	14.331380000	15.700557000
1	3.788023000	17.662521000	18.006737000
1	0.910047000	18.986292000	14.222130000
1	-1.245434000	16.934377000	14.768724000
1	2.562139000	15.262817000	17.194122000
1	1.911185000	20.015533000	16.798886000
1	-0.784768000	16.107219000	17.932497000
1	1.229940000	17.639692000	18.988814000
1	-0.888346000	18.917507000	16.965127000

$[B_{11}H_{10}]^-$_eq2_MeCN E = -279.500500166 Ha

5	1.850940000	16.647757000	15.269050000
5	2.574297000	18.212891000	15.340682000
5	0.491614000	15.488209000	15.803126000
5	2.708142000	17.431268000	16.814914000
5	0.910028000	18.331063000	15.134316000
5	0.024195000	16.946468000	15.568218000
5	1.614836000	16.012224000	17.035285000
5	1.662338000	18.880168000	16.680872000
5	-0.041443000	16.529282000	17.258097000
5	1.266319000	17.565515000	17.823757000
5	0.017321000	18.228061000	16.704856000
1	2.318102000	16.017621000	14.374589000
1	3.408027000	18.666543000	14.633608000
1	0.384114000	14.344018000	15.549104000
1	3.773155000	17.290781000	17.327759000
1	0.497622000	18.854968000	14.147358000
1	2.175198000	15.068392000	17.493436000
1	1.879191000	19.983831000	17.067189000
1	-0.926807000	16.115883000	17.927992000
1	1.355162000	17.769523000	18.991764000
1	-0.938866000	18.892800000	16.934642000

$[B_{12}H_{12}]^{2-}$_MeCN E = -305.838190013 Ha

5	-0.789798000	-0.933910000	1.182040000
5	0.379520000	0.325520000	1.614424000
1	-1.362355000	-1.570183000	2.015677000
1	0.633994000	0.579427000	2.753683000
5	0.933935000	-1.173015000	0.848661000
1	1.582212000	-1.980812000	1.444305000
5	-0.276057000	-1.654587000	-0.353052000
1	-0.484477000	-2.803171000	-0.607126000
5	-1.173182000	0.770430000	0.885956000
1	-2.019191000	1.341098000	1.507393000
5	-1.578648000	-0.453458000	-0.329925000
1	-2.711372000	-0.748310000	-0.569012000
5	1.210375000	-0.840392000	-0.869540000
1	2.056371000	-1.411047000	-1.491011000
5	1.615839000	0.383500000	0.346337000
1	2.748553000	0.678390000	0.585432000
5	-0.896756000	1.103061000	-0.832255000
1	-1.545038000	1.910863000	-1.427887000
5	0.313243000	1.584622000	0.369450000
1	0.521678000	2.733213000	0.623493000
5	0.826984000	0.863950000	-1.165641000
1	1.399538000	1.500253000	-1.999234000
5	-0.342347000	-0.395474000	-1.598017000
1	-0.596822000	-0.649443000	-2.737266000

$[B_{12}H_{11}]^-$_MeCN E = -305.041477377 Ha

5	-0.665172000	-0.795516000	1.001263000
5	0.400289000	0.347911000	1.639712000
1	0.616184000	0.560011000	2.785244000
5	0.969320000	-1.190056000	0.853855000
1	1.565210000	-2.004745000	1.474388000
5	-0.272381000	-1.684100000	-0.379361000
1	-0.504767000	-2.828198000	-0.581435000
5	-1.193141000	0.804536000	0.892308000
1	-2.040949000	1.321794000	1.538348000
5	-1.609006000	-0.451455000	-0.355601000
1	-2.734217000	-0.772060000	-0.541981000
5	1.216729000	-0.848031000	-0.872869000
1	2.057243000	-1.419698000	-1.489139000
5	1.624857000	0.384097000	0.351487000
1	2.751405000	0.676031000	0.593284000
5	-0.905027000	1.108934000	-0.835470000
1	-1.553275000	1.911275000	-1.426298000
5	0.313413000	1.593899000	0.374545000
1	0.519367000	2.735955000	0.631736000
5	0.815688000	0.851423000	-1.149265000
1	1.386251000	1.484853000	-1.978529000
5	-0.346616000	-0.399923000	-1.606502000
1	-0.602852000	-0.656229000	-2.738511000

$[B_{13}H_{13}]^{2-}$_MeCN E = -331.205624141 Ha

5	0.073777000	1.445963000	0.698923000
5	-0.036353000	-1.195234000	0.841157000
5	-1.343146000	0.206872000	1.233446000
5	1.400691000	0.090006000	1.175126000
1	0.131350000	2.557281000	1.141486000
1	-0.070231000	-2.252407000	1.402637000
5	0.048514000	0.193062000	2.049130000
5	-1.456770000	1.003726000	-0.342533000
5	-1.529078000	-0.742034000	-0.248322000
5	1.443220000	-0.869174000	-0.311538000
5	1.515573000	0.876402000	-0.405823000
5	0.034868000	1.440298000	-1.126242000
5	-0.082070000	-1.382331000	-0.974230000
5	0.841936000	-0.043274000	-1.719192000
5	-0.920057000	0.032255000	-1.681392000
1	0.076178000	0.255968000	3.239098000
1	-2.357707000	0.281805000	1.869641000
1	2.442718000	0.078356000	1.769649000
1	-2.415988000	1.710368000	-0.431751000
1	-2.543468000	-1.372928000	-0.266763000
1	2.399057000	-1.583428000	-0.371020000
1	2.527055000	1.498220000	-0.536848000
1	0.066358000	2.471715000	-1.727482000
1	-0.140993000	-2.471887000	-1.459238000
1	1.399300000	-0.123831000	-2.771270000
1	-1.526827000	0.001881000	-2.708785000

$[B_{13}H_{12}]^-$_ap_MeCN E = -330.475236632 Ha

5	0.362162000	1.549611000	0.556191000
5	0.242430000	-1.337579000	0.712587000
5	-2.005852000	0.251143000	1.465760000
5	1.258409000	0.090703000	1.080694000
1	0.423353000	2.586982000	1.130376000
1	0.221047000	-2.309185000	1.394903000
5	-0.474509000	0.173729000	1.290746000
5	-1.206016000	1.014461000	-0.130575000
5	-1.279704000	-0.752471000	-0.035080000
5	1.555639000	-0.889512000	-0.365672000
5	1.629027000	0.881817000	-0.461637000
5	0.119322000	1.459305000	-1.200909000
5	0.000335000	-1.416549000	-1.045133000
5	0.849450000	-0.046488000	-1.772727000
5	-0.900266000	0.036161000	-1.579880000
1	-3.143842000	0.311797000	1.727787000
1	1.997813000	0.110452000	2.010176000
1	-2.212853000	1.653036000	0.028729000
1	-2.334260000	-1.286783000	0.184487000
1	2.534285000	-1.558056000	-0.463247000
1	2.658980000	1.452493000	-0.626847000
1	0.054646000	2.447153000	-1.857184000
1	-0.149161000	-2.460567000	-1.591035000
1	1.347534000	-0.126936000	-2.849744000
1	-1.646238000	0.016964000	-2.504002000

$[B_{13}H_{12}]^-$_eq1_MeCN E = -330.475227695 Ha

5	-0.006962000	1.690303000	0.390194000
5	-0.143896000	-1.175351000	0.733684000
5	-0.974824000	0.348801000	1.083971000
5	0.829648000	0.300915000	1.098811000
1	0.009975000	2.741271000	0.942197000
1	-0.180901000	-2.066304000	1.539891000
5	0.284793000	-0.288300000	2.416568000
5	-1.455529000	1.086162000	-0.440683000
5	-1.552774000	-0.690440000	-0.232604000
5	1.351575000	-0.799926000	-0.184256000
5	1.436558000	0.976996000	-0.394095000
5	0.024606000	1.460028000	-1.351213000
5	-0.102790000	-1.394347000	-1.013232000
5	0.857785000	-0.067868000	-1.703713000
5	-0.926660000	-0.003082000	-1.729524000
1	-0.018187000	-0.699007000	3.468681000
1	-1.559357000	0.470712000	2.128331000
1	-2.440588000	1.746876000	-0.502922000
1	-2.589011000	-1.268540000	-0.179631000
1	2.312877000	-1.480651000	-0.035199000
1	2.473881000	1.553942000	-0.438301000
1	0.076143000	2.382970000	-2.099299000
1	-0.133267000	-2.486165000	-1.479699000
1	1.491645000	-0.213761000	-2.699134000
1	-1.529549000	-0.095939000	-2.750609000

$[B_{13}H_{12}]^-$_eq2_MeCN E = -330.475267460 Ha

5	-0.342460000	1.747573000	0.518099000
5	-0.170567000	-1.094640000	0.836327000
5	-1.201489000	0.313579000	1.161877000
5	1.710925000	-1.121263000	1.338898000
1	-0.445470000	2.806235000	1.046284000
1	-0.084216000	-2.030923000	1.586121000
5	0.563527000	0.451683000	1.293113000
5	-1.645495000	0.987422000	-0.404809000
5	-1.537181000	-0.768478000	-0.225169000
5	1.309165000	-0.527543000	-0.027295000
5	1.201039000	1.231009000	-0.192038000
5	-0.171944000	1.551512000	-1.240541000
5	0.001442000	-1.295297000	-0.938429000
5	0.854870000	0.145506000	-1.574170000
5	-0.907754000	0.002017000	-1.698245000
1	1.137392000	0.542321000	2.345973000
1	-1.917157000	0.373349000	2.108136000
1	2.122772000	-1.605802000	2.320118000
1	-2.690560000	1.531364000	-0.567479000
1	-2.483349000	-1.486528000	-0.221655000
1	2.180416000	1.899064000	-0.125507000
1	-0.188373000	2.487767000	-1.973616000
1	0.146094000	-2.383793000	-1.389987000
1	1.576430000	0.081430000	-2.515622000
1	-1.439208000	-0.146489000	-2.751496000

$[B_{13}H_{12}]^-$_eq3_MeCN E = -330.422095391 Ha

5	0.072530000	1.420465000	0.688861000
5	-0.025770000	-0.886278000	0.714993000
5	-1.366758000	0.199567000	1.246656000
5	1.423938000	0.082024000	1.187068000
1	0.129869000	2.497174000	1.193866000
5	0.048241000	0.181347000	2.069290000
5	-1.467584000	1.000136000	-0.363783000
5	-1.556819000	-0.744029000	-0.275796000
5	1.468878000	-0.873501000	-0.339857000
5	1.525604000	0.871950000	-0.427604000
5	0.034417000	1.429672000	-1.129881000
5	-0.082603000	-1.380149000	-0.984487000
5	0.845354000	-0.046485000	-1.739597000
5	-0.924694000	0.029371000	-1.701965000
1	0.076159000	0.240334000	3.249373000
1	-2.369324000	0.275650000	1.877338000
1	2.454217000	0.073175000	1.776121000
1	-2.421898000	1.707623000	-0.402424000
1	-2.533698000	-1.415155000	-0.252482000
1	2.384895000	-1.625584000	-0.355165000
1	2.534459000	1.494971000	-0.508625000
1	0.067551000	2.474380000	-1.695965000
1	-0.139320000	-2.499306000	-1.375121000
1	1.394774000	-0.136429000	-2.788870000
1	-1.524281000	-0.010866000	-2.726716000

$[B_{13}H_{12}]^-$_eq4_MeCN E = -330.408317226 Ha

5	0.100453000	1.445505000	0.715793000
5	-0.009612000	-1.195024000	0.857997000
5	-1.398422000	0.209722000	1.240580000
5	1.377209000	0.089156000	1.145374000
1	0.144878000	2.556436000	1.144863000
1	-0.056386000	-2.252606000	1.405065000
5	0.012786000	0.193581000	2.029154000
5	-1.480179000	1.026077000	-0.328392000
5	-1.554011000	-0.761026000	-0.232098000
5	1.463997000	-0.882769000	-0.332436000
5	1.537469000	0.885753000	-0.427915000
5	0.062590000	1.481199000	-1.110258000
5	-0.057908000	-1.423607000	-0.953812000
5	0.859479000	-0.045651000	-1.750446000
5	-0.784055000	0.038506000	-1.458634000
1	0.058614000	0.255356000	3.214236000
1	-2.403171000	0.284744000	1.882531000
1	2.404232000	0.079006000	1.753852000
1	-2.405580000	1.750482000	-0.482700000

1	-2.537070000	-1.418534000	-0.310871000
1	2.427985000	-1.576602000	-0.389588000
1	2.555342000	1.486781000	-0.555341000
1	0.014756000	2.489847000	-1.729912000
1	-0.192695000	-2.485694000	-1.461054000
1	1.364030000	-0.124868000	-2.819340000

$[B_{14}H_{14}]^{2-}$_MeCN E = -356.701323224 Ha

5	-1.703558000	-0.346995000	0.756726000
5	-1.130911000	1.287220000	0.748512000
5	0.570489000	1.608807000	0.745957000
5	1.699534000	0.296090000	0.752094000
5	1.126910000	-1.338141000	0.760271000
5	-0.574488000	-1.659732000	0.762274000
1	-1.805104000	2.074623000	1.341142000
1	0.912678000	2.588207000	1.336770000
1	2.716657000	0.490702000	1.346041000
1	1.801744000	-2.119532000	1.360083000
1	-0.916202000	-2.633483000	1.362603000
1	-2.720228000	-0.535739000	1.353349000
5	-1.636980000	0.539724000	-0.756877000
5	-0.324212000	1.668755000	-0.763191000
5	1.310041000	1.096159000	-0.761125000
5	1.631624000	-0.605236000	-0.752743000
5	0.318886000	-1.734269000	-0.747023000
5	-1.315361000	-1.161629000	-0.749056000
1	-0.516264000	2.682736000	-1.363320000
1	2.094233000	1.767696000	-1.361003000
1	2.607964000	-0.950011000	-1.347106000
1	0.510224000	-2.754059000	-1.337446000
1	-2.099984000	-1.839059000	-1.341666000
1	-2.613801000	0.878926000	-1.353640000
5	-0.002999000	-0.036601000	-1.547043000
5	-0.001674000	-0.021576000	1.546337000
1	-0.003494000	-0.042418000	-2.742457000
1	-0.001177000	-0.015737000	2.741751000

$[B_{14}H_{13}]^{-}$_ap_MeCN E = -355.905634542 Ha

5	-1.707150000	-0.347471000	0.761079000
5	-1.133306000	1.289985000	0.753142000
5	0.571738000	1.612127000	0.751051000
5	1.703120000	0.296632000	0.756446000
5	1.129304000	-1.340851000	0.764920000
5	-0.575742000	-1.662921000	0.767419000
1	-1.812036000	2.082567000	1.321946000
1	0.915936000	2.597703000	1.319001000
1	2.727142000	0.492269000	1.326739000
1	1.808596000	-2.127788000	1.340846000
1	-0.919509000	-2.643023000	1.345007000
1	-2.730693000	-0.537551000	1.334097000
5	-1.654878000	0.546042000	-0.757701000
5	-0.327562000	1.688124000	-0.763420000
5	1.324800000	1.109125000	-0.761520000
5	1.649520000	-0.611547000	-0.753548000
5	0.322253000	-1.753651000	-0.747009000
5	-1.330098000	-1.174628000	-0.749290000
1	-0.514129000	2.669682000	-1.399869000
1	2.084043000	1.759280000	-1.397505000
1	2.594962000	-0.945829000	-1.384394000
1	0.508148000	-2.741396000	-1.374007000
1	-2.089865000	-1.830869000	-1.378356000
1	-2.600851000	0.874291000	-1.390906000
5	-0.002872000	-0.035117000	-1.251724000
5	-0.001677000	-0.021551000	1.555299000
1	-0.001152000	-0.015789000	2.744927000

$[B_{14}H_{13}]^{-}$_eq_MeCN E = -355.910791002 Ha

5	-1.736293000	-0.353336000	0.767447000
5	-1.138884000	1.292806000	0.760109000
5	0.571118000	1.610448000	0.744863000
5	1.708920000	0.295198000	0.763971000
5	1.148243000	-1.363785000	0.771373000
5	-0.488151000	-1.413339000	0.630884000
1	-1.814410000	2.068872000	1.354368000
1	0.912680000	2.588409000	1.328329000
1	2.720192000	0.480351000	1.359356000
1	1.749433000	-2.146464000	1.428729000
1	-2.695392000	-0.589950000	1.423295000
5	-1.675794000	0.540254000	-0.755741000
5	-0.334813000	1.648965000	-0.761081000
5	1.306179000	1.073969000	-0.758940000
5	1.662061000	-0.629124000	-0.751235000
5	0.343635000	-1.740151000	-0.790004000
5	-1.338773000	-1.150906000	-0.792394000
1	-0.536133000	2.654593000	-1.363471000
1	2.092498000	1.733161000	-1.360827000
1	2.645430000	-0.954022000	-1.334401000
1	0.442805000	-2.771743000	-1.361728000
1	-2.058382000	-1.895829000	-1.365321000
1	-2.645576000	0.899362000	-1.341561000
5	0.005628000	-0.011299000	-1.552224000
5	0.017950000	0.034825000	1.570180000
1	-0.007109000	-0.052613000	-2.741299000
1	-0.006312000	-0.029741000	2.754936000

$[B_{15}H_{15}]^{2-}$_MeCN E = -382.136137414 Ha

5	0.017219000	6.759286000	13.091669000
5	-0.285927000	5.468433000	12.006638000
1	-1.157242000	5.474040000	11.194263000
5	1.156193000	6.383109000	11.868333000
1	1.404191000	7.098372000	10.947960000
5	-0.717032000	5.287178000	13.695590000
5	1.214316000	4.649165000	11.620255000
1	1.333045000	4.289619000	10.488234000
5	1.728300000	6.838118000	13.460659000
1	2.189266000	7.935159000	13.554399000
5	0.146032000	3.819982000	12.915390000
5	2.581436000	5.364531000	12.681459000
1	3.621448000	5.581638000	12.133488000
5	0.657849000	5.999845000	14.748898000
1	0.321213000	6.670795000	15.679227000
5	1.987141000	3.588355000	12.666040000
1	2.497094000	2.693749000	12.062277000
5	2.501523000	5.777596000	14.506626000
1	3.353484000	6.339930000	15.125442000
5	0.055932000	4.225851000	14.740730000
1	-0.719967000	3.755939000	15.516579000
5	2.821165000	4.098552000	14.120405000
1	3.908650000	3.660941000	14.334868000
5	1.682575000	4.474306000	15.344130000
1	1.886615000	4.327679000	16.509112000
5	1.379341000	3.183610000	14.259134000
1	1.348680000	2.036916000	14.581960000
1	-0.619245000	7.766557000	13.120560000
1	-1.882963000	5.351412000	13.943382000
1	-0.556992000	2.931439000	12.534047000

$[B_{15}H_{14}]^{-}$_ap_MeCN E = -381.332291945 Ha

5	0.016173000	6.744856000	13.097520000
5	-0.289883000	5.456125000	12.006682000
1	-1.157101000	5.469802000	11.195818000
5	1.157261000	6.368030000	11.871757000
1	1.396989000	7.082380000	10.952974000
5	-0.747495000	5.286464000	13.700345000
5	1.215087000	4.638224000	11.591327000
1	1.330893000	4.293241000	10.459530000
5	1.730801000	6.826118000	13.466586000
1	2.188826000	7.920607000	13.557707000
5	0.112245000	3.821051000	12.883548000
5	2.595979000	5.373854000	12.675483000
1	3.635144000	5.577127000	12.132847000
5	0.656749000	6.014561000	14.759822000
1	0.320977000	6.671124000	15.693517000
5	2.008606000	3.581918000	12.620698000
1	2.517177000	2.645040000	12.100819000
5	2.522292000	5.792318000	14.521486000
1	3.371763000	6.347098000	15.140921000
5	0.013630000	4.240714000	14.764157000
1	-0.694258000	3.705311000	15.550831000
5	2.859525000	4.114138000	14.124100000
1	3.897797000	3.598060000	14.352869000
5	1.695295000	4.498214000	15.375395000
1	1.883026000	4.262502000	16.518183000
5	1.347511000	3.462681000	14.143601000
1	-0.620390000	7.748098000	13.118800000
1	-1.909299000	5.363049000	13.940711000
1	-0.550660000	2.892481000	12.551760000

$[B_{15}H_{14}]^{-}$_eq1_MeCN E = -381.346494683 Ha

5	0.009782000	6.773570000	13.110191000
5	-0.299755000	5.487739000	12.000180000
1	-1.172901000	5.507136000	11.197442000
5	1.143262000	6.403279000	11.861745000
1	1.380681000	7.127263000	10.952226000
5	-0.738237000	5.271381000	13.676298000
5	1.208138000	4.659607000	11.624491000
1	1.325268000	4.304110000	10.496025000
5	1.748620000	6.848472000	13.437400000
1	2.214709000	7.926945000	13.580990000
5	0.159350000	3.811237000	12.896628000
5	2.581301000	5.346967000	12.663659000
1	3.621846000	5.588319000	12.139475000
5	0.747680000	5.820314000	14.500152000
5	1.987387000	3.590597000	12.678632000
1	2.496226000	2.698389000	12.079749000
5	2.532509000	5.772810000	14.498064000
1	3.346972000	6.373880000	15.111519000
5	0.045939000	4.195796000	14.736693000
1	-0.750154000	3.774560000	15.504594000
5	2.829489000	4.089821000	14.142269000
1	3.909853000	3.656529000	14.372127000
5	1.676306000	4.487048000	15.364050000
1	1.845648000	4.396906000	16.532481000
5	1.386377000	3.174484000	14.280634000
1	1.356432000	2.037299000	14.617517000
1	-0.616036000	7.774422000	13.203116000
1	-1.882537000	5.328028000	13.973337000
1	-0.562026000	2.934397000	12.540845000

$[B_{15}H_{14}]^{-}$_eq2_MeCN E = -381.353257646 Ha

5	0.003083000	6.780251000	13.097129000
5	-0.305931000	5.464078000	11.990751000
1	-1.174920000	5.486385000	11.184075000
5	1.142695000	6.384717000	11.870190000
1	1.373485000	7.102423000	10.952374000
5	-0.715974000	5.286277000	13.695902000
5	1.217401000	4.657731000	11.608734000
1	1.353957000	4.292977000	10.486031000
5	1.731577000	6.846949000	13.449468000
1	2.208007000	7.931264000	13.546438000
5	0.131136000	3.833788000	12.835551000
5	2.570069000	5.360738000	12.689015000
1	3.617315000	5.560936000	12.159429000
5	0.658033000	6.078701000	14.722730000
1	0.323784000	6.663752000	15.698787000
5	1.978999000	3.588613000	12.673788000
1	2.455126000	2.672690000	12.084221000
5	2.492004000	5.771991000	14.509633000
1	3.314700000	6.332183000	15.159730000
5	0.278315000	4.354372000	14.527969000
5	2.836504000	4.071911000	14.147685000
1	3.937105000	3.665135000	14.322821000
5	1.713075000	4.434728000	15.390229000
1	1.815550000	4.398681000	16.567174000
5	1.407656000	3.136803000	14.298004000
1	1.248808000	1.991126000	14.541524000
1	-0.637276000	7.778706000	13.110528000
1	-1.843916000	5.235729000	14.070242000
1	-0.557062000	2.912528000	12.545026000

$[B_{16}H_{16}]^{2-}$_MeCN E = -407.597844685 Ha

5	0.138251000	1.280422000	0.932762000
5	-0.872345000	-1.447266000	1.497525000
5	-1.414882000	0.245570000	1.106670000
5	1.546487000	0.157171000	1.269131000
1	0.025763000	2.354728000	1.442159000
1	-1.291157000	-2.181447000	2.338340000
5	0.022480000	-0.074605000	2.047255000
5	-1.606656000	0.333569000	-0.753226000
5	-1.921806000	-1.133949000	0.161533000
5	0.848665000	-1.445043000	1.170579000
5	1.505972000	1.123977000	-0.160277000
5	-0.044761000	1.356443000	-0.931296000
5	0.544445000	-1.355536000	-1.474797000
5	1.225084000	0.252060000	-1.625448000
5	-1.204203000	-1.336272000	-1.395328000
1	-0.005220000	0.148178000	3.218279000
1	-2.289467000	0.807921000	1.693672000
1	2.482060000	0.327520000	1.988642000
1	-2.583764000	0.936325000	-1.082178000
1	-3.047561000	-1.512157000	0.267863000
1	1.465789000	-2.301054000	1.729532000
1	2.302398000	2.009579000	-0.211120000
1	-0.249822000	2.459941000	-1.338177000
1	1.009636000	-2.169728000	-2.213832000
1	1.980676000	0.467404000	-2.522436000
1	-1.815947000	-2.007458000	-2.168160000
5	-0.441555000	0.069658000	-2.045151000
1	-0.746288000	0.359426000	-3.160921000
5	1.796523000	-0.678212000	-0.251863000
1	2.881331000	-1.157791000	-0.390328000
5	-0.401613000	-2.125238000	-0.051048000
1	-0.401151000	-3.318737000	-0.094192000

$[B_{16}H_{15}]^{-}$_ap_MeCN E = -406.809772083 Ha

5	0.138089000	1.288703000	0.926475000
5	-0.896431000	-1.473928000	1.489558000
5	-1.422394000	0.250460000	1.098913000
5	1.580003000	0.166740000	1.257520000
1	0.051527000	2.342336000	1.470986000
1	-1.172371000	-2.173675000	2.402852000
5	0.020943000	-0.077577000	2.003707000
5	-1.614715000	0.328183000	-0.755930000
5	-1.974876000	-1.145561000	0.166160000
5	0.670244000	-1.229613000	0.782913000
5	1.538623000	1.167247000	-0.162976000
5	-0.041215000	1.359355000	-0.927065000
5	0.556932000	-1.353796000	-1.331706000
5	1.243152000	0.279841000	-1.627074000
5	-1.236416000	-1.342880000	-1.394128000
1	0.052311000	0.056776000	3.178425000
1	-2.282826000	0.787667000	1.718985000
1	2.439825000	0.227097000	2.067864000
1	-2.584540000	0.933802000	-1.084764000
1	-3.104446000	-1.491056000	0.282911000
1	2.307738000	2.070435000	-0.202601000
1	-0.252046000	2.457237000	-1.333490000
1	1.061459000	-2.213418000	-1.977698000
1	1.952084000	0.454847000	-2.562922000
1	-1.797469000	-1.992831000	-2.213876000
5	-0.427056000	0.050750000	-2.015786000
1	-0.703205000	0.300658000	-3.144141000
5	1.920728000	-0.582459000	-0.275828000
1	2.970109000	-1.113415000	-0.415900000

5	-0.541287000	-2.201981000	-0.051029000
1	-0.480900000	-3.383492000	-0.105720000

$[B_{16}H_{15}]^{-}$_eq_MeCN E = -406.809788763 Ha

5	0.154106000	1.290249000	0.959400000
5	-0.877958000	-1.430358000	1.523313000
5	-1.399584000	0.264995000	1.089856000
5	1.557810000	0.140226000	1.304621000
1	0.094846000	2.359839000	1.475110000
1	-1.331943000	-2.189350000	2.315094000
5	0.023123000	-0.059787000	2.096581000
5	-1.625617000	0.318035000	-0.784808000
5	-1.887450000	-1.132417000	0.152907000
5	0.848986000	-1.449235000	1.318148000
5	1.473622000	1.100035000	-0.153423000
5	-0.076424000	1.348413000	-0.907726000
5	0.513595000	-1.348588000	-1.616383000
5	1.213550000	0.244428000	-1.653043000
5	-1.216374000	-1.334678000	-1.428354000
1	-0.025786000	0.181709000	3.257707000
1	-2.272527000	0.833296000	1.664710000
1	2.574943000	0.317533000	1.882290000
1	-2.603306000	0.911859000	-1.111260000
1	-2.991899000	-1.554889000	0.269868000
1	1.467601000	-2.328902000	1.814332000
1	2.334085000	1.907850000	-0.229539000
1	-0.245531000	2.450829000	-1.320264000
1	0.999580000	-2.185856000	-2.298707000
1	2.071644000	0.473726000	-2.434770000
1	-1.841219000	-2.045610000	-2.144729000
5	-0.460223000	0.081615000	-2.092259000
1	-0.760031000	0.406834000	-3.193793000
5	1.330112000	-0.693259000	-0.199845000
5	-0.297038000	-2.046849000	-0.059420000
1	-0.188664000	-3.228504000	-0.111123000

$[B_{17}H_{17}]^{2-}$_MeCN E = -433.066183332 Ha

5	0.295705000	1.411782000	0.841571000
5	-1.160099000	-1.700370000	1.205659000
5	-2.091790000	-0.361666000	0.593746000
5	1.226887000	0.072755000	1.453634000
1	0.154655000	2.414401000	1.468843000
1	-1.488292000	-2.295034000	2.184042000
5	-0.582685000	-0.013704000	1.537143000
5	-1.142413000	0.978924000	-0.174565000
5	-2.037225000	-1.863071000	-0.295086000
5	0.636563000	-1.578948000	0.994082000
5	1.826288000	1.007000000	0.106181000
5	0.459634000	1.433537000	-0.892612000
5	0.830254000	-1.553660000	-1.052870000
5	1.492057000	0.107634000	-1.352157000
5	-1.927973000	-0.339701000	-1.140528000
1	-0.908253000	0.269324000	2.653584000
1	-3.088251000	0.004537000	1.133820000
1	1.754389000	0.113738000	2.520637000
1	-1.851847000	1.941636000	-0.229669000
1	-2.988572000	-2.570688000	-0.393137000
1	1.143798000	-2.366722000	1.738818000
1	2.777785000	1.714379000	0.204349000
1	0.435662000	2.451265000	-1.511149000
1	1.470409000	-2.323177000	-1.709282000
1	2.209852000	0.172820000	-2.300632000
1	-2.806875000	0.043397000	-1.847110000
5	-0.269298000	0.027478000	-1.775123000
1	-0.380971000	0.338480000	-2.925552000
5	1.966119000	-0.732906000	0.097755000
1	3.024043000	-1.271727000	0.192136000
5	-0.420950000	-2.506220000	-0.150351000
1	-0.217256000	-3.679796000	-0.145956000
5	-0.894933000	-1.665182000	-1.600143000
1	-1.031813000	-2.234020000	-2.637507000

$[B_{17}H_{16}]^{-}$_ap1_MeCN E = -432.253630078 Ha

5	0.281269000	1.404855000	0.842328000
5	-1.135740000	-1.696897000	1.249090000
5	-2.094378000	-0.318829000	0.619291000
5	1.214846000	0.062585000	1.455716000
1	0.144734000	2.406013000	1.467289000
1	-1.530424000	-2.323674000	2.171686000
5	-0.590510000	0.000121000	1.582172000
5	-1.167643000	1.023429000	-0.175227000
5	-1.807887000	-1.692778000	-0.271445000
5	0.662577000	-1.608157000	1.025906000
5	1.807895000	0.993261000	0.104079000
5	0.444833000	1.426300000	-0.895626000
5	0.862144000	-1.582294000	-1.080085000
5	1.479130000	0.096344000	-1.356142000
5	-1.925283000	-0.295522000	-1.165264000
1	-0.918373000	0.279795000	2.692989000
1	-3.125129000	-0.031558000	1.124228000
1	1.743656000	0.106736000	2.518661000
1	-1.877413000	1.978955000	-0.230006000
1	1.163322000	-2.394027000	1.768238000
1	2.757262000	1.698795000	0.201006000
1	0.425859000	2.442056000	-1.511859000
1	1.495949000	-2.351751000	-1.732600000
1	2.197991000	0.164318000	-2.299944000
1	-2.844226000	0.006389000	-1.846583000
5	-0.269585000	0.045243000	-1.822660000
1	-0.384121000	0.352055000	-2.968182000
5	1.955135000	-0.745504000	0.096796000
1	3.012944000	-1.278160000	0.190647000
5	-0.374515000	-2.525699000	-0.146294000
1	-0.263752000	-3.703636000	-0.150715000
5	-0.861783000	-1.659332000	-1.638274000
1	-1.075608000	-2.262249000	-2.633510000

$[B_{17}H_{16}]^{-}$_ap2_MeCN E = -432.262818236 Ha

5	0.296551000	1.427786000	0.890699000
5	-1.163799000	-1.696129000	1.202480000
5	-2.111621000	-0.364344000	0.590081000
5	1.241704000	0.075868000	1.465183000
1	0.164781000	2.461156000	1.446618000
1	-1.488944000	-2.289001000	2.178973000
5	-0.586831000	-0.017320000	1.549573000
5	-1.182540000	0.976202000	-0.156745000
5	-2.044434000	-1.865642000	-0.297669000
5	0.634924000	-1.566652000	0.985966000
5	1.853199000	1.012971000	0.116895000
5	0.417563000	1.151418000	-0.748184000
5	0.835349000	-1.567701000	-1.055746000
5	1.531088000	0.080931000	-1.373593000
5	-1.954161000	-0.349643000	-1.148894000
1	-0.917500000	0.280415000	2.655030000
1	-3.098794000	0.012275000	1.131419000
1	1.767783000	0.121568000	2.527335000
1	-1.805440000	1.985848000	-0.263799000
1	-2.986330000	-2.581739000	-0.385613000
1	1.149190000	-2.344607000	1.729490000
1	2.758185000	1.771071000	0.159174000
1	1.470943000	-2.323554000	-1.722787000
1	2.219774000	0.219616000	-2.322777000
1	-2.814297000	0.056243000	-1.859192000
5	-0.284705000	-0.001554000	-1.803416000
1	-0.358067000	0.409714000	-2.919090000
5	1.984836000	-0.734310000	0.103198000
1	3.041017000	-1.266438000	0.194432000
5	-0.423548000	-2.502856000	-0.155239000
1	-0.219354000	-3.672958000	-0.149782000
5	-0.910638000	-1.671451000	-1.610774000
1	-1.036942000	-2.231953000	-2.649525000

$[B_{17}H_{16}]^{-}$_eq_MeCN E = -432.297950034 Ha

5	0.278847000	1.416636000	0.863112000
5	-1.157226000	-1.693232000	1.232016000
5	-2.094014000	-0.346241000	0.616358000
5	1.215369000	0.069580000	1.478729000
1	0.005155000	2.380649000	1.491369000
1	-1.368850000	-2.236247000	2.261286000
5	-0.478613000	-0.104107000	1.182350000
5	-1.112368000	0.950260000	-0.255366000
5	-2.062795000	-1.866202000	-0.263047000
5	0.656355000	-1.593338000	0.906443000
5	1.832143000	1.027278000	0.141564000
5	0.488891000	1.448563000	-0.901997000
5	0.825893000	-1.549411000	-1.042295000
5	1.512621000	0.128468000	-1.338862000
5	-1.948689000	-0.362120000	-1.154976000
1	-3.017611000	0.134598000	1.177473000
1	1.654320000	0.009147000	2.575447000
1	-1.819996000	1.903305000	-0.260074000
1	-3.012342000	-2.569008000	-0.354144000
1	1.138012000	-2.350472000	1.683418000
1	2.778054000	1.733195000	0.245333000
1	0.449240000	2.479229000	-1.486208000
1	1.464213000	-2.317470000	-1.692855000
1	2.236933000	0.202851000	-2.275894000
1	-2.840447000	0.035792000	-1.826996000
5	-0.271698000	0.028916000	-1.763659000
1	-0.384940000	0.341241000	-2.908272000
5	1.994589000	-0.716681000	0.087787000
1	3.044328000	-1.251087000	0.220041000
5	-0.442922000	-2.527473000	-0.165602000
1	-0.245704000	-3.695551000	-0.121804000
5	-0.922234000	-1.680338000	-1.591845000
1	-1.071656000	-2.253558000	-2.619840000

$Li_2[B_2H_2]$_MeCN E = -65.9199338927 Ha

5	0.121911000	0.018464000	-0.038680000
5	1.371085000	-0.130635000	-0.761327000
1	2.392998000	-0.244212000	-1.354863000
1	-0.900539000	0.137897000	0.552737000
3	-0.370130000	-0.496174000	-2.220135000
3	1.837238000	0.747313000	1.309495000

$\{Li_2[B_2H]\}^{+}$_MeCN E = -65.1026054635 Ha

5	-0.132362000	-0.234811000	-0.000214000
5	1.008839000	0.834432000	-0.140313000
1	1.823222000	1.674776000	-0.253650000
3	-1.798416000	-1.715929000	0.197637000
3	1.671962000	-1.707890000	0.259376000

$Li_2[B_3H_3]$_MeCN E = -91.3972239722 Ha

5	4.245291000	1.130537000	1.473249000
5	4.102352000	1.046832000	-0.093805000
5	2.807473000	0.964576000	0.821446000
1	1.609020000	0.855146000	0.934919000
1	4.886310000	1.239341000	2.487326000
1	4.549974000	1.033707000	-1.211942000
3	2.698496000	1.073903000	3.060262000
3	2.295339000	0.795300000	-1.355652000

$\{Li_2[B_3H_2]\}^{+}$_MeCN E = -90.6315938884 Ha

5	4.258771000	1.127470000	1.567331000
5	4.097753000	1.045403000	-0.186192000
5	2.990160000	0.965714000	0.805263000
1	5.202697000	1.245977000	2.261013000
1	4.897886000	1.087793000	-1.048753000
3	2.264479000	1.063361000	3.038398000
3	1.873488000	0.748478000	-1.256175000

$Li_2[B_4H_4]$_MeCN E = -116.843807351 Ha

5	2.486946000	0.147439000	2.319492000
5	2.837920000	0.838554000	0.931855000
1	3.056211000	1.912447000	0.430380000
5	2.507913000	-0.675428000	0.495329000
5	3.552871000	-0.872517000	1.676112000
1	1.903395000	0.335317000	3.357070000
1	1.919378000	-1.276117000	-0.367756000
1	4.505682000	-1.533841000	2.003768000
3	2.128295000	2.334978000	2.408609000
3	3.555932000	-2.624917000	0.317833000

$\{Li_2[B_4H_3]\}^{+}$_MeCN E = -116.110132545 Ha

5	2.592793000	0.199981000	2.653051000
5	2.571101000	0.637704000	1.053582000
1	2.431485000	1.676728000	0.482380000
5	2.812187000	-0.708596000	0.079686000
5	2.789021000	-0.901630000	1.598778000
1	2.494884000	0.664807000	3.738140000
1	2.872594000	-0.931581000	-1.074776000
3	2.234034000	2.519866000	2.415390000
3	3.150762000	-3.037524000	0.622693000

$Li_2[B_5H_5]$_MeCN E = -142.336473283 Ha

5	4.469625000	-1.152196000	0.777111000
5	5.039239000	-1.300549000	-0.745829000
5	2.953647000	-0.458975000	0.548701000
5	3.386387000	-1.563913000	-0.572609000
5	4.131260000	0.059774000	-0.493746000
1	2.779653000	-2.403688000	-1.187747000
1	4.917086000	-1.434783000	1.859698000
1	4.287542000	1.153028000	-0.950934000
1	2.010647000	-0.089102000	1.183163000
3	1.200425000	-1.703613000	-0.034618000
1	5.973614000	-1.725554000	-1.358183000
3	6.480356000	-2.175195000	0.708538000

$\{Li_2[B_5H_4]\}^{+}$_ap_MeCN E = -141.510542071 Ha

5	4.634912000	-1.239912000	0.880843000
5	4.864620000	-1.379376000	-0.652739000
5	2.996762000	-0.780697000	0.640131000
5	3.316136000	-2.072151000	-0.165626000
5	3.929290000	0.068539000	-0.269329000
1	2.932076000	-3.023424000	-0.753699000
1	5.164431000	-1.342977000	1.951264000
1	4.097714000	1.023845000	-0.944695000
3	1.525175000	-0.490453000	-1.091503000
1	5.371127000	-1.574554000	-1.704354000
3	6.786592000	-1.894504000	0.660090000

$\{Li_2[B_5H_4]\}^{+}$_eq_MeCN E = -141.544416029 Ha

5	4.454428000	-1.425281000	0.724039000
5	5.077446000	-1.359928000	-0.731799000
5	2.733508000	-0.307406000	0.278705000
5	3.405180000	-1.346953000	-0.576045000
5	4.196739000	0.082973000	-0.548198000
1	4.518373000	-1.573892000	1.897356000
1	4.556942000	1.201235000	-0.653735000
1	1.991257000	0.255258000	0.999325000
3	1.244561000	-2.116239000	0.372653000
1	5.919300000	-1.760699000	-1.470592000
3	6.752093000	-2.040145000	0.629583000

$Li_2[B_6H_6]$_conf1_MeCN E = -167.858188199 Ha

3	1.539542000	1.578435000	1.723381000
5	1.292672000	0.042367000	-0.001054000
1	2.479497000	0.180251000	0.090667000
5	-0.022828000	1.168837000	0.034903000
5	0.038220000	-0.128708000	1.181508000
5	0.124794000	0.048409000	-1.240945000
5	-1.129661000	-0.122624000	-0.058385000
5	0.185857000	-1.249063000	-0.094308000
1	-2.316527000	-0.260504000	-0.150022000

1	0.113950000	0.076174000	-2.438622000
1	0.233801000	-2.440087000	-0.216190000
1	0.049037000	-0.156484000	2.379087000
1	-0.070765000	2.359748000	0.156817000
3	-1.376258000	-1.658917000	-1.782863000

$\{Li_2[B_6H_5]\}^+$_conf1_MeCN E = -167.044102828 Ha

3	1.456350000	1.443025000	2.074684000
5	1.410377000	0.101341000	-0.020571000
1	2.578170000	0.213079000	0.133609000
5	0.019192000	1.297803000	0.014364000
5	0.051760000	-0.100920000	0.996079000
5	0.234585000	0.173229000	-1.132977000
5	-1.117494000	-0.052068000	-0.229409000
5	0.242062000	-1.219741000	-0.263140000
1	-2.292755000	-0.172692000	-0.347492000
1	0.298167000	-2.396614000	-0.410402000
1	-0.028921000	-0.229708000	2.179275000
1	-0.046136000	2.464995000	0.195331000
3	-1.777978000	-2.160067000	-1.166756000

$Li_2[B_6H_6]$_conf0_MeCN E = -167.857182107 Ha

3	1.701758000	1.643263000	1.628782000
5	1.214805000	-0.013345000	0.095460000
1	2.412115000	0.027789000	0.146045000
5	0.000373000	1.219868000	0.133714000
5	-0.000941000	-0.033329000	1.314487000
5	0.005589000	0.049145000	-1.122255000
5	-1.210090000	0.029484000	0.090749000
5	0.004142000	-1.203940000	0.052435000
1	-2.407907000	-0.015668000	0.132012000
1	0.007989000	0.088435000	-2.314007000
1	-0.058787000	-2.401554000	0.057605000
1	-0.001185000	-0.070493000	2.512458000
1	0.062781000	2.414297000	0.221023000
3	-1.730643000	-1.733951000	1.470993000

$\{Li_2[B_6H_5]\}^+$_conf0_MeCN E = -167.046347916 Ha

3	1.655339000	1.605715000	1.862479000
5	1.276382000	-0.016384000	0.050599000
1	2.458268000	0.005875000	0.104697000
5	0.000053000	1.281694000	0.091180000
5	-0.000573000	-0.029520000	1.179577000
5	0.006616000	0.044380000	-0.965535000
5	-1.270093000	0.034401000	0.045153000
5	0.004779000	-1.263264000	0.004167000
1	-2.452743000	0.008781000	0.086423000
1	-0.042025000	-2.445952000	0.008870000
1	0.002551000	-0.066911000	2.374313000
1	0.045997000	2.460878000	0.181676000
3	-1.692543000	-1.708128000	1.709909000

$Li_2[B_7H_7]$_MeCN E = -193.318267060 Ha

5	8.293540000	19.096675000	4.103677000
1	9.085311000	19.014917000	5.005821000
5	6.941164000	20.015633000	4.187364000
1	6.650851000	20.666553000	5.156360000
5	6.132897000	19.862222000	2.770400000
1	5.095646000	20.378160000	2.444460000
5	6.989589000	18.846444000	1.807737000
1	6.693804000	18.511206000	0.702272000
5	8.326742000	18.373241000	2.633696000
1	9.156525000	17.623111000	2.189854000
5	7.916431000	20.133211000	2.666461000
1	8.523377000	21.067598000	2.228618000
5	6.754049000	18.340694000	3.522626000
1	6.151086000	17.410629000	3.975029000
3	5.022009000	21.202893000	4.210999000
3	10.149382000	17.834499000	3.854983000

$\{Li_2[B_7H_6]\}^+$_ap_MeCN E = -192.532570768 Ha

5	8.305986000	19.062653000	4.138582000
1	9.079448000	18.989531000	5.039023000
5	6.905533000	20.004460000	4.227636000
1	6.626767000	20.637968000	5.194436000
5	6.077133000	19.853492000	2.770220000
1	5.057505000	20.363759000	2.432390000
5	6.971583000	18.820266000	1.768203000
1	6.694335000	18.512199000	0.664023000
5	8.348360000	18.327321000	2.624799000
1	9.171814000	17.600860000	2.168018000
5	7.857449000	20.048381000	2.715468000
1	8.451541000	20.973802000	2.272999000
5	6.879715000	18.526119000	3.416181000
3	4.989910000	21.397265000	4.207367000
3	10.314238000	17.848982000	3.845985000

$\{Li_2[B_7H_6]\}^+$_eq_MeCN E = -192.506372318 Ha

5	8.186241000	19.145487000	4.024403000
5	6.941848000	20.069083000	4.229286000
1	6.609674000	20.736875000	5.159631000
5	6.142009000	19.891943000	2.786379000
1	5.112117000	20.417336000	2.472049000
5	7.041497000	18.846090000	1.866547000
1	6.885641000	18.413938000	0.773285000
5	8.421672000	18.360615000	2.725977000
1	9.122772000	17.609766000	2.130432000
5	7.871471000	20.213464000	2.620548000
1	8.495612000	21.125228000	2.190976000
5	6.654752000	18.366300000	3.521960000
1	6.070744000	17.446190000	3.988752000
3	4.826210000	21.191837000	4.251255000
3	10.414831000	17.528617000	3.713056000

$Li_2[B_8H_8]$_MeCN E = -218.764986129 Ha

5	1.598567000	4.455509000	2.294757000
5	1.631082000	4.881114000	3.937567000
1	1.385028000	3.317167000	1.942418000
1	1.459980000	4.165215000	4.886387000
5	0.583958000	5.912667000	2.898747000
5	3.052908000	5.424270000	2.975662000
5	1.080676000	5.828516000	1.267488000
5	2.660860000	5.516001000	1.316737000
5	1.942025000	6.451173000	3.951039000
5	2.077945000	6.884512000	2.315537000
1	-0.562913000	6.136684000	3.152209000
1	4.181201000	5.198159000	3.300758000
1	0.325354000	5.985186000	0.358313000
1	3.471601000	5.363145000	0.456116000
1	2.053295000	7.162491000	4.912214000
1	2.313066000	8.024344000	1.982683000
3	1.266869000	2.632435000	3.701621000
3	2.332260000	8.697710000	3.752019000

$\{Li_2[B_8H_7]\}^+$_ap_MeCN E = -217.961262540 Ha

5	1.592200000	4.385557000	2.341928000
5	1.638026000	4.863795000	3.937703000
1	1.413934000	3.268058000	1.949188000
1	1.518946000	4.188952000	4.916331000
5	0.578902000	5.900217000	2.923776000
5	2.992761000	5.446784000	2.927376000
5	1.213261000	5.792730000	1.407478000
5	2.669627000	5.521662000	1.143199000
5	1.939502000	6.467252000	3.950408000
5	2.073517000	6.953088000	2.361686000
1	-0.579832000	6.116129000	3.072811000
1	4.153530000	5.227818000	3.058073000
1	3.616048000	5.349753000	0.467910000
1	2.071537000	7.123029000	4.940614000
1	2.313013000	8.063899000	1.983076000
3	1.055327000	2.586115000	3.806458000
3	2.268111000	8.796272000	3.855944000

$\{Li_2[B_8H_7]\}^+$_eq_MeCN E = -217.980272934 Ha

5	1.463437000	4.456743000	2.290812000
5	1.601831000	4.869832000	3.959622000
1	1.241294000	3.326764000	1.969539000
1	1.467619000	4.091469000	4.851921000
5	0.553203000	5.908027000	2.924155000
5	2.843618000	5.477608000	2.955009000
5	1.139703000	5.810610000	1.277190000
5	2.800145000	5.497809000	1.391648000
5	1.903997000	6.478959000	3.971800000
5	1.929492000	6.939756000	2.309606000
1	-0.586646000	6.119864000	3.192186000
1	0.567353000	5.925601000	0.246012000
1	3.443130000	5.384696000	0.409379000
1	2.057612000	7.240827000	4.874985000
1	2.133069000	8.078943000	2.008834000
3	1.452498000	2.462216000	3.705925000
3	2.661208000	8.768414000	3.762892000

$Li_2[B_9H_9]$_MeCN E = -244.241430062 Ha

3	1.703130000	-0.573447000	6.190603000
5	1.639582000	1.663174000	6.871643000
5	0.667392000	0.731134000	7.907587000
5	1.621081000	0.712392000	9.590124000
1	1.657259000	1.690006000	5.673086000
1	-0.005081000	-0.149539000	7.442132000
1	1.628668000	-0.161301000	10.402065000
5	2.622274000	0.775730000	7.936561000
5	2.581239000	2.546939000	7.976502000
5	0.626636000	2.502439000	7.947740000
5	0.179189000	1.574504000	9.299069000
5	3.030081000	1.639860000	9.341376000
1	3.347274000	-0.073293000	7.491139000
5	1.579856000	2.488180000	9.630577000
1	3.265526000	3.447657000	7.570588000
1	-0.085774000	3.371660000	7.521410000
1	-0.854960000	1.536728000	9.881889000
1	4.047522000	1.649337000	9.954167000
1	1.548116000	3.322919000	10.481951000
3	1.594275000	3.924329000	6.284065000

$\{Li_2[B_9H_8]\}^+$_ap_MeCN E = -243.425249665 Ha

3	1.629299000	-0.495127000	5.902475000
5	1.786473000	1.780674000	6.860783000
5	0.961884000	0.702946000	8.060023000
5	1.354758000	0.734055000	10.000522000
1	1.604984000	1.827840000	5.676844000
1	0.361949000	-0.244319000	7.638572000
1	1.114816000	-0.076662000	10.825509000
5	2.880659000	0.816063000	7.586578000
5	2.686909000	2.528647000	8.119309000
5	0.814896000	2.570492000	8.015975000
5	0.187413000	1.692981000	9.348401000
5	2.638656000	1.102114000	9.141791000
1	3.542073000	0.000193000	7.030616000
5	1.675801000	2.488755000	9.653814000
1	3.473580000	3.413540000	8.094977000
1	0.061288000	3.423262000	7.629113000
1	-0.953172000	1.714727000	9.663137000
1	1.967240000	3.354743000	10.409254000
3	0.556256000	3.635146000	5.782774000

$\{Li_2[B_9H_8]\}^+$_eq_MeCN E = -243.435127211 Ha

3	1.704499000	-0.680749000	6.206962000
5	1.641843000	1.726555000	6.888632000
5	0.662428000	0.757660000	7.918952000
5	1.621974000	0.656564000	9.590861000
1	1.660883000	1.764110000	5.693516000
1	-0.010319000	-0.125380000	7.473168000
1	1.628215000	-0.224076000	10.375604000
5	2.628639000	0.800846000	7.949250000
5	2.661022000	2.559329000	7.965956000
5	0.553807000	2.512210000	7.931601000
5	0.148390000	1.583769000	9.320196000
5	3.061838000	1.645924000	9.366138000
1	3.351636000	-0.052665000	7.524926000
5	1.587930000	2.336379000	9.445246000
1	3.337961000	3.465736000	7.597664000
1	-0.151818000	3.388721000	7.543635000
1	-0.843597000	1.555129000	9.957811000
1	4.033295000	1.657640000	10.035278000
3	1.566525000	3.968764000	6.126916000

$Li_2[B_{10}H_{10}]$_MeCN E = -269.748372412 Ha

3	4.570504000	6.232865000	3.203916000
5	0.333622000	5.339559000	5.487075000
5	3.076539000	4.585337000	6.147550000
5	1.773642000	5.829582000	6.245533000
5	2.520579000	3.580133000	4.714122000
5	3.108612000	6.064642000	5.091701000
5	0.985078000	4.412800000	4.238488000
5	1.407910000	6.181304000	4.500400000
5	3.859931000	4.602982000	4.657007000
5	2.550271000	5.066767000	3.672785000
5	1.360282000	4.074618000	5.982420000
1	3.665328000	4.303028000	7.143969000
1	0.270297000	3.868062000	3.456594000
1	3.758637000	7.073234000	5.103583000
1	-0.789783000	5.554571000	5.829495000
1	1.711649000	6.482095000	7.250842000
1	2.634912000	2.422941000	4.455342000
1	4.996345000	4.425907000	4.335949000
1	2.740923000	5.245219000	2.500909000
1	1.071375000	7.177763000	3.940845000
1	0.946867000	3.269205000	6.770717000
3	0.093129000	5.055461000	7.750629000

$\{Li_2[B_{10}H_9]\}^+$_ap_MeCN E = -268.914499212 Ha

3	4.740883000	6.159332000	3.298508000
5	0.383749000	5.371300000	5.233952000
5	2.852674000	4.644868000	6.197139000
5	1.587062000	5.931258000	6.175177000
5	2.419453000	3.590059000	4.737269000
5	3.015278000	6.107330000	5.109192000
5	0.953757000	4.406940000	4.058831000
5	1.395116000	6.252832000	4.323058000
5	3.753593000	4.615100000	4.784081000
5	2.576100000	5.063293000	3.655779000
5	1.157764000	4.109039000	5.908872000
1	3.390248000	4.372765000	7.231741000
1	0.286074000	3.867045000	3.243983000
1	3.687929000	7.091905000	5.162930000
1	1.412747000	6.602397000	7.142551000
1	2.562140000	2.431436000	4.527163000
1	4.907800000	4.399111000	4.594874000
1	2.892015000	5.197281000	2.511103000
1	1.100453000	7.228089000	3.720417000
1	0.648898000	3.349926000	6.674148000
3	1.712699000	4.502201000	8.359610000

$\{Li_2[B_{10}H_9]\}^+$_eq_MeCN E = -268.934766905 Ha

3	4.864051000	6.174843000	3.186670000
5	0.297496000	5.379768000	5.501877000
5	3.098892000	4.625293000	6.133338000
5	1.785958000	5.853728000	6.296394000
5	2.520525000	3.595750000	4.694565000
5	3.136173000	6.095546000	5.058864000
5	0.949889000	4.372603000	4.244740000
5	1.475467000	5.988672000	4.603686000
5	3.860054000	4.623695000	4.623934000
5	2.555471000	5.072312000	3.629389000
5	1.354963000	4.123215000	5.985545000
1	3.712578000	4.361687000	7.113498000
1	0.228809000	3.829721000	3.484234000

1	3.708083000	7.139540000	5.076332000
1	-0.818172000	5.606961000	5.829588000
1	1.741379000	6.523402000	7.273720000
1	2.638442000	2.445021000	4.432576000
1	4.990721000	4.406608000	4.321257000
1	2.651641000	5.263148000	2.458919000
1	0.907145000	3.331426000	6.763995000
3	-0.084289000	4.857376000	7.825905000

$Li_2[B_{11}H_{11}]$_MeCN E = -295.189448901 Ha

5	1.802065000	16.614537000	15.248607000
5	2.555538000	18.175395000	15.310389000
5	0.613800000	15.515608000	15.884957000
5	2.711467000	17.411863000	16.782770000
5	0.914014000	18.364852000	15.120722000
5	-0.159793000	16.919201000	15.455159000
5	1.659565000	15.954779000	17.093600000
5	1.659658000	18.842081000	16.655603000
5	-0.043176000	16.498868000	17.182794000
5	1.247296000	17.535303000	17.781972000
5	0.010748000	18.196139000	16.657947000
1	2.329506000	16.033966000	14.347878000
1	3.419488000	18.611144000	14.623944000
1	0.393489000	14.392388000	15.535370000
1	3.771281000	17.319703000	17.343029000
1	0.460802000	19.010933000	14.227392000
1	-1.115218000	16.955962000	14.720758000
1	2.252383000	15.136352000	17.725410000
1	1.901145000	19.935135000	17.091238000
1	-0.907109000	16.098498000	17.897370000
1	1.336022000	17.742326000	18.962371000
1	-0.894611000	18.920919000	16.927096000
3	2.995120000	18.907155000	18.623091000
3	-0.980034000	15.078852000	14.282136000

$\{Li_2[B_{11}H_{10}]\}^+$_ap_MeCN E = -294.378270974 Ha

5	1.667804000	16.750816000	15.460928000
5	2.573813000	18.177189000	15.303019000
5	0.583477000	15.504657000	15.887115000
5	2.762576000	17.425826000	16.784675000
5	0.930832000	18.395006000	15.095926000
5	-0.213326000	16.903536000	15.469781000
5	1.660193000	15.903466000	17.094447000
5	1.693238000	18.843245000	16.646167000
5	-0.040622000	16.482284000	17.193389000
5	1.283562000	17.519383000	17.778064000
5	0.022588000	18.192891000	16.653823000
1	3.438178000	18.501304000	14.566067000
1	0.430313000	14.405751000	15.454696000
1	3.812108000	17.289058000	17.323752000
1	0.457800000	18.995950000	14.195868000
1	-1.145113000	16.966184000	14.726970000
1	2.291093000	15.101383000	17.689163000
1	1.919605000	19.937035000	17.074565000
1	-0.890668000	16.098649000	17.924441000
1	1.390029000	17.685476000	18.959057000
1	-0.874394000	18.931115000	16.891642000
3	2.897871000	19.101450000	18.774639000
3	-1.047018000	15.026341000	14.185533000

$\{Li_2[B_{11}H_{10}]\}^+$_eq1_MeCN E = -294.393271285 Ha

5	1.733790000	16.551092000	15.167372000
5	2.736060000	18.102323000	15.288215000
5	0.568586000	15.486357000	15.870451000
5	2.603984000	17.317639000	16.620082000
5	1.002663000	18.305110000	15.185728000
5	-0.144350000	16.946350000	15.491167000
5	1.728974000	15.907534000	16.995913000
5	1.732158000	18.826836000	16.677938000
5	0.022641000	16.477099000	17.212340000
5	1.350623000	17.484254000	17.786238000
5	0.086254000	18.190877000	16.713443000
1	2.245932000	16.038757000	14.226979000
1	3.436854000	18.578081000	14.474127000
1	0.287159000	14.381171000	15.528811000
1	0.724544000	18.938927000	14.222432000
1	-1.121182000	17.028614000	14.801966000
1	2.415719000	15.060114000	17.467791000
1	2.056416000	19.909602000	17.055815000
1	-0.813367000	16.081922000	17.953827000
1	1.545907000	17.635503000	18.954761000
1	-0.791800000	18.921257000	17.031187000
3	2.000813000	19.584919000	19.054847000
3	-1.246213000	15.097918000	14.357143000

$\{Li_2[B_{11}H_{10}]\}^+$_eq2_MeCN E = -294.390642608 Ha

5	1.832971000	16.646596000	15.271441000
5	2.564632000	18.208163000	15.321447000
5	0.477224000	15.505816000	15.834302000
5	2.700703000	17.415927000	16.794449000
5	0.896450000	18.338232000	15.139967000
5	0.005671000	16.968140000	15.596199000
5	1.610426000	16.014011000	17.046428000
5	1.667701000	18.875003000	16.671947000
5	-0.048451000	16.547759000	17.286723000
5	1.272827000	17.569009000	17.823171000
5	0.016522000	18.247221000	16.729645000
1	2.287263000	16.011255000	14.376806000
1	3.397627000	18.656906000	14.616314000
1	0.368825000	14.355721000	15.581682000
1	3.768133000	17.306601000	17.321387000
1	0.482114000	18.864551000	14.158218000
1	2.165803000	15.062791000	17.487150000
1	1.926281000	19.965051000	17.087791000
1	-0.927201000	16.141677000	17.964028000
1	1.389909000	17.775117000	18.994845000
1	-0.923994000	18.923714000	16.971767000
3	3.080455000	18.986239000	18.679844000
3	-0.963227000	14.830498000	14.005293000

$Li_2[B_{12}H_{12}]$_MeCN E = -320.749960793 Ha

5	-0.782012000	-0.929927000	1.167789000
5	0.382290000	0.332676000	1.603693000
1	-1.361522000	-1.554000000	2.020586000
1	0.626555000	0.560701000	2.756942000
5	0.934413000	-1.168333000	0.835326000
1	1.571714000	-1.975210000	1.455689000
5	-0.274955000	-1.663559000	-0.361304000
1	-0.489951000	-2.808691000	-0.599599000
5	-1.164264000	0.775793000	0.878171000
1	-2.026498000	1.320171000	1.511218000
5	-1.567559000	-0.451445000	-0.338451000
1	-2.712218000	-0.745971000	-0.549410000
5	1.212049000	-0.842558000	-0.874051000
1	2.056669000	-1.414582000	-1.487108000
5	1.620080000	0.388005000	0.343784000
1	2.747313000	0.675023000	0.594110000
5	-0.894961000	1.108531000	-0.836156000
1	-1.551993000	1.908585000	-1.422861000
5	0.317144000	1.594560000	0.367580000
1	0.519431000	2.736545000	0.631789000
5	0.827483000	0.865183000	-1.165860000
1	1.398563000	1.498657000	-1.996759000
5	-0.340515000	-0.397668000	-1.602631000
1	-0.601057000	-0.651960000	-2.735601000
3	-3.187398000	-0.393638000	1.411198000
3	0.634614000	-1.448870000	3.180483000

$\{Li_2[B_{12}H_{11}]\}^+$_conf1_MeCN E = -319.930565862 Ha

5	-0.793306000	-0.956581000	1.194336000
5	0.390016000	0.300507000	1.612008000
1	-1.362020000	-1.632391000	1.994447000
1	0.635956000	0.539069000	2.757756000
5	0.973150000	-1.196392000	0.852091000
1	1.572848000	-2.043514000	1.434804000
5	-0.219622000	-1.430076000	-0.324472000
5	-1.165674000	0.753920000	0.885783000
1	-2.012593000	1.308349000	1.522886000
5	-1.596720000	-0.447687000	-0.349254000
1	-2.711910000	-0.796864000	-0.580639000
5	1.257128000	-0.846917000	-0.900462000
1	2.062641000	-1.453572000	-1.517041000
5	1.638875000	0.370143000	0.346636000
1	2.761852000	0.652668000	0.597588000
5	-0.892306000	1.107671000	-0.836901000
1	-1.546716000	1.906978000	-1.417306000
5	0.321522000	1.554856000	0.372247000
1	0.525952000	2.690243000	0.644398000
5	0.842526000	0.863862000	-1.170152000
1	1.412065000	1.496319000	-1.995266000
5	-0.337195000	-0.382612000	-1.645104000
1	-0.598970000	-0.678950000	-2.758905000
3	-3.443311000	-0.152717000	1.298243000
3	0.669177000	-1.399605000	3.370444000

$\{Li_2[B_{12}H_{11}]\}^+$_conf2_MeCN E = -319.932562087 Ha

5	-0.740923000	-0.989598000	1.067954000
5	0.294714000	0.162399000	1.752366000
1	0.531911000	0.311910000	2.908126000
5	0.900460000	-1.328487000	0.896889000
1	1.527932000	-2.135577000	1.506163000
5	-0.311747000	-1.800093000	-0.360214000
1	-0.518377000	-2.935720000	-0.613184000
5	-1.286622000	0.612725000	1.023666000
1	-2.171935000	1.082223000	1.663579000
5	-1.663350000	-0.598845000	-0.285765000
1	-2.794180000	-0.904136000	-0.492582000
5	1.161742000	-0.904236000	-0.809353000
1	2.023289000	-1.426316000	-1.433002000
5	1.521487000	0.282991000	0.472964000
1	2.645768000	0.581892000	0.750994000
5	-0.988440000	0.997649000	-0.684928000
1	-1.664706000	1.812281000	-1.241863000
5	0.199397000	1.469233000	0.550392000
1	0.376144000	2.597571000	0.863580000
5	0.729966000	0.803054000	-1.009913000
1	1.288811000	1.487689000	-1.799687000
5	-0.400842000	-0.459118000	-1.530226000
1	-0.655386000	-0.662363000	-2.669376000
3	-3.402044000	1.299023000	-0.289018000
3	2.651868000	-0.484135000	2.530417000

$Li_2[B_{13}H_{13}]$_MeCN E = -346.119377560 Ha

5	0.086542000	1.455103000	0.691524000
5	-0.025924000	-1.207540000	0.844128000
5	-1.352419000	0.204917000	1.232731000
5	1.389152000	0.086959000	1.171285000
1	0.142656000	2.556501000	1.146852000
1	-0.054095000	-2.260308000	1.403696000
5	0.031789000	0.193615000	2.043578000
5	-1.453458000	1.007216000	-0.340226000
5	-1.530778000	-0.746242000	-0.244619000
5	1.438263000	-0.857443000	-0.301235000
5	1.519521000	0.886799000	-0.403564000
5	0.034058000	1.448086000	-1.127570000
5	-0.087911000	-1.372955000	-0.966276000
5	0.832966000	-0.033518000	-1.708276000
5	-0.927421000	0.036045000	-1.678292000
1	0.159764000	0.258717000	3.233259000
1	-2.417068000	0.281754000	1.802853000
1	2.409466000	0.071877000	1.790390000
1	-2.411256000	1.710994000	-0.413900000
1	-2.538314000	-1.379999000	-0.253525000
1	2.395377000	-1.579077000	-0.385694000
1	2.534159000	1.494668000	-0.534720000
1	0.064180000	2.474467000	-1.729221000
1	-0.129203000	-2.461481000	-1.471234000
1	1.396004000	-0.158991000	-2.759999000
1	-1.528142000	-0.002513000	-2.704081000
3	-1.733253000	0.375136000	3.544640000
3	1.794942000	-2.171512000	-2.245689000

$\{Li_2[B_{13}H_{12}]\}^+$_ap_MeCN E = -345.367335785 Ha

5	0.160157000	1.446790000	0.669562000
5	0.113193000	-1.451448000	0.676773000
5	-2.251646000	0.191780000	1.289854000
5	1.044359000	-0.007435000	1.202715000
1	0.120747000	2.442718000	1.328765000
1	0.093625000	-2.464201000	1.288795000
5	-0.703338000	0.002555000	1.243503000
5	-1.312221000	0.900560000	-0.162461000
5	-1.359006000	-0.866329000	-0.173743000
5	1.493902000	-0.880590000	-0.255751000
5	1.531920000	0.898026000	-0.256004000
5	0.062282000	1.479591000	-1.098918000
5	0.023334000	-1.407815000	-1.096769000
5	0.883413000	0.029454000	-1.667023000
5	-0.879916000	0.041666000	-1.636380000
1	-3.400248000	0.156676000	1.504039000
1	1.690628000	-0.001229000	2.206407000
1	-2.384781000	1.441727000	0.052055000
1	-2.412502000	-1.427532000	-0.085997000
1	2.499695000	-1.520601000	-0.340083000
1	2.552176000	1.498946000	-0.293841000
1	0.026869000	2.497717000	-1.700553000
1	0.000387000	-2.417271000	-1.733685000
1	1.489487000	-0.002106000	-2.698444000
1	-1.537161000	0.046544000	-2.620964000
3	0.273568000	1.433061000	3.161943000
3	2.060915000	-2.008693000	-2.400237000

$\{Li_2[B_{13}H_{12}]\}^+$_eq1_MeCN E = -345.368324066 Ha

5	0.167173000	1.353823000	0.783684000
5	-0.103049000	-1.513707000	0.595334000
5	-0.826986000	-0.028150000	1.283800000
5	0.961545000	-0.209891000	1.158188000
1	0.325659000	2.244708000	1.583192000
1	-0.182861000	-2.544228000	1.171227000
5	0.712860000	0.582538000	2.462800000
5	-1.360659000	1.029380000	-0.048275000
5	-1.514139000	-0.744615000	-0.169051000
5	1.340900000	-1.016677000	-0.335808000
5	1.512554000	0.760005000	-0.223372000
5	0.085030000	1.518273000	-0.972499000
5	-0.190196000	-1.344769000	-1.158264000
5	0.796108000	0.042409000	-1.652632000
5	-0.970547000	0.214698000	-1.556164000
1	0.533258000	1.045791000	3.519525000
1	-1.386563000	-0.021268000	2.344780000
1	-2.323677000	1.728600000	0.064009000
1	-2.587609000	-1.267066000	-0.119733000
1	2.281396000	-1.737762000	-0.474002000
1	2.563074000	1.303214000	-0.180117000
1	0.156242000	2.577879000	-1.494534000
1	-0.297376000	-2.298543000	-1.870358000
1	1.371532000	0.032920000	-2.700987000
1	-1.640804000	0.337101000	-2.525616000
3	-3.480133000	0.244688000	0.984406000
3	1.687396000	-2.049956000	-2.623110000

$\{Li_2[B_{13}H_{12}]\}^+$_eq2_MeCN E = -345.306653459 Ha

5	0.081263000	1.467150000	0.747857000
5	-0.020092000	-1.184854000	0.819646000
5	-1.356588000	0.211839000	1.237738000

5	1.397486000	0.101779000	1.168871000
1	0.134957000	2.585455000	1.140179000
1	-0.042415000	-2.241961000	1.362350000
5	0.035552000	0.176488000	2.045222000
5	-1.500530000	1.017976000	-0.340849000
5	-1.537966000	-0.741237000	-0.257448000
5	1.452539000	-0.838397000	-0.326546000
5	1.550518000	0.909917000	-0.412505000
5	0.018598000	1.240180000	-0.971957000
5	-0.082730000	-1.361530000	-0.982309000
5	0.857493000	-0.025190000	-1.746015000
5	-0.957040000	0.037625000	-1.704194000
1	0.157829000	0.213120000	3.231808000
1	-2.412545000	0.287477000	1.809377000
1	2.421040000	0.092396000	1.772406000
1	-2.410037000	1.769654000	-0.423433000
1	-2.541063000	-1.372871000	-0.261086000
1	2.415808000	-1.544933000	-0.395601000
1	2.512075000	1.584324000	-0.547541000
1	-0.137967000	-2.437497000	-1.501684000
1	1.372374000	-0.100697000	-2.812448000
1	-1.512592000	0.044191000	-2.746067000
3	-1.753963000	0.377058000	3.612194000
3	1.833416000	-2.430657000	-2.151930000

$\{Li_2[B_{13}H_{12}]\}^+$_eq3_MeCN E = -345.315067319 Ha

5	0.062113000	1.140683000	0.596074000
5	-0.011880000	-1.191639000	0.829197000
5	-1.382849000	0.245141000	1.251838000
5	1.402422000	0.065402000	1.176752000
1	-0.050233000	-2.208281000	1.441169000
5	0.022428000	0.196564000	2.060723000
5	-1.485002000	1.003368000	-0.372672000
5	-1.529327000	-0.745796000	-0.258440000
5	1.458592000	-0.855484000	-0.326832000
5	1.538523000	0.891973000	-0.426894000
5	0.031251000	1.444057000	-1.143160000
5	-0.082455000	-1.366975000	-0.972924000
5	0.837472000	-0.032137000	-1.729520000
5	-0.930187000	0.031506000	-1.700016000
1	0.172262000	0.270517000	3.237823000
1	-2.437995000	0.362839000	1.804186000
1	2.404284000	0.043274000	1.805208000
1	-2.404404000	1.745422000	-0.421390000
1	-2.528880000	-1.380254000	-0.196463000
1	2.419933000	-1.563516000	-0.392031000
1	2.516750000	1.550227000	-0.508600000
1	0.056433000	2.511832000	-1.653463000
1	-0.126160000	-2.463945000	-1.444581000
1	1.397046000	-0.141600000	-2.778757000
1	-1.526525000	0.002828000	-2.723006000
3	-1.786475000	0.406827000	3.655876000
3	1.859804000	-2.208061000	-2.320133000

$\{Li_2[B_{13}H_{12}]\}^+$_eq4_MeCN E = -345.302989340 Ha

5	0.109633000	1.458199000	0.710028000
5	-0.008287000	-1.205643000	0.846512000
5	-1.403260000	0.198470000	1.243145000
5	1.368393000	0.091756000	1.134644000
1	0.151340000	2.557256000	1.159284000
1	-0.043103000	-2.262816000	1.386767000
5	0.004643000	0.188096000	2.020587000
5	-1.480537000	1.039361000	-0.314778000
5	-1.558617000	-0.757852000	-0.231976000
5	1.450170000	-0.859580000	-0.338462000
5	1.536241000	0.904792000	-0.430702000
5	0.052811000	1.503346000	-1.110224000
5	-0.071819000	-1.401942000	-0.961871000
5	0.846658000	-0.015345000	-1.751680000
5	-0.799785000	0.062254000	-1.458444000
1	0.142143000	0.246635000	3.206118000
1	-2.453172000	0.265611000	1.829665000
1	2.382228000	0.078188000	1.755339000
1	-2.410554000	1.758157000	-0.446235000
1	-2.541975000	-1.409686000	-0.314804000
1	2.421281000	-1.556268000	-0.413314000
1	2.556101000	1.495386000	-0.556614000
1	0.001741000	2.513229000	-1.722199000
1	-0.191872000	-2.458119000	-1.497629000
1	1.349258000	-0.131352000	-2.822624000
3	-1.761828000	0.393369000	3.614540000
3	1.919907000	-2.381716000	-2.194176000

$Li_2[B_{14}H_{14}]$_MeCN E = -371.610850280 Ha

5	-1.689012000	-0.349164000	0.740782000
5	-1.121780000	1.284008000	0.734468000
5	0.576538000	1.621744000	0.746106000
5	1.701433000	0.298382000	0.764685000
5	1.127663000	-1.345371000	0.767850000
5	-0.575623000	-1.672211000	0.757028000
1	-1.791285000	2.058476000	1.362478000
1	0.912649000	2.596673000	1.337989000
1	2.714715000	0.491209000	1.355686000
1	1.805290000	-2.117237000	1.367443000
1	-0.926010000	-2.632819000	1.365215000
1	-2.694396000	-0.532544000	1.372272000
5	-1.638672000	0.542380000	-0.769404000
5	-0.328150000	1.682294000	-0.763476000
5	1.300529000	1.094070000	-0.747034000
5	1.617234000	-0.605354000	-0.737021000
5	0.317949000	-1.746592000	-0.741863000
5	-1.317542000	-1.168716000	-0.756541000
1	-0.514988000	2.690881000	-1.364634000
1	2.077889000	1.753417000	-1.382292000
1	2.583535000	-0.943148000	-1.365551000
1	0.520557000	-2.754747000	-1.340024000
1	-2.103664000	-1.836174000	-1.348971000
1	-2.611929000	0.878767000	-1.363597000
5	-0.008290000	-0.036335000	-1.535597000
5	0.003585000	-0.022200000	1.534986000
1	0.015313000	-0.036565000	-2.737691000
1	-0.018993000	-0.007699000	2.736921000
3	-2.046607000	0.707788000	2.876072000
3	2.123088000	0.368455000	-2.883860000

$\{Li_2[B_{14}H_{13}]\}^+$_ap_MeCN E = -370.797022597 Ha

5	-1.572790000	-0.403015000	0.959167000
5	-0.991159000	1.255400000	0.952401000
5	0.715847000	1.583261000	0.823098000
5	1.847485000	0.248395000	0.692959000
5	1.264154000	-1.410284000	0.694063000
5	-0.452936000	-1.731135000	0.833049000
1	-1.598802000	2.004732000	1.650858000
1	1.103547000	2.507889000	1.448173000
1	2.884543000	0.418491000	1.232601000
1	1.968994000	-2.184409000	1.241935000
1	-0.735347000	-2.685306000	1.470854000
1	-2.514184000	-0.603462000	1.660525000
5	-1.622786000	0.540628000	-0.525618000
5	-0.317613000	1.680284000	-0.611466000
5	1.300996000	1.090187000	-0.758118000
5	1.617918000	-0.612493000	-0.825790000
5	0.309489000	-1.747138000	-0.755583000
5	-1.316576000	-1.168953000	-0.605201000
1	-0.557902000	2.711927000	-1.141601000
1	2.030576000	1.775260000	-1.412371000
1	2.535145000	-0.935645000	-1.521629000
1	0.454099000	-2.746395000	-1.375954000
1	-2.161616000	-1.817178000	-1.124073000
1	-2.670593000	0.908692000	-0.966572000
5	-0.070831000	-0.014920000	-1.470475000
5	0.183012000	-0.091490000	1.326927000
1	-0.154087000	0.023324000	-2.662173000
3	-3.508702000	1.220331000	1.045373000
3	2.060136000	0.452390000	-3.029855000

$\{Li_2[B_{14}H_{13}]\}^+$_eq_MeCN E = -370.801500548 Ha

5	-1.565090000	-0.389326000	0.937349000
5	-0.990297000	1.254644000	0.946440000
5	0.711139000	1.604221000	0.830171000
5	1.557642000	0.210069000	0.600607000
5	1.259610000	-1.406722000	0.717202000
5	-0.459349000	-1.716455000	0.841575000
1	-1.626228000	2.029329000	1.598012000
1	1.147812000	2.502501000	1.464297000
1	1.998135000	-2.129562000	1.294087000
1	-0.753340000	-2.694326000	1.442077000
1	-2.548175000	-0.591498000	1.588873000
5	-1.594572000	0.549784000	-0.543984000
5	-0.308720000	1.717152000	-0.617913000
5	1.293288000	1.117304000	-0.791686000
5	1.618443000	-0.632196000	-0.850139000
5	0.331291000	-1.773551000	-0.746091000
5	-1.289112000	-1.163635000	-0.613513000
1	-0.564858000	2.737525000	-1.162277000
1	2.077786000	1.729607000	-1.444006000
1	2.565298000	-0.876297000	-1.529879000
1	0.462340000	-2.771738000	-1.371275000
1	-2.116035000	-1.810972000	-1.163739000
1	-2.627864000	0.922279000	-1.014999000
5	-0.081722000	-0.019482000	-1.471118000
5	0.132023000	-0.095962000	1.646246000
1	-0.139225000	0.018563000	-2.666348000
1	0.286179000	-0.109420000	2.819651000
3	-3.455749000	1.176174000	1.047282000
3	1.975661000	0.382447000	-3.160165000

$Li_2[B_{15}H_{15}]$_MeCN E = -397.048749535 Ha

5	0.027001000	6.745678000	13.107545000
5	-0.276310000	5.452152000	12.020429000
1	-1.144871000	5.497835000	11.196782000
5	1.168152000	6.369243000	11.881970000
1	1.376554000	7.101236000	10.956928000
5	-0.725982000	5.284073000	13.701578000
5	1.218338000	4.642154000	11.612617000
1	1.330072000	4.293744000	10.480702000
5	1.735395000	6.845008000	13.465179000
1	2.187150000	7.942274000	13.549317000
5	0.140464000	3.812949000	12.912584000
5	2.589817000	5.366165000	12.677034000
1	3.625997000	5.582597000	12.130710000
5	0.655330000	6.004866000	14.756130000
1	0.320184000	6.673646000	15.683142000
5	1.989702000	3.583247000	12.656489000
1	2.501516000	2.686777000	12.065277000
5	2.507643000	5.786302000	14.508843000
1	3.359243000	6.336319000	15.131282000
5	0.045753000	4.224857000	14.744793000
1	-0.718090000	3.750285000	15.523518000
5	2.812553000	4.112652000	14.105617000
1	3.888892000	3.649540000	14.353215000
5	1.671686000	4.489771000	15.331122000
1	1.897245000	4.309770000	16.493579000
5	1.367698000	3.196821000	14.243873000
1	1.367770000	2.051798000	14.595027000
1	-0.616294000	7.756036000	13.094340000
1	-1.889227000	5.356725000	13.939697000
1	-0.560042000	2.927583000	12.532435000
3	-0.701247000	7.565421000	10.968257000
3	2.945282000	2.543497000	15.927280000

$\{Li_2[B_{15}H_{14}]\}^+$_ap_MeCN E = -396.226005579 Ha

5	0.167540000	6.834329000	12.994138000
5	-0.141337000	5.514659000	11.881066000
1	-0.921171000	5.627014000	10.991694000
5	1.350723000	6.196214000	12.039301000
5	-0.668419000	5.393049000	13.552793000
5	1.367834000	4.599517000	11.551954000
1	1.512639000	4.311999000	10.413012000
5	1.900356000	6.869887000	13.463652000
1	2.366640000	7.957702000	13.481591000
5	0.169351000	3.865001000	12.820795000
5	2.744956000	5.300008000	12.715469000
1	3.783400000	5.505644000	12.182617000
5	0.686917000	6.076209000	14.682570000
1	0.330253000	6.778688000	15.569282000
5	2.043565000	3.540653000	12.659332000
1	2.542701000	2.611473000	12.118603000
5	2.567974000	5.775599000	14.541030000
1	3.403754000	6.283792000	15.210395000
5	0.006987000	4.321854000	14.637282000
1	-0.819401000	3.895193000	15.373120000
5	2.802099000	4.077601000	14.151498000
1	3.836124000	3.570244000	14.465027000
5	1.612773000	4.525599000	15.306983000
1	1.769961000	4.347430000	16.477790000
5	1.309984000	3.230555000	14.216613000
1	1.243709000	2.095306000	14.582318000
1	-0.395255000	7.875151000	12.887361000
1	-1.845879000	5.506516000	13.739921000
1	-0.549816000	3.019091000	12.403346000
3	-2.256089000	6.843841000	12.259711000
3	2.797949000	2.489967000	16.020101000

$\{Li_2[B_{15}H_{14}]\}^+$_eq_MeCN E = -396.245106803 Ha

5	-0.016397000	6.784061000	13.089873000
5	-0.324011000	5.474247000	11.990601000
1	-1.224878000	5.402223000	11.222981000
5	1.125004000	6.388100000	11.869651000
1	1.379178000	7.102002000	10.947153000
5	-0.415995000	5.258363000	13.646604000
5	1.200338000	4.641216000	11.623365000
1	1.274631000	4.281978000	10.496167000
5	1.716539000	6.840127000	13.470954000
1	2.134845000	7.944590000	13.573348000
5	0.181860000	3.743317000	12.917610000
5	2.567609000	5.367425000	12.681741000
1	3.584221000	5.604629000	12.116735000
5	0.711197000	5.999335000	14.813797000
1	0.304504000	6.680044000	15.692066000
5	2.002717000	3.580112000	12.660370000
1	2.515477000	2.700661000	12.053800000
5	2.518766000	5.781293000	14.510708000
1	3.369377000	6.344184000	15.113904000
5	0.056489000	4.215412000	14.754660000
1	-0.829108000	3.837855000	15.450378000
5	2.809197000	4.106991000	14.115330000
1	3.881076000	3.643932000	14.366511000
5	1.683267000	4.484154000	15.361156000
1	1.911942000	4.295521000	16.516225000
5	1.373833000	3.167637000	14.253233000
1	1.380254000	2.029749000	14.609664000
1	-0.669094000	7.766608000	13.206795000
1	-0.577485000	2.921027000	12.533767000
3	-0.627693000	7.719685000	10.770235000
3	2.988942000	2.477818000	15.978212000

$Li_2[B_{16}H_{16}]$_MeCN E = -422.505672254 Ha

5	0.175294000	1.282969000	0.949291000
5	-0.874640000	-1.439456000	1.491850000
5	-1.397143000	0.278112000	1.061803000
5	1.555683000	0.140824000	1.260243000
1	0.106278000	2.351851000	1.493564000
1	-1.301194000	-2.159468000	2.336215000

5	0.024318000	-0.077957000	2.032360000
5	-1.653178000	0.304517000	-0.791982000
5	-1.927164000	-1.134861000	0.156014000
5	0.848370000	-1.456459000	1.183041000
5	1.516475000	1.127904000	-0.158827000
5	-0.078691000	1.353639000	-0.886035000
5	0.539239000	-1.348042000	-1.458885000
5	1.209740000	0.261596000	-1.604657000
5	-1.213240000	-1.352728000	-1.398690000
1	-0.022691000	0.144822000	3.210661000
1	-2.257729000	0.862195000	1.641149000
1	2.481637000	0.336349000	1.995918000
1	-2.626013000	0.899538000	-1.130654000
1	-3.046527000	-1.513330000	0.290289000
1	1.464312000	-2.305407000	1.744227000
1	2.308639000	2.011187000	-0.220158000
1	-0.295230000	2.453575000	-1.284009000
1	1.001882000	-2.139719000	-2.235397000
1	1.963105000	0.481873000	-2.512466000
1	-1.798913000	-2.031167000	-2.179807000
3	1.279730000	1.701160000	3.104549000
3	0.979700000	-0.741117000	-3.798206000
5	-0.455661000	0.068581000	-2.025704000
1	-0.714912000	0.346953000	-3.163031000
5	1.805188000	-0.686050000	-0.257589000
1	2.883085000	-1.166458000	-0.406101000
5	-0.397099000	-2.130206000	-0.047412000
1	-0.385859000	-3.319778000	-0.091055000

$\{Li_2[B_{16}H_{15}]\}^+$_ap_MeCN E = -421.698936244 Ha

5	0.174033000	1.259396000	0.789445000
5	-0.940945000	-1.403404000	1.572884000
5	-1.420909000	0.271823000	1.041394000
5	1.567413000	0.158790000	1.235131000
1	0.101370000	2.365832000	1.238884000
1	-1.317598000	-2.032556000	2.502544000
5	0.015733000	-0.027388000	1.971143000
5	-1.577031000	0.231109000	-0.817351000
5	-1.984993000	-1.168523000	0.202199000
5	0.804980000	-1.461212000	1.118994000
5	1.598796000	1.034026000	-0.258922000
5	0.024187000	1.196711000	-1.050622000
5	0.475869000	-1.354318000	-0.956987000
5	1.310561000	0.015365000	-1.631765000
5	-1.216556000	-1.530141000	-1.315817000
1	-0.006579000	0.245030000	3.136369000
1	-2.270312000	0.906466000	1.572847000
1	2.446031000	0.365214000	2.016008000
1	-2.519530000	0.809083000	-1.246657000
1	-3.124982000	-1.473476000	0.305694000
1	1.413779000	-2.323550000	1.656315000
1	2.380229000	1.916258000	-0.368829000
1	-0.114576000	2.250539000	-1.600741000
1	1.973478000	0.061778000	-2.617461000
1	-1.700845000	-2.211684000	-2.150032000
3	1.230168000	1.793184000	3.102993000
3	0.558416000	1.634514000	-3.508843000
5	-0.387979000	-0.176680000	-2.021375000
1	-0.634191000	-0.061662000	-3.180222000
5	1.949535000	-0.728139000	-0.201842000
1	2.989010000	-1.284661000	-0.275520000
5	-0.569202000	-2.275858000	0.114873000
1	-0.532448000	-3.456704000	0.161173000

$\{Li_2[B_{16}H_{15}]\}^+$_eq_MeCN E = -421.696171733 Ha

5	0.284842000	1.324702000	1.040853000
5	-0.778533000	-1.209258000	1.262126000
5	-1.324709000	0.436552000	0.878847000
5	1.597445000	0.121729000	1.306472000
1	0.236918000	2.382857000	1.592627000
5	0.039957000	-0.050537000	2.077355000
5	-1.754436000	0.220372000	-0.906171000
5	-1.944909000	-1.160227000	0.130923000
5	0.869050000	-1.458905000	1.243428000
5	1.517875000	1.091686000	-0.130774000
5	-0.195243000	1.279414000	-0.748591000
5	0.527886000	-1.363262000	-1.522836000
5	1.159271000	0.251940000	-1.572552000
5	-1.226132000	-1.420482000	-1.440815000
1	-0.153327000	0.121688000	3.238841000
1	-2.179877000	1.028472000	1.442636000
1	2.546444000	0.308047000	2.006409000
1	-2.742713000	0.770897000	-1.252418000
1	-3.032545000	-1.527972000	0.423400000
1	1.367659000	-2.365147000	1.814807000
1	2.305174000	1.968222000	-0.254926000
1	-0.447275000	2.381434000	-1.109822000
1	1.005235000	-2.134809000	-2.301085000
1	1.901028000	0.542536000	-2.467174000
1	-1.804580000	-2.127750000	-2.194474000
3	1.428336000	1.763219000	3.251571000
3	1.032104000	-0.575719000	-3.906888000
5	-0.490522000	0.022018000	-2.042599000
1	-0.710671000	0.354212000	-3.169499000
5	1.784680000	-0.720337000	-0.253104000
1	2.846316000	-1.225422000	-0.404414000
5	-0.351297000	-2.142871000	-0.073971000
1	-0.315466000	-3.322389000	0.006111000

$Li_2[B_{17}H_{17}]$_MeCN E = -447.973689147 Ha

5	0.303504000	1.408673000	0.835999000
5	-1.162784000	-1.708587000	1.207310000
5	-2.089555000	-0.369199000	0.586993000
5	1.235538000	0.072922000	1.456437000
1	0.132251000	2.407854000	1.469850000
1	-1.486827000	-2.290517000	2.190182000
5	-0.570348000	-0.027335000	1.519282000
5	-1.130851000	0.964265000	-0.178707000
5	-2.045711000	-1.870601000	-0.295420000
5	0.638610000	-1.581992000	0.997560000
5	1.835818000	1.012907000	0.107587000
5	0.463998000	1.442934000	-0.895367000
5	0.820555000	-1.544150000	-1.030966000
5	1.490503000	0.112417000	-1.343041000
5	-1.937424000	-0.341361000	-1.145154000
1	-0.919087000	0.288544000	2.628254000
1	-3.077113000	0.024105000	1.134973000
1	1.748795000	0.113492000	2.526204000
1	-1.847972000	1.931321000	-0.197251000
1	-2.995486000	-2.573368000	-0.394519000
1	1.143855000	-2.366606000	1.739541000
1	2.782892000	1.719895000	0.202155000
1	0.423627000	2.452189000	-1.518644000
1	1.453595000	-2.298846000	-1.724991000
1	2.192895000	0.160728000	-2.309997000
1	-2.803343000	0.056085000	-1.852783000
3	-2.062893000	1.907538000	1.906298000
3	0.921495000	-1.292488000	-3.476452000
5	-0.271212000	0.026988000	-1.756534000
1	-0.361254000	0.312980000	-2.922454000
5	1.973249000	-0.731793000	0.102963000
1	3.024199000	-1.277644000	0.186610000
5	-0.424814000	-2.513219000	-0.146458000
1	-0.210861000	-3.681075000	-0.149576000
5	-0.900942000	-1.664525000	-1.592604000
1	-1.013694000	-2.220988000	-2.644862000

$\{Li_2[B_{17}H_{16}]\}^+$_ap_MeCN E = -447.151782972 Ha

5	0.308914000	1.431959000	0.858486000
5	-1.162113000	-1.723707000	1.211513000
5	-2.072956000	-0.373092000	0.581983000
5	1.226342000	0.102633000	1.526065000
1	0.132188000	2.427004000	1.487764000
1	-1.482322000	-2.300696000	2.194642000
5	-0.581136000	-0.017963000	1.539885000
5	-1.103881000	0.960119000	-0.173457000
5	-2.026862000	-1.874580000	-0.302571000
5	0.624614000	-1.602890000	1.033897000
5	1.858164000	1.048214000	0.144019000
5	0.492408000	1.462664000	-0.880638000
5	0.836859000	-1.566661000	-1.050662000
5	1.537159000	0.150030000	-1.362269000
5	-1.902367000	-0.343620000	-1.151041000
1	-0.934226000	0.286490000	2.644334000
1	-3.068205000	0.011881000	1.117261000
1	1.788832000	0.104919000	2.561920000
1	-1.812572000	1.928715000	-0.207940000
1	-2.980927000	-2.564503000	-0.416213000
1	1.223806000	-2.353318000	1.734262000
1	2.847140000	1.679512000	0.259705000
1	0.457663000	2.468970000	-1.501934000
1	1.554910000	-2.287779000	-1.675540000
1	2.290410000	0.169402000	-2.272082000
1	-2.758195000	0.051368000	-1.867632000
3	-2.203888000	1.935167000	1.893458000
3	0.482115000	-1.430419000	-3.809414000
5	-0.233425000	0.026213000	-1.762213000
1	-0.309748000	0.316674000	-2.924260000
5	1.692088000	-0.629060000	0.104361000
5	-0.418356000	-2.534877000	-0.140193000
1	-0.183294000	-3.695201000	-0.138502000
5	-0.870986000	-1.675956000	-1.589931000
1	-0.999144000	-2.248426000	-2.631253000

$\{Li_2[B_{17}H_{16}]\}^+$_eq_MeCN E = -447.185779009 Ha

5	0.320973000	1.426232000	0.837105000
5	-1.141876000	-1.723894000	1.233518000
5	-2.115315000	-0.386762000	0.581347000
5	1.235624000	0.046056000	1.484202000
1	0.166349000	2.407849000	1.491990000
1	-1.373211000	-2.231000000	2.274226000
5	-0.605343000	0.020668000	1.473184000
5	-1.124486000	0.950026000	-0.167191000
5	-2.036686000	-1.913461000	-0.270978000
5	0.486083000	-1.346674000	0.777084000
5	1.866562000	0.992481000	0.140214000
5	0.495309000	1.441293000	-0.879388000
5	0.794835000	-1.499530000	-1.074483000
5	1.516269000	0.128436000	-1.340969000
5	-1.946460000	-0.375788000	-1.135995000
1	-0.924171000	0.288869000	2.590214000
1	-3.092709000	-0.019105000	1.151312000
1	1.642985000	0.014161000	2.591798000
1	-1.837227000	1.915632000	-0.201722000
1	-2.979143000	-2.619626000	-0.367035000
1	2.812232000	1.693555000	0.241281000
1	0.470125000	2.450589000	-1.496050000
1	1.438938000	-2.275714000	-1.710323000
1	2.237957000	0.155347000	-2.286844000
1	-2.816471000	0.005110000	-1.841361000
3	-2.131597000	2.081758000	1.869912000
3	0.826326000	-1.182895000	-3.663572000
5	-0.258508000	0.009659000	-1.744652000
1	-0.358096000	0.307471000	-2.903771000
5	1.981627000	-0.764155000	0.124514000
1	2.962374000	-1.418228000	0.188106000
5	-0.395557000	-2.534051000	-0.126004000
1	-0.054326000	-3.664140000	-0.129259000
5	-0.918491000	-1.683870000	-1.597625000
1	-1.015539000	-2.268149000	-2.629907000
